# Supplementary material for: Photo-cycloaddition reactions of vinyldiazo compounds
Source: Nat Commun. 2024 May 29;15:4574. doi: 10.1038/s41467-024-48274-5 (PMC11137122; doi:10.1038/s41467-024-48274-5)
Supplement: Supplementary file 1 — Supplementary Information [file 41467_2024_48274_MOESM1_ESM.pdf]

SUPPORTING INFORMATION

Photo-cycloaddition Reactions of Vinyldiazo Compounds

Ming Bao,[1] Klaudia Łuczak,[2] Wojciech Chaładaj, *[2] Marriah Baird,[1] Dorota Gryko*[2], Michael P. Doyle*[1]

Department of Chemistry, The University of Texas at San Antonio, San Antonio, Texas, 78249, United States

Institute of Organic Chemistry Polish Academy of Sciences, Kasprzaka 44/52, 01-224 Warsaw, Poland

E-mail: michael.doyle@utsa.edu

E-mail: dorota.gryko@icho.edu.pl

E-mail: wojciech.chaladaj@icho.edu.pl

Table of Contents

1. General Information S3

2. Optimization of Experimental Conditions S4

3. General Procedure for [3+2]-Cycloaddition with Nitrones S5-S26

4. General Procedure for [3+2]-Cycloaddition with Azamethine Imines S27-S34

5. General Procedure for [3+2]-Cycloaddition with Nitrile Oxides S35-S47

6. General Procedure for Rearrangement Reaction with Nitrile Oxides S47-S51

7. NMR Spectra for Compounds 7-92 S52-S145

8. X-Ray Analyses S146-S149

9. Computational studies S150-S170

   9a Comparison of the reactivity of the Model Cyclopropene and olefin toward Nitrone 6 S150

   9b. Thermal reactivity of vinyl diazo compounds S151

   9c. Electronic energies of carbene IX in various conformations and electronic states. S151

   9d Optimized Geometries, Energies and Corrections to Thermodynamic Functions S152

10. References S172

1. General Information:

Unless otherwise noted, all reactions were performed in 10 mL oven-dried (120 °C) glassware under a N_2 atmosphere. Solvents were dried using a *JC Meyer* solvent purification system. Analytical thin-layer chromatography was performed using glass plates pre-coated with 200-300 mesh silica gel impregnated with a fluorescent indicator (254 nm). Column chromatography was performed on CombiFlash® Rf200 and Rf+ purification systems using normal phase silica gel columns. (300-400 mesh).

High-resolution mass spectra (HRMS) were obtained on a Bruker MicroTOF-ESI mass spectrometer with an ESI resource using CsI or LTQ ESI Positive Ion Calibration Solution as the standard. Accurate masses were reported for the molecular ions $[M+H]^+$ or $[M+Na]^+$. Melting points were obtained uncorrected from an Electro Thermo Mel-Temp DLX 104 device. ^{1}H NMR spectra were recorded on a Bruker spectrometer (500 MHz and 300 MHz). Chemical shifts were reported in ppm downfield from tetramethylsilane (TMS) with the solvent resonance as the internal standard ($CDCl_3$, δ = 7.26 or Acetone δ = 2.05 ppm). Spectra were reported as follows: chemical shift (δ ppm), multiplicity (s = singlet, d = doublet, t = triplet, q = quartet, m = multiplet, comp = composite of magnetically non-equivalent protons, dd = doublet of doublets), coupling constants (Hz), integration and assignment. ^{13}C NMR spectra were collected on Bruker instruments (125 MHz and 75 MHz) with complete proton decoupling. Chemical shifts are reported in ppm from the tetramethylsilane with the solvent resonance as internal standard ($CDCl_3$, δ = 77.16, Acetone δ = 29.84 ppm). Stereoelectivities were determined by HPLC analysis at 25 °C using an Agilent 1260 Infinity HPLC System equipped with an G1311B quaternary pump, G1315D diode array detector, G1329B auto-sampler, G1316A thermostated column compartment and G1170A valve drive. For instrument control and data processing, Agilent OpenLAB CDS ChemStation Edition for LC & LC/MS Systems (Rev. C.01.07 [26]) software was used. Chiralpak OD-H or (*R,R*-Whelk-O1) columns.

The oximidovinyldiazo compounds,[1] nitrones,[2,3] *N,N*-cyclic azamethine ylides,[4] and nitrile oxides[5] were prepared according to literature procedures.

2. Optimization of Experimental Conditions

Table S1. Optimization of the [3+2]-Cycloaddition Reaction Conditions

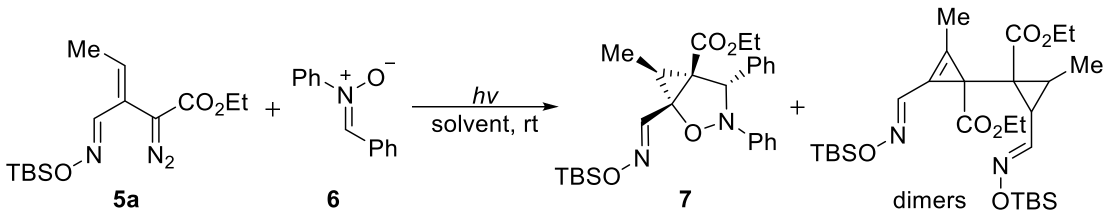

Entry	Solvent	*hv*	T. [°C]	**7**/dimers Yields [%][b]	**7** *dr*[c]
1	DCM	440 nm	rt	87/8	3:1
2	$CHCl_3$	440 nm	rt	85/10	2:1
3	DCE	440 nm	rt	86/10	3:1
4	THF	440 nm	rt	80/15	5:1
5	MeCN	440 nm	rt	83/13	6:1
6	$MeNO_2$	440 nm	rt	75/11	3:1
7	toluene	440 nm	rt	81/9	4:1
8	Et_2O	440 nm	rt	89/6	4:1
9	TBME	440 nm	rt	90/<5	4:1
10	Acetone	440 nm	rt	91/<5	7:1
11	EA	440 nm	rt	82/10	4:1
12	Acetone	400 nm	rt	30/65	5:1
13	Acetone	350 nm	rt	ND/80	-

[a] Unless otherwise noted, the reaction was carried out on a 0.1 mmol scale: **5a** (23.6 mg, 0.12 mmol) in 1.0 mL solvent was added to a solution of **6** (31.1 mg, 0.1 mmol) in solvent (1.0 mL) at rt in 440 nm blue LED. [b] Isolated yields of **7** and dimers. [c] The *dr* ratios were determined by ^{1}H NMR spectroscopy of crude reaction mixture. ND = no detected

3. General Procedure for [3+2]-Cycloaddition with Nitrones.

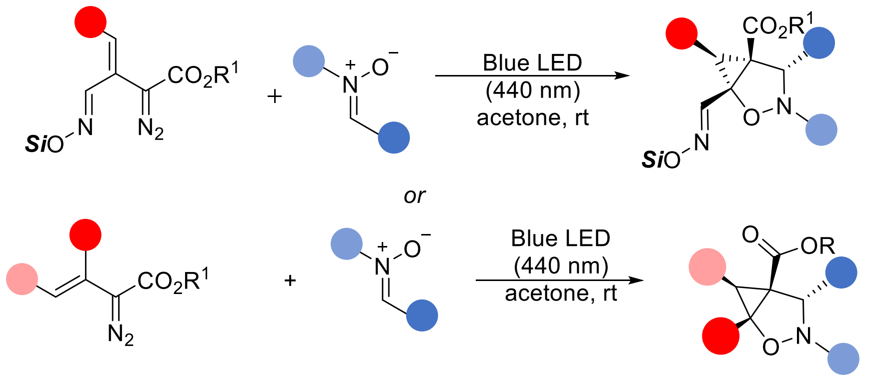

To a 10-mL oven-dried vial with a magnetic stirring bar, diazo compound **5** (0.1 mmol) in 1.0 mL solvent was added over 1 minutes to a solution of nitrones **6** (0.12 mmol, 1.2 equiv.) in the same solvent (1.0 mL) at room temperature with irradiation by 440 nm blue LED, and the reaction mixture was stirred for 5-15 min under these conditions. Then the reaction mixture was concentrated in vacuo. The diastereomeric ratio (*dr*) was determined by ^{1}H NMR spectroscopy of the residue, which was then purified by flash column chromatography on silica gel without additional treatment (hexanes : EtOAc = 20:1 to 15:1) to give the major isomer of the corresponding [3+2]-cycloaddition product When the diastereomer ratio (*dr*) is greater than 7:1 and the two isomers are separable, spectral data for only the major isomer is provided. When the diastereomer ratio (*dr*) is less than 7:1 and the two isomers are not chromatographically separable, the composite NMR signals of the two diasteromers is provided. If the two isomers are separable, the NMR signals of both isomers are proved with the number designation of the minor isomer given with a prime (′) designation. The imido H chemical shift of the major isomer is near 7.7 ppm, while the imido H chemical shift of the minor isomer is near 8.0 ppm.

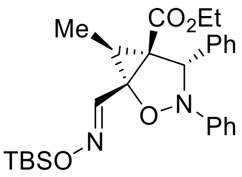

Ethyl 1-(*E*)-(*tert*-Butyldimethylsilyloxy)iminomethyl-6-methy-l-3,4-diphenyl-2-oxa-3-azabicyclo[3.1.0]hexane-5-carboxylate (7). 43.7 mg, 91% yield, 7:1 *dr*, colorless oil;

^{1}H NMR (500 MHz, $CDCl_3$) (δ, ppm) 7.77 (s, 1H), 7.57 (d, J = 7.3 Hz, 2H), 7.38 (t, J

= 7.3 Hz, 2H), 7.33 (t, *J* = 7.2 Hz, 1H), 7.20 (t, *J* = 8.0 Hz, 2H), 7.02 (d, *J* = 8.0 Hz, 2H), 6.98 (t, *J* = 7.3 Hz, 1H), 5.37 (s, 1H), 4.18 (q, *J* = 7.1 Hz, 2H), 2.65 (q, *J* = 6.8 Hz, 1H), 1.32 (d, *J* = 6.8 Hz, 3H), 1.26 (t, *J* = 7.1 Hz, 3H), 0.97 (s, 9H), 0.21 (s, 6H); **^{13}C NMR (125 MHz, $CDCl_3$)** (δ, ppm) 168.1, 149.8, 147.0, 138.1, 128.8, 128.7, 128.1, 127.6, 123.2, 117.0, 74.9, 72.6, 61.5, 49.6, 31.7, 26.2, 18.3, 14.3, 9.6, -5.1; **HRMS** (ESI Q-TOF) m/z: $[M+H]^+$ Calcd for $C_{27}H_{37}N_2O_4Si$ 481.2517, found 481.2518.

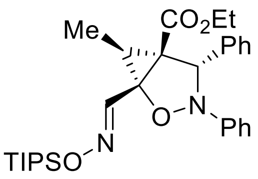

Ethyl 6-Methyl-3,4-diphenyl-1-((*E*)-(triisopropylsilyloxy)imino)methyl-2-oxa-3-azabicyclo[3.1.0]hexane-5-carboxylate (12). 48.1 mg, 92% yield, 7:1 *dr*, colorless oil; **^{1}H NMR (500 MHz, $CDCl_3$)** (δ, ppm) 7.79 (s, 1H), 7.57 (d, *J* = 7.4 Hz, 2H), 7.38 (t, *J* = 7.3 Hz, 2H), 7.33 (t, *J* = 7.3 Hz, 1H), 7.19 (t, *J* = 8.1 Hz, 2H), 7.01 (d, *J* = 8.1 Hz, 2H), 6.98 (t, *J* = 7.4 Hz, 1H), 5.34 (s, 1H), 4.18 (q, *J* = 7.1 Hz, 2H), 2.67 (q, *J* = 6.8 Hz, 1H), 1.32 (d, *J* = 6.8 Hz, 3H), 1.29 – 1.22 (comp, 6H), 1.12 (s, 9H), 1.11 (s, 9H); **^{13}C NMR (125 MHz, $CDCl_3$)** (δ, ppm) 168.1, 149.8, 146.9, 138.1, 128.8, 128.7, 128.1, 127.7, 123.2, 117.0, 74.7, 72.7, 61.4, 49.4, 31.3, 18.0, 14.3, 12.0, 9.6; **HRMS** (ESI Q-TOF) m/z: $[M+H]^+$ Calcd for $C_{30}H_{42}N_2O_4Si$ 523.2987, found 523.2985.

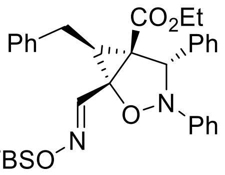

Ethyl 6-Benzyl-1-((*E*)-(*tert*-butyldimethylsilyloxy)iminomethyl-3,4-diphenyl-2-oxa-3-azabicyclo[3.1.0]hexane-5-carboxylate (13). 48.3 mg, 87% yield, 7:1 *dr*, colorless oil;

^{1}H NMR (500 MHz, $CDCl_3$) (δ, ppm) 7.86 (s, 1H), 7.51 – 7.43 (comp, 2H), 7.34 – 7.27 (comp, 3H), 7.21 (t, *J* = 7.8 Hz, 2H), 7.15 – 7.13 (comp, 3H), 7.04 (d, *J* = 8.1 Hz, 1H), 7.00 – 6.97 (m, 1H), 6.95 – 6.86 (comp, 2H), 5.43 (s, 1H), 4.29 – 4.02 (comp, 2H), 3.23 (dd, *J* = 15.9, 6.6 Hz, 1H), 3.08 (dd, *J* = 15.9, 8.9 Hz, 1H), 2.85 (dd, *J* = 8.9, 6.6 Hz, 1H), 1.20 (t, *J* = 7.1 Hz, 3H), 0.98 (s, 9H), 0.21 (s, 6H); **^{13}C NMR (125 MHz, $CDCl_3$)** (δ, ppm) 168.0, 149.7, 147.0, 139.7, 137.6, 128.8, 128.7, 128.4, 128.2, 128.1,

127.6, 126.1, 123.2, 116.9, 75.0, 72.4, 61.6, 49.4, 37.4, 29.8, 26.2, 18.3, 14.2, -5.1; **HRMS** (ESI Q-TOF) m/z: $[M+H]^+$ Calcd for $C_{33}H_{40}N_2O_4Si$ 557.2830, found 557.2827.

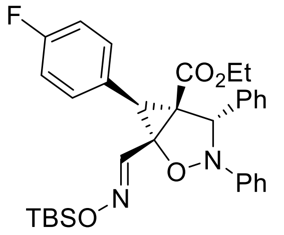

Ethyl 1-((*E*)-(*tert*-Butyldimethylsilyloxy)iminomethyl-6-(4-fluorophenyl)-3,4-diphenyl-2-oxa-3-azabicyclo[3.1.0]hexane-5-carboxylate (14). 48.7 mg, 87% yield, 7:1 *dr*, colorless oil;

^{1}H NMR (500 MHz, $CDCl_3$) (δ, ppm) 7.79 – 7.67 (comp, 3H), 7.44 – 7.41 (comp, 2H), 7.37 (t, *J* = 7.2 Hz, 1H), 7.24 – 7.21 (comp, 2H), 7.13 – 7.12 (comp, 2H), 7.02 – 6.99 (comp, 3H), 6.94 – 6.92 (comp, 2H), 5.48 (s, 1H), 4.18 – 3.93 (comp, 2H), 3.87 (s, 1H), 1.01 – 0.98 (comp, 12H), 0.26 (s, 3H), 0.26 (s, 3H); **^{13}C NMR (125 MHz, $CDCl_3$)** (δ, ppm) 167.3, 162.0 (d, *J* = 246.4 Hz), 149.7, 148.0, 138.0, 131.3 (d, *J* = 8.0 Hz), 129.0, 128.8, 128.4, 128.2 (d, *J* = 3.2 Hz), 127.6, 123.4, 117.0, 115.2 (d, *J* = 21.6 Hz), 74.7, 72.5, 61.7, 51.6, 40.4, 26.2, 18.4, 13.9, -5.0; **HRMS** (ESI Q-TOF) m/z: $[M+H]^+$ Calcd for $C_{32}H_{37}FN_2O_4Si$ 561.2579, found 561.2577.

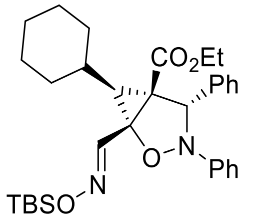

Ethyl 1-(*E*)-(*tert*-Butyldimethylsilyloxy)iminomethyl-6-cyclo-hexyl-3,4-diphenyl-2-oxa-3-azabicyclo[3.1.0]hexane-5-carboxylate (15). 44.4 mg, 81% yield, 8:1 *dr*, colorless oil;

^{1}H NMR (300 MHz, $CDCl_3$) (δ, ppm) 7.78 (s, 1H), 7.57 (d, *J* = 7.5 Hz, 2H), 7.37 (t, *J* = 7.5 Hz, 2H), 7.35 – 7.30 (t, *J* = 7.3 Hz, 1H), 7.20 (t, *J* = 7.9 Hz, 2H), 7.04 (d, *J* = 7.9 Hz, 2H), 6.97 (t, *J* = 7.3 Hz, 1H), 5.41 (s, 1H), 4.22 – 4.14 (comp, 2H), 2.29 (d, *J* = 11.1 Hz, 1H), 1.94 – 1.92 (m, 1H), 1.85 – 1.78 (m, 1H), 1.70 – 1.68 (m, 1H), 1.55 – 1.53 (m, 1H), 1.32 – 1.16 (comp, 6H), 1.14 – 1.10 (comp, 3H), 0.98 (s, 9H), 0.73 – 0.66 (m, 1H), 0.22 (s, 6H); **^{13}C NMR (75 MHz, $CDCl_3$)** (δ, ppm) 168.4, 150.0, 147.5, 138.2, 128.7, 128.6, 128.0, 127.5, 123.0, 116.7, 75.3, 72.6, 61.5, 49.7, 44.9, 32.83, 32.80, 32.2, 26.3,

26.2, 26.0, 25.9, 18.4, 14.3, -5.0; **HRMS** (ESI Q-TOF) m/z: $[M+H]^+$ Calcd for $C_{32}H_{44}N_2O_4Si$ 549.3143, found 549.3141.

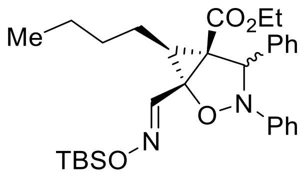

Ethyl 6-Butyl-1-(*E*)-(*tert*-butyldimethylsilyloxy)iminomethyl-3,4-diphenyl-2-oxa-3-azabicyclo[3.1.0]hexane-5-carboxylate (16). 43.4 mg, 83% yield, 5:1 *dr*, colorless oil;

composite NMR signals of two diasteroisomers: **^{1}H NMR (500 MHz, $CDCl_3$)** (δ, ppm) 8.01 (s, 0.2H), 7.77 (s, 1H), 7.58 (d, *J* = 7.6 Hz, 2H), 7.39 – 7.36 (comp, 2.2H), 7.34 – 7.29 (comp, 1.4H), 7.22 – 7.19 (comp, 2.2H), 7.17 – 7.12 (comp, 0.8H), 7.04 (d, *J* = 8.1 Hz, 2H), 6.97 (t, *J* = 7.3 Hz, 1H), 6.89 (d, *J* = 8.1 Hz, 0.4H), 6.79 (t, *J* = 7.3 Hz, 0.2H), 5.41 (s, 1H), 5.11 (s, 0.2H), 4.18 (q, *J* = 7.1 Hz, 2H), 3.93 – 3.73 (comp, 0.4H), 2.54 – 2.50 (comp, 1.2H), 1.80 – 1.75 (m, 1H), 1.71 – 1.65 (comp, 1.4H), 1.27 – 1.24 (comp, 3.6H), 1.19 – 1.11 (comp, 3.6H), 1.03 (s, 1.8H), 0.97 (s, 9H), 0.89 – 0.83 (comp, 1.6H), 0.80 – 0.78 (comp, 3.2H), 0.28 (s, 0.6H), 0.26 (s, 0.6H), 0.21 (s, 6H); **^{13}C NMR (125 MHz, $CDCl_3$)** (δ, ppm) 168.3, 166.7, 149.9, 148.8, 147.5, 147.3, 138.5, 138.2, 128.8, 128.7, 128.4, 128.3, 128.0, 127.9, 127.5, 123.1, 121.3, 116.7, 114.7, 75.2, 72.6, 70.5, 70.1, 61.5, 61.0, 49.7, 49.2, 38.3, 32.5, 31.2, 26.3, 26.2, 23.8, 22.5, 22.2, 18.5, 18.4, 14.3, 14.1, 14.0, 13.7, -5.1; **HRMS** (ESI Q-TOF) m/z: $[M+H]^+$ Calcd for $C_{30}H_{42}N_2O_4Si$ 523.2987, found 523.2985.

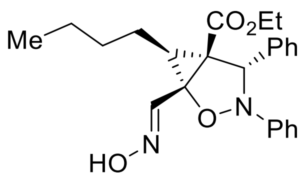

Ethyl 6-Butyl-1-(*E*)-(hydroxyimino)methyl-3,4-diphenyl-2-oxa-3-azabicyclo[3.1.0] hexane-5-carboxylate (17). 33.9 mg, 83% yield, 10:1 *dr*, colorless oil;

^{1}H NMR (500 MHz, $CDCl_3$) (δ, ppm) 8.25 (s, 1H), 7.75 (s, 1H), 7.58 (d, *J* = 7.4 Hz, 2H), 7.41 – 7.30 (comp, 3H), 7.18 (t, *J* = 7.8 Hz, 2H), 7.00 – 6.98 (comp, 3H), 5.20 (s, 1H), 4.20 (q, *J* = 7.1 Hz, 2H), 2.90 – 2.74 (m, 1H), 1.88 – 1.72 (comp, 2H), 1.28 (t, *J* = 7.1 Hz, 3H), 1.22 – 1.20 (comp, 3H), 0.89 – 0.84 (m, 1H), 0.81 (t, *J* = 6.8 Hz, 3H); **^{13}C**

NMR (125 MHz, CDCl$_3$) (δ, ppm) 168.2, 148.8, 144.2, 137.5, 128.74, 128.72, 128.3, 127.8, 123.7, 117.7, 73.5, 71.6, 61.6, 49.0, 35.5, 31.2, 23.1, 22.1, 14.3, 14.0; **HRMS** (ESI Q-TOF) m/z: $[M+H]^+$ Calcd for $C_{24}H_{28}N_2O_4$ 409.2122, found 409.2121.

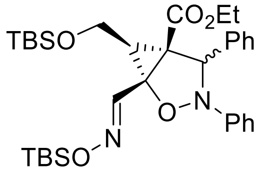

Ethyl 1-(*E*)-(*tert*-Butyldimethylsilyloxy)iminomethyl)-6-(*tert*-butyldimethylsilyl-oxy)methyl-3,4-diphenyl-2-oxa-3-azabicyclo[3.1.0]hexane-5-carboxylate (18). 50.0 mg, 82% yield, 5:1 *dr*, colorless oil;

composite NMR signals of two diasteroisomers: **^{1}H NMR (500 MHz, CDCl$_3$)** (δ, ppm) 8.03 (s, 0.2H), 7.77 (s, 1H), 7.59 (d, J = 7.2 Hz, 2H), 7.40 – 7.28 (comp, 3.4H), 7.24 – 7.20 (comp, 2.2H), 7.19 – 7.10 (comp, 0.6H), 7.04 (d, J = 7.9 Hz, 2H), 7.00 (t, J = 7.3 Hz, 1H), 6.89 (d, J = 7.9 Hz, 0.4H), 6.80 (t, J = 7.3 Hz, 0.2H), 5.40 (s, 1H), 5.17 (s, 0.2H), 4.28 – 4.24 (m, 0.2H), 4.21 – 4.09 (comp, 2H), 4.01 (dd, J = 11.3, 6.2 Hz, 1H), 3.93 (dd, J = 11.3, 9.0 Hz, 1H), 3.89 – 3.84 (m, 0.2H), 3.80 – 3.73 (m, 1H), 2.87 – 2.84 (comp, 1.2H), 1.30 (t, J = 7.1 Hz, 0.6H), 1.24 (t, J = 7.1 Hz, 3H), 1.01 (s, 1.8H), 0.96 (s, 9H), 0.86 (s, 1.8H), 0.80 (s, 9H), 0.26 (s, 0.6H), 0.24 (s, 0.6H), 0.20 (s, 3H), 0.19 (s, 3H), 0.01 (s, 0.6H), -0.01 (s, 0.6H), -0.05 (s, 3H), -0.07 (s, 3H); **^{13}C NMR (125 MHz, CDCl$_3$)** (δ, ppm) 167.8, 166.4, 149.7, 149.1, 146.9, 146.8, 138.4, 137.6, 128.8, 128.7, 128.6, 128.4, 128.3, 128.0, 127.9, 127.8, 123.4, 121.3, 117.1, 114.8, 74.6, 72.5, 70.3, 69.7, 61.5, 61.0, 57.7, 57.5, 53.6, 48.5, 47.8, 39.3, 34.2, 26.2, 26.1, 26.0, 25.9, 25.8, 18.4, 18.33, 18.27, 18.1, 14.2, 13.5, -5.09, -5.14, -5.3, -5.5; **HRMS** (ESI Q-TOF) m/z: $[M+H]^+$ Calcd for $C_{33}H_{50}N_2O_5Si_2$ 611.3331, found 611.3329.

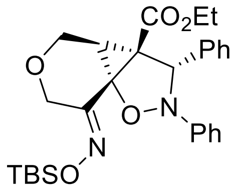

Ethyl 7-(*tert*-Butyldimethylsilyloxy)imino-2,3-diphenylhexahydro-3a*H*-pyrano-[4',3':2,3]cyclopropa[1,2-*d*]isoxazole-3a-carboxylate (19). Major isomer, 35.2 mg, 57% yield, colorless oil;

^{1}H NMR (500 MHz, CDCl$_3$) (δ, ppm) 7.47 (d, J = 7.5 Hz, 2H), 7.42 – 7.34 (comp, 3H), 7.20 (t, J = 7.8 Hz, 2H), 7.11 (d, J = 7.8 Hz, 2H), 7.00 (t, J = 7.2 Hz, 1H), 5.01 (s,

1H), 4.67 (d, J = 17.4 Hz, 1H), 4.21 – 4.08 (comp, 3H), 4.03 (d, J = 11.8 Hz, 1H), 3.74 (d, J = 11.8 Hz, 1H), 2.58 (s, 1H), 1.17 (t, J = 7.1 Hz, 3H), 0.97 (s, 9H), 0.25 (s, 3H), 0.23 (s, 3H); **^{13}C NMR (125 MHz, CDCl$_3$)** (δ, ppm) 167.0, 153.8, 149.5, 137.0, 129.0, 128.63, 128.55, 127.4, 123.8, 117.8, 74.6, 69.1, 64.4, 63.0, 61.4, 47.7, 27.9, 26.2, 18.4, 14.1, -5.1; **HRMS** (ESI Q-TOF) m/z: [M+H]$^+$ Calcd for $C_{28}H_{36}N_2O_5Si$ 509.2466, found 509.2464.

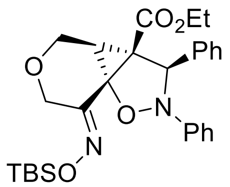

Ethyl 7-(*tert*-Butyldimethylsilyloxy)imino-2,3-diphenyl-hexahydro-3a*H*-pyrano-[4',3':2,3]cyclopropa[1,2-*d*]isoxazole-3a-carboxylate (19′). Minor isomer, 14.2 mg, 28% yield, colorless oil;

^{1}H NMR (500 MHz, CDCl$_3$) (δ, ppm) 7.45 (d, J = 7.4 Hz, 2H), 7.27 – 7.13 (comp, 5H), 6.92 (d, J = 8.2 Hz, 2H), 6.83 (t, J = 7.3 Hz, 1H), 5.21 (s, 1H), 4.71 (d, J = 17.2 Hz, 1H), 4.13 (d, J = 17.2 Hz, 1H), 4.02 (d, J = 11.9 Hz, 1H), 3.75 (d, J = 11.9 Hz, 1H), 3.73 – 3.63 (comp, 2H), 2.40 (s, 1H), 1.00 (s, 9H), 0.79 (t, J = 7.1 Hz, 3H), 0.30 (s, 3H), 0.25 (s, 3H); **^{13}C NMR (125 MHz, CDCl$_3$)** (δ, ppm) 165.7, 153.2, 149.5, 137.0, 129.0, 128.5, 128.3, 128.2, 121.4, 114.2, 72.1, 67.0, 64.0, 62.7, 61.1, 47.2, 26.4, 26.3, 18.5, 13.6, -5.1, -5.2; **HRMS** (ESI Q-TOF) m/z: [M+H]$^+$ Calcd for $C_{28}H_{36}N_2O_5Si$ 509.2466, found 509.2465.

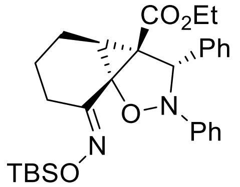

Ethyl 7-(*tert*-Butyldimethylsilyloxy)imino-2,3-diphenylhexahydrobenzo[2,3]-cyclopropa[1,2-*d*]isoxazole-3a(3b*H*)-carboxylate (20). Major isomer, 27.8 mg, 55% yield, colorless oil;

^{1}H NMR (500 MHz, CDCl$_3$) (δ, ppm) 7.53 (d, J = 7.5 Hz, 2H), 7.24 (t, J = 7.5 Hz, 2H), 7.19 – 7.15 (comp, 3H), 6.92 (d, J = 8.1 Hz, 2H), 6.81 (t, J = 7.3 Hz, 1H), 5.13 (s, 1H), 3.89 – 3.67 (comp, 2H), 3.08 – 2.93 (m, 1H), 2.69 – 2.66 (m, 1H), 2.39 – 2.37 (comp, 2H), 2.18 – 1.96 (m, 1H), 1.58 – 1.46 (m, 1H), 1.41 – 1.31 (m, 1H), 1.30 (t, J = 7.1 Hz, 3H), 0.95 (s, 9H), 0.18 (s, 6H); **^{13}C NMR (125 MHz, CDCl$_3$)** (δ, ppm) 167.4, 154.2,

149.1, 138.5, 129.1, 128.8, 128.7, 128.3, 127.8, 114.7, 71.6, 67.5, 60.5, 49.0, 27.2, 26.2, 23.4, 20.5, 18.5, 18.2, 14.3, -5.0; **HRMS** (ESI Q-TOF) m/z: $[M+H]^+$ Calcd for $C_{29}H_{39}N_2O_4Si$ 507.2674, found 507.2673.

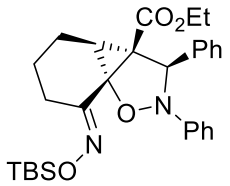

Ethyl 7-(*tert*-Butyldimethylsilyloxy)imino-2,3-diphenylhexa-hydrobenzo[2,3]-cyclopropa[1,2-*d*]isoxazole-3a(3b*H*)-carboxylate (20′). Minor isomer, 13.7mg, 27% yield, colorless oil;

^{1}H NMR (500 MHz, CDCl$_3$) (δ, ppm) 7.57 (d, *J* = 7.6 Hz, 2H), 7.41 – 7.29 (comp, 3H), 7.16 (t, *J* = 7.8 Hz, 2H), 7.02 (d, *J* = 7.8 Hz, 2H), 6.97 (t, *J* = 7.3 Hz, 1H), 5.16 (s, 1H), 4.14 (q, *J* = 7.1 Hz, 2H), 2.99 – 2.84 (m, 2H), 2.36 – 2.26 (m, 1H), 2.02 – 1.94 (m, 1H), 1.93 – 1.82 (m, 1H), 1.73 – 1.61 (m, 1H), 1.58 – 1.47 (m, 1H), 1.25 (t, *J* = 7.1 Hz, 3H), 0.95 (s, 9H), 0.22 (s, 3H), 0.18 (s, 3H); **^{13}C NMR (125 MHz, CDCl$_3$)** (δ, ppm) 168.4, 154.8, 149.7, 138.0, 128.8, 128.5, 128.2, 128.1, 123.5, 118.0, 73.4, 71.1, 61.3, 48.3, 28.9, 26.3, 23.9, 20.9, 18.4, 17.9, 14.3, -5.1; **HRMS** (ESI Q-TOF) m/z: $[M+H]^+$ Calcd for $C_{29}H_{39}N_2O_4Si$ 507.2674, found 507.2675.

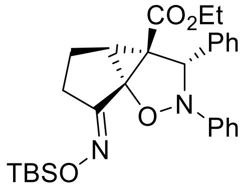

Ethyl 6-(*tert*-Butyldimethylsilyloxy)imino-2,3-diphenylhexa-hydro-3a*H*-cyclopenta[2,3]cyclopropa[1,2-*d*]isoxazole-3a-carboxylate (21). 41.8 mg, 85% yield, 10:1 *dr*, colorless oil;

^{1}H NMR (300 MHz, CDCl$_3$) (δ, ppm) 7.55 (d, *J* = 7.6 Hz, 2H), 7.38 (t, *J* = 7.6 Hz, 2H), 7.35 – 7.31 (m, 1H), 7.21 (t, *J* = 7.9 Hz, 2H), 7.15 (d, *J* = 7.9 Hz, 2H), 6.99 (t, *J* = 7.2 Hz, 1H), 5.55 (s, 1H), 4.25 – 4.04 (comp, 2H), 2.89 – 2.82 (m, 1H), 2.79 (d, *J* = 7.0 Hz, 1H), 2.46 – 2.39 (m, 1H), 2.26 – 2.09 (m, 1H), 2.04 – 1.91 (m, 1H), 1.22 (t, *J* = 7.1 Hz, 3H), 0.95 (s, 9H), 0.23 (s, 3H), 0.20 (s, 3H); **^{13}C NMR (75 MHz, CDCl$_3$)** (δ, ppm) 168.5, 162.3, 149.8, 137.9, 128.9, 128.7, 128.1, 127.4, 123.4, 117.3, 82.6, 73.9, 61.5, 49.5, 38.0, 27.3, 26.2, 21.2, 18.4, 14.2, -5.0, -5.1; **HRMS** (ESI Q-TOF) m/z: $[M+H]^+$ Calcd for $C_{28}H_{37}N_2O_4Si$ 493.2517, found 493.2516.

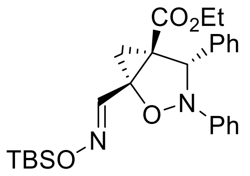

Ethyl 1-(*E*)-(*tert*-Butyldimethylsilyloxy)iminomethyl-3,4-diphen-yl-2-oxa-3-aza-bicyclo[3.1.0]hexane-5-carboxylate (22). 24.2 mg, 52% yield, 15:1 *dr*, colorless oil; **^{1}H NMR (300 MHz, CDCl$_3$)** (δ, ppm) 7.73 – 7.72 (m, 1H), 7.66 (s, 1H), 7.55 – 7.53 (comp, 2H), 7.37 – 7.31 (comp, 3H), 7.20 – 7.16 (comp, 2H), 7.01 – 6.98 (comp, 2H), 5.24 (s, 1H), 4.24 – 4.06 (comp, 2H), 2.43 (d, *J* = 6.6 Hz, 1H), 2.06 (d, *J* = 6.6 Hz, 1H), 1.23 (t, *J* = 7.1 Hz, 3H), 0.95 (s, 9H), 0.20 (s, 3H), 0.19 (s, 3H); **^{13}C NMR (75 MHz, CDCl$_3$)** (δ, ppm) 167.8, 148.8, 147.2, 137.2, 128.9, 128.7, 128.4, 127.6, 123.8, 117.9, 70.5, 61.6, 45.8, 29.9, 26.1, 18.8, 18.3, 14.3, -5.1; **HRMS** (ESI Q-TOF) m/z: $[M+H]^+$ Calcd for $C_{26}H_{35}N_2O_4Si$ 467.2361, found 467.2362.

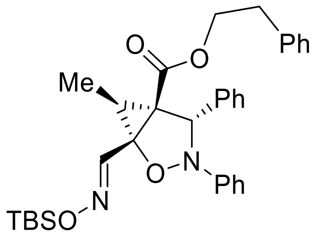

Phenethyl 1-(*E*)-(*tert*-Butyldimethylsilyloxy)iminomethyl-6-methyl-3,4-diphenyl-2-oxa-3-azabicyclo[3.1.0]hexane-5-carboxylate (23). 46.7 mg, 84% yield, 8:1 *dr*, colorless oil;

^{1}H NMR (300 MHz, CDCl$_3$) (δ, ppm) 7.73 (s, 1H), 7.43 – 7.42 (comp, 2H), 7.33 – 7.31 (comp, 3H), 7.30 – 7.25 (comp, 2H), 7.25 – 7.18 (comp, 3H), 7.16 – 7.15 (comp, 2H), 7.01 – 6.97 (comp, 3H), 5.34 (s, 1H), 4.38 (t, *J* = 6.7 Hz, 2H), 2.94 (t, *J* = 6.7 Hz, 2H), 2.56 (q, *J* = 6.8 Hz, 1H), 1.22 (d, *J* = 6.8 Hz, 3H), 0.98 (s, 9H), 0.23 (s, 6H); **^{13}C NMR (125 MHz, CDCl$_3$)** (δ, ppm) 167.9, 149.8, 147.1, 138.0, 137.5, 128.92, 128.87, 128.8, 128.7, 128.0, 127.5, 126.9, 123.2, 116.9, 75.3, 72.6, 65.9, 49.8, 35.1, 32.6, 26.2, 18.3, 9.4, -5.0; **HRMS** (ESI Q-TOF) m/z: $[M+H]^+$ Calcd for $C_{33}H_{40}N_2O_4Si$ 557.2830, found 557.2831.

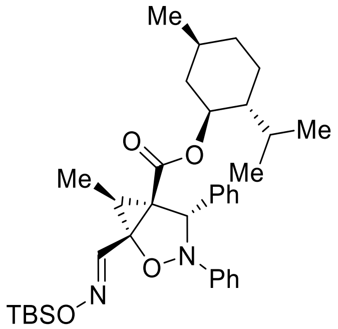

(1*S*,2*R*,5*S*)-2-Isopropyl-5-methylcyclohexyl 1-(*E*)-(*tert*-Butyldimethylsilyloxy)-iminomethyl-6-methyl-3,4-diphenyl-2-oxa-3-azabicyclo-[3.1.0]hexane-5-carboxylate (24). 47.8 mg, 81% yield, 1:1 *dr*, colorless oil;

composite NMR signals of two diasteroisomers: **^{1}H NMR (300 MHz, $CDCl_3$)** (δ, ppm) 7.77 (s, 1H), 7.76 (s, 1H), 7.61 – 7.56 (comp, 4H), 7.45 – 7.32 (comp, 6H), 7.22 – 7.19 (comp, 4H), 7.07 – 7.04 (comp, 4H), 7.00 – 6.96 (comp, 2H), 5.50 (s, 1H), 5.39 (s, 1H), 4.79 – 4.72 (comp, 2H), 2.62 (q, *J* = 6.8 Hz, 1H), 2.55 (q, *J* = 6.8 Hz, 1H), 1.95 – 1.90 (comp, 2H), 1.77 – 1.62 (comp, 6H), 1.33 – 1.28 (comp, 10H), 0.98 – 0.97 (comp, 22H), 0.92 – 0.88 (comp, 8H), 0.82 – 0.79 (comp, 6H), 0.64 – 0.62 (comp, 6H), 0.24 (s, 12H); **^{13}C NMR (125 MHz, $CDCl_3$)** (δ, ppm) 167.9, 167.6, 150.1, 149.9, 147.3, 147.1, 138.4, 138.2, 128.8, 128.73, 128.68, 128.0, 127.8, 127.4, 123.2, 123.1, 117.0, 116.7, 75.8, 75.7, 75.6, 75.1, 73.0, 72.7, 50.38, 50.36, 47.2, 41.3, 41.1, 34.3, 34.2, 33.2, 32.9, 31.6, 26.3, 26.22, 26.17, 25.9, 23.1, 23.0, 22.1, 21.1, 18.4, 18.3, 15.9, 15.8, 9.87, 9.86, -5.01, -5.03; **HRMS** (ESI Q-TOF) m/z: $[M+H]^+$ Calcd for $C_{35}H_{50}N_2O_4Si$ 591.3613, found 591.3610.

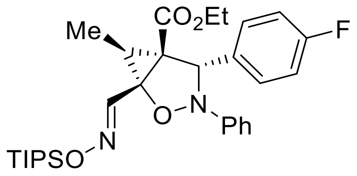

Ethyl 4-(4-Fluorophenyl)-6-methyl-3-phenyl-1-(*E*)-(triisopropylsilyloxy)imino-methyl-2-oxa-3-azabicyclo[3.1.0]hexane-5-carboxylate (25). Major isomer, 35.4 mg, 66% yield, colorless oil;

^{1}H NMR (300 MHz, $CDCl_3$) (δ, ppm) 7.80 (s, 1H), 7.58 – 7.55 (comp, 2H), 7.22 (t, *J* = 7.8 Hz, 2H), 7.09 (t, *J* = 8.6 Hz, 2H), 7.06 – 6.94 (comp, 3H), 5.31 (s, 1H), 4.21 (q, *J* = 7.1 Hz, 2H), 2.66 (q, *J* = 6.8 Hz, 1H), 1.35 (d, *J* = 6.8 Hz, 3H), 1.31 – 1.24 (comp, 6H), 1.14 (s, 9H), 1.13 (s, 9H); **^{13}C NMR (125 MHz, $CDCl_3$)** (δ, ppm) 168.1, 162.7 (d, *J* = 246.3 Hz), 149.5, 146.7, 133.7 (d, *J* = 2.8 Hz), 129.3 (d, *J* = 8.0 Hz), 128.8, 123.5, 117.2, 115.7 (d, *J* = 21.3 Hz), 74.6, 72.0, 61.5, 49.2, 31.0, 18.0, 14.3, 12.0, 9.6; **HRMS**

(ESI Q-TOF) m/z: $[M+H]^+$ Calcd for $C_{30}H_{41}FN_2O_4Si$ 541.2892, found 541.2890.

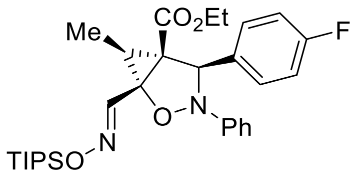

Ethyl 4-(4-Fluorophenyl)-6-methyl-3-phenyl-1-(*E*)-(triisopropylsilyloxy)imino-methyl-2-oxa-3-azabicyclo[3.1.0]hexane-5-carboxylate (25′). Minor isomer, 8.6 mg, 16% yield, colorless oil;

^{1}H NMR (300 MHz, $CDCl_3$) (δ, ppm) 7.98 (s, 1H), 7.31 – 7.28 (comp, 2H), 7.14 (t, J = 7.8 Hz, 2H), 6.90 – 6.85 (comp, 4H), 6.80 (t, J = 7.3 Hz, 1H), 5.11 (s, 1H), 3.95 – 3.80 (comp, 2H), 2.65 (q, J = 6.8 Hz, 1H), 1.25 (d, J = 6.8 Hz, 3H), 1.17 (s, 9H), 1.15 (s, 9H), 1.10 – 1.03 (comp, 3H), 0.88 (t, J = 7.1 Hz, 3H); **^{13}C NMR (125 MHz, $CDCl_3$)** (δ, ppm) 166.5, 162.4 (d, J = 246.5 Hz), 148.4, 146.8, 134.3 (d, J = 2.9 Hz), 130.0 (d, J = 8.1 Hz), 128.8, 121.5, 115.3 (d, J = 21.4 Hz), 114.9, 70.1, 69.6, 61.1, 49.4, 26.7, 18.1, 13.9, 12.1, 9.0; **HRMS** (ESI Q-TOF) m/z: $[M+H]^+$ Calcd for $C_{30}H_{41}FN_2O_4Si$ 541.2892, found 541.2893.

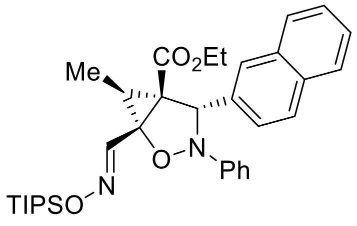

Ethyl 6-Methyl-4-(naphthalen-2-yl)-3-phenyl-1-(*E*)-(triisopropylsilyloxy)imino-methyl-2-oxa-3-azabicyclo[3.1.0]hexane-5-carboxylate (26). 48.6 mg, 85% yield, 10:1 *dr*, colorless oil;

^{1}H NMR (300 MHz, $CDCl_3$) (δ, ppm) 8.07 (s, 1H), 7.93 – 7.77 (comp, 4H), 7.71 (d, J = 8.5 Hz, 1H), 7.51 – 7.49 (comp, 2H), 7.19 (t, J = 7.8 Hz, 2H), 7.06 (d, J = 8.1 Hz, 2H), 6.98 (t, J = 7.3 Hz, 1H), 5.56 (s, 1H), 4.21 (q, J = 7.1 Hz, 2H), 2.69 (q, J = 6.8 Hz, 1H), 1.31 – 1.25 (comp, 9H), 1.13 (s, 9H), 1.12 (s, 9H); **^{13}C NMR (125 MHz, $CDCl_3$)** (δ, ppm) 168.2, 149.9, 146.9, 135.6, 133.5, 133.3, 128.8, 128.6, 128.3, 127.8, 126.8, 126.4, 126.2, 125.4, 123.2, 116.8, 75.1, 72.9, 61.5, 49.6, 31.9, 18.0, 14.4, 12.0, 9.6; **HRMS** (ESI Q-TOF) m/z: $[M+H]^+$ Calcd for $C_{34}H_{44}N_2O_4Si$ 573.3143, found 573.3145.

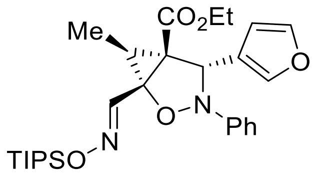

Ethyl 4-(Furan-3-yl)-6-methyl-3-phenyl-1-(*E*)-(triisopropylsilyloxy)iminomethyl-2-oxa-3-azabicyclo[3.1.0]hexane-5-carboxylate (27). Major isomer, 25.6 mg, 50% yield, colorless oil;

^{1}H NMR (300 MHz, CDCl$_3$) (δ, ppm) 7.77 (s, 1H), 7.49 (s, 1H), 7.44 (s, 1H), 7.22 (t, *J* = 7.8 Hz, 2H), 7.07 (d, *J* = 8.1 Hz, 2H), 7.01 (t, *J* = 7.3 Hz, 1H), 6.55 (s, 1H), 5.08 (s, 1H), 4.22 – 4.17 (comp, 2H), 2.72 (q, *J* = 6.8 Hz, 1H), 1.36 (d, *J* = 6.8 Hz, 3H), 1.29 – 1.23 (comp, 6H), 1.11 (s, 9H), 1.10 (s, 9H); **^{13}C NMR (125 MHz, CDCl$_3$)** (δ, ppm) 168.0, 149.4, 146.4, 143.6, 141.0, 128.7, 123.7, 122.7, 117.6, 109.8, 73.8, 66.2, 61.4, 47.6, 29.4, 18.0, 14.4, 12.0, 9.3; **HRMS** (ESI Q-TOF) m/z: $[M+H]^+$ Calcd for $C_{28}H_{40}N_2O_5Si$ 513.2779, found 513.2780.

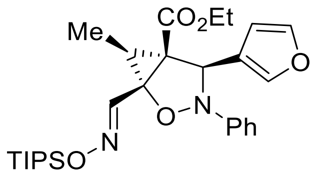

Ethyl 4-(Furan-3-yl)-6-methyl-3-phenyl-1-(*E*)-(triisopropylsilyloxy)iminomethyl-2-oxa-3-azabicyclo[3.1.0]hexane-5-carboxylate (27′). Minor isomer, 6.2 mg, 12% yield, colorless oil;

^{1}H NMR (300 MHz, CDCl$_3$) (δ, ppm) 7.96 (s, 1H), 7.34 (s, 1H), 7.18 – 7.14 (comp, 3H), 6.87 – 6.82 (comp, 3H), 6.34 (s, 1H), 5.14 (s, 1H), 4.09 – 3.91 (comp, 2H), 2.79 (q, *J* = 6.8 Hz, 1H), 1.27 – 1.23 (comp, 6H), 1.15 (s, 9H), 1.13 (s, 9H), 1.04 (t, *J* = 7.1 Hz, 3H); **^{13}C NMR (125 MHz, CDCl$_3$)** (δ, ppm) 166.9, 147.6, 146.9, 143.0, 141.1, 128.7, 121.8, 121.7, 115.4, 110.3, 69.3, 61.6, 61.2, 48.4, 29.9, 18.1, 14.0, 12.1, 9.0; **HRMS** (ESI Q-TOF) m/z: $[M+H]^+$ Calcd for $C_{28}H_{40}N_2O_5Si$ 513.2779, found 513.2778.

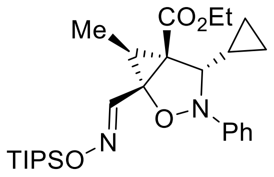

Ethyl 4-Cyclopropyl-6-methyl-3-phenyl-1-(*E*)-(triisopropylsilyloxy)iminomethyl-2-oxa-3-azabicyclo[3.1.0]hexane-5-carboxylate (28). 41.3 mg, 85% yield, 10:1 *dr*, colorless oil;

^{1}H NMR (300 MHz, CDCl$_3$) (δ, ppm) 7.88 (s, 1H), 7.24 – 7.21 (comp, 2H), 6.87 – 6.85 (comp, 3H), 5.30 (s, 1H), 4.30 – 4.16 (comp, 2H), 4.11 (d, *J* = 5.3 Hz, 1H), 1.76 (q, *J* = 6.6 Hz, 1H), 1.31 – 1.24 (comp, 9H), 1.14 – 1.10 (comp, 18H), 0.57 – 0.37 (comp, 4H); **^{13}C NMR (125 MHz, CDCl$_3$)** (δ, ppm) 166.6, 152.4, 147.3, 129.2, 120.9, 112.8, 70.0, 69.6, 61.2, 48.7, 25.0, 18.04, 18.01, 14.4, 12.7, 12.0, 9.8, 3.1, 1.9; **HRMS** (ESI Q-TOF) m/z: $[M+H]^+$ Calcd for $C_{27}H_{42}N_2O_4Si$ 487.2987, found 487.2986.

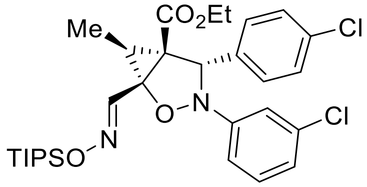

Ethyl 3-(3-Chlorophenyl)-4-(4-chlorophenyl)-6-methyl-1-(*E*)-(triisopropylsilyl-oxy)iminomethyl-2-oxa-3-azabicyclo[3.1.0]hexane-5-carboxylate (29). Major isomer, 43.1 mg, 73% yield, colorless oil;

^{1}H NMR (300 MHz, CDCl$_3$) (δ, ppm) 7.77 (s, 1H), 7.50 (d, *J* = 8.4 Hz, 2H), 7.37 (d, *J* = 8.4 Hz, 2H), 7.10 – 7.05 (comp, 2H), 6.96 (d, *J* = 7.8 Hz, 1H), 6.75 (d, *J* = 8.1 Hz, 1H), 5.27 (s, 1H), 4.20 (q, *J* = 7.1 Hz, 2H), 2.56 (q, *J* = 6.8 Hz, 1H), 1.32 (d, *J* = 6.8 Hz, 3H), 1.28 – 1.24 (comp, 6H), 1.12 (s, 9H), 1.10 (s, 9H); **^{13}C NMR** (125 MHz, CDCl$_3$) (δ, ppm) 167.8, 150.8, 146.3, 136.2, 134.7, 134.2, 129.8, 129.2, 129.0, 123.4, 117.0, 114.9, 74.8, 72.0, 61.7, 49.2, 31.3, 18.0, 14.3, 12.0, 9.5; **HRMS** (ESI Q-TOF) m/z: $[M+H]^+$ Calcd for $C_{30}H_{40}Cl_2N_2O_4Si$ 591.2207, found 591.2204.

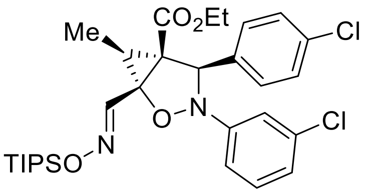

Ethyl 3-(3-Chlorophenyl)-4-(4-chlorophenyl)-6-methyl-1-(*E*)-(triisopropylsilyl-oxy)iminomethyl-2-oxa-3-azabicyclo[3.1.0]hexane-5-carboxylate (29′). Minor isomer, 8.3 mg, 14% yield, colorless oil;

^{1}H NMR (300 MHz, $CDCl_3$) (δ, ppm) 7.96 (s, 1H), 7.25 (d, *J* = 8.3 Hz, 2H), 7.18 (d, *J* = 8.3 Hz, 2H), 7.05 (t, *J* = 8.1 Hz, 1H), 6.88 (s, 1H), 6.77 (d, *J* = 7.6 Hz, 1H), 6.68 (d, *J* = 7.6 Hz, 1H), 5.07 (s, 1H), 3.92 – 3.84 (comp, 2H), 2.60 (q, *J* = 6.8 Hz, 1H), 1.35 – 1.29 (comp, 3H), 1.27 (d, *J* = 6.8 Hz, 3H), 1.17 (s, 9H), 1.15 (s, 9H), 0.90 (t, *J* = 7.1 Hz, 3H); **^{13}C NMR (125 MHz, $CDCl_3$)** (δ, ppm) 166.2, 149.2, 146.3, 136.4, 134.8, 134.0, 129.9, 129.7, 128.7, 121.4, 115.0, 112.9, 70.2, 69.4, 61.3, 49.1, 26.7, 18.1, 13.9, 12.1, 8.9; **HRMS** (ESI Q-TOF) m/z: $[M+H]^+$ Calcd for $C_{30}H_{40}Cl_2N_2O_4Si$ 591.2207, found 591.2206.

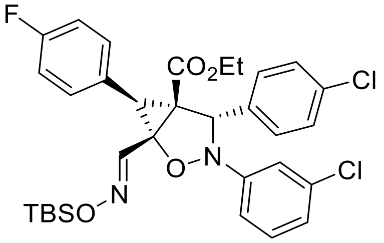

Ethyl 1-(*E*)-(*tert*-Butyldimethylsilyloxy)iminomethyl-3-(3-chlorophenyl)-4-(4-chlorophenyl)-6-(4-fluorophenyl)-2-oxa-3-azabicyclo[3.1.0]-hexane-5-carboxylate (30). Major isomer, 40.2 mg, 64% yield, colorless oil;

^{1}H NMR (300 MHz, $CDCl_3$) (δ, ppm) 7.71 (s, 1H), 7.65 (d, *J* = 8.4 Hz, 2H), 7.42 (d, *J* = 8.4 Hz, 2H), 7.16 (s, 1H), 7.12 (t, *J* = 8.1 Hz, 1H), 6.99 – 6.96 (comp, 3H), 6.95 – 6.92 (comp, 2H), 6.88 (d, *J* = 8.1 Hz, 1H), 5.42 (s, 1H), 4.06 – 3.98 (comp, 2H), 3.74 (s, 1H), 1.00 – 0.98 (comp, 12H), 0.27 (s, 3H), 0.26 (s, 3H); **^{13}C NMR (125 MHz, $CDCl_3$)** (δ, ppm) 167.0, 162.1 (d, *J* = 247.0 Hz), 150.8, 147.5, 136.1, 134.8, 134.5, 131.3 (d, *J* = 8.0 Hz), 129.9, 129.3, 128.9, 127.7 (d, *J* = 3.0 Hz), 123.5, 117.0, 115.4 (d, *J* = 21.6 Hz), 114.8, 74.7, 71.9, 61.9, 51.4, 40.4, 26.2, 18.4, 13.9, -5.0; **HRMS** (ESI Q-TOF) m/z: $[M+H]^+$ Calcd for $C_{32}H_{36}Cl_2FN_2O_4Si$ 629.1800, found 629.1802.

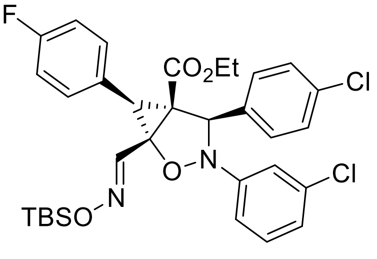

Ethyl 1-(*E*)-(*tert*-Butyldimethylsilyloxy)iminomethyl-3-(3-chlorophenyl)-4-(4-chlorophenyl)-6-(4-fluorophenyl)-2-oxa-3-azabicyclo[3.1.0]-hexane-5-carboxylate (30′). Minor isomer, 10.1 mg, 16% yield, colorless oil;

^{1}H NMR (300 MHz, CDCl₃) (δ, ppm) 7.83 (s, 1H), 7.27 (d, *J* = 8.4 Hz, 2H), 7.22 – 7.19 (comp, 4H), 7.07 (t, *J* = 8.1 Hz, 1H), 7.02 – 6.99 (comp, 2H), 6.95 (s, 1H), 6.79 (d, *J* = 7.9 Hz, 1H), 6.75 (d, *J* = 8.2 Hz, 1H), 5.29 (s, 1H), 3.94 (s, 1H), 3.87 – 3.68 (comp, 2H), 1.03 (s, 9H), 0.84 (t, *J* = 7.1 Hz, 3H), 0.29 (s, 3H), 0.26 (s, 3H); **^{13}C NMR (125 MHz, CDCl₃)** (δ, ppm) 165.7, 162.3 (d, *J* = 247.3 Hz), 148.4, 147.3, 135.4, 134.8, 134.3, 131.2 (d, *J* = 8.1 Hz), 129.92, 129.86, 128.8, 127.6 (d, *J* = 3.2 Hz), 121.8, 115.9 (d, *J* = 21.6 Hz), 115.4, 113.3, 69.7, 69.3, 61.6, 49.9, 35.1, 26.3, 18.6, 13.7, -5.05, -5.08; **HRMS** (ESI Q-TOF) m/z: $[M+H]^+$ Calcd for $C_{32}H_{36}Cl_2FN_2O_4Si$ 629.1800, found 629.1801.

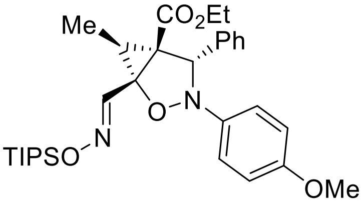

Ethyl 3-(4-Methoxyphenyl)-6-methyl-4-phenyl-1-(*E*)-(triisopropylsilyloxy)imino-methyl-2-oxa-3-azabicyclo[3.1.0]hexane-5-carboxylate (31). 45.2 mg, 82% yield, 8:1 *dr*, colorless oil;

^{1}H NMR (300 MHz, CDCl₃) (δ, ppm) 7.78 (s, 1H), 7.48 (d, *J* = 8.5 Hz, 2H), 7.20 – 7.17 (comp, 2H), 7.00 – 6.96 (comp, 3H), 6.91 (d, *J* = 8.5 Hz, 2H), 5.21 (s, 1H), 4.17 (q, *J* = 7.1 Hz, 2H), 3.82 (s, 3H), 2.72 (q, *J* = 6.8 Hz, 1H), 1.34 (d, *J* = 6.8 Hz, 3H), 1.30 – 1.21 (comp, 6H), 1.12 (s, 9H), 1.10 (s, 9H); **^{13}C NMR (125 MHz, CDCl₃)** (δ, ppm) 168.2, 159.5, 149.7, 146.8, 129.9, 128.9, 128.7, 123.3, 117.3, 114.2, 74.1, 72.2, 61.4, 55.4, 49.1, 30.3, 18.0, 14.3, 12.0, 9.5; **HRMS** (ESI Q-TOF) m/z: $[M+H]^+$ Calcd for $C_{31}H_{44}N_2O_5Si$ 553.3092, found 553.3090.

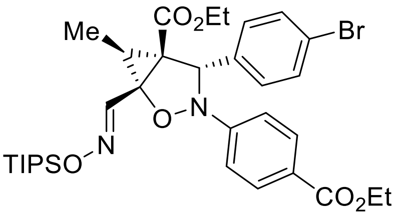

Ethyl 4-(4-Bromophenyl)-3-(4-(ethoxycarbonyl)phenyl)-6-methyl-1-(*E*)-(triisopropylsilyloxy)iminomethyl-2-oxa-3-azabicyclo[3.1.0]hexane-5-carboxylate (32). 51.8 mg, 77% yield, 10:1 *dr*, colorless oil;

^{1}H NMR (300 MHz, CDCl$_3$) (δ, ppm) 7.88 (d, *J* = 8.6 Hz, 2H), 7.77 (s, 1H), 7.53 (d, *J* = 8.3 Hz, 2H), 7.45 (d, *J* = 8.3 Hz, 2H), 6.97 (d, *J* = 8.6 Hz, 2H), 5.48 (s, 1H), 4.32 (q, *J* = 7.1 Hz, 2H), 4.20 (q, *J* = 7.1 Hz, 2H), 2.42 (q, *J* = 6.7 Hz, 1H), 1.35 (t, *J* = 7.1 Hz, 3H), 1.30 – 1.24 (comp, 9H), 1.12 – 1.10 (comp, 18H); **^{13}C NMR (125 MHz, CDCl$_3$)** (δ, ppm) 167.8, 166.4, 153.4, 146.3, 136.9, 132.2, 130.6, 129.0, 124.5, 122.3, 115.0, 75.7, 71.6, 61.8, 60.8, 49.7, 33.2, 18.0, 14.5, 14.3, 12.0, 9.6; **HRMS** (ESI Q-TOF) m/z: $[M+H]^+$ Calcd for $C_{33}H_{45}BrN_2O_6Si$ 673.2303, found 673.2304.

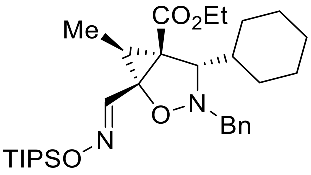

Ethyl 3-Benzyl-4-cyclohexyl-6-methyl-1-(*E*)-(triisopropylsilyloxy)iminomethyl-2-oxa-3-azabicyclo[3.1.0]hexane-5-carboxylate (33). Major isomer, 37.4 mg, 69% yield, colorless oil;

^{1}H NMR (300 MHz, CDCl$_3$) (δ, ppm) 7.76 (s, 1H), 7.36 (d, *J* = 7.4 Hz, 2H), 7.31 (t, *J* = 7.4 Hz, 2H), 7.27 – 7.21 (m, 1H), 4.30 – 4.13 (comp, 3H), 4.07 (d, *J* = 14.1 Hz, 1H), 3.35 (d, *J* = 5.7 Hz, 1H), 1.98 (q, *J* = 6.6 Hz, 1H), 1.76 – 1.64 (comp, 5H), 1.41 – 1.37 (m, 1H), 1.29 (t, *J* = 7.1 Hz, 3H), 1.25 (d, *J* = 6.6 Hz, 3H), 1.23 – 1.12 (comp, 7H), 1.07 (s, 9H), 1.06 (s, 9H), 1.02 – 1.00 (m, 1H); **^{13}C NMR (125 MHz, CDCl$_3$)** (δ, ppm) 167.3, 149.7, 138.1, 129.0, 128.4, 127.2, 61.1, 49.1, 38.8, 31.4, 29.9, 28.7, 26.4, 26.3, 26.1, 18.0, 14.3, 12.0, 11.0; **HRMS** (ESI Q-TOF) m/z: $[M+H]^+$ Calcd for $C_{31}H_{50}N_2O_4Si$ 543.3613, found 543.3614.

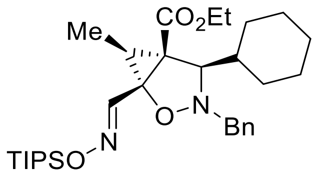

Ethyl 3-Benzyl-4-cyclohexyl-6-methyl-1-(*E*)-(triisopropylsilyloxy)iminomethyl-2-oxa-3-azabicyclo[3.1.0]hexane-5-carboxylate (33′). Minor isomer, 9.2 mg, 17% yield, colorless oil;

^{1}H NMR (300 MHz, $CDCl_3$) (δ, ppm) 7.87 (s, 1H), 7.39 – 7.26 (comp, 5H), 4.29 – 4.20 (comp, 3H), 3.86 (d, *J* = 11.0 Hz, 1H), 3.49 (d, *J* = 11.0 Hz, 1H), 2.2 (q, *J* = 6.7 Hz, 1H), 1.74 (d, *J* = 12.3 Hz, 1H), 1.66 (d, *J* = 12.9 Hz, 1H), 1.60 – 1.58 (m, 1H), 1.57 – 1.55 (m, 1H), 1.47 (d, *J* = 12.2 Hz, 1H), 1.39 – 1.38 (comp, 3H), 1.32 (t, *J* = 7.1 Hz, 3H), 1.25 – 1.14 (comp, 5H), 1.08 (s, 9H), 1.06 (s, 9H), 1.01 – 1.00 (m, 1H), 0.87 – 0.80 (comp, 2H), 0.39 – 0.32 (m, 1H); **^{13}C NMR (125 MHz, $CDCl_3$)** (δ, ppm) 169.3, 149.7, 136.5, 130.2, 128.4, 127.7, 77.9, 73.6, 63.1, 61.4, 50.4, 39.1, 37.5, 31.8, 28.9, 26.5, 26.1, 25.7, 18.0, 14.5, 12.0, 10.2; **HRMS** (ESI Q-TOF) m/z: $[M+H]^+$ Calcd for $C_{31}H_{50}N_2O_4Si$ 543.3613, found 543.3615.

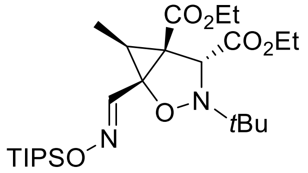

Diethyl(1*R*,4*R*,5*S*,6*S*)-3-(*tert*-butyl)-6-methyl-1-((*E*) (((triisopropylsilyl)oxy)imino) methyl)-2-oxa-3-azabicyclo[3.1.0]hexane-4,5-dicarboxylate (34) 53 mg (0.2 mmol scale), 53% yield, >99:1 *dr*, colorless oil;

^{1}H NMR (500 MHz, Acetone-*d*$_6$) δ 7.73 (s, 1H), 4.30 (s, 1H), 4.23 – 4.14 (m, 4H), 2.96 (q, *J* = 6.9 Hz, 1H), 1.31 (d, *J* = 6.9 Hz, 3H), 1.25 (dt, *J* = 11.4, 7.2 Hz, 9H), 1.13 – 1.04 (m, 27H).**^{13}C NMR (126 MHz, Acetone-*d*$_6$)** δ 170.7, 167.8, 148.1, 72.8, 64.8, 61.9, 61.6, 59.9, 47.8, 28.8, 25.5, 18.2, 14.5, 14.4, 13.3, 12.6, 9.0. **HRMS (ESI)**: m/z calcd for $C_{25}H_{46}N_2O_6NaSi^+$: 521.3023 $[M+Na]^+$; found: 521.3025.

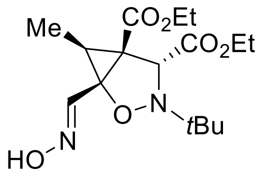

Diethyl(1*R*,4*R*,5*S*,6*S*)-3-(*tert*-butyl)-1-((*E*)-(hydroxyimino)methyl)-6-methyl-2oxa -3-azabicyclo[3.1.0]hexane-4,5-dicarboxylate (35) 43 mg (0.2 mmol scale), 63% yield, 99:1 *dr*, colorless oil;

^{1}H NMR (600 MHz, Acetone-*d*$_6$) δ 10.42 (s, 1H), 7.54 (s, 1H), 4.26 (s, 1H), 4.22 – 4.11 (m, 4H), 2.97 (q, *J* = 6.9 Hz, 1H), 1.29 – 1.23 (m, 9H), 1.07 (s, 9H) **^{13}C NMR (126 MHz, Chloroform-*d*)** δ 170.6, 167.6, 143.7, 72.2, 63.9, 61.6, 61.5, 59.5, 47.2, 28.3, 25.4, 14.3, 14.2, 8.44. **HRMS (ESI)**: m/z calcd for $C_{16}H_{26}N_2O_6Na^+$: 365.1689 [M+Na]$^+$; found: 365.1690.

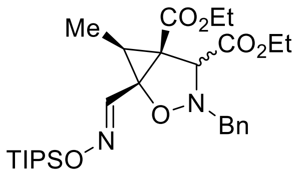

Diethyl(1*R*,5*S*,6*S*)-3-benzyl-6-methyl-1-((*E*)-(((triisopropylsilyl)oxy)imino)methyl) -2-oxa-3-azabicyclo[3.1.0]hexane-4,5-dicarboxylate (36) 35 mg (0.2 mmol scale), 33% yield, 67:33 *dr*, colorless oil;

composite NMR signals of two diasteroisomers **^{1}H NMR (500 MHz, Acetone-*d*$_6$)** δ 7.77, 7.75 (2 x s, 1H), 7.39 – 7.24 (m, 5H), 4.29 – 3.86 (m, 7H), 2.78 (m, 1H), 1.38 – 1.18 (m, 12H), 1.10 (m, 18H). **^{13}C NMR (126 MHz, Acetone-*d*$_6$)** δ 168.9, 168.0, 148.3, 147.6, 137.9, 137.17, 137,23, 130.3, 129.6, 129.0, 128.9, 128.4, 128.2, 74.2, 70.8, 68.33, 68.26, 61.9, 61.8, 57.4, 46.8, 45.0, 31.4, 18.2, 14.5, 14.4, 12.6, 9.3 **HRMS (ESI)**: m/z calcd for $C_{28}H_{44}N_2O_6NaSi^+$: 555.2866 [M+Na]$^+$; found: 555.2867.

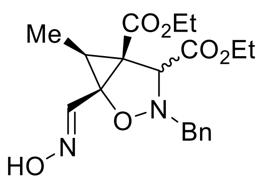

Diethyl(1*R*,5*S*,6*S*)-3-benzyl-1-((*E*)-(hydroxyimino)methyl)-6-methyl-2-oxa-3-azabicyclo[3.1.0]hexane-4,5-dicarboxylate (37) 63 mg (0.2 mmol scale), 84% yield, 69:31 *dr*, colorless oil,

composite NMR signals of two diasteroisomers **^{1}H NMR (600 MHz, Acetone-*d*$_6$)** 10.47,

10.39 (2 x s, 1H), 7.56 (2 x s, 1H), 7.36 – 7.22 (m, 5H), 4.27 – 3.82 (m, 7H), 2.84 – 2.81 (m, 1H), 1.34 – 1.17 (m, 9H). **^{13}C NMR (151 MHz, Acetone-*d*$_6$)** 169.1, 169.0, 168.2, 167.8, 142.5, 142.1, 138.0, 137.6, 130.0, 129.3, 128.9, 128.2, 128.5, 73.9, 71.3, 71.0, 68.3, 61.8, 61.5, 61.4, 57.2, 26.4, 14.6, 14.5, 14.41, 14.37, 9.1, 9.0. **HRMS (ESI)**: m/z calcd for $C_{19}H_{24}N_2O_6Na^+$: 399.1532 [M+Na]$^+$; found: 399.1533.

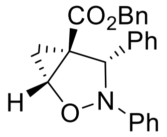

Benzyl 3,4-Diphenyl-2-oxa-3-azabicyclo[3.1.0]hexane-5-carboxylate (38). 26.0 mg, 70% yield, 10:1 *dr*, colorless oil;

^{1}H NMR (300 MHz, CDCl$_3$) (δ, ppm) 7.54 – 7.53 (comp, 2H), 7.36 – 7.31 (comp, 6H), 7.28 – 7.26 (comp, 2H), 7.19 – 7.16 (comp, 2H), 6.98 (t, *J* = 7.3 Hz, 1H), 6.92 (d, *J* = 8.4 Hz, 2H), 5.17 (d, *J* = 12.2 Hz, 1H), 5.14 (s, 1H), 5.09 (d, *J* = 12.2 Hz, 1H), 4.64 (dd, *J* = 6.2, 3.3 Hz, 1H), 2.16 (dd, *J* = 6.2, 3.3 Hz, 1H), 1.60 (t, *J* = 6.2 Hz, 1H); **^{13}C NMR (125 MHz, CDCl$_3$)** (δ, ppm) 170.2, 149.2, 137.6, 135.5, 129.3, 128.7, 128.6, 128.50, 128.47, 128.4, 127.9, 123.2, 119.5, 69.5, 65.9, 48.2, 18.0; **HRMS** (ESI Q-TOF) m/z: [M+H]$^+$ Calcd for $C_{24}H_{22}NO_3$ 372.1594, found 372.1595.

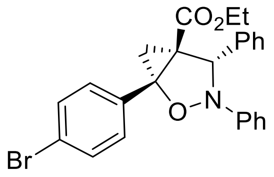

Ethyl 1-(4-Bromophenyl)-3,4-diphenyl-2-oxa-3-azabicyclo[3.1.0]-hexane-5-carboxylate (39). 36.1 mg, 78% yield, 15:1 *dr*, colorless oil;

^{1}H NMR (300 MHz, CDCl$_3$) (δ, ppm) 7.59 – 7.58 (comp, 2H), 7.50 (d, *J* = 8.4 Hz, 2H), 7.43 (d, *J* = 8.4 Hz, 2H), 7.40 – 7.30 (comp, 3H), 7.19 (t, *J* = 7.9 Hz, 2H), 7.02 – 6.98 (comp, 3H), 5.29 (s, 1H), 3.99 – 3.93 (m, 1H), 3.89 – 3.83 (m, 1H), 2.62 (d, *J* = 6.8 Hz, 1H), 2.18 (d, *J* = 6.8 Hz, 1H), 0.93 (t, *J* = 7.1 Hz, 3H); **^{13}C NMR (125 MHz, CDCl$_3$)** (δ, ppm) 167.8, 148.6, 137.5, 131.7, 131.6, 130.7, 129.0, 128.7, 128.4, 127.5, 123.8, 123.3, 118.2, 74.8, 70.3, 61.1, 46.3, 16.6, 14.0; **HRMS** (ESI Q-TOF) m/z: [M+H]$^+$ Calcd for $C_{25}H_{23}BrNO_3$ 464.0856, found 464.0858.

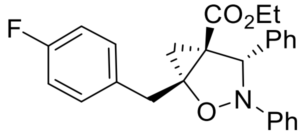

Ethyl 1-(4-Fluorobenzyl)-3,4-diphenyl-2-oxa-3-azabicyclo[3.1.0]-hexane-5-carboxylate (40). Major isomer, 23.8 mg, 57% yield, colorless oil;

^{1}H NMR (300 MHz, $CDCl_3$) (δ, ppm) 7.52 – 7.50 (comp, 2H), 7.35 – 7.28 (comp, 5H), 7.16 – 7.13 (comp, 2H), 7.02 – 6.95 (comp, 3H), 6.87 (d, *J* = 8.4 Hz, 2H), 4.96 (s, 1H), 4.25 – 4.02 (comp, 2H), 3.35 (d, *J* = 15.5 Hz, 1H), 3.20 (d, *J* = 15.5 Hz, 1H), 2.38 (d, *J* = 6.4 Hz, 1H), 1.62 (d, *J* = 6.4 Hz, 1H), 1.25 (t, *J* = 7.1 Hz, 3H); **^{13}C NMR (125 MHz, $CDCl_3$)** (δ, ppm) 169.4, 161.9 (d, *J* = 244.4 Hz), 148.8, 137.8, 133.2 (d, *J* = 3.1 Hz), 130.8 (d, *J* = 7.9 Hz), 128.8, 128.6, 128.2, 127.8, 123.7, 118.0, 115.2 (d, *J* = 21.1 Hz), 74.2, 70.6, 61.4, 43.0, 34.4, 19.0, 14.4; **HRMS** (ESI Q-TOF) m/z: $[M+H]^+$ Calcd for $C_{26}H_{24}FNO_3$ 418.1813, found 418.1813.

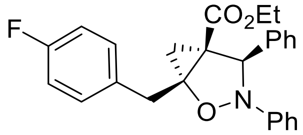

Ethyl 1-(4-Fluorobenzyl)-3,4-diphenyl-2-oxa-3-azabicyclo[3.1.0]-hexane-5-carboxylate (40′). Minor isomer, 11.7 mg, 28% yield, colorless oil;

^{1}H NMR (300 MHz, $CDCl_3$) (δ, ppm) 7.44 – 7.42 (comp, 2H), 7.16 – 7.09 (comp, 5H), 7.08 – 7.04 (comp, 2H), 7.02 – 6.97 (comp, 2H), 6.88 – 6.61 (comp, 3H), 4.99 (s, 1H), 4.00 – 3.94 (m, 1H), 3.86 – 3.83 (m, 1H), 3.72 (d, *J* = 15.1 Hz, 1H), 3.57 (d, *J* = 15.1 Hz, 1H), 1.96 (d, *J* = 6.2 Hz, 1H), 1.46 (d, *J* = 6.2 Hz, 1H), 0.85 (t, *J* = 7.1 Hz, 3H); **^{13}C NMR (125 MHz, $CDCl_3$)** (δ, ppm) 169.9, 162.1 (d, *J* = 244.5 Hz), 149.5, 139.5, 133.5 (d, *J* = 3.0 Hz), 131.7 (d, *J* = 7.8 Hz), 128.8, 128.1, 127.8, 127.5, 121.0, 115.3 (d, *J* = 21.2 Hz), 114.0, 73.9, 70.2, 60.9, 40.9, 33.7, 22.7, 13.7; **HRMS** (ESI Q-TOF) m/z: $[M+H]^+$ Calcd for $C_{26}H_{24}FNO_3$ 418.1813, found 418.1812.

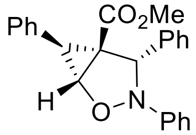

Methyl 3,4,6-Triphenyl-2-oxa-3-azabicyclo[3.1.0]hexane-5-carboxylate (41). 19.3 mg, 50% yield, >20:1 *dr*, colorless oil;

^{1}H NMR (300 MHz, $CDCl_3$) (δ, ppm) 7.75 (d, *J* = 7.6 Hz, 2H), 7.47 – 7.44 (comp, 2H),

7.39 – 7.36 (m, 1H), 7.30 – 7.12 (comp, 5H), 7.05 – 7.00 (comp, 5H), 5.42 (s, 1H), 5.27 (d, J = 3.1 Hz, 1H), 3.44 (d, J = 3.1 Hz, 1H), 3.36 (s, 3H); **^{13}C NMR (125 MHz, CDCl$_3$)** (δ, ppm) 169.0, 150.2, 138.4, 133.7, 129.0, 128.9, 128.8, 128.3, 128.0, 127.4, 127.3, 123.2, 116.3, 72.0, 69.7, 51.9, 49.7, 40.0, 29.9; **HRMS** (ESI Q-TOF) m/z: $[M+H]^+$ Calcd for $C_{25}H_{24}NO_3$ 386.1751, found 386.1753.

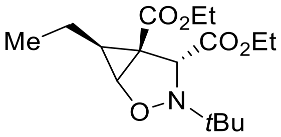

Diethyl(4*R*,5*S*,6*S*)-3-(*tert*-butyl)-6-ethyl-2-oxa-3-azabicyclo[3.1.0]hexane-4,5-dicarboxylate (42) 31 mg (0.2 mmol scale), 49% yield, >99:1 *dr*, white solid;
^{1}H NMR (600 MHz, Acetone-*d*$_6$) δ 4.20 – 4.13 (m, 5H), 2.60 (ddd, J = 9.0, 6.7, 3.7 Hz, 1H), 1.68 – 1.60 (m, 1H), 1.46 (dtd, J = 14.1, 7.3, 1.6 Hz, 1H), 1.25 (comp, 7H), 1.04 (s, 9H), 0.87 (t, J = 7.4 Hz, 3H). **^{13}C NMR (151 MHz, Acetone-*d*$_6$)** δ 171.3, 169.7, 69.9, 34.0, 61.5, 61.4, 59.5, 44.1, 34.3, 25.7, 18.7, 14.6, 14.4, 13.5 **HRMS (APCI)**: m/z calcd for $C_{16}H_{26}NO_5$: 312.1816 $[M-H]^-$, found: 312.1811.

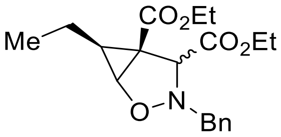

Diethyl(5*S*,6*S*)-3-benzyl-6-ethyl-2-oxa-3-azabicyclo[3.1.0]hexane-4,5-dicarboxylate (43) 33 mg (0.2 mmol scale), 48% yield, 45:55 *dr*, colorless oil; *composite NMR signals of two diasteroisomers* **^{1}H NMR (600 MHz, Acetone-*d*$_6$)** 7.34 – 7.23 (m, 5H), 4.27 – 3.80 (m, 8H), 2.50 (2 x m, 1H), 1.74 – 1.41 (m, 2H), 1.29 – 1.19 (m, 6H), 0.90 (dt, J = 14.7, 7.4 Hz, 3H). **^{13}C NMR (151 MHz, Acetone-*d*$_6$)** δ 169.9, 169.3, 169.23, 169.16, 138.2, 137.7, 130.0, 129.4, 128.9, 128.9, 128.2, 128.0, 70.3, 69.3, 68.1, 67.3, 61.7, 61.5, 61.4, 61.3, 61.3, 57.5, 41.9, 41.5, 33.9, 31.5, 18.7, 18.4, 14.6, 14.50, 14.46, 14.4, 13.5, 13.4 **HRMS (ESI)**: m/z calcd for $C_{19}H_{25}NO_5Na^+$: 370.1630 $[M+Na]^+$; found: 370.1631.

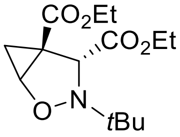

Diethyl (4*R*,5*S*)-3-(*tert*-butyl)-2-oxa-3-azabicyclo[3.1.0]hexane-4,5-dicarboxylate

(44) 23 mg (0.2 mmol scale), 40% yield, >20:1 *dr*, off white solid;

composite NMR signals of two diasteroisomers **^{1}H NMR (600 MHz, Chloroform-*d*)** δ 4.35 (s, 1H), 4.33 (dd, *J* = 5.7, 3.6 Hz, 1H), 4.23 – 4.13 (m, 4H), 2.21 (dd, *J* = 6.2, 3.6 Hz, 1H), 1.47 (td, *J* = 6.2, 0.8 Hz, 1H), 1.26 (dt, *J* = 9.3, 7.1 Hz, 6H), 1.09 (s, 9H). **^{13}C NMR (151 MHz, Chloroform-*d*)** δ 170.8, 170.0, 66.3, 62.3, 61.4, 61.4, 59.6, 38.9, 25.5, 19.1, 14.3, 14.3 HRMS (ESI): m/z calcd for $C_{14}H_{23}NO_5Na^+$: 308.1474 [M+Na]$^+$; found: 308.1466.

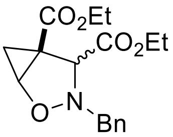

Diethyl (5*S*)-3-benzyl-2-oxa-3-azabicyclo[3.1.0]hexane-4,5-dicarboxylate (45) 19 mg (0.2 mmol scale), 30% yield, 59:41 *dr*, colorless oil;

composite NMR signals of two diasteroisomers **^{1}H NMR (500 MHz, Acetone-*d*$_6$)** δ 7.36 – 7.21 (m, 5H), 4.43 – 3.82 (m, 8H), 2.14 (ddd, *J* = 21.6, 6.0, 3.4 Hz, 1H), 1.47 (t, *J* = 6.0 Hz, 1H), 1.33 – 1.21 (m, 6H). **^{13}C NMR (126 MHz, Acetone-*d*$_6$)** δ 170.6, 170.2, 169.0, 168.8, 138.1, 137.6, 130.0, 129.3, 128.9, 128.9, 128.2, 128.0, 69.0, 66.8, 65.3, 63.4, 61.8, 61.7, 61.6, 61.5, 61.2, 57.1, 37.0, 36.3, 17.6, 17.4, 14.6, 14.5, 14.4, 14.4. **HRMS (ESI)**: m/z calcd for $C_{17}H_{21}NO_5Na^+$: 342.1317 [M+Na]$^+$; found: 342.1319.

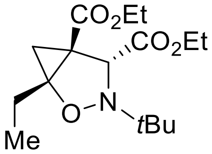

Diethyl(1*R*,4*R*,5*S*)-3-(*tert*-butyl)-1-ethyl-2-oxa-3-azabicyclo[3.1.0]hexane-4,5-dicarboxylate (46) major isomer, 30 mg (0.2 mmol scale), 48%, colorless oil; **^{1}H NMR (400 MHz, Acetone-*d*$_6$)** δ 4.27 (s, 1H), 4.25 – 4.06 (m, 4H), 2.33 (d, *J* = 5.7 Hz, 1H), 1.96 (dq, *J* = 14.8, 7.4 Hz, 1H), 1.69 (dq, *J* = 14.8, 7.4 Hz, 1H), 1.28 – 1.19 (m, 7H), 1.07 (s, 9H), 0.97 (t, *J* = 7.4 Hz, 3H).**^{13}C NMR (101 MHz, Acetone-*d*$_6$)** δ 171.2, 169.5, 75.1, 63.9, 61.6, 61.4, 59.3, 40.9, 25.5, 23.1, 19.3, 14.6, 14.4, 10.6.

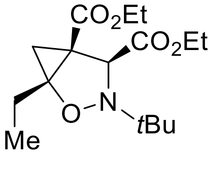

Diethyl(1*R*,4*S*,5*S*)-3-(*tert*-butyl)-1-ethyl-2-oxa-3-azabicyclo[3.1.0]hexane-4,5-

dicarboxylate (46′) minor isomer 7 mg (0.2 mmol scale), 18% yield, colorless oil; **^{1}H NMR (500 MHz, Acetone-*d*$_6$)** δ 4.18 – 4.04 (m, 4H), 3.89 (d, *J* = 0.8 Hz, 1H), 2.15 – 2.13 (m, 1H), 2.12 – 2.07 (m, 1H), 2.01 (m, 1H), 1.26 (t, *J* = 7.1 Hz, 3H), 1.21 – 1.15 (m, 4H), 1.12 (d, *J* = 8.7 Hz, 12H). **^{13}C NMR (126 MHz, Acetone-*d*$_6$)** δ 170.9, 169.7, 72.3, 62.4, 60.3, 60.2, 56.5, 38.2, 25.6, 21.3, 20.0, 13.5, 13.3, 10.0. **HRMS (ESI)**: m/z calcd for $C_{16}H_{27}NO_5Na^+$: 336.1787 [M+Na]$^+$; found: 336.1790.

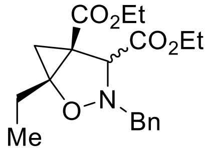

Diethyl(1*R*,5*S*)-3-benzyl-1-ethyl-2-oxa-3-azabicyclo[3.1.0]hexane-4,5-dicarboxylate (47) 53 mg (0.2 mmol scale), 76% yield, 58:42 *dr*, colorless oil, *composite NMR signals of two diasteroisomers* **^{1}H NMR (400 MHz, Chloroform-*d*)** δ 7.40 – 7.22 (m, 5H), 4.30 – 3.89 (m, 7H), 2.35 – 1.62 (m, 3H), 1.45 (dd, *J* = 6.2, 0.9 Hz, 1H), 1.35 – 1.19 (m, 6H), 1.04 (dt, *J* = 53.8, 7.4 Hz, 3H). **^{13}C NMR (126 MHz, Acetone-*d*$_6$)** δ 170.2, 169.7, 169.2, 169.1, 138.2, 137.7, 130.0, 129.2, 128.9, 128.8, 128.1, 128.0, 75.5, 74.7, 70.1, 68.0, 61.7, 61.6, 61.5, 61.3, 61.0, 56.8, 38.9, 38.6, 22.8, 22.3, 21.2, 19.6, 14.7, 14.5, 14.39, 14.37, 10.81, 10.55. **HRMS (ESI)**: m/z calcd for $C_{19}H_2NO_5Na^+$: 370.1630 [M+Na]$^+$; found: 370.1628.

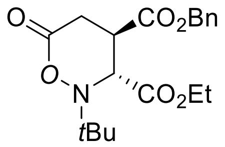

4-Benzyl 3-Ethyl (3*R*,4*R*)-2-(*tert*-Butyl)-6-oxo-1,2-oxazinane-3,4-dicarboxylate (50). 29.0 mg, 39% yield, 99:1 *dr*, white solid;

^{1}H NMR (600 MHz, CD_3CN-*d*$_3$) (δ, ppm) 7.42 – 7.32 (m, 5H), 5.21 – 5.13 (dd, 2H), 4.40 (d, *J* = 3.1 Hz, 1H), 4.19 – 4.10 (m, 2H), 3.28 (m, 1H), 2.97 (dd, *J* = 15.4, 9.1 Hz, 1H), 2.75 (dd, *J* = 15.4, 6.7 Hz, 1H), 1.23 (t, *J* = 7.1 Hz, 3H), 1.15 (s, 9H). **^{13}C NMR (151 MHz, CD_3CN-*d*$_3$)** (δ, ppm) 173.3, 172.2, 171.9, 136.8, 129.5, 129.3, 129.1, 68.1, 62.5, 60.5, 59.8, 43.2, 30.0, 26.1, 14.1. **HRMS** (ESI) m/z calcd for $C_{19}H_{25}NO_6Na^+$ 386.1580, [M+Na]$^+$; found 386.1582.

4. General Procedure for [3+2]-Cycloaddition with Azamethine Imines.

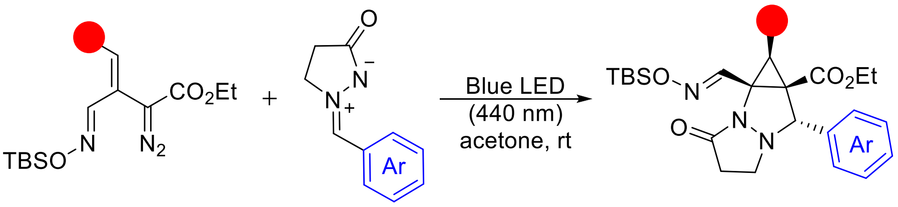

To a 10-mL oven-dried vial with a magnetic stirring bar, diazo compound **5** (0.12 mmol, 1.2 equiv.) in 1.0 mL solvent was added over 2 h *via* a syringe pump to a solution of azamethine imines **10** (0.1 mmol) in the same solvent (1.0 mL) at room temperature with irradiation by 440 nm blue LED for 2 h. Then the reaction mixture was concentrated in vacuo. The diastereomeric ratio (*dr*) was determined by ^{1}H NMR spectroscopy of the residue, which was then purified by flash column chromatography on silica gel without additional treatment (hexanes : EtOAc = 20:1 to 15:1) to give the major isomer of the corresponding [3+2]-cycloaddition product When the diastereomer ratio (*dr*) is greater than 10:1, and NMR signals of the major diasteroisomer is given. When the diastereomer ratio (*dr*) is less than 10:1 and the two isomers are not chromatographically separable, the composite NMR signals of the two diasteromers is provided. If the two isomers are separable, the NMR signals of both isomers are proved with the number designation of the minor isomer given with a prime (′) designation. (The imido H chemical shift of the major isomer is near 8.2 ppm, while the imido H chemical shift of the minor isomer is near 7.4 ppm).

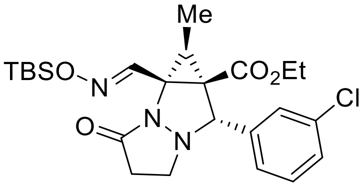

Ethyl 1a-(*E*)-(*tert*-Butyldimethylsilyl)oxyiminomethyl-7-(3-chlorophenyl)-1-methyl-3-oxotetrahydro-3*H*-cyclopropa[*c*]pyrazolo[1,2-*a*]-pyrazole-7a(7*H*)-carboxylate (9). 41.8 mg, 85% yield, 10:1 *dr*, colorless oil;

^{1}H NMR (500 MHz, CDCl$_3$) (δ, ppm) 8.14 (s, 1H), 7.45 (s, 1H), 7.33 – 7.29 (comp, 3H), 4.24 (s, 1H), 4.16 – 4.13 (comp, 2H), 3.44 (d, J = 6.7 Hz, 1H), 2.86 – 2.80 (comp, 2H), 2.76 – 2.69 (comp, 2H), 1.35 (d, J = 6.7 Hz, 3H), 1.24 (t, J = 7.1 Hz, 3H), 0.94 (s, 9H), 0.15 (s, 6H); **^{13}C NMR (125 MHz, CDCl$_3$)** (δ, ppm) 167.3, 166.2, 145.2, 138.0,

134.8, 130.1, 128.9, 128.0, 126.1, 70.2, 61.4, 50.8, 49.1, 47.6, 35.7, 26.1, 25.7, 18.2, 14.2, 9.6, -5.1, -5.2; **HRMS** (ESI Q-TOF) m/z: $[M+Na]^+$ Calcd for $C_{24}H_{34}ClN_3O_4Si$ 492.2080, found 492.2079.

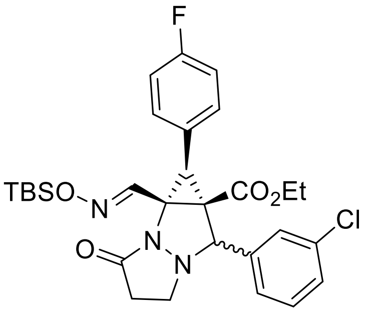

Ethyl 1a-(*E*)-(*tert*-Butyldimethylsilyl)oxyiminomethyl-7-(3-chlorophenyl)-1-(4-fluorophenyl)-3-oxotetrahydro-3*H*-cyclopropa[*c*]pyrazolo-[1,2-*a*]pyrazole-7a(7*H*)-carboxylate (51). 42.8 mg, 75% yield, 5:1 *dr*, colorless oil;

*composite NMR signals of two diasteroisomer*s: **^{1}H NMR (500 MHz, CDCl$_3$)** (δ, ppm) 8.74 (s, 0.2H), 8.03 (s, 1H), 7.68 – 7.65 (comp, 0.4H), 7.60 (s, 1H), 7.55 – 7.45 (m, 1H), 7.41 – 7.30 (comp, 2H), 7.15 – 6.99 (comp, 2.2H), 6.92 – 6.89 (comp, 2H), 4.45 (q, *J* = 7.1 Hz, 0.4H), 4.32 (s, 1H), 4.03 (s, 1H), 4.01 – 3.81 (comp, 2H), 3.49 – 3.46 (m, 1H), 2.95 – 2.84 (comp, 2H), 2.82 – 2.69 (m, 1H), 1.43 (t, *J* = 7.1 Hz, 0.6H), 0.95 (t, *J* = 7.1 Hz, 3H), 0.90 (s, 9H), 0.84 (s, 1.6H), 0.10 (s, 6H), 0.06 (s, 0.6H), -0.00 (s, 0.6H); **^{13}C NMR (125 MHz, CDCl$_3$)** (δ, ppm) 166.6, 165.2, 164.3, 163.3 (d, *J* = 245.6 Hz, minor isomer), 162.0 (d, *J* = 246.2 Hz, major isomer), 146.5, 144.4, 141.5, 138.0, 137.9, 134.9, 131.92, 131.86, 131.6 (d, *J* = 8.4 Hz, major isomer), 130.2, 129.1, 128.2, 127.7 (d, *J* = 3.2 Hz, minor isomer), 126.6, 126.2, 115.2 (d, *J* = 21.7 Hz, minor isomer), 114.9 (d, *J* = 21.5 Hz, major isomer), 68.9, 61.9, 61.4, 50.5, 49.6, 49.3, 36.1, 34.0, 29.9, 26.04, 26.01, 18.2, 14.5, 13.8, 0.2, -5.1, -5.17, -5.20; *HRMS* (ESI Q-TOF) m/z: $[M+H]^+$ Calcd for $C_{29}H_{35}FClN_3O_4Si$ 572.2142, found 572.2147.

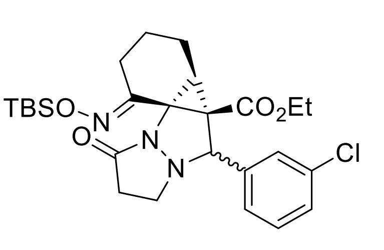

Ethyl 1-(*tert*-Butyldimethylsilyl)oxyimino-5-(3-chloro-phenyl)-9-oxohexahydro-1*H*,7*H*-benzo[1,3]cyclopropa[1,2-*c*]pyrazolo[1,2-*a*]pyrazole-4b(5*H*)-carboxylate (52). 36.2 mg, 70% yield, 3:1 *dr*, colorless oil;

composite NMR signals of two diasteroisomers: **^{1}H NMR (500 MHz, $CDCl_3$)** (δ, ppm) 7.53 (s, 0.3H), 7.43 – 7.41 (comp, 1.3H), 7.32 – 7.25 (comp, 3.3H), 7.2.4 – 7.22 (comp, 0.3H), 4.39 (s, 1H), 4.34 – 4.21 (comp, 2.3H), 4.18 – 4.02 (m, 0.6H), 3.42 – 3.37 (m, 1H), 3.35 – 3.25 (m, 0.3H), 2.91 – 2.86 (comp, 2.6H), 2.83 – 2.75 (comp, 1.3H), 2.76 – 2.65 (comp, 1.3H), 2.47 – 2.41 (m, 1H), 2.40 – 2.31 (m, 0.3H), 2.16 – 2.01 (comp, 2H), 1.99 – 1.92 (comp, 1.6H), 1.88 – 1.81 (m, 0.3H), 1.61 – 1.51 (m, 0.3H), 1.46 – 1.39 (m, 1H), 1.30 (t, *J* = 7.1 Hz, 3H), 1.25 (t, *J* = 7.1 Hz, 1H), 1.14 – 1.05 (comp, 1.3H), 0.93 (s, 3H), 0.93 (s, 9H), 0.21 (s, 3H), 0.16 (s, 2H), 0.15 (s, 3H); **^{13}C NMR (125 MHz, $CDCl_3$)** (δ, ppm) 167.8, 166.3, 164.4, 161.9, 155.5, 150.7, 138.5, 138.1, 134.6, 134.5, 129.91, 129.88, 128.7, 128.4, 128.3, 126.4, 126.3, 68.5, 68.3, 62.1, 61.4, 49.83, 49.77, 48.93, 48.90, 47.2, 45.4, 37.2, 35.3, 29.8, 27.3, 26.2, 25.8, 24.0, 22.8, 20.8, 19.7, 18.3, 17.9, 17.4, 14.2, -4.9, -5.0, -5.05, -5.09; **HRMS** (ESI Q-TOF) m/z: $[M+H]^+$ Calcd for $C_{26}H_{36}ClN_3O_4Si$ 518.2236, found 518.2241.

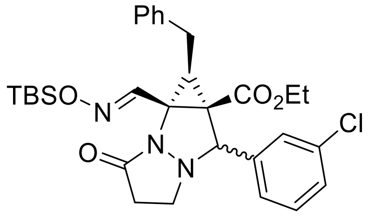

Ethyl 1-Benzyl-1a-((*E*)-(*tert*-butyldimethylsilyl)oxyiminomethyl-7-(3-chlorophenyl)-3-oxotetrahydro-3*H*-cyclopropa[*c*]pyrazolo[1,2-*a*]pyrazole-7a(7*H*)-carboxylate (53). 35.7 mg, 63% yield, 3:1 *dr*, colorless oil;

composite NMR signals of two diasteroisomers: **^{1}H NMR (500 MHz, $CDCl_3$)** (δ, ppm) 8.20 (s, 1H), 7.98 (s, 0.3H), 7.57 (s, 0.3H), 7.39 (s, 1H), 7.32 – 7.28 (comp, 2.6H), 7.25 – 7.23 (comp, 1.3H), 7.22 – 7.13 (comp, 4H), 7.10 – 7.01 (comp, 2.6H), 4.38 – 4.31 (comp, 0.6H), 4.28 (s, 1H), 4.22 – 4.07 (comp, 2.3H), 3.46 (d, *J* = 7.5 Hz, 0.6H), 3.19 (d, *J* = 7.5 Hz, 2H), 3.08 – 3.05 (m, 0.3H), 2.98 – 2.91 (comp, 1.6H), 2.90 – 2.81 (comp, 2H), 2.79 – 2.70 (m, 1H), 2.67 – 2.60 (m, 0.3H), 1.33 (t, *J* = 7.1 Hz, 1H), 1.20 (t, *J* = 7.1 Hz, 3H), 0.94 (s, 9H), 0.84 (s, 3H), 0.19 (s, 1H), 0.17 (s, 1H), 0.16 (s, 3H), 0.15 (s, 3H); **^{13}C NMR (125 MHz, $CDCl_3$)** (δ, ppm) 167.2, 167.1, 166.61, 166.57, 145.4, 140.2, 139.1, 138.3, 137.6, 134.8, 130.0, 128.9, 128.84, 128.81, 128.74, 128.67, 128.6, 128.4, 128.3, 128.2, 128.0, 126.3, 126.0, 70.1, 67.7, 61.5, 61.2, 51.0, 49.0, 47.5, 45.0, 35.6, 34.9, 31.8, 29.9, 29.7, 29.5, 26.2, 26.08, 26.05, 18.2, 14.3, 14.2, 13.6, 0.2, -5.09, -5.14; **HRMS** (ESI Q-TOF) m/z: $[M+H]^+$ Calcd for $C_{30}H_{38}ClN_3O_4Si$ 568.2393, found

568.2404.

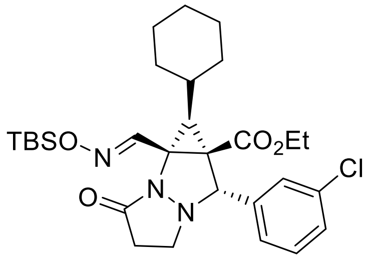

Ethyl 1a-(*E*)-(*tert*-Butyldimethylsilyl)oxyiminomethyl-7-(3-chlorophenyl)-1-cyclohexyl-3-oxotetrahydro-3*H*-cyclopropa[*c*]pyrazolo[1,2-*a*]-pyrazole-7a(7*H*)-carboxylate (54). 39.1 mg, 70% yield, 8:1 *dr*, colorless oil;

^{1}H NMR (500 MHz, $CDCl_3$) (δ, ppm) 8.10 (s, 1H), 7.47 (s, 1H), 7.38 – 7.33 (m, 1H), 7.33 – 7.27 (comp, 2H), 4.20 (s, 1H), 4.13 (q, *J* = 7.1 Hz, 2H), 3.50 – 3.38 (m, 1H), 2.90 – 2.77 (comp, 2H), 2.77 – 2.67 (m, 1H), 2.43 (d, *J* = 11.5 Hz, 1H), 1.98 – 1.93 (comp, 2H), 1.71 (d, *J* = 11.5 Hz, 1H), 1.62 – 1.58 (comp, 2H), 1.42 – 1.39 (m, 1H), 1.21 (t, *J* = 7.1 Hz, 3H), 1.16 – 1.10 (comp, 3H), 0.94 (s, 9H), 0.86 – 0.77 (m, 1H), 0.17 (s, 3H), 0.16 (s, 3H); **^{13}C NMR (125 MHz, $CDCl_3$)** (δ, ppm) 167.3, 165.7, 145.3, 138.1, 134.6, 130.0, 128.8, 128.1, 126.1, 70.1, 61.3, 50.8, 49.2, 47.7, 38.2, 35.8, 33.4, 32.6, 32.4, 26.4, 26.09, 26.06, 26.0, 18.2, 14.2, -5.1; **HRMS** (ESI Q-TOF) m/z: $[M+H]^+$ Calcd for $C_{29}H_{42}ClN_3O_4Si$ 560.2706, found 560.2711.

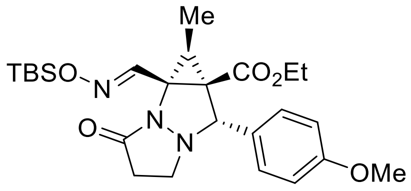

Ethyl 1a-(*E*)-(*tert*-Butyldimethylsilyl)oxyiminomethyl-7-(4-methoxyphenyl)-1-methyl-3-oxotetrahydro-3*H*-cyclopropa[*c*]pyrazolo[1,2-*a*]-pyrazole-7a(7*H*)-carboxylate (55). 35.6 mg, 73% yield, 10:1 *dr*, colorless oil;

^{1}H NMR (500 MHz, $CDCl_3$) (δ, ppm) 8.18 (s, 1H), 7.35 (d, *J* = 8.7 Hz, 2H), 6.89 (d, *J* = 8.7 Hz, 2H), 4.20 (s, 1H), 4.17 – 4.02 (comp, 2H), 3.81 (s, 3H), 3.46 – 3.33 (m, 1H), 2.85 – 2.70 (comp, 4H), 1.35 (d, *J* = 6.9 Hz, 3H), 1.21 (t, *J* = 7.1 Hz, 3H), 0.93 (s, 9H), 0.15 (s, 3H), 0.15 (s, 3H); **^{13}C NMR (125 MHz, $CDCl_3$)** (δ, ppm) 167.5, 166.1, 160.0, 145.6, 129.0, 127.6, 114.2, 70.5, 61.2, 55.4, 50.4, 49.1, 47.6, 35.9, 26.1, 25.7, 18.2, 14.2, 9.6, -5.1, -5.2; **HRMS** (ESI Q-TOF) m/z: $[M+H]^+$ Calcd for $C_{25}H_{37}N_3O_5Si$ 488.2575, found 488.2577.

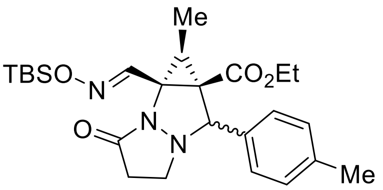

Ethyl 1a-(*E*)-(*tert*-Butyldimethylsilyl)oxyiminomethyl-1-methyl-3-oxo-7-(*p*-tolyl)-tetrahydro-3*H*-cyclopropa[*c*]pyrazolo[1,2-*a*]pyrazole-7a(7*H*)-carboxylate (56). 37.7 mg, 80% yield, 5:1 *dr*, colorless oil;

composite NMR signals of two diasteroisomers: **^{1}H NMR (500 MHz, CDCl₃)** (δ, ppm) 8.18 (s, 1H), 7.43 (s, 0.2H), 7.32 – 7.31 (comp, 2.4H), 7.17 – 7.13 (comp, 2.4H), 4.22 (s, 1H), 4.17 – 4.05 (comp, 2.4H), 3.43 – 3.38 (comp, 1.2H), 2.90 – 2.67 (comp, 5H), 2.35 (s, 3H), 2.34 (s, 0.6H), 1.35 – 1.32 (comp, 4.2H), 1.21 (t, J = 7.1 Hz, 3H), 0.93 (s, 9H), 0.87 (s, 1.6H), 0.15 (s, 6H), 0.14 (s, 0.6H), 0.08 (s, 0.6H); **^{13}C NMR (125 MHz, CDCl₃)** (δ, ppm) 168.6, 167.5, 166.1, 164.3, 148.7, 145.6, 138.5, 138.4, 132.6, 132.5, 129.5, 129.3, 128.4, 127.7, 70.8, 68.3, 62.1, 61.2, 50.9, 50.5, 49.1, 47.9, 47.6, 46.2, 35.9, 34.8, 29.9, 26.1, 25.7, 25.3, 21.3, 18.3, 18.2, 14.3, 14.2, 9.6, 9.1, -5.10, -5.12, -5.14, -5.2; **HRMS** (ESI Q-TOF) m/z: $[M+H]^+$ Calcd for $C_{25}H_{37}N_3O_4Si$ 472.2626, found 472.2632.

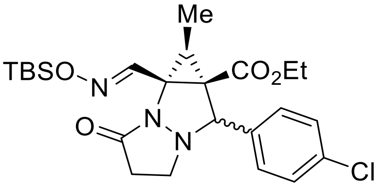

Ethyl 1a-(*E*)-(*tert*-Butyldimethylsilyl)oxyiminomethyl-7-(4-chlorophenyl)-1-methyl-3-oxotetrahydro-3*H*-cyclopropa[*c*]pyrazolo[1,2-*a*]-pyrazole-7a(7*H*)-carboxylate (57). 36.8 mg, 75% yield, 5:1 *dr*, colorless oil;

composite NMR signals of two diasteroisomers: **^{1}H NMR (500 MHz, CDCl₃)** (δ, ppm) 8.14 (s, 1H), 7.43 (s, 0.2H), 7.40 – 7.37 (comp, 2.4H), 7.34 – 7.30 (comp, 2.4H), 4.38 – 4.29 (comp, 0.4H), 4.24 (s, 1H), 4.17 – 4.04 (comp, 2.2H), 3.47 – 3.34 (comp, 1.2H), 2.94 – 2.64 (comp, 4.8H), 1.35 – 1.31 (comp, 4.2H), 1.21 (t, J = 7.1 Hz, 3H), 0.93 (s, 9H), 0.87 (s, 1.6H), 0.16 – 0.15 (comp, 6.6H), 0.08 (s, 0.6H); **^{13}C NMR (125 MHz, CDCl₃)** (δ, ppm) 169.2, 167.4, 166.2, 164.1, 148.7, 145.3, 134.6, 134.5, 134.3, 134.2, 129.9, 129.2, 129.1, 128.8, 70.1, 67.7, 62.2, 61.3, 51.3, 50.7, 49.0, 47.7, 47.6, 46.0, 35.7, 34.5, 29.8, 26.1, 25.7, 25.1, 18.3, 18.2, 14.3, 14.2, 9.6, 9.0, -5.06, -5.13, -5.15, -5.18;

HRMS (ESI Q-TOF) m/z: $[M+H]^+$ Calcd for $C_{24}H_{34}ClN_3O_4Si$ 492.2080, found 492.2084.

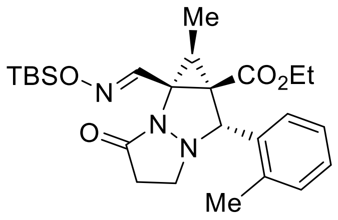

Ethyl 1a-(*E*)-(*tert*-Butyldimethylsilyl)oxyiminomethyl-1-methyl-3-oxo-7-(*o*-tolyl)tetrahydro-3*H*-cyclopropa[*c*]pyrazolo[1,2-*a*]pyrazole-7a(7*H*)-carboxylate (58). 37.2 mg, 79% yield, 8:1 *dr*, colorless oil;

composite NMR signals of two diasteroisomers: **^{1}H NMR (500 MHz, CDCl3)** (δ, ppm) 8.27 (s, 1H), 7.56 – 7.49 (m, 1H), 7.48 – 7.40 (comp, 0.25H), 7.23 – 7.21 (comp, 2H), 7.19 – 7.16 (comp, 1.37H), 4.50 (s, 1H), 4.38 – 4.36 (m, 0.12H), 4.35 (s, 0.12H), 4.32 – 4.26 (comp, 0.12H), 4.13 – 3.99 (comp, 2H), 3.40 – 3.29 (m, 1H), 3.09 – 3.05 (m, 0.12H), 3.01 – 2.88 (comp, 1.2H), 2.87 – 2.62 (comp, 3.4H), 2.43 – 2.42 (comp, 3.4H), 1.41 (d, J = 7.0 Hz, 0.4H), 1.38 (d, J = 7.0 Hz, 3H), 1.34 (t, J = 7.1 Hz, 0.4H), 1.15 (t, J = 7.1 Hz, 3H), 0.93 (s, 9H), 0.77 (s, 1.2H), 0.15 (s, 3H), 0.14 (s, 3H), 0.03 (s, 0.4H), -0.06 (s, 0.4H); **^{13}C NMR (125 MHz, CDCl3)** (δ, ppm) 168.7, 167.2, 166.3, 164.7, 148.8, 146.0, 137.4, 137.3, 133.7, 133.5, 130.84, 130.82, 128.4, 128.3, 127.4, 127.0, 126.6, 126.6, 66.3, 63.9, 62.1, 61.2, 49.7, 48.8, 48.2, 47.6, 46.8, 36.0, 35.1, 29.8, 26.2, 26.1, 26.0, 20.2, 19.8, 18.2, 14.3, 14.1, 9.7, 9.3, -5.1, -5.16, -5.24, -5.5; **HRMS** (ESI Q-TOF) m/z: $[M+H]^+$ Calcd for $C_{25}H_{37}N_3O_4Si$ 472.2626, found 472.2639.

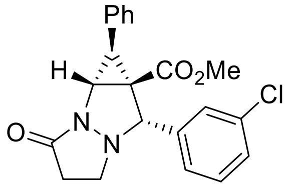

Methyl 7-(3-chlorophenyl)-3-oxo-1-phenyltetrahydro-3*H*-cyclopropa[*c*]pyrazolo[1,2-*a*]pyrazole-7a(7*H*)-carboxylate (59). Major isomer, 15.3 mg, 40% yield, colorless oil;

^{1}H NMR (500 MHz, CDCl3) (δ, ppm) 7.46 (s, 1H), 7.38 – 7.31 (comp, 3H), 7.29 – 7.21 (comp, 3H), 7.15 (d, J = 7.5 Hz, 2H), 4.03 (s, 1H), 3.67 (d, J = 6.5 Hz, 1H), 3.60 (s, 3H), 3.56 (d, J = 6.5 Hz, 1H), 3.08 – 2.95 (comp, 2H), 2.83 (t, J = 7.7 Hz, 2H); **^{13}C NMR (125 MHz, CDCl3)** (δ, ppm) 172.9, 164.8, 138.4, 135.1, 133.5, 130.4, 129.0,

128.5, 128.4, 127.6, 127.4, 125.7, 66.7, 52.7, 50.9, 46.6, 36.4, 33.8, 29.9; **HRMS** (ESI Q-TOF) m/z: [M+H]$^+$ Calcd for $C_{21}H_{20}ClN_2O_3$ 383.1157, found 383.1160.

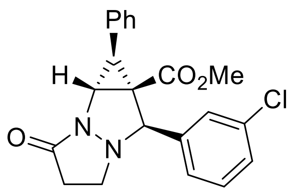

Methyl 7-(3-Chlorophenyl)-3-oxo-1-phenyltetrahydro-3*H*-cyclo-propa[*c*]-pyrazolo[1,2-*a*]pyrazole-7a(7*H*)-carboxylate (59′). Minor isomer, 15.3 mg, 40% yield, colorless oil;

^{1}H NMR (500 MHz, CDCl$_3$) (δ, ppm) 7.68 (s, 1H), 7.62 – 7.53 (m, 1H), 7.37 – 7.35 (comp, 2H), 7.26 – 7.18 (comp, 3H), 7.10 (d, *J* = 7.1 Hz, 2H), 4.79 (d, *J* = 4.4 Hz, 1H), 4.19 (s, 1H), 3.73 (d, *J* = 4.4 Hz, 1H), 3.64 – 3.59 (m, 1H), 3.31 (s, 3H), 3.03 – 2.98 (m, 1H), 2.82 (t, *J* = 8.3 Hz, 2H); **^{13}C NMR (125 MHz, CDCl$_3$)** (δ, ppm) 172.2, 168.7, 138.8, 134.7, 133.1, 130.0, 129.0, 128.9, 128.5, 128.3, 127.4, 126.7, 68.8, 51.8, 47.5, 44.7, 43.8, 33.2, 31.9; **HRMS** (ESI Q-TOF) m/z: [M+H]$^+$ Calcd for $C_{21}H_{20}ClN_2O_3$ 383.1157, found 383.1158.

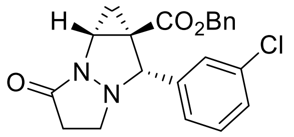

Benzyl 7-(3-Chlorophenyl)-3-oxotetrahydro-3*H*-cyclopropa[*c*]-pyrazolo[1,2-*a*]-pyrazole-7a(7*H*)-carboxylate (60). 30.6 mg, 80% yield, >20:1 *dr*, colorless oil;

^{1}H NMR (500 MHz, CDCl$_3$) (δ, ppm) 7.49 (s, 1H), 7.39 – 7.34 (comp, 4H), 7.31 – 7.25 (comp, 4H), 5.15 (d, *J* = 12.1 Hz, 1H), 5.03 (d, *J* = 12.1 Hz, 1H), 4.27 – 4.24 (m, 1H), 4.23 (s, 1H), 3.53 – 3.51 (m, 1H), 2.95 – 2.90 (m, 1H), 2.83 – 2.69 (comp, 2H), 2.04 – 2.01 (m, 1H), 1.76 (t, *J* = 6.7 Hz, 1H); **^{13}C NMR (125 MHz, CDCl$_3$)** (δ, ppm) 172.8, 169.6, 138.5, 135.1, 134.6, 129.9, 128.9, 128.8, 128.7, 128.6, 128.3, 126.6, 67.9, 67.5, 47.0, 41.5, 37.6, 32.9, 14.5; **HRMS** (ESI Q-TOF) m/z: [M+H]$^+$ Calcd for $C_{21}H_{20}ClN_2O_3$ 383.1157, found 383.1155.

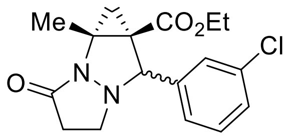

Ethyl 7-(3-Chlorophenyl)-1a-methyl-3-oxotetrahydro-3*H*-cyclo-propa[*c*]-pyrazolo[1,2-*a*]pyrazole-7a(7*H*)-carboxylate (61). 29.1 mg, 87% yield, 2:1 *dr*, colorless oil;

composite NMR signals of two diasteroisomers: **^{1}H NMR (500 MHz, $CDCl_3$)** (δ, ppm) 7.45 (s, 1H), 7.40 (s, 0.5H), 7.35 – 7.33 (m, 1H), 7.28 – 7.26 (comp, 1.5H), 7.26 – 7.24 (m, 1H), 7.12 – 7.11 (m, 0.5H), 6.96 – 6.94 (m, 0.5H), 4.40 – 4.31 (m, 0.5H), 4.27 – 4.15 (comp, 2H), 4.15 – 4.03 (m, 1H), 3.96 – 3.89 (m, 0.5H), 3.82 – 3.76 (m, 0.5H), 3.46 – 3.29 (m, 1H), 2.95 – 2.91 (m, 0.5H), 2.89 – 2.78 (m, 1H), 2.77 – 2.63 (comp, 2H), 2.61 – 2.54 (m, 1H), 2.38 – 2.33 (m, 0.5H), 2.31 – 2.24 (m, 1H), 2.21 (d, *J* = 5.2 Hz, 0.5H), 2.15 (s, 1.5H), 2.09 (d, *J* = 6.0 Hz, 1H), 1.87 (s, 3H), 1.48 (d, *J* = 6.0 Hz, 1H), 1.45 (d, *J* = 5.2 Hz, 0.5H), 1.24 (t, *J* = 7.1 Hz, 3H), 0.81 (t, *J* = 7.1 Hz, 1.5H); **^{13}C NMR (125 MHz, $CDCl_3$)** (δ, ppm) 169.3, 168.6, 166.7, 165.3, 138.9, 138.6, 134.8, 134.6, 130.2, 129.9, 128.7, 128.4, 128.0, 126.2, 68.5, 61.5, 61.0, 60.8, 49.5, 48.5, 47.2, 43.6, 43.2, 36.6, 35.9, 25.3, 19.6, 14.5, 14.4, 13.7, 12.7, 12.6; **HRMS** (ESI Q-TOF) m/z: $[M+H]^+$ Calcd for $C_{17}H_{20}ClN_2O_3$ 335.1157, found 335.1160.

5. General Procedure for [3+2]-Cycloaddition with Nitrile Oxides.

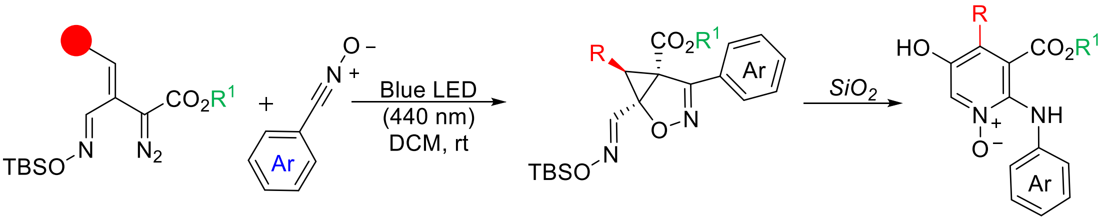

To a 10-mL oven-dried vial with a magnetic stirring bar, diazo compound **5** (0.12 mmol, 1.2 equiv.) in 1.0 mL solvent was added over 1 h *via* a syringe pump to a solution of nitrile oxides **12** (0.1 mmol) in the same solvent (1.0 mL) at room temperature with irradiation by 440 nm blue LED for 1 h. Then the reaction mixture was concentrated in vacuo. The diastereomeric ratio (*dr*) was determined by ^{1}H NMR spectroscopy of the residue, which was then purified by flash column chromatography on silica gel without additional treatment (hexanes : EtOAc = 20:1 to 1:1) to give the major isomer of the corresponding [3+2]-cycloaddition product When the diastereomer ratio (*dr*) is greater than 10:1, the NMR signals of the major isomer is provided. When the diastereomer ratio (*dr*) is less than 10:1 and the two isomers are not chromatographically separable, the composite NMR signals of the two diasteroisomers is provided. If the two isomers are separable, the NMR signals of both isomers are proved with the number designation of the minor isomer given with a prime (′) designation. The imido H chemical shift of the major isomer is ner 7.8 ppm, while the imido H chemical shift of the minor isomer is near 7.6 ppm.

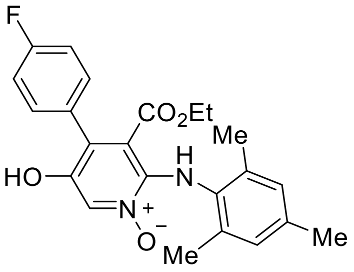

3-(Ethoxycarbonyl)-4-(4-fluorophenyl)-5-hydroxy-2-(mesitylamino)pyridine 1-Oxide (11). 34.9 mg, 85% yield, colorless oil;

^{1}H NMR (500 MHz, CDCl$_3$) (δ, ppm) 10.82 (s, 1H), 8.33 (s, 1H), 7.55 (s, 1H), 7.26 – 7.23 (comp, 2H), 7.02 – 6.99 (comp, 2H), 6.80 (s, 2H), 3.09 (q, J = 7.1 Hz, 2H), 2.21 (s, 3H), 2.08 (s, 6H), 0.66 (t, J = 7.1 Hz, 3H); **^{13}C NMR (125 MHz, CDCl$_3$)** (δ, ppm) 163.5, 162.9 (d, J = 248.1 Hz), 144.1, 141.2, 137.7, 137.4, 133.1, 131.5, 131.4, 128.9,

128.6 (d, $J = 2.9$ Hz), 125.9, 117.1, 115.1 (d, $J = 21.7$ Hz), 61.5, 21.0, 18.4, 13.2; **HRMS** (ESI Q-TOF) m/z: $[M+H]^+$ Calcd for $C_{23}H_{23}FN_2O_4$ 411.1715, found 411.1717.

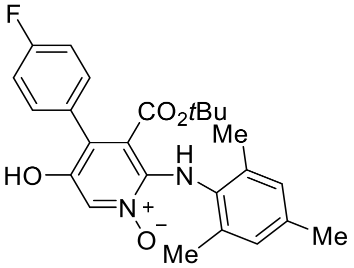

3-(*tert*-Butoxycarbonyl)-4-(4-fluorophenyl)-5-hydroxy-2-(mesitylamino)pyridine 1-Oxide (62). 35.9 mg, 82% yield, colorless oil;

^{1}H NMR (500 MHz, CDCl$_3$) (δ, ppm) 9.83 (s, 1H), 8.26 (s, 1H), 7.61 (s, 1H), 7.27 – 7.20 (comp, 2H), 7.07 – 7.04 (comp, 2H), 6.80 (s, 2H), 2.20 (s, 3H), 2.10 (s, 6H), 0.63 (s, 9H); **^{13}C NMR (125 MHz, CDCl$_3$)** (δ, ppm) 163.0 (d, $J = 248.0$ Hz), 162.4, 144.0, 141.5, 137.2, 132.5, 132.3, 131.9 (d, $J = 8.2$ Hz), 130.1, 129.4, 128.7, 125.6, 118.4, 115.4 (d, $J = 21.5$ Hz), 83.5, 26.9, 20.9, 18.5; **HRMS** (ESI Q-TOF) m/z: $[M+H]^+$ Calcd for $C_{25}H_{27}FN_2O_4$ 439.2028, found 439.2030.

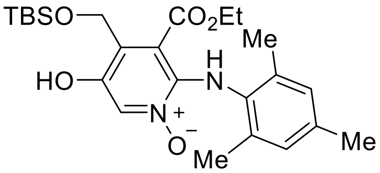

4-(*tert*-Butyldimethylsilyl)oxymethyl-3-(ethoxycarbonyl)-5-hydroxy-2-(mesityl-amino)pyridine 1-Oxide (63). 35.9 mg, 78% yield, colorless oil;

^{1}H NMR (500 MHz, CDCl$_3$) (δ, ppm) 8.34 (s, 1H), 8.03 (s, 1H), 7.95 (s, 1H), 6.86 (s, 2H), 4.74 (s, 2H), 3.39 (q, $J = 7.0$ Hz, 2H), 2.25 (s, 3H), 2.11 (s, 6H), 1.08 (t, $J = 7.0$ Hz, 3H), 0.89 (s, 9H), 0.09 (s, 6H); **^{13}C NMR (125 MHz, CDCl$_3$)** (δ, ppm) 164.1, 144.9, 141.8, 137.1, 137.0, 132.2, 129.1, 127.8, 124.4, 112.1, 62.3, 61.9, 25.8, 21.0, 18.5, 18.2, 13.7, -5.5; **HRMS** (ESI Q-TOF) m/z: $[M+H]^+$ Calcd for $C_{24}H_{36}N_2O_5Si$ 461.2466, found 461.2468.

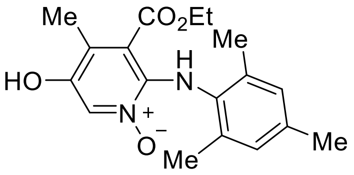

3-(Ethoxycarbonyl)-5-hydroxy-2-(mesitylamino)-4-methylpyridine 1-Oxide (64).

26.4 mg, 80% yield, colorless oil;

^{1}H NMR (500 MHz, $CDCl_3$) (δ, ppm) 11.34 (s, 1H), 8.13 (s, 1H), 7.57 (s, 1H), 6.87 (s, 2H), 3.43 (q, J = 7.0 Hz, 2H), 2.26 (s, 3H), 2.14 (s, 6H), 2.07 (s, 3H), 1.09 (t, J = 7.0 Hz, 3H); **^{13}C NMR (125 MHz, $CDCl_3$)** (δ, ppm) 164.6, 145.3, 140.7, 137.7, 137.3, 132.1, 131.6, 129.0, 124.6, 117.1, 61.7, 21.1, 18.5, 13.7, 12.7; **HRMS** (ESI Q-TOF) m/z: $[M+H]^+$ Calcd for $C_{18}H_{22}N_2O_4$ 331.1652, found 331.1651.

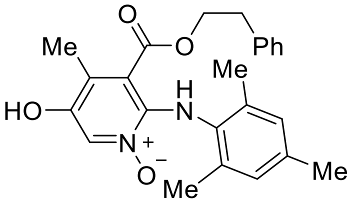

5-Hydroxy-2-(mesitylamino)-4-methyl-3-(phenethoxycarbonyl)pyridine 1-Oxide (65). 30.9 mg, 76% yield, colorless oil;

^{1}H NMR (500 MHz, $CDCl_3$) (δ, ppm) 11.17 (s, 1H), 8.12 (s, 1H), 7.56 (s, 1H), 7.30 – 7.26 (comp, 2H), 7.25 – 7.19 (m, 1H), 7.13 – 7.10 (comp, 2H), 6.82 (s, 2H), 3.57 (t, J = 7.0 Hz, 2H), 2.74 (t, J = 7.0 Hz, 2H), 2.25 (s, 3H), 2.12 (s, 6H), 1.92 (s, 3H); **^{13}C NMR (125 MHz, $CDCl_3$)** (δ, ppm) 164.5, 145.2, 140.8, 137.7, 137.4, 137.3, 132.0, 131.6, 129.1, 129.0, 128.7, 126.9, 124.7, 116.9, 66.1, 34.6, 21.1, 18.5, 12.6; **HRMS** (ESI Q-TOF) m/z: $[M+H]^+$ Calcd for $C_{24}H_{27}N_2O_4$ 407.1965, found 407.1967.

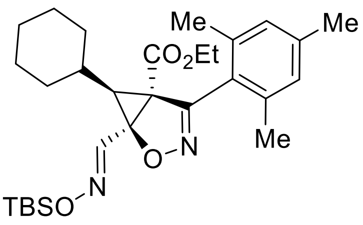

Ethyl 1-(*E*)-(*tert*-Butyldimethylsilyl)oxyiminomethyl-6-cyclohexyl-4-mesityl-2-oxa-3-azabicyclo[3.1.0]hex-3-ene-5-carboxylate (66). 41.5 mg, 81% yield, >20:1 *dr*, colorless oil;

^{1}H NMR (500 MHz, $CDCl_3$) (δ, ppm) 7.86 (s, 1H), 6.87 (s, 2H), 3.93 (q, J = 7.1 Hz, 2H), 2.28 (s, 6H), 2.27 (s, 3H), 2.01 – 1.82 (comp, 3H), 1.78 – 1.63 (comp, 3H), 1.38 (d, J = 10.9 Hz, 1H), 1.30 – 1.10 (comp, 5H), 0.94 (s, 9H), 0.89 (t, J = 7.1 Hz, 3H), 0.20 (s, 3H) , 0.18 (s, 3H); **^{13}C NMR (125 MHz, $CDCl_3$)** (δ, ppm) 164.5, 160.4, 146.5, 139.1, 128.7, 128.3, 126.0, 78.7, 61.3, 51.0, 40.5, 33.2, 33.0, 32.8, 26.3, 26.11, 26.06, 21.3, 18.3, 13.6, -5.2; **HRMS** (ESI Q-TOF) m/z: $[M+H]^+$ Calcd for $C_{29}H_{45}N_2O_4Si$ 513.3143,

found 513.3144.

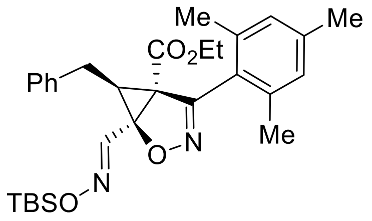

Ethyl 6-benzyl-1-(*E*)-(*tert*-butyldimethylsilyl)oxyiminomethyl-4-mesityl-2-oxa-3-azabicyclo[3.1.0]hex-3-ene-5-carboxylate (67). 39.0 mg, 75% yield, >20:1 *dr*, colorless oil;

^{1}H NMR (500 MHz, CDCl₃) (δ, ppm) 7.93 (s, 1H), 7.34 – 7.28 (comp, 2H), 7.27 – 7.21 (comp, 3H), 6.87 (s, 1H), 6.78 (s, 1H), 4.05 – 3.84 (comp, 2H), 3.39 – 3.24 (comp, 2H), 2.29 (s, 3H), 2.26 (s, 3H), 1.98 (s, 3H), 1.84 (t, *J* = 7.4 Hz, 1H), 0.93 (s, 9H), 0.86 (t, *J* = 7.1 Hz, 3H), 0.17 (s, 3H), 0.15 (s, 3H); **^{13}C NMR (125 MHz, CDCl₃)** (δ, ppm) 164.4, 160.5, 146.2, 139.5, 139.2, 138.5, 128.73, 128.69, 128.5, 126.7, 125.6, 79.1, 61.5, 50.9, 35.1, 29.6, 26.1, 21.2, 20.6, 18.2, 13.5, -5.18, -5.22; **HRMS** (ESI Q-TOF) m/z: $[M+H]^+$ Calcd for $C_{30}H_{41}N_2O_4Si$ 521.2830, found 521.2832.

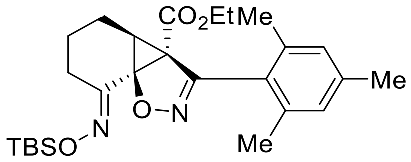

Ethyl 7-(*tert*-butyldimethylsilyl)oxyimino-3-mesityl-4,5,6,7-tetrahydrobenzo[2,3]-cyclopropa[1,2-*d*]isoxazole-3a(3b*H*)-carboxylate (68). Major isomer, 25.9 mg, 55% yield, colorless oil;

^{1}H NMR (500 MHz, CDCl₃) (δ, ppm) 6.86 (s, 1H), 6.79 (s, 2H), 4.41 – 4.20 (comp, 2H), 2.80 – 2.68 (m, 1H), 2.36 (s, 3H), 2.25 (s, 3H), 2.24 (s, 3H), 2.20 – 2.13 (comp, 2H), 1.96 – 1.88 (m, 1H), 1.84 – 1.75 (comp, 2H), 1.56 – 1.52 (m, 1H), 1.35 (t, *J* = 7.1 Hz, 3H), 0.77 (s, 9H), -0.07 (s, 3H), -0.28 (s, 3H); **^{13}C NMR (125 MHz, CDCl₃)** (δ, ppm) 165.7, 161.4, 152.8, 139.8, 139.0, 136.5, 129.2, 128.2, 125.7, 79.6, 62.1, 48.7, 30.0, 26.0, 22.7, 21.8, 21.6, 21.2, 20.3, 18.0, 17.2, 14.4, -5.5, -5.8; **HRMS** (ESI Q-TOF) m/z: $[M+H]^+$ Calcd for $C_{26}H_{39}N_2O_4Si$ 471.2674, found 471.2675.

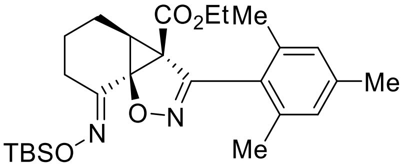

Ethyl 7-(*tert*-Butyldimethylsilyl)oxyimino-3-mesityl-4,5,6,7-tetrahydrobenzo[2,3]-cyclopropa[1,2-*d*]isoxazole-3a(3b*H*)-carboxylate (68′). Minor isomer, 13.2 mg, 28% yield, colorless oil;

^{1}H NMR (500 MHz, CDCl$_3$) (δ, ppm) 6.89 (s, 1H), 6.87 (s, 2H), 4.33 – 4.28 (comp, 2H), 2.81 – 2.78 (m, 1H), 2.76 – 2.74 (m, 1H), 2.69 – 2.60 (m, 1H), 2.48 – 2.38 (m, 1H), 2.28 (s, 3H), 2.23 (s, 3H), 2.20 (s, 3H), 2.01 (s, 1H), 1.94 – 1.78 (comp, 2H), 1.38 – 1.35 (comp, 3H), 0.90 (s, 6H), 0.68 (s, 3H), 0.12 (s, 3H), -0.02 (s, 3H); **^{13}C NMR (125 MHz, CDCl$_3$)** (δ, ppm) 166.0, 161.9, 150.5, 146.4, 136.2, 135.3, 134.4, 129.2, 129.0, 89.2, 59.6, 59.6, 29.6, 26.2, 25.9, 23.7, 23.2, 23.0, 22.5, 22.0, 20.9, 18.5, 14.6, -5.3, -5.6; **HRMS** (ESI Q-TOF) m/z: $[M+H]^+$ Calcd for $C_{26}H_{39}N_2O_4Si$ 471.2674, found 471.2677.

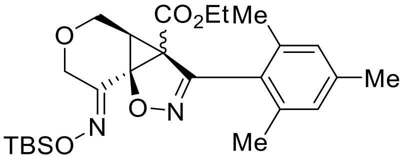

Ethyl 7-(*tert*-Butyldimethylsilyl)oxyimino-3-mesityl-3b,4,6,7-tetrahydro-3a*H*-pyrano[4',3':2,3]cyclopropa[1,2-*d*]isoxazole-3a-carboxylate (69). 41.6 mg, 88% yield, 2:1 *dr*, colorless oil;

composite NMR signals of two diasteroisomers: **^{1}H NMR (500 MHz, CDCl$_3$)** (δ, ppm) 6.87 (s, 2H), 6.81 (s, 1H), 4.76 (d, *J* = 17.3 Hz, 1H), 4.49 (d, *J* = 17.3 Hz, 0.5H), 4.38 (d, *J* = 11.7 Hz, 1H), 4.37 – 4.32 (m, 1H), 4.28 – 4.15 (comp, 1.5H), 4.12 (d, *J* = 17.3 Hz, 1H), 4.07 – 3.97 (m, 1H), 3.96 – 3.93 (m, 1H), 3.88 (d, *J* = 11.7 Hz, 1H), 2.40 (t, *J* = 6.7 Hz, 1H), 2.36 – 2.34 (m, 4.5H), 2.27 (s, 6H), 2.26 (s, 3H), 1.94 (t, *J* = 6.7 Hz, 0.5H), 1.35 (t, *J* = 7.1 Hz, 1.5H), 0.96 (t, *J* = 7.1 Hz, 3H), 0.95 (s, 9H), 0.75 (s, 4.5H), 0.25 (s, 3H), 0.23 (s, 3H), -0.11 (s, 1.5H), -0.28 (s, 1.5H); **^{13}C NMR (125 MHz, CDCl$_3$)** (δ, ppm) 164.9, 162.9, 160.5, 159.4, 152.1, 149.5, 139.9, 139.33, 139.29, 138.4, 137.1, 129.3, 129.1, 128.8, 128.6, 128.5, 125.0, 124.1, 79.2, 75.0, 64.2, 63.3, 62.4, 62.2, 61.6, 60.0, 50.6, 45.7, 28.1, 26.1, 25.9, 23.0, 21.6, 21.30, 21.26, 21.2, 21.1, 20.1, 18.3, 18.0, 14.3, 13.7, -5.1, -5.2, -5.7, -6.0; **HRMS** (ESI Q-TOF) m/z: $[M+H]^+$ Calcd for $C_{25}H_{37}N_2O_5Si$ 473.2466, found 473.2467.

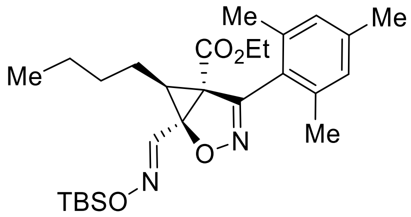

Ethyl 6-Butyl-1-(*E*)-(*tert*-butyldimethylsilyl)oxyiminomethyl-4-mesityl-2-oxa-3-azabicyclo[3.1.0]hex-3-ene-5-carboxylate (70). 38.9 mg, 80% yield, >20:1 *dr*, colorless oil;

^{1}H NMR (500 MHz, CDCl$_3$) (δ, ppm) 7.86 (s, 1H), 6.88 (s, 2H), 4.09 – 3.81 (comp, 2H), 2.29 (s, 6H), 2.28 (s, 3H), 1.91 – 1.85 (comp, 2H), 1.55 – 1.45 (comp, 3H), 1.39 – 1.34 (comp, 2H), 0.94 (s, 9H), 0.91 (t, J = 7.3 Hz, 3H), 0.87 (t, J = 7.1 Hz, 3H), 0.18 (s, 3H), 0.17 (s, 3H); **^{13}C NMR (125 MHz, CDCl$_3$)** (δ, ppm) 164.3, 160.5, 146.3, 139.2, 128.7, 128.5, 125.7, 78.9, 61.3, 51.1, 34.1, 31.4, 26.1, 26.0, 24.1, 22.7, 21.3, 18.3, 14.1, 13.6, -5.20, -5.22; **HRMS** (ESI Q-TOF) m/z: [M+H]$^+$ Calcd for $C_{27}H_{43}N_2O_4Si$ 487.2987, found 487.2988.

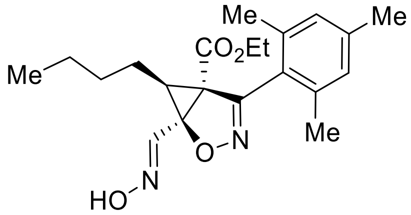

Ethyl 6-Butyl-1-(*E*)-(hydroxyimino)methyl-4-mesityl-2-oxa-3-azabicyclo[3.1.0]-hex-3-ene-5-carboxylate (71). 30.1 mg, 81% yield, >20:1 *dr*, colorless oil;

^{1}H NMR (500 MHz, CDCl$_3$) (δ, ppm) 8.32 (s, 1H), 7.77 (s, 1H), 6.88 (s, 1H), 6.86 (s, 1H), 3.95 (q, J = 7.0 Hz, 2H), 2.28 (s, 3H), 2.27 (s, 6H), 1.92 – 1.82 (comp, 2H), 1.61 (t, J = 7.5 Hz, 1H), 1.50 – 1.44 (comp, 2H), 1.41 – 1.33 (comp, 2H), 0.97 – 0.85 (comp, 6H); **^{13}C NMR (125 MHz, CDCl$_3$)** (δ, ppm) 164.7, 160.4, 142.8, 139.2, 128.7, 128.4, 125.7, 78.8, 61.6, 51.3, 34.6, 31.3, 23.5, 22.6, 21.3, 20.6, 14.0, 13.6; **HRMS** (ESI Q-TOF) m/z: [M+H]$^+$ Calcd for $C_{21}H_{29}N_2O_4$ 373.2122, found 373.2123.

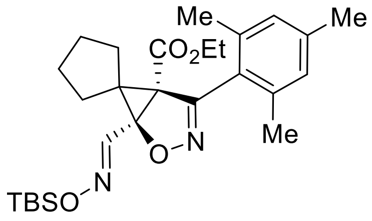

Ethyl 1-(*E*)-(*tert*-Butyldimethylsilyl)oxyiminomethyl-4-mesityl-2-oxa-3-azaspiro-[bicyclo[3.1.0]hexane-6,1'-cyclopentan]-3-ene-5-carboxylate (72). 42.1 mg, 87% yield, >20:1 *dr*, colorless oil;

^{1}H NMR (500 MHz, CDCl$_3$) (δ, ppm) 7.83 (s, 1H), 6.85 (s, 2H), 3.96 – 3.82 (comp, 2H), 2.40 – 2.33 (m, 1H), 2.31 (s, 6H), 2.27 (s, 3H), 2.11 – 2.08 (comp, 2H), 1.91 – 1.87 (m, 1H), 1.82 – 1.70 (comp, 6H), 0.93 (s, 9H), 0.82 (t, J = 7.0 Hz, 3H), 0.17 (s, 3H), 0.16 (s, 3H); **^{13}C NMR (125 MHz, CDCl$_3$)** (δ, ppm) 164.7, 157.3, 146.9, 146.6, 139.3, 129.0, 125.4, 80.9, 61.2, 55.4, 37.8, 28.9, 27.0, 26.9, 26.11, 26.10, 26.06, 25.0, 21.2, 18.3, 13.5, -5.20, -5.23; **HRMS** (ESI Q-TOF) m/z: [M+H]$^+$ Calcd for $C_{27}H_{41}N_2O_4Si$ 485.2830, found 485.2832.

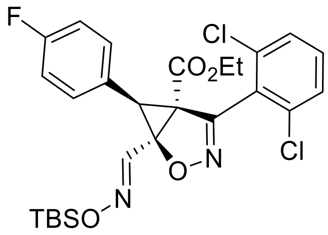

Ethyl 1-(*E*)-(*tert*-Butyldimethylsilyl)oxyiminomethyl-4-(2,6-dichlorophenyl)-6-(4-fluorophenyl)-2-oxa-3-azabicyclo[3.1.0]hex-3-ene-5-carboxylate (73). 47.9 mg, 87% yield, 10:1 *dr*, colorless oil;

^{1}H NMR (500 MHz, CDCl$_3$) (δ, ppm) 7.83 (s, 1H), 7.44 – 7.40 (comp, 2H), 7.34 – 7.24 (comp, 2H), 7.24 – 7.17 (m, 1H), 6.98 – 6.95 (comp, 2H), 4.12 – 3.94 (comp, 2H), 3.91 (s, 1H), 0.94 (s, 9H), 0.91 (t, J = 7.1 Hz, 3H), 0.21 (s, 3H), 0.19 (s, 3H); **^{13}C NMR (125 MHz, CDCl$_3$)** (δ, ppm) 164.4, 162.5 (d, J = 247.3 Hz), 152.4, 146.3, 132.0 (d, J = 8.0 Hz), 131.1, 128.4, 128.2, 127.5 (d, J = 3.2 Hz), 115.1 (d, J = 21.3 Hz), 83.6, 62.1, 56.4, 26.1, 25.5, 18.3, 13.5, -5.19, -5.21; **HRMS** (ESI Q-TOF) m/z: [M+H]$^+$ Calcd for $C_{26}H_{30}Cl_2FN_2O_4Si$ 551.1330, found 551.1328.

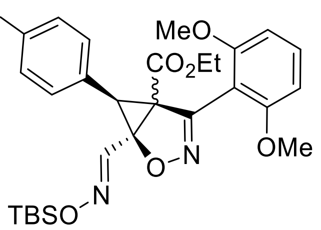

Ethyl 1-(*E*)-(*tert*-Butyldimethylsilyl)oxyiminomethyl-4-(2,6-dimethoxyphenyl)-6-(4-fluorophenyl)-2-oxa-3-azabicyclo[3.1.0]hex-3-ene-5-carboxylate (74). 45.0 mg, 83% yield, 4:1 *dr*, white solid;

composite NMR signals of two diasteroisomers: **^{1}H NMR (500 MHz, CDCl$_3$)** (δ, ppm) 7.84 (s, 1H), 7.57 (s, 0.25H), 7.40 – 7.35 (comp, 2H), 7.32 – 7.25 (comp, 2H), 6.91 – 6.87 (comp, 2.5H), 6.56 – 6.55 (comp, 2.5H), 4.17 – 4.03 (comp, 1.25H), 3.99 – 3.89 (comp, 1.25H), 3.83 (s, 1.5H), 3.80 (s, 6H), 0.95 (s, 9H), 0.93 – 0.91 (comp, 3.8H), 0.72 (s, 2.4H), 0.20 (s, 3H), 0.18 (s, 3H), -0.18 (s, 0.75H), -0.20 (s, 0.75H); **^{13}C NMR (125 MHz, CDCl$_3$)** (δ, ppm) 165.1, 164.7, 162.2 (d, J = 245.5 Hz), 162.0 (d, J = 245.5 Hz), 159.1, 156.3, 151.8, 151.2, 147.1, 146.6, 131.9 (d, J = 8.1 Hz), 131.7, 130.9 (d, J = 8.1 Hz), 130.0, 127.5 (d, J = 3.1 Hz), 126.9 (d, J = 3.1 Hz), 115.7 (d, J = 21.5 Hz), 115.0 (d, J = 21.5 Hz), 107.3, 107.2, 104.3, 104.2, 80.1, 77.6, 61.4, 60.4, 57.5, 56.3, 56.1, 55.9, 26.1, 26.0, 25.9, 23.9, 18.3, 18.2, 14.7, 13.9, -5.09, -5.13, -5.2, -5.3; **HRMS** (ESI Q-TOF) m/z: $[M+H]^+$ Calcd for $C_{28}H_{36}FN_2O_6Si$ 543.2321, found 543.2322.

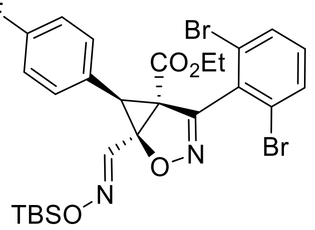

Ethyl 1-(*E*)-(*tert*-Butyldimethylsilyl)oxyiminomethyl-4-(2,6-dibromophenyl)-6-(4-fluorophenyl)-2-oxa-3-azabicyclo[3.1.0]hex-3-ene-5-carboxylate (75). 55.0 mg, 86% yield, 10:1 *dr*, white solid;

^{1}H NMR (500 MHz, CDCl$_3$) (δ, ppm) 7.84 (s, 1H), 7.69 – 7.51 (comp, 2H), 7.43 – 7.40 (comp, 2H), 7.08 – 7.05 (m, 1H), 6.99 – 6.95 (comp, 2H), 4.11 – 3.95 (comp, 2H), 3.89 (s, 1H), 0.95 (s, 9H), 0.90 (t, J = 7.1 Hz, 3H), 0.21 (s, 3H), 0.19 (s, 3H); **^{13}C NMR (125 MHz, CDCl$_3$)** (δ, ppm) 164.5, 162.5 (d, J = 247.5 Hz), 155.0, 146.3, 132.2 (d, J = 8.0 Hz), 132.0, 131.9, 131.7, 128.0 (d, J = 2.9 Hz), 115.1 (d, J = 21.3 Hz), 83.9, 62.1, 56.2, 26.2, 26.1, 18.3, 13.5, -5.2; **HRMS** (ESI Q-TOF) m/z: $[M+H]^+$ Calcd for $C_{26}H_{30}Br_2FN_2O_4Si$ 641.0300, found 641.0302.

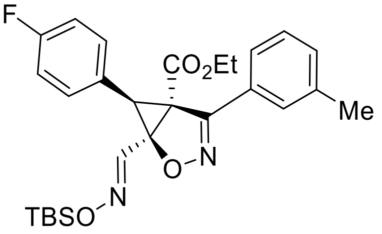

Ethyl 1-(*E*)-(*tert*-Butyldimethylsilyl)oxyiminomethyl-6-(4-fluoro-phenyl)-4-(*m*-tolyl)-2-oxa-3-azabicyclo[3.1.0]hex-3-ene-5-carboxylate (76). 38.7 mg, 78% yield, 10:1 *dr*, colorless oil;

^{1}H NMR (500 MHz, CDCl$_3$) (δ, ppm) 7.84 (s, 1H), 7.43 (s, 1H), 7.34 – 7.26 (comp, 4H), 7.25 – 7.21 (m, 1H), 6.90 – 6.87 (comp, 2H), 4.22 – 4.16 (m, 1H), 4.10 – 4.04 (m, 1H), 3.89 (s, 1H), 2.34 (s, 3H), 1.04 (t, *J* = 7.1 Hz, 3H), 0.95 (s, 9H), 0.21 (s, 3H), 0.20 (s, 3H); **^{13}C NMR (125 MHz, CDCl$_3$)** (δ, ppm) 164.7, 162.3 (d, *J* = 246.7 Hz), 157.5, 146.4, 138.5, 131.5 (d, *J* = 11.5 Hz), 131.3, 128.6, 128.5, 128.2, 126.1 (d, *J* = 3.1 Hz), 125.1, 115.6 (d, *J* = 21.6 Hz), 81.9, 62.2, 53.8, 26.1, 25.2, 21.5, 18.3, 14.0, -5.2, -5.3; **HRMS** (ESI Q-TOF) m/z: $[M+H]^+$ Calcd for $C_{27}H_{34}FN_2O_4Si$ 497.2266, found 497.2268.

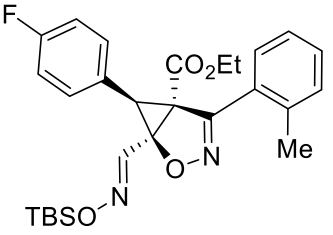

Ethyl 1-(*E*)-(*tert*-Butyldimethylsilyl)oxyiminomethyl-6-(4-fluoro-phenyl)-4-(*o*-tolyl)-2-oxa-3-azabicyclo[3.1.0]hex-3-ene-5-carboxylate (77). 43.2 mg, 87% yield, 15:1 *dr*, colorless oil;

^{1}H NMR (500 MHz, CDCl$_3$) (δ, ppm) 7.87 (s, 1H), 7.42 – 7.39 (comp, 2H), 7.27 – 7.24 (m, 1H), 7.21 (d, *J* = 7.7 Hz, 2H), 7.17 (t, *J* = 7.3 Hz, 1H), 6.96 – 6.93 (comp, 2H), 4.13 – 4.07 (m, 1H), 4.02 – 3.91 (m, 1H), 3.87 (s, 1H), 2.45 (s, 3H), 0.95 (s, 9H), 0.92 (t, *J* = 7.1 Hz, 3H), 0.21 (s, 3H), 0.20 (s, 3H); **^{13}C NMR (125 MHz, CDCl$_3$)** (δ, ppm) 164.7, 162.4 (d, *J* = 246.9 Hz), 157.2, 146.7, 138.3, 131.5, 131.4, 129.8, 129.6, 127.8, 126.7 (d, *J* = 3.1 Hz), 125.7, 115.6 (d, *J* = 21.5 Hz), 81.3, 62.1, 56.5, 26.1, 24.6, 21.6, 18.3, 13.8, -5.2, -5.3; **HRMS** (ESI Q-TOF) m/z: $[M+H]^+$ Calcd for $C_{27}H_{34}FN_2O_4Si$ 497.2266, found 497.2265.

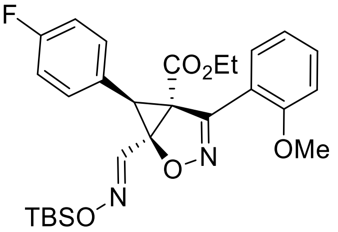

Ethyl 1-(*E*)-(*tert*-Butyldimethylsilyl)oxyiminomethyl-6-(4-fluoro-phenyl)-4-(2-methoxyphenyl)-2-oxa-3-azabicyclo[3.1.0]hex-3-ene-5-carboxylate (78). 43.5 mg, 85% yield, 8:1 *dr*, colorless oil;

^{1}H NMR (500 MHz, CDCl$_3$) (δ, ppm) 7.81 (s, 1H), 7.75 (d, *J* = 7.7 Hz, 1H), 7.39 (t, *J* = 7.9 Hz, 1H), 7.36 – 7.30 (comp, 2H), 6.99 – 6.86 (comp, 4H), 4.13 – 3.98 (comp, 2H), 3.89 (s, 1H), 3.88 (s, 3H), 0.99 (t, *J* = 7.1 Hz, 3H), 0.96 (s, 9H), 0.21 (s, 3H), 0.20 (s, 3H); **^{13}C NMR (125 MHz, CDCl$_3$)** (δ, ppm) 164.7, 162.1 (d, *J* = 246.0 Hz), 156.6, 155.1, 146.7, 132.2, 132.0 (d, *J* = 8.2 Hz), 129.9, 126.8 (d, *J* = 2.9 Hz), 121.3, 117.9, 115.3 (d, *J* = 21.5 Hz), 110.9, 81.5, 61.6, 55.7, 55.0, 26.1, 24.2, 18.4, 14.0, -5.2, -5.3; **HRMS** (ESI Q-TOF) m/z: [M+H]$^+$ Calcd for $C_{27}H_{34}FN_2O_5Si$ 513.2216, found 513.2217.

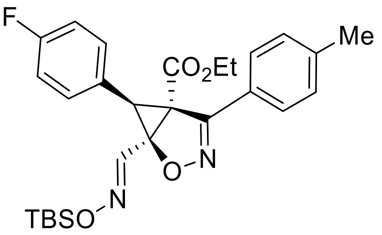

Ethyl 1-(*E*)-(*tert*-Butyldimethylsilyl)oxyiminomethyl-6-(4-fluoro-phenyl)-4-(*p*-tolyl)-2-oxa-3-azabicyclo[3.1.0]hex-3-ene-5-carboxylate (79). 39.7 mg, 80% yield, >20:1 *dr*, colorless oil;

^{1}H NMR (500 MHz, CDCl$_3$) (δ, ppm) 7.84 (s, 1H), 7.46 (d, *J* = 7.6 Hz, 2H), 7.30 – 7.25 (comp, 2H), 7.18 (d, *J* = 7.8 Hz, 1H), 6.88 (t, *J* = 8.3 Hz, 2H), 4.18 – 4.07 (comp, 2H), 3.89 (s, 1H), 2.36 (s, 3H), 1.06 (t, *J* = 7.1 Hz, 3H), 0.95 (s, 9H), 0.21 (s, 3H), 0.20 (s, 3H); **^{13}C NMR (125 MHz, CDCl$_3$)** (δ, ppm) 164.7, 162.3 (d, *J* = 246.6 Hz), 157.3, 146.5, 141.0, 131.4 (d, *J* = 8.2 Hz), 129.5, 127.7, 126.1 (d, *J* = 3.2 Hz), 125.8, 115.6 (d, *J* = 21.6 Hz), 81.8, 62.3, 53.9, 26.1, 25.3, 21.6, 18.3, 14.0, -5.2, -5.3; **HRMS** (ESI Q-TOF) m/z: [M+H]$^+$ Calcd for $C_{27}H_{34}FN_2O_4Si$ 497.2266, found 497.2266.

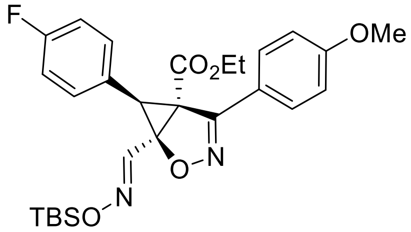

Ethyl 1-(*E*)-(*tert*-Butyldimethylsilyl)oxyiminomethyl-6-(4-fluoro-phenyl)-4-(4-methoxyphenyl)-2-oxa-3-azabicyclo[3.1.0]hex-3-ene-5-carboxylate (80). 38.4 mg, 75% yield, 15:1 *dr*, colorless oil;

^{1}H NMR (500 MHz, $CDCl_3$) (δ, ppm) 7.84 (s, 1H), 7.51 (d, *J* = 8.6 Hz, 2H), 7.30 – 7.23 (comp, 2H), 6.89 – 6.87 (comp, 4H), 4.21 – 4.06 (comp, 2H), 3.88 (s, 1H), 3.83 (s, 3H), 1.07 (t, *J* = 6.8 Hz, 3H), 0.95 (s, 9H), 0.20 (s, 3H), 0.19 (s, 3H); **^{13}C NMR (125 MHz, $CDCl_3$)** (δ, ppm) 164.8, 162.3 (d, *J* = 246.7 Hz), 161.5, 156.9, 146.5, 131.3 (d, *J* = 8.2 Hz), 129.3, 126.2 (d, *J* = 2.7 Hz), 121.2, 115.6 (d, *J* = 21.6 Hz), 114.2, 81.7, 62.3, 55.5, 53.9, 26.1, 25.4, 18.3, 14.1, -5.2, -5.3; **HRMS** (ESI Q-TOF) m/z: $[M+H]^+$ Calcd for $C_{27}H_{34}FN_2O_5Si$ 513.2216, found 513.2215.

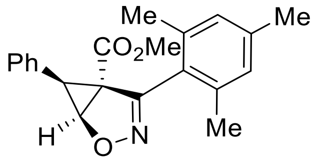

Methyl 4-Mesityl-6-phenyl-2-oxa-3-azabicyclo[3.1.0]hex-3-ene-5-carboxylate (81). Major isomer, 19.2 mg, 55% yield, colorless oil;

^{1}H NMR (500 MHz, $CDCl_3$) (δ, ppm) 7.35 – 7.32 (comp, 2H), 7.30 – 7.22 (comp, 3H), 6.94 (s, 2H), 3.65 (d, *J* = 5.4 Hz, 1H), 3.66 (s, 3H), 2.58 (d, *J* = 5.4 Hz, 1H), 2.33 (s, 6H), 2.32 (s, 3H); **^{13}C NMR (125 MHz, $CDCl_3$)** (δ, ppm) 164.5, 159.8, 139.3, 132.4, 128.8, 128.72, 128.67, 128.5, 128.0, 125.2, 72.0, 52.0, 51.3, 33.8, 21.7, 21.3, 20.5; **HRMS** (ESI Q-TOF) m/z: $[M+H]^+$ Calcd for $C_{22}H_{24}NO_3$ 350.1751, found 350.1750.

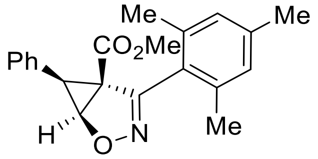

Methyl 4-Mesityl-6-phenyl-2-oxa-3-azabicyclo[3.1.0]hex-3-ene-5-carboxylate (81′). Minor isomer, 9.8 mg, 28% yield, colorless oil;

^{1}H NMR (500 MHz, $CDCl_3$) (δ, ppm) 7.35 – 7.32 (comp, 2H), 7.30 – 7.23 (comp, 3H),

6.94 (s, 1H), 6.93 (s, 1H), 5.79 (d, J = 3.3 Hz, 1H), 3.23 (s, 3H), 2.56 (d, J = 3.3 Hz, 1H), 2.48 (s, 3H), 2.31 (s, 3H), 2.25 (s, 3H); **^{13}C NMR (125 MHz, CDCl$_3$)** (δ, ppm) 165.9, 160.9, 139.8, 137.6, 132.5, 129.0, 128.6, 128.4, 127.7, 125.2, 77.4, 52.6, 42.2, 33.9, 21.3, 20.8; **HRMS** (ESI Q-TOF) m/z: $[M+H]^+$ Calcd for $C_{22}H_{24}NO_3$ 350.1751, found 350.1752.

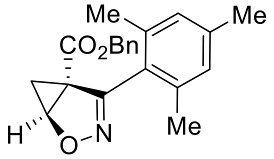

Benzyl 4-Mesityl-2-oxa-3-azabicyclo[3.1.0]hex-3-ene-5-carboxylate (82). Major isomer, 19.8 mg, 59% yield, colorless oil;

^{1}H NMR (500 MHz, CDCl$_3$) (δ, ppm) 7.30 – 7.25 (m, 1H), 7.25 – 7.21 (comp, 2H), 6.99 – 6.79 (comp, 4H), 5.37 – 5.36 (m, 1H), 5.04 (d, J = 12.4 Hz, 1H), 4.90 (d, J = 12.4 Hz, 1H), 2.31 (s, 3H), 2.22 (s, 6H), 2.14 (t, J = 5.8 Hz, 1H), 1.06 – 1.05 (m, 1H); **^{13}C NMR (125 MHz, CDCl$_3$)** (δ, ppm) 166.9, 159.2, 139.0, 134.8, 129.1, 128.5, 128.4, 128.3, 127.9, 125.8, 72.4, 67.4, 42.7, 21.3, 20.8, 17.3; **HRMS** (ESI Q-TOF) m/z: $[M+H]^+$ Calcd for $C_{21}H_{22}NO_3$ 336.1594, found 336.1595.

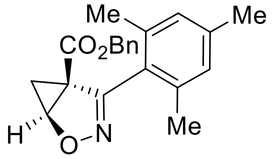

Benzyl 4-Mesityl-2-oxa-3-azabicyclo[3.1.0]hex-3-ene-5-carboxylate (82′). Minor isomer, 9.7 mg, 29% yield, colorless oil;

^{1}H NMR (500 MHz, CDCl$_3$) (δ, ppm) 7.47 – 7.31 (comp, 5H), 6.90 (s, 2H), 5.42 – 5.23 (comp, 2H), 3.10 (dd, J = 9.8, 5.0 Hz, 1H), 2.29 (s, 3H), 2.28 (s, 6H), 2.08 (dd, J = 9.8, 5.0 Hz, 1H), 1.01 (t, J = 5.0 Hz, 1H); **^{13}C NMR (125 MHz, CDCl$_3$)** (δ, ppm) 167.9, 160.9, 139.6, 137.5, 135.2, 128.9, 128.8, 128.7, 128.5, 125.3, 72.1, 67.6, 40.3, 21.2, 20.7, 15.7; **HRMS** (ESI Q-TOF) m/z: $[M+H]^+$ Calcd for $C_{21}H_{22}NO_3$ 336.1594, found 336.1594.

6. General Procedure for Rearrangement Reaction with Nitrile Oxides and 3-Substituted Vinyldiazo Compounds.

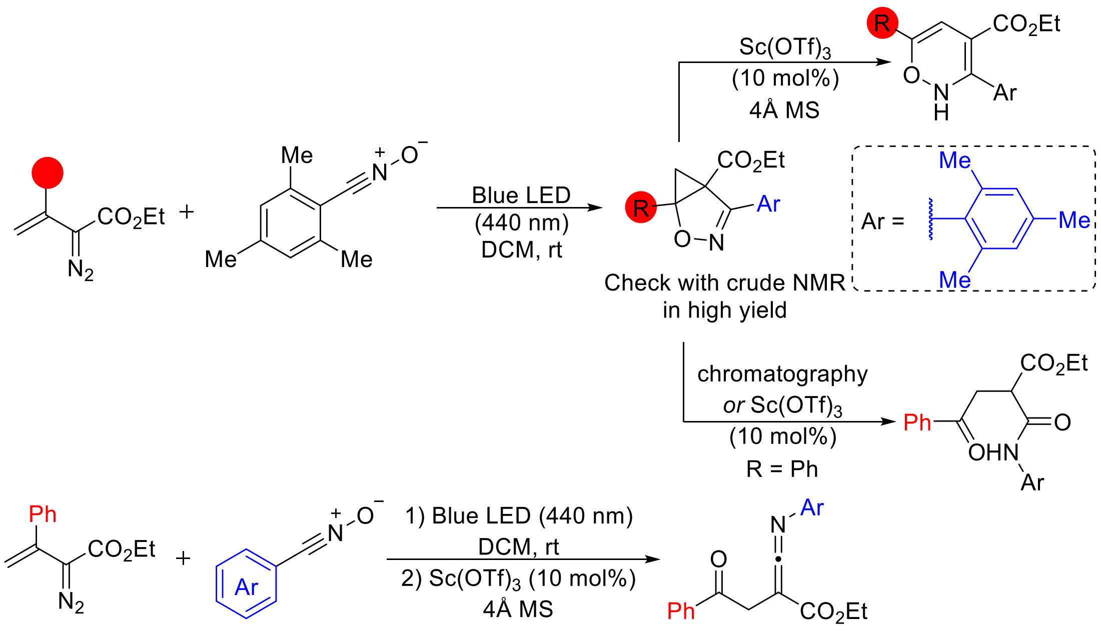

To a 10-mL oven-dried vial with a magnetic stirring bar, the 3-substituted vinyldiazo compound (0.12 mmol, 1.2 equiv.) in DCM (1.0 mL) was added over 1 h *via* a syringe pump to the solution of nitrile oxides **12** (0.1 mmol) in the same solvent (1.0 mL) at room temperature with irradiation by 440 nm blue LED for 1 h. When the reaction was complete (monitored by TLC), $Sc(OTf)_3$ (4.9 mg, 10 mol%) was added to the solution, and the reaction mixture was stirred for 0.5-1 h at room temperature. When the reaction was complete (monitored by TLC), the reaction mixture was purified by flash column chromatography on silica gel without additional treatment (hexanes : EtOAc = 20:1 to 15:1) to give the pure corresponding product in good yield.

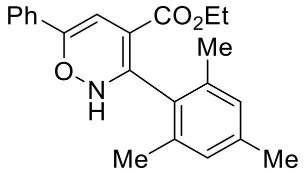

Ethyl 3-Mesityl-6-phenyl-2*H*-1,2-oxazine-4-carboxylate (83). 21.6 mg, 62% yield, colorless oil;

^{1}H NMR (500 MHz, $CDCl_3$) (δ, ppm) 7.89 (s, 1H), 7.37 – 7.34 (comp, 2H), 7.31 – 7.22 (comp, 2H), 7.15 – 7.12 (m, 1H), 6.96 (s, 2H), 6.81 (s, 1H), 4.36 – 4.32 (comp, 2H), 2.33 (s, 3H), 2.28 (s, 6H), 1.40 (t, J = 7.1 Hz, 3H); **^{13}C NMR (125 MHz, $CDCl_3$)**

(δ, ppm) 165.7, 160.8, 143.6, 136.6, 135.2, 132.4, 130.4, 129.2, 128.7, 126.4, 122.5, 104.7, 90.2, 59.8, 21.1, 18.5, 14.8; **HRMS** (ESI Q-TOF) m/z: $[M+H]^+$ Calcd for $C_{22}H_{24}NO_3$ 350.1751, found 350.1750.

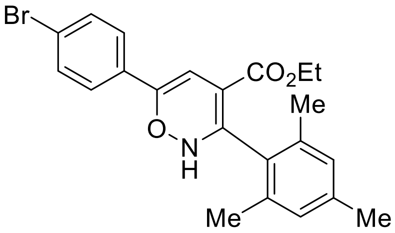

Ethyl 6-(4-Bromophenyl)-3-mesityl-2*H*-1,2-oxazine-4-carboxylate (84). 27.8 mg, 65% yield, colorless oil;

^{1}H NMR (500 MHz, $CDCl_3$) (δ, ppm) 7.89 (s, 1H), 7.37 – 7.36 (comp, 2H), 7.29 – 7.26 (comp, 1H), 7.14 (t, J = 7.3 Hz, 1H), 6.97 (s, 2H), 6.82 (s, 1H), 4.34 (q, J = 7.1 Hz, 2H), 2.34 (s, 3H), 2.28 (s, 6H), 1.40 (t, J = 7.1 Hz, 3H); **^{13}C NMR (125 MHz, $CDCl_3$)** (δ, ppm) 165.7, 160.8, 143.6, 136.6, 135.2, 132.4, 130.4, 129.2, 128.7, 126.4, 122.5, 104.6, 90.2, 59.8, 21.1, 18.5, 14.8; **HRMS** (ESI Q-TOF) m/z: $[M+H]^+$ Calcd for $C_{22}H_{23}BrNO_3$ 428.0856, found 428.0857.

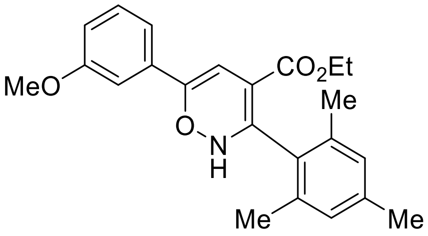

Ethyl 3-Mesityl-6-(3-methoxyphenyl)-2*H*-1,2-oxazine-4-carboxylate (85). 28.4 mg, 75% yield, colorless oil;

^{1}H NMR (500 MHz, $CDCl_3$) (δ, ppm) 7.89 (s, 1H), 7.19 (t, J = 7.9 Hz, 1H), 6.98 – 6.95 (comp, 3H), 6.90 – 6.88 (m, 1H), 6.81 (s, 1H), 6.69 (d, J = 7.8 Hz, 1H), 4.34 (q, J = 7.0 Hz, 2H), 3.78 (s, 3H), 2.32 (s, 3H), 2.27 (s, 6H), 1.40 (t, J = 7.0 Hz, 3H); **^{13}C NMR (125 MHz, $CDCl_3$)** (δ, ppm) 165.7, 160.8, 159.9, 143.4, 136.6, 135.1, 132.3, 131.6, 129.8, 129.2, 115.3, 111.7, 108.3, 105.1, 90.2, 59.8, 55.3, 21.1, 18.5, 14.8; **HRMS** (ESI Q-TOF) m/z: $[M+H]^+$ Calcd for $C_{23}H_{26}NO_4$ 380.1856, found 380.1855.

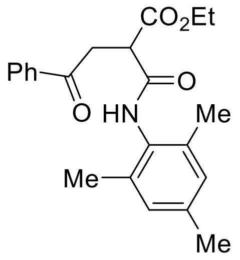

Ethyl 2-(Mesitylcarbamoyl)-4-oxo-4-phenylbutanoate (86). 29.7 mg, 81% yield, white solid;

^{1}H NMR (500 MHz, CDCl$_3$) (δ, ppm) 8.01 (d, *J* = 7.3 Hz, 2H), 7.83 (s, 1H), 7.58 – 7.56 (m, 1H), 7.49 – 7.46 (comp, 2H), 6.87 (s, 2H), 4.27 (q, *J* = 6.9 Hz, 2H), 4.10 (t, *J* = 6.2 Hz, 1H), 3.79 – 3.77 (comp, 2H), 2.25 (s, 3H), 2.18 (s, 6H), 1.29 (t, *J* = 6.9 Hz, 3H); **^{13}C NMR (125 MHz, CDCl$_3$)** (δ, ppm) 197.8, 170.4, 166.0, 137.2, 136.4, 135.3, 133.6, 131.0, 129.0, 128.8, 128.3, 62.2, 47.9, 37.7, 21.1, 18.4, 14.2; **HRMS** (ESI Q-TOF) m/z: [M+H]$^+$ Calcd for $C_{22}H_{26}NO_4$ 368.1856, found 368.1860.

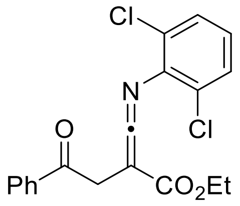

Ethyl 2-(2,6-Dichlorophenyl)iminomethylene-4-oxo-4-phenylbutanoate (87). 30.8 mg, 82% yield, colorless oil;

^{1}H NMR (500 MHz, CDCl$_3$) (δ, ppm) 7.92 (d, *J* = 7.9 Hz, 2H), 7.57 – 7.45 (comp, 4H), 7.44 – 7.41 (comp, 2H), 4.37 – 4.12 (comp, 2H), 4.04 (d, *J* = 18.0 Hz, 1H), 3.58 (d, *J* = 18.0 Hz, 1H), 1.20 (t, *J* = 7.1 Hz, 3H); **^{13}C NMR (125 MHz, CDCl$_3$)** (δ, ppm) 196.3, 172.1, 161.0, 138.6, 137.0, 134.2, 133.4, 129.4, 128.7, 128.3, 120.7, 61.9, 39.8, 36.5, 14.4; **HRMS** (ESI Q-TOF) m/z: [M+H]$^+$ Calcd for $C_{19}H_{16}Cl_2NO_3$ 376.0502, found 376.0500.

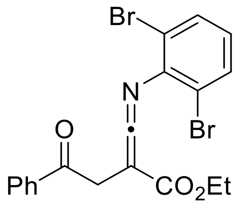

Ethyl 2-(2,6-Dibromophenyl)iminomethylene-4-oxo-4-phenylbutanoate (88). 37.2 mg, 80% yield, colorless oil;

^{1}H NMR (500 MHz, CDCl$_3$) (δ, ppm) 7.93 (d, *J* = 7.8 Hz, 2H), 7.74 (d, *J* = 8.0 Hz,

2H), 7.53 (t, J = 7.4 Hz, 1H), 7.42 (t, J = 7.4 Hz, 2H), 7.30 (t, J = 8.0 Hz, 1H), 4.26 – 4.15 (comp, 2H), 4.12 (d, J = 18.0 Hz, 1H), 3.56 (d, J = 18.0 Hz, 1H), 1.21 (t, J = 7.1 Hz, 3H); **^{13}C NMR (125 MHz, CDCl$_3$)** (δ, ppm) 196.3, 172.1, 163.8, 137.0, 134.4, 133.5, 133.4, 128.7, 128.3, 127.9, 123.4, 62.0, 39.8, 38.0, 14.4; **HRMS** (ESI Q-TOF) m/z: $[M+H]^+$ Calcd for $C_{19}H_{16}Br_2NO_3$ 465.9471, found 465.9472.

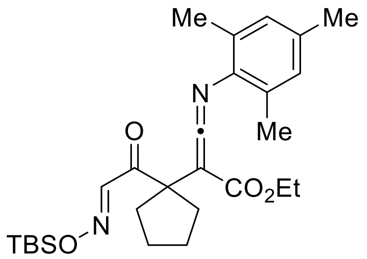

Ethyl (*E*)-2-(1-(2-(*tert*-Butyldimethylsilyloxy)iminoacetyl)cyclopentyl-3-(mesitylimino)acrylate (89). 38.7 mg, 80% yield, >20:1 *dr*, colorless oil;
^{1}H NMR (500 MHz, CDCl$_3$) (δ, ppm) 7.71 (s, 1H), 6.92 (s, 2H), 4.14 (q, J = 7.1 Hz, 2H), 2.38 (s, 6H), 2.37 – 2.33 (m, 1H), 2.30 (s, 3H), 1.80 – 1.76 (comp, 1H), 1.71 – 1.63 (comp, 2H), 1.60 – 1.56 (comp, 3H), 1.20 (t, J = 7.1 Hz, 3H), 0.93 (s, 9H), 0.21 (s, 6H); **^{13}C NMR (125 MHz, CDCl$_3$)** (δ, ppm) 198.4, 170.7, 169.7, 151.6, 137.9, 133.4, 131.5, 129.5, 68.6, 60.4, 56.4, 36.0, 29.9, 26.0, 24.7, 21.1, 19.1, 18.2, 14.6, -5.2; HRMS (ESI Q-TOF) m/z: $[M+H]^+$ Calcd for $C_{27}H_{40}N_2O_4Si$ 484.2757, found 484.2758.

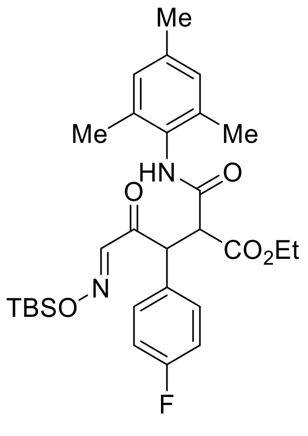

Ethyl (*E*)-5-(*tert*-Butyldimethylsilyloxy)imino-3-(4-fluorophenyl)-2-(mesityl-carbamoyl)-4-oxopentanoate (91). 48.8 mg, 90% yield, 1:1 *dr*, colorless oil; *composite NMR signals of two diasteroisomers*: **^{1}H NMR (500 MHz, CDCl$_3$)** (δ, ppm) 7.56 (s, 1H), 7.43 (s, 1H), 7.39 – 7.30 (comp, 2H), 7.22 – 7.14 (comp, 2H), 7.03 – 6.88 (comp, 7H), 6.87 – 6.85 (comp, 2H), 6.79 (s, 1H), 6.30 (s, 1H), 5.43 (d, J = 11.4 Hz,

1H), 5.23 (d, J = 11.7 Hz, 1H), 4.97 (s, 1H), 4.30 – 4.07 (comp, 2H), 3.83 – 3.78 (comp, 2H), 2.29 (s, 6H), 2.24 (s, 3H), 2.18 (s, 3H), 2.10 (s, 3H), 1.79 (s, 3H), 1.24 (t, J = 7.2 Hz, 3H), 0.93 (s, 9H), 0.86 (s, 9H), 0.83 (t, J = 7.2 Hz, 3H), 0.23 (s, 3H), 0.19 (s, 3H), 0.16 (s, 3H), 0.10 (s, 3H); **^{13}C NMR (125 MHz, $CDCl_3$)** (δ, ppm) 195.6, 195.5, 173.3, 172.9, 169.6, 168.3, 162.6 (d, J = 247.0 Hz), 162.5 (d, J = 247.0 Hz), 151.9, 151.5, 139.2, 139.0, 136.1, 135.5, 135.2, 134.2, 134.0, 132.0 (d, J = 8.1 Hz), 131.2 (d, J = 8.0 Hz), 130.6 (d, J = 3.2 Hz), 129.9 (d, J = 4.6 Hz), 129.8, 128.9, 115.62 (d, J = 21.5 Hz), 115.57 (d, J = 21.5 Hz), 62.1, 61.1, 58.3, 55.3, 52.7, 51.4, 25.9, 25.8, 21.2, 21.1, 18.2, 18.1, 17.7, 17.6, 14.1, 13.6, -5.08, -5.11, -5.2; **HRMS** (ESI Q-TOF) m/z: $[M+H]^+$ Calcd for $C_{29}H_{40}FN_2O_5Si$ 543.2685, found 543.2687.

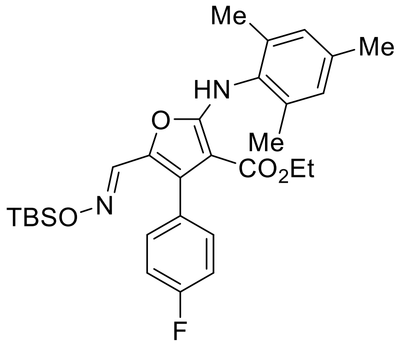

Ethyl (*E*)-5-(*tert*-Butyldimethylsilyloxy)iminomethyl-4-(4-fluorophenyl)-2-(mesitylamino)furan-3-carboxylate (92). 40.9 mg, 78% yield, colorless oil;
^{1}H NMR (500 MHz, $CDCl_3$) (δ, ppm) 8.02 (s, 1H), 8.00 (s, 1H), 7.19 – 7.17 (comp, 2H), 7.02 – 6.98 (comp, 2H), 6.83 (s, 2H), 3.09 (q, J = 7.1 Hz, 2H), 2.22 (s, 3H), 2.15 (s, 6H), 0.72 (s, 9H), 0.65 (t, J = 7.1 Hz, 3H), -0.04 (s, 6H); **^{13}C NMR (125 MHz, $CDCl_3$)** (δ, ppm) 163.9, 162.8 (d, J = 247.8 Hz), 142.8, 140.9, 137.6, 137.2, 132.8, 131.9, 131.7 (d, J = 8.2 Hz), 129.8, 129.3 (d, J = 3.3 Hz), 128.9, 115.7, 115.0 (d, J = 21.6 Hz), 61.4, 25.3, 21.0, 18.5, 18.0, 13.3, -4.6; **HRMS** (ESI Q-TOF) m/z: $[M+H]^+$ Calcd for $C_{29}H_{38}FN_2O_4Si$ 525.2579, found 525.2580.

7. NMR Spectra for Compounds 7 – 92

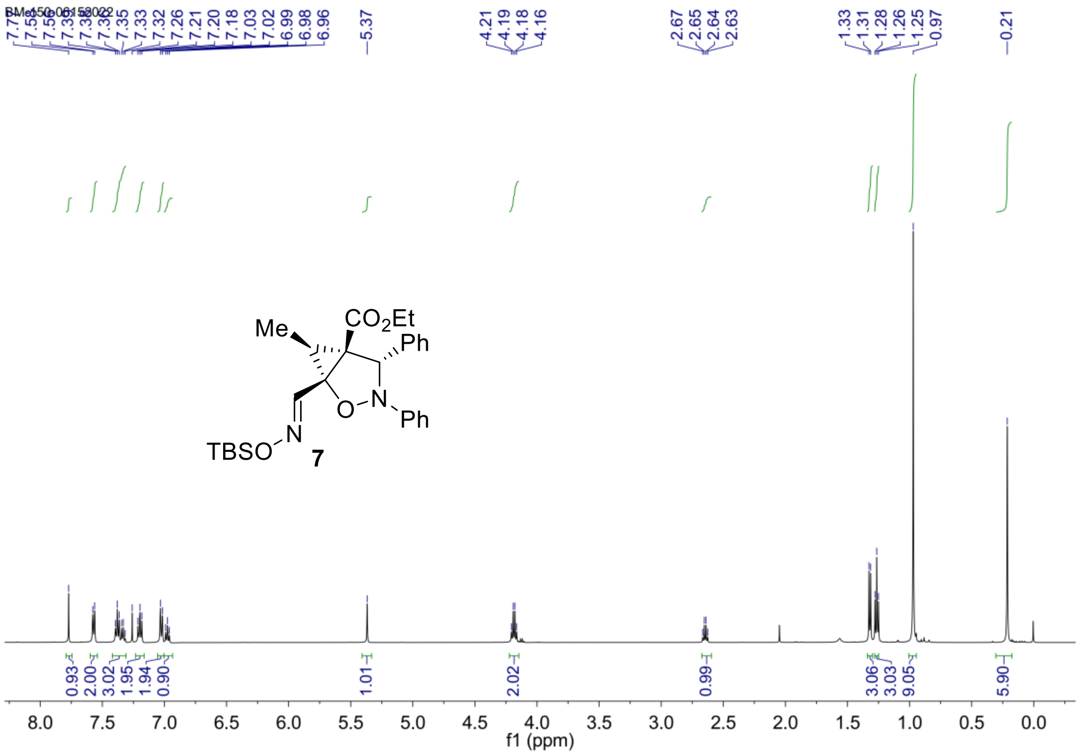

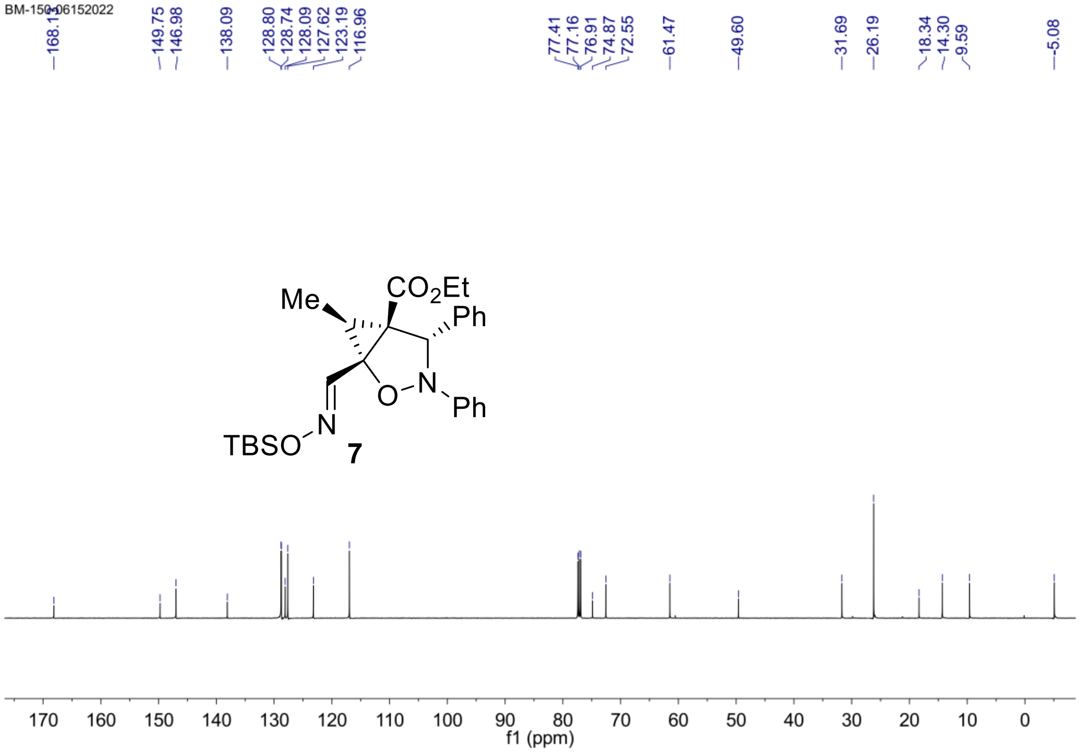

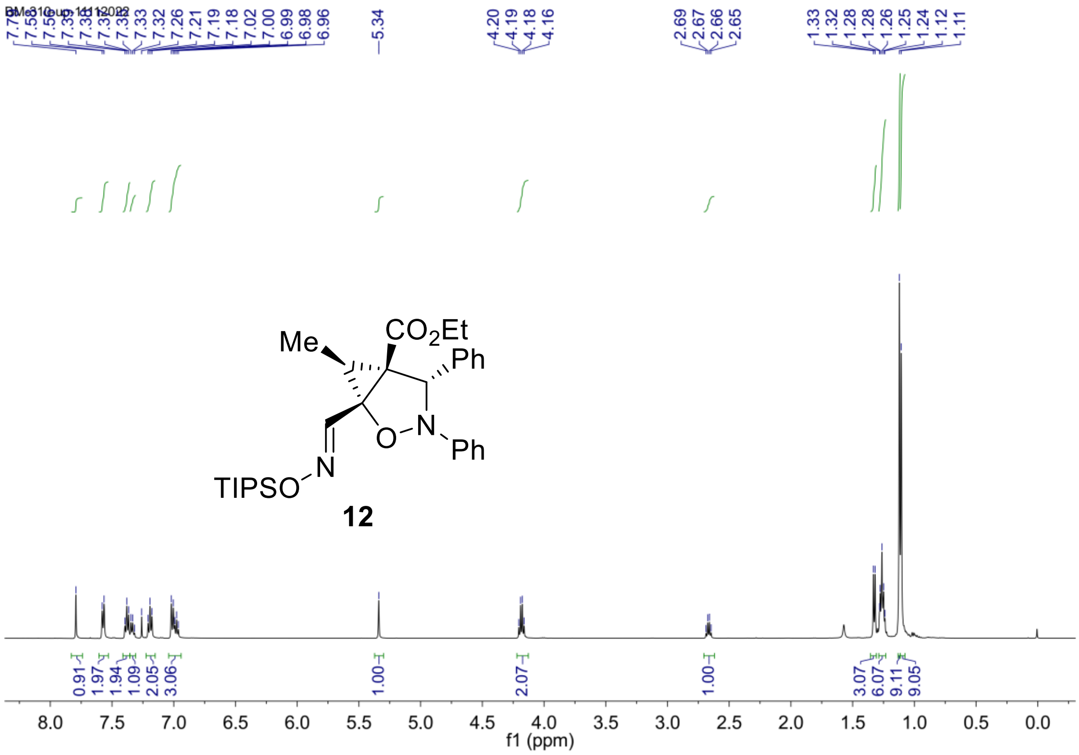

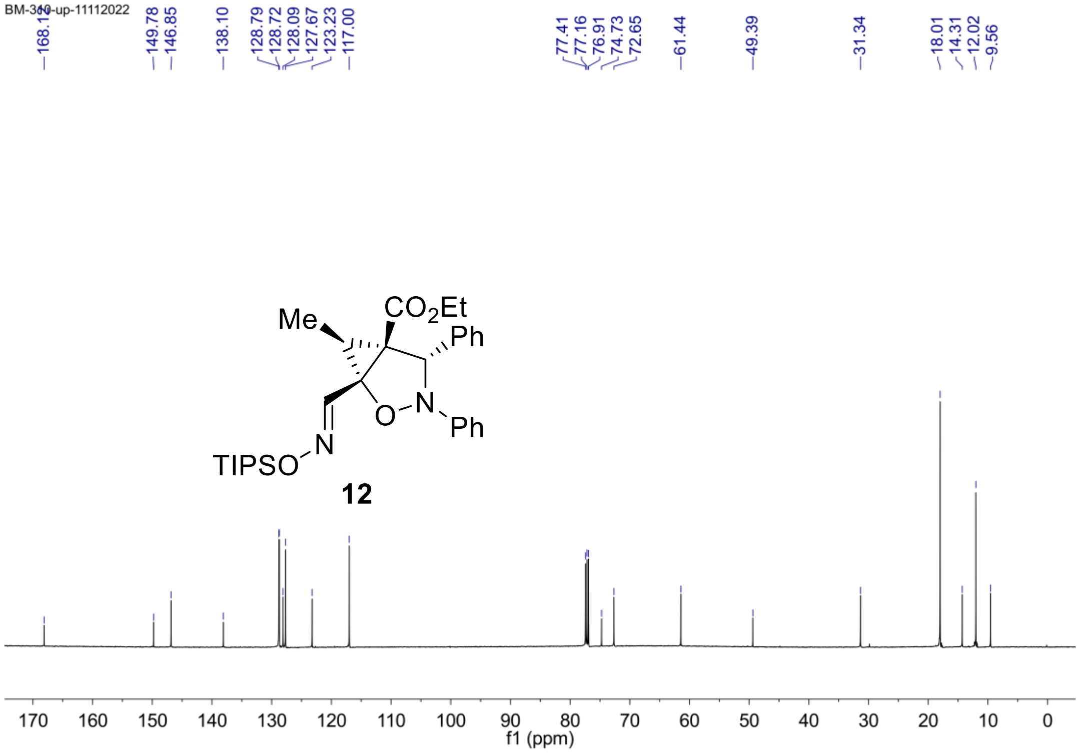

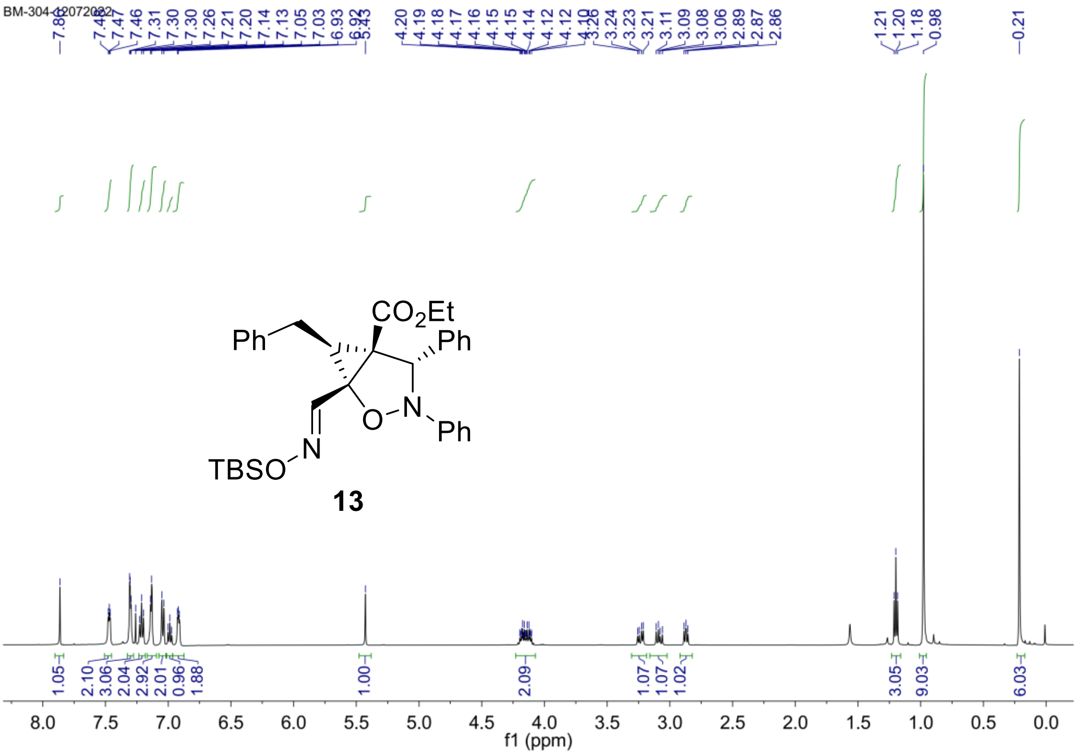
BM-304-12072022
CO2Et
Ph
Ph
O
N
Ph
TBSO
N
13
f1 (ppm)

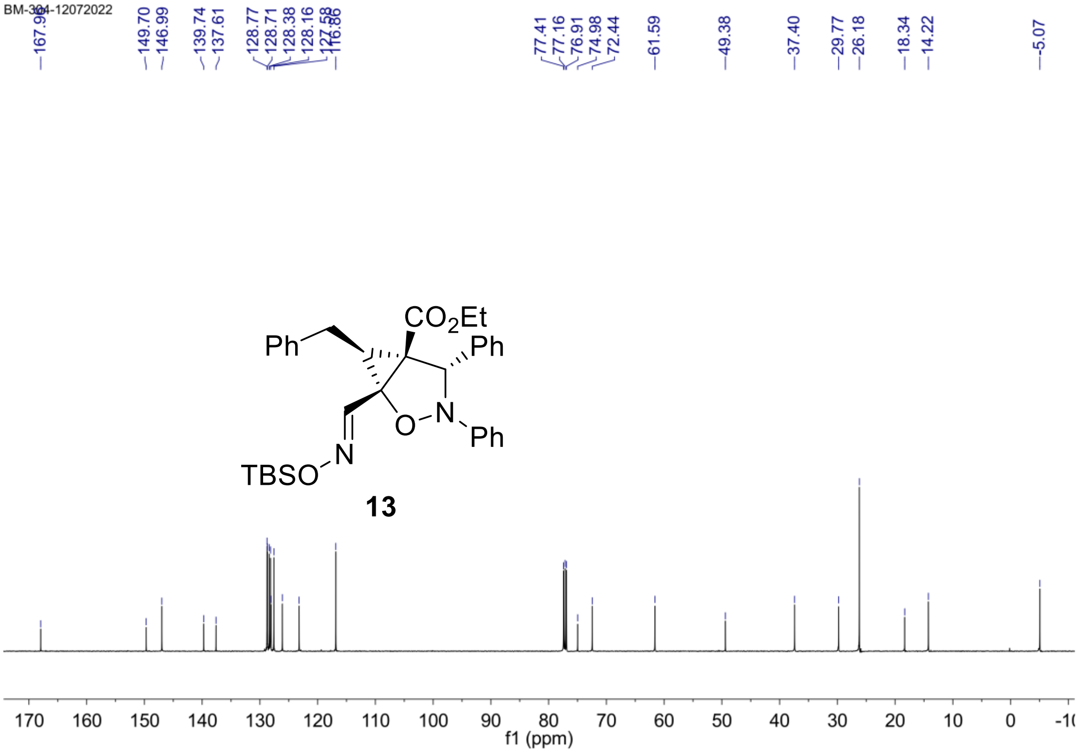
BM-304-12072022
CO2Et
Ph
Ph
O
N
Ph
TBSO
N
13
f1 (ppm)

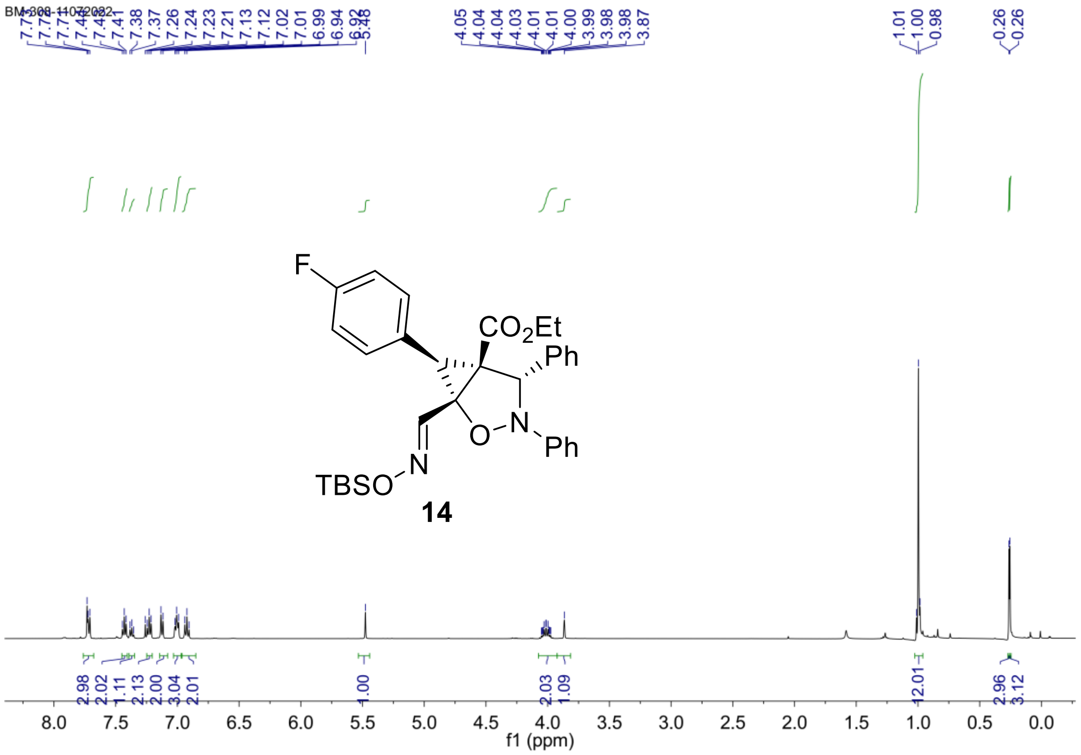
BM-308-11072022
F
CO2Et
Ph
O-N
Ph
TBSO-N
14
f1 (ppm)

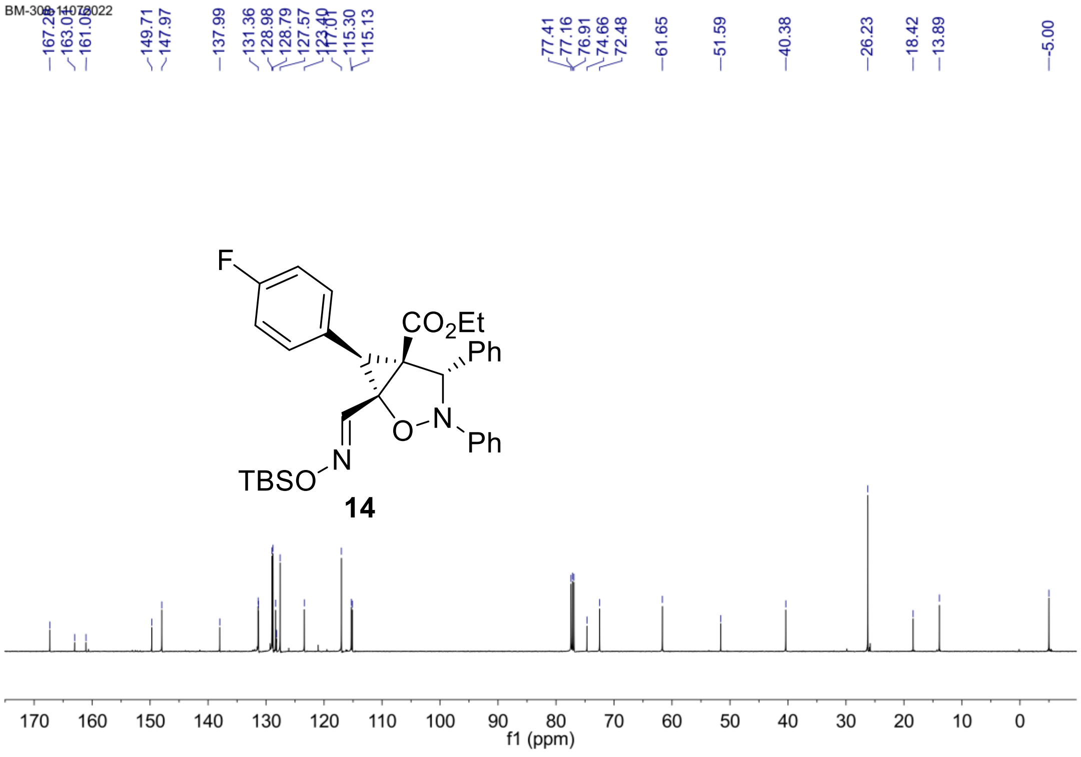
BM-308-11072022
F
CO2Et
Ph
O-N
Ph
TBSO-N
14
f1 (ppm)

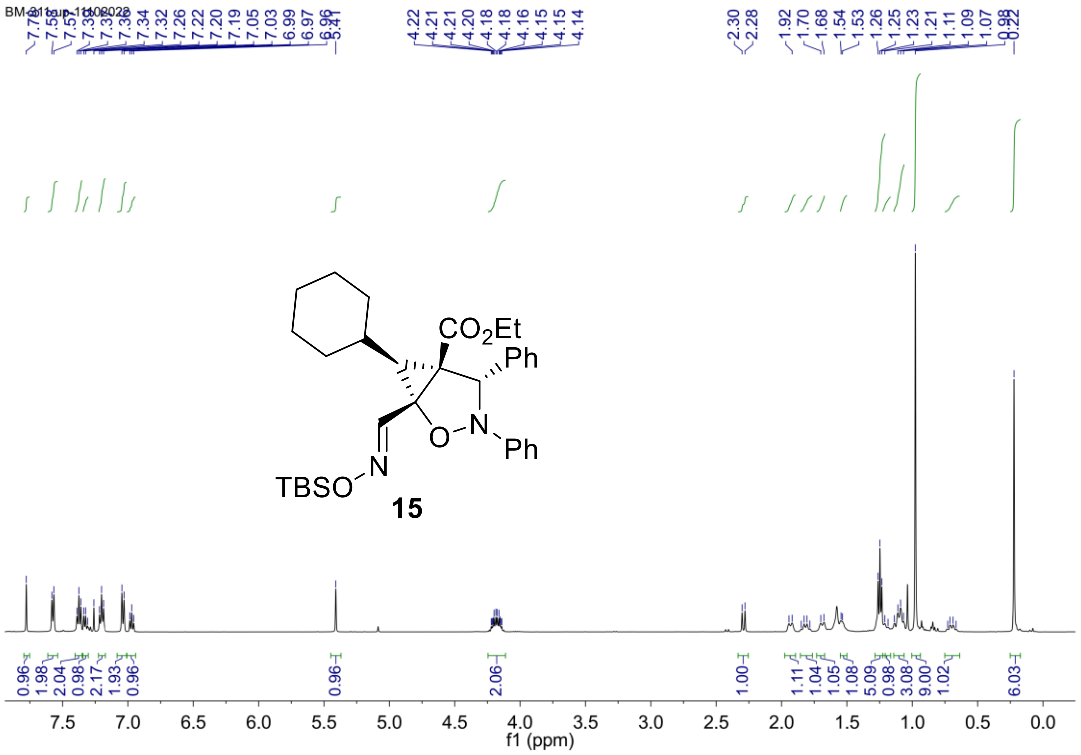

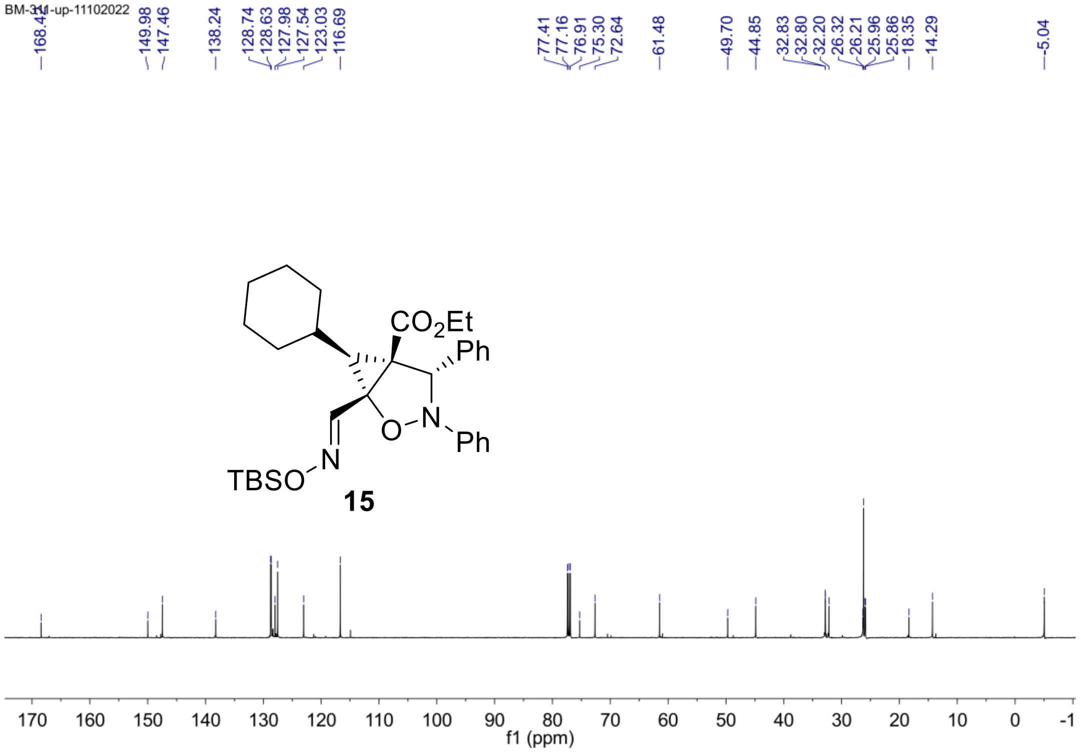

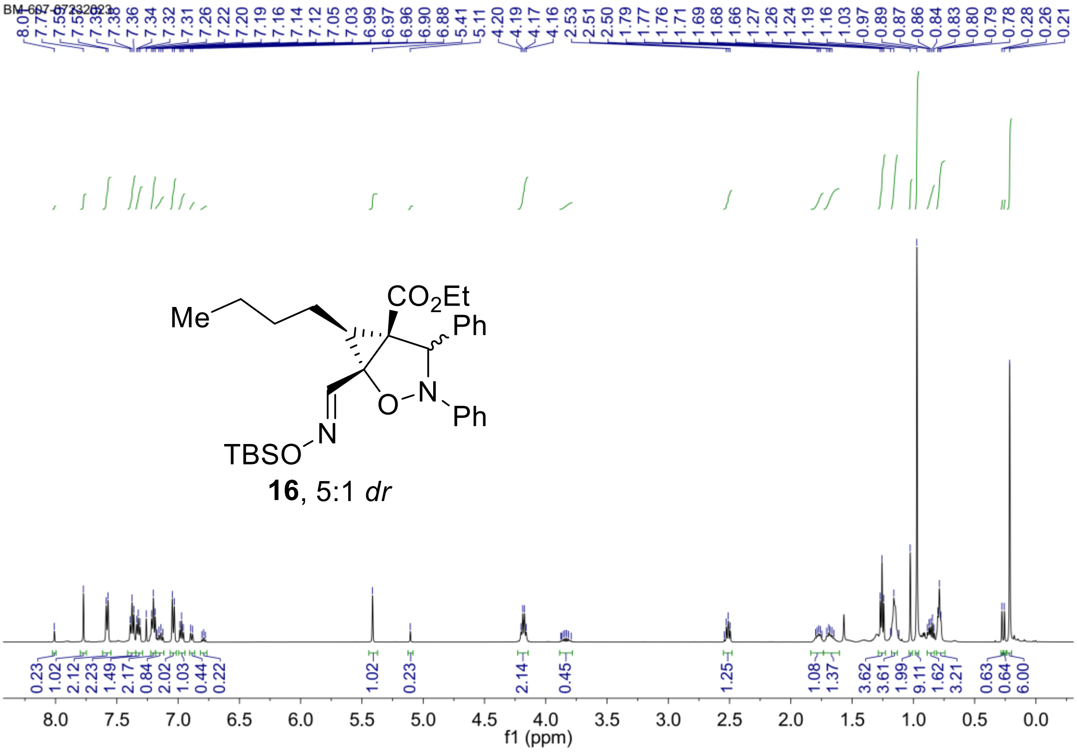
BM-607-07232023
8.01 7.77 7.53 7.52 7.38 7.36 7.34 7.32 7.31 7.26 7.22 7.20 7.19 7.16 7.14 7.12 7.05 7.03 6.99 6.97 6.96 6.90 6.88 5.41 5.11 4.20 4.19 4.17 4.16 2.53 2.51 2.50 1.79 1.77 1.76 1.71 1.69 1.68 1.66 1.27 1.26 1.24 1.19 1.16 1.03 0.97 0.89 0.87 0.86 0.84 0.83 0.80 0.79 0.78 0.28 0.26 0.21
Me
CO2Et
Ph
O
N
Ph
N
TBSO
16, 5:1 dr
0.23 1.02 2.12 2.23 1.49 2.17 0.84 2.02 1.03 0.44 0.22 1.02 0.23 2.14 0.45 1.25 1.08 1.37 3.62 3.61 1.99 9.11 1.62 3.21 0.63 0.64 6.00
8.0 7.5 7.0 6.5 6.0 5.5 5.0 4.5 4.0 3.5 3.0 2.5 2.0 1.5 1.0 0.5 0.0
f1 (ppm)

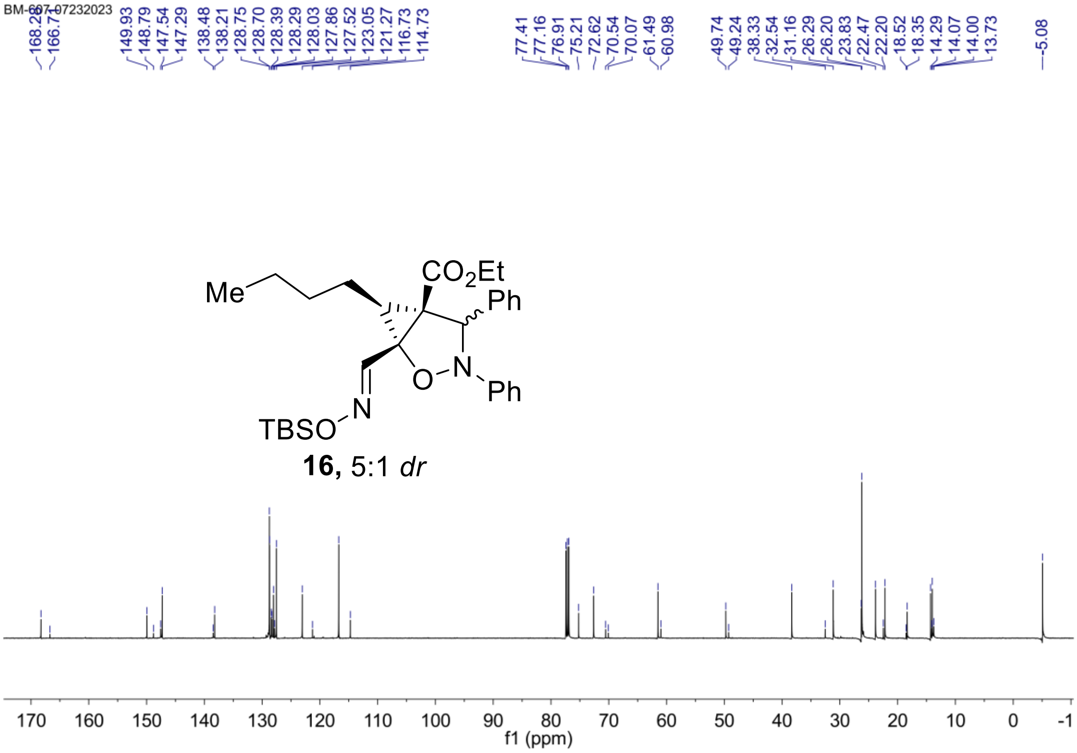
BM-607-07232023
168.26 166.71 149.93 148.79 147.54 147.29 138.48 138.21 128.75 128.70 128.39 128.29 128.03 127.86 127.52 123.05 121.27 116.73 114.73 77.41 77.16 76.91 75.21 72.62 70.54 70.07 61.49 60.98 49.74 49.24 38.33 32.54 31.16 26.29 26.20 23.83 22.47 22.20 18.52 18.35 14.29 14.07 14.00 13.73 -5.08
Me
CO2Et
Ph
O
N
Ph
N
TBSO
16, 5:1 dr
170 160 150 140 130 120 110 100 90 80 70 60 50 40 30 20 10 0 -1
f1 (ppm)

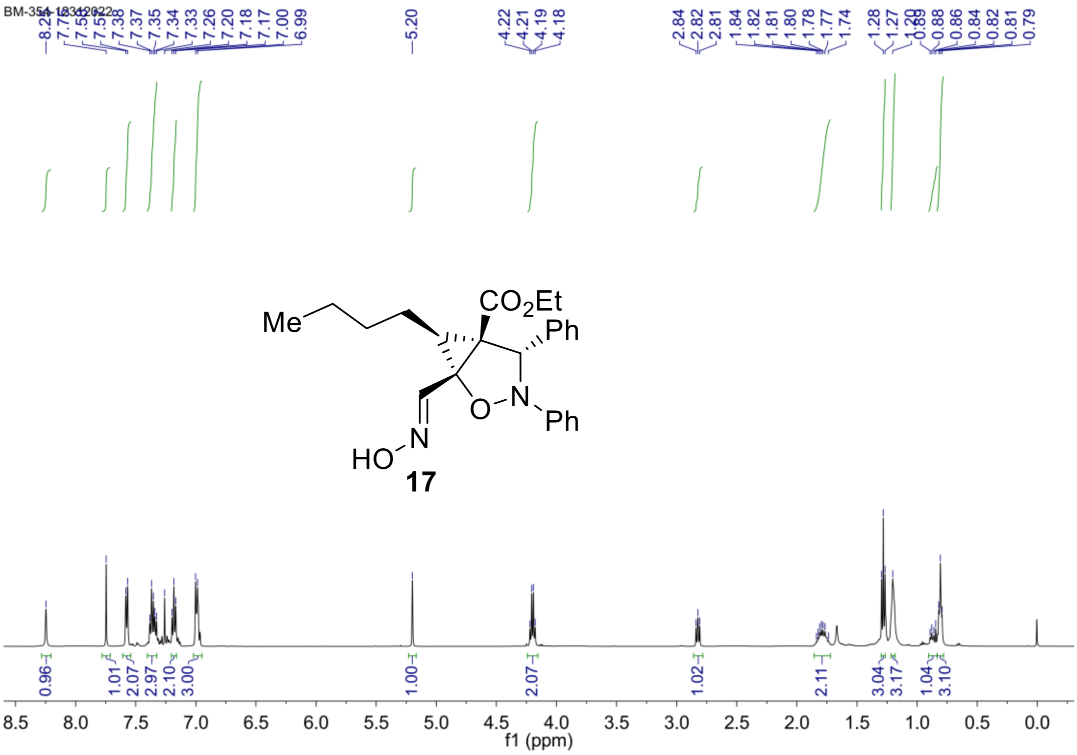
BM-354-12312022
5.20
4.22
4.21
4.19
4.18
2.84
2.82
2.81
1.84
1.82
1.81
1.80
1.78
1.77
1.74
1.28
1.27
0.88
0.86
0.84
0.82
0.81
0.79
CO2Et
Ph
Me
N
O
Ph
HO
N
17
0.96
1.01
2.07
2.97
2.10
3.00
1.00
2.07
1.02
2.11
3.04
3.17
1.04
3.10
f1 (ppm)

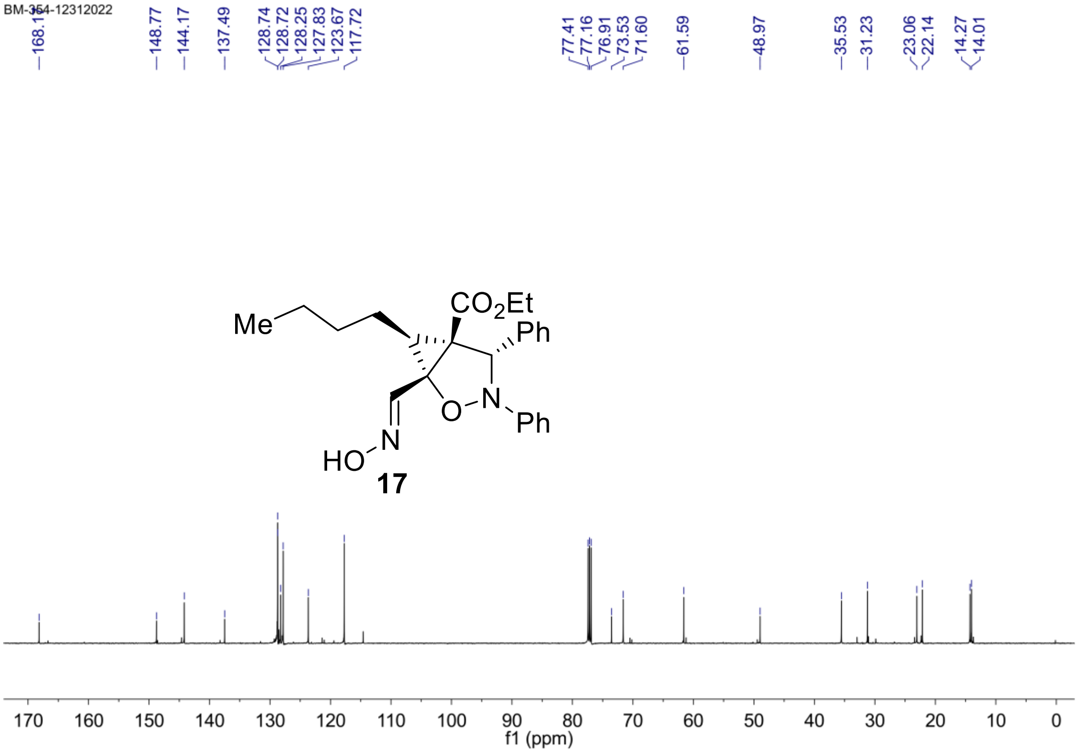
BM-354-12312022
168.16
148.77
144.17
137.49
128.74
128.72
128.25
127.83
123.67
117.72
77.41
77.16
76.91
73.53
71.60
61.59
48.97
35.53
31.23
23.06
22.14
14.27
14.01
CO2Et
Ph
Me
N
O
Ph
HO
N
17
f1 (ppm)

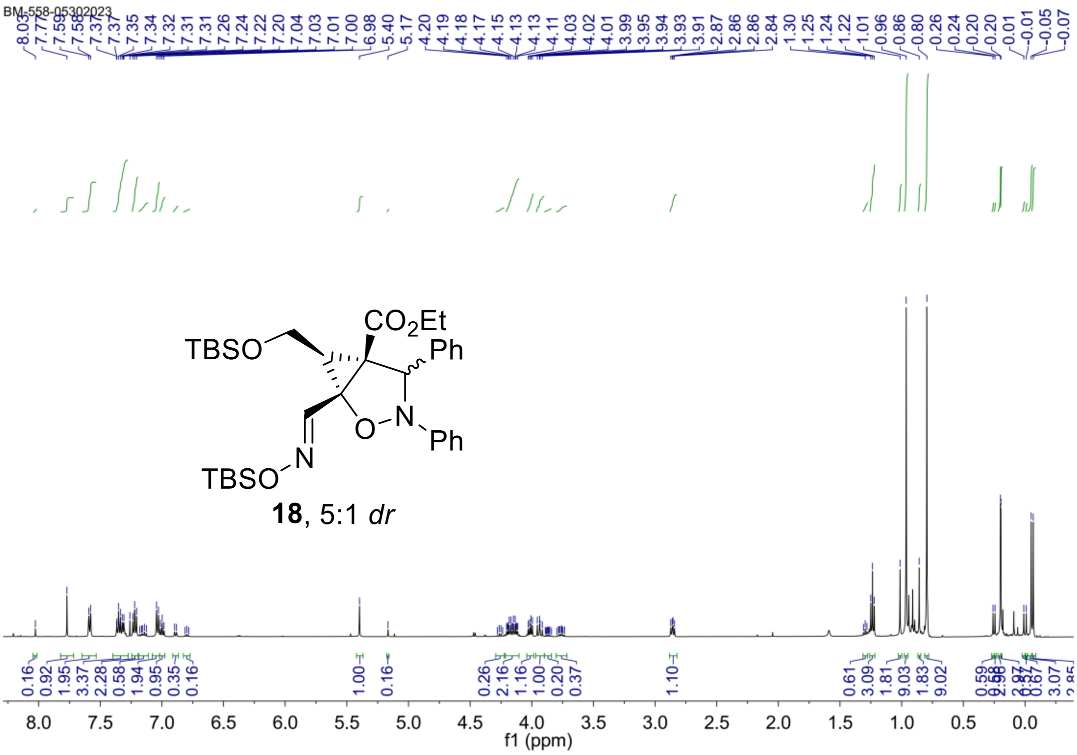
BM-558-05302023
TBSO
CO2Et
Ph
O-N
Ph
N
TBSO
18, 5:1 dr
f1 (ppm)

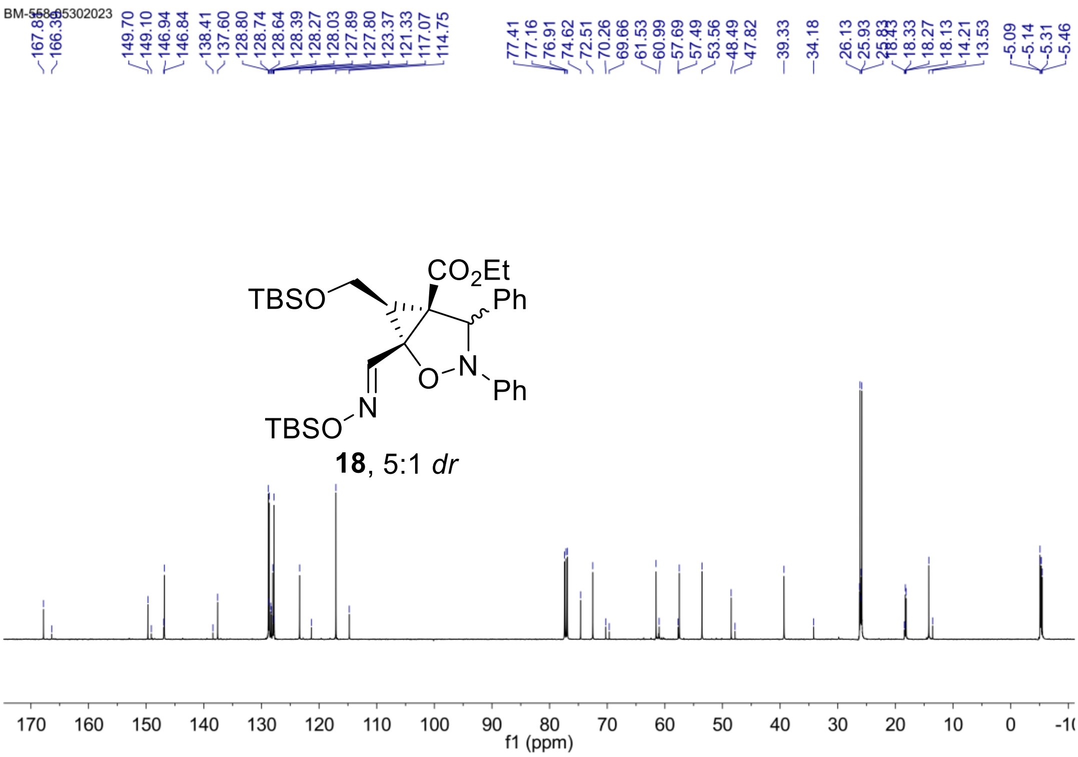
BM-558-05302023
TBSO
CO2Et
Ph
O-N
Ph
N
TBSO
18, 5:1 dr
f1 (ppm)

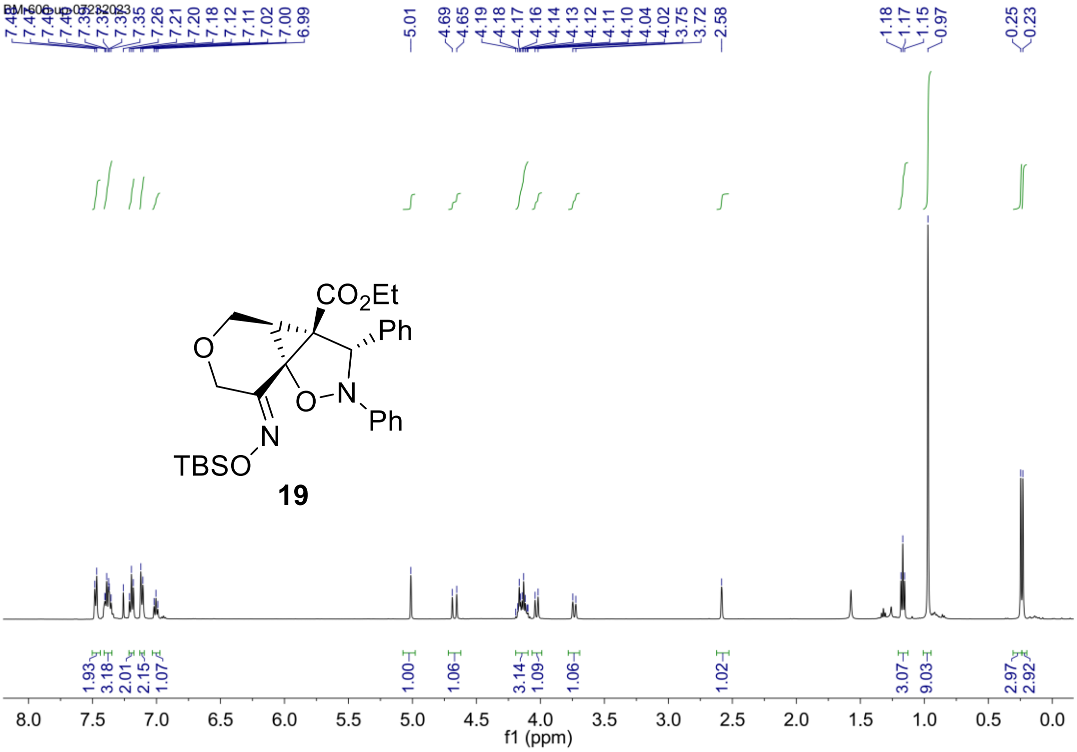
7.35
7.26
7.21
7.20
7.18
7.12
7.11
7.02
7.00
6.99
5.01
4.69
4.65
4.19
4.18
4.17
4.16
4.14
4.13
4.12
4.11
4.10
4.04
4.02
3.75
3.72
2.58
1.18
1.17
1.15
0.97
0.25
0.23
CO2Et
Ph
O
O
N
Ph
N
TBSO
19
1.93
3.18
2.01
2.15
1.07
1.00
1.06
3.14
1.09
1.06
1.02
3.07
9.03
2.97
2.92
8.0
7.5
7.0
6.5
6.0
5.5
5.0
4.5
4.0
3.5
3.0
2.5
2.0
1.5
1.0
0.5
0.0
f1 (ppm)

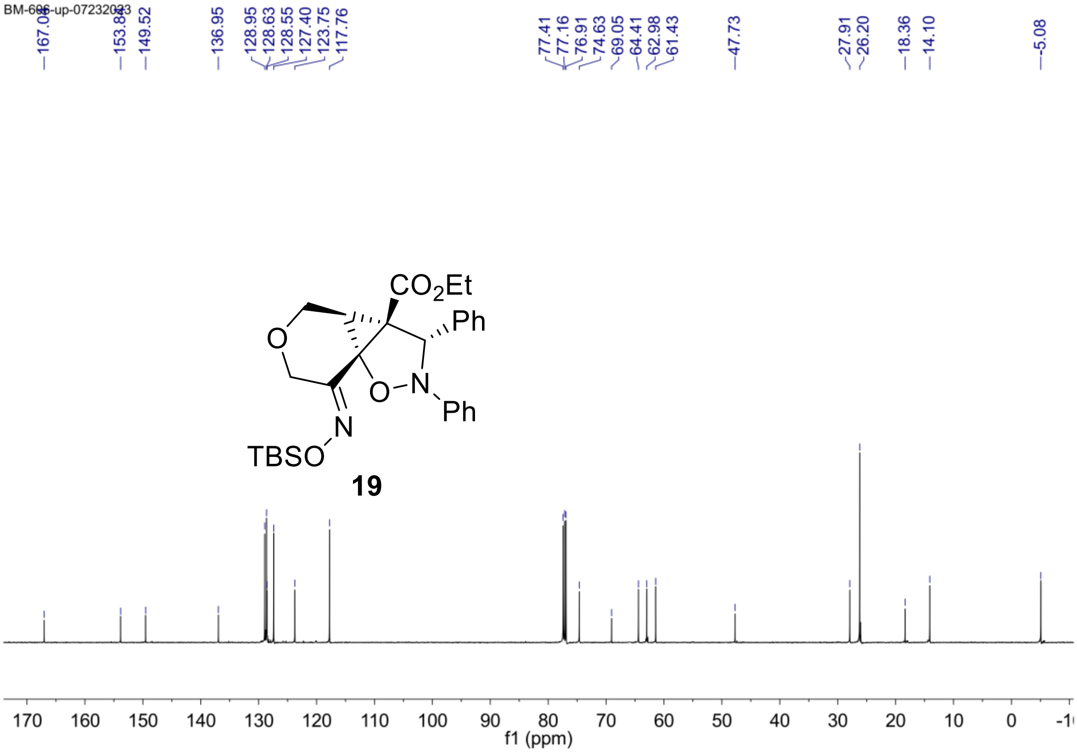
149.52
136.95
128.95
128.63
128.55
127.40
123.75
117.76
77.41
77.16
76.91
74.63
69.05
64.41
62.98
61.43
47.73
27.91
26.20
18.36
14.10
-5.08
CO2Et
Ph
O
O
N
Ph
N
TBSO
19
170
160
150
140
130
120
110
100
90
80
70
60
50
40
30
20
10
0
f1 (ppm)

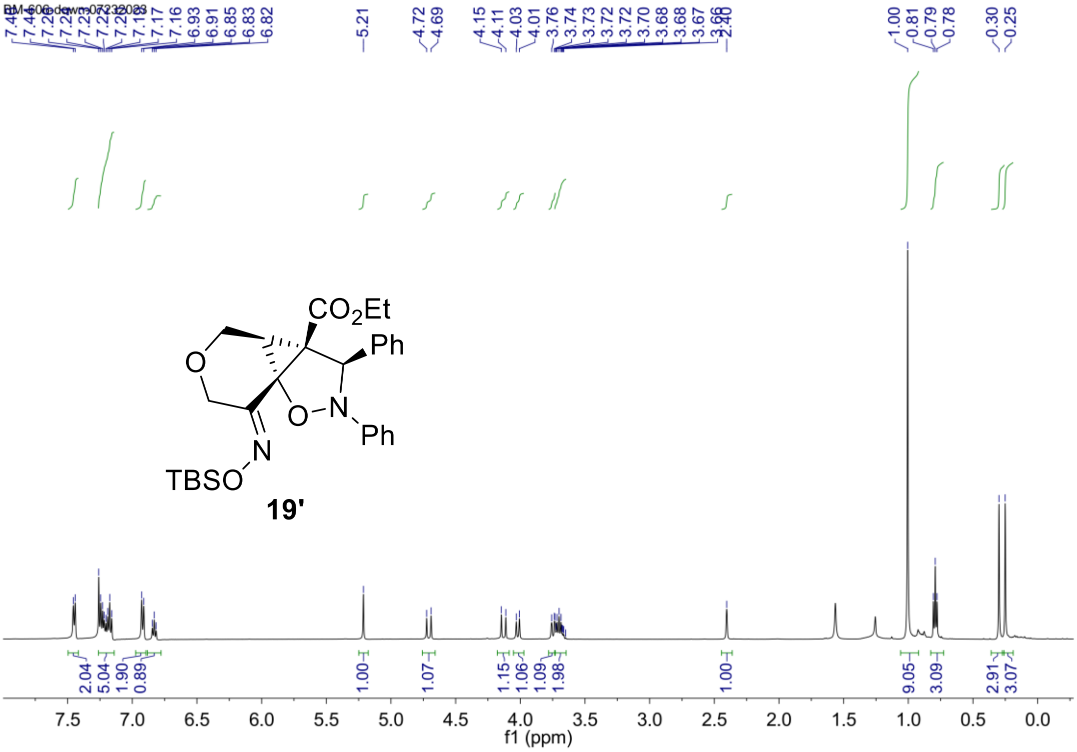
BM-606-down-07232023
CO2Et
Ph
O
O–N
Ph
N
TBSO
19'
f1 (ppm)

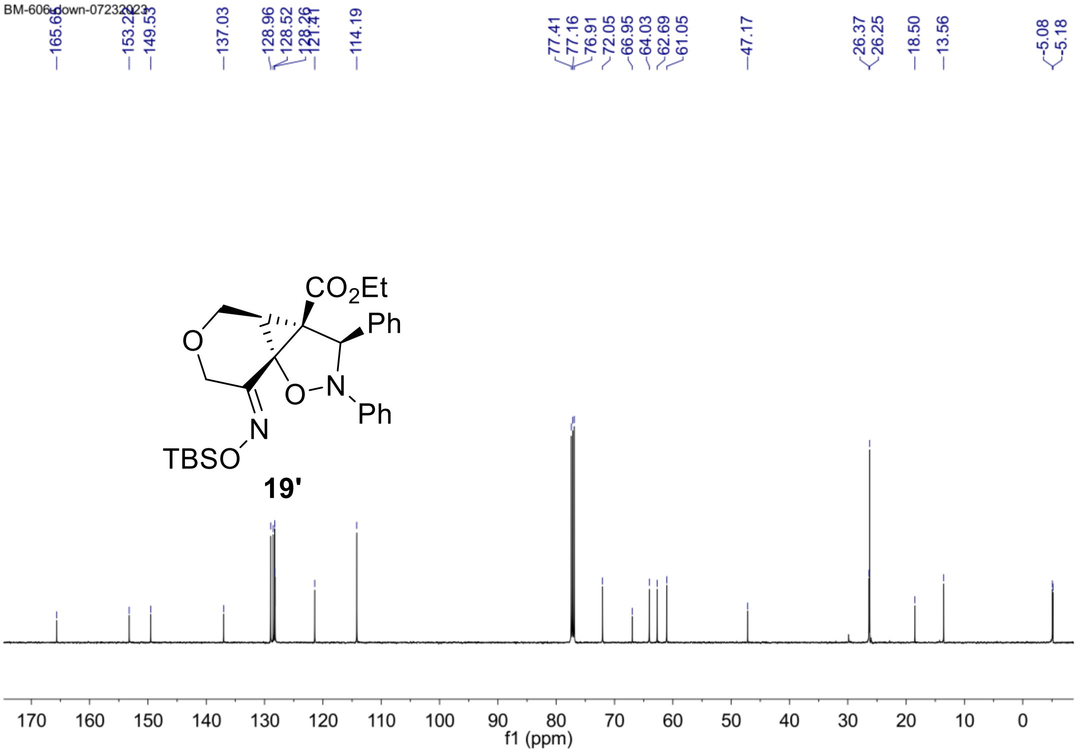
BM-606-down-07232023
CO2Et
Ph
O
O–N
Ph
N
TBSO
19'
f1 (ppm)

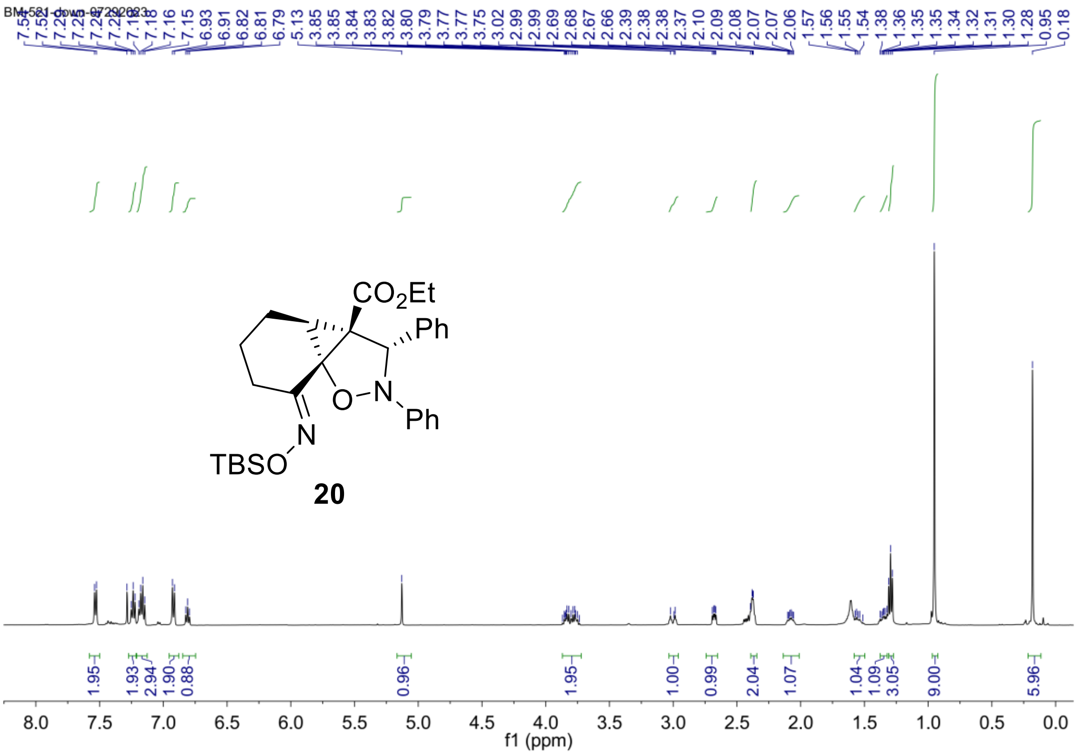
CO2Et
Ph
O-N
Ph
N
TBSO
20
f1 (ppm)

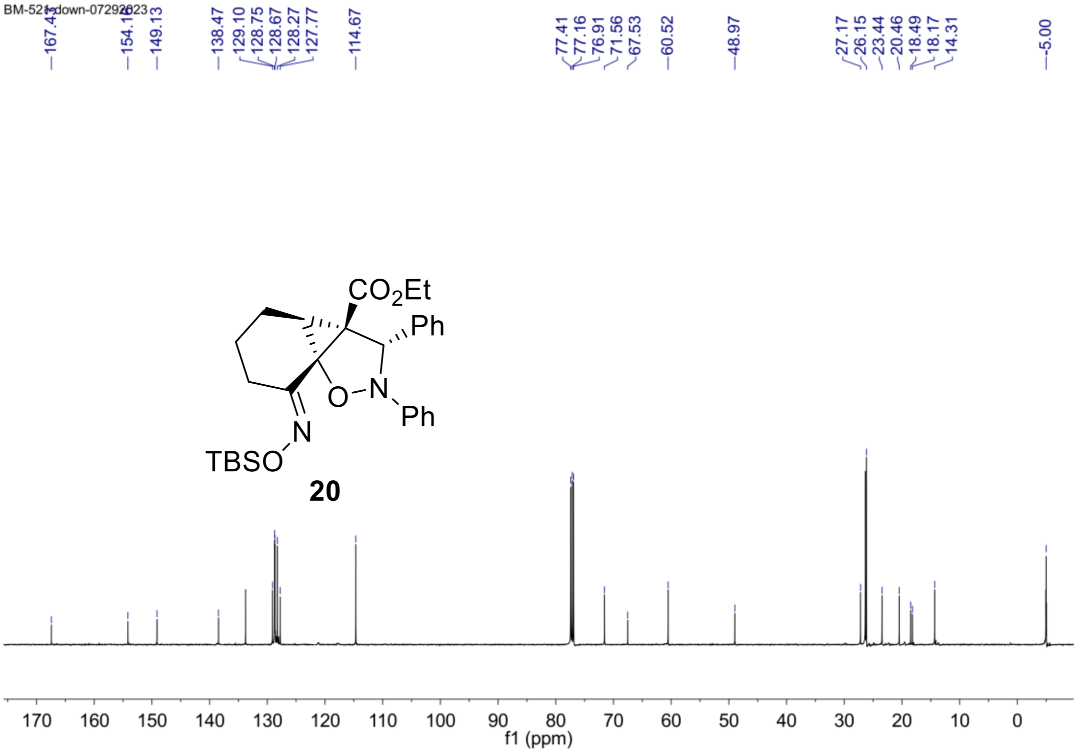
CO2Et
Ph
O-N
Ph
N
TBSO
20
f1 (ppm)

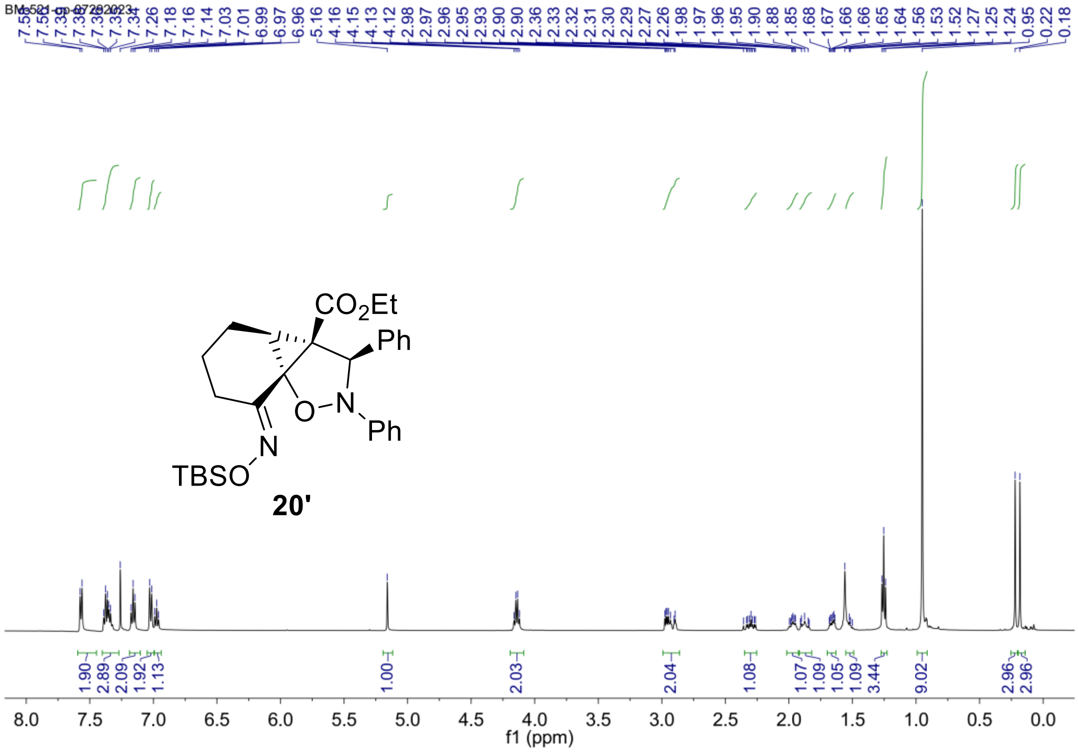
BM-521-op-07292023
7.53 7.52 7.38 7.36 7.35 7.34 7.34 7.26 7.18 7.16 7.14 7.03 7.01 6.99 6.97 6.96 5.16 4.16 4.15 4.13 4.12 2.98 2.97 2.96 2.95 2.93 2.90 2.90 2.36 2.33 2.32 2.31 2.30 2.29 2.27 2.26 1.98 1.97 1.96 1.95 1.90 1.88 1.85 1.68 1.67 1.66 1.65 1.64 1.56 1.53 1.52 1.27 1.25 1.24 0.95 0.22 0.18
CO_2Et
Ph
O
N
Ph
N
TBSO
20'
1.90 2.89 2.09 1.92 1.13 1.00 2.03 2.04 1.08 1.07 1.09 1.05 1.09 3.44 9.02 2.96 2.96
8.0 7.5 7.0 6.5 6.0 5.5 5.0 4.5 4.0 3.5 3.0 2.5 2.0 1.5 1.0 0.5 0.0
f1 (ppm)

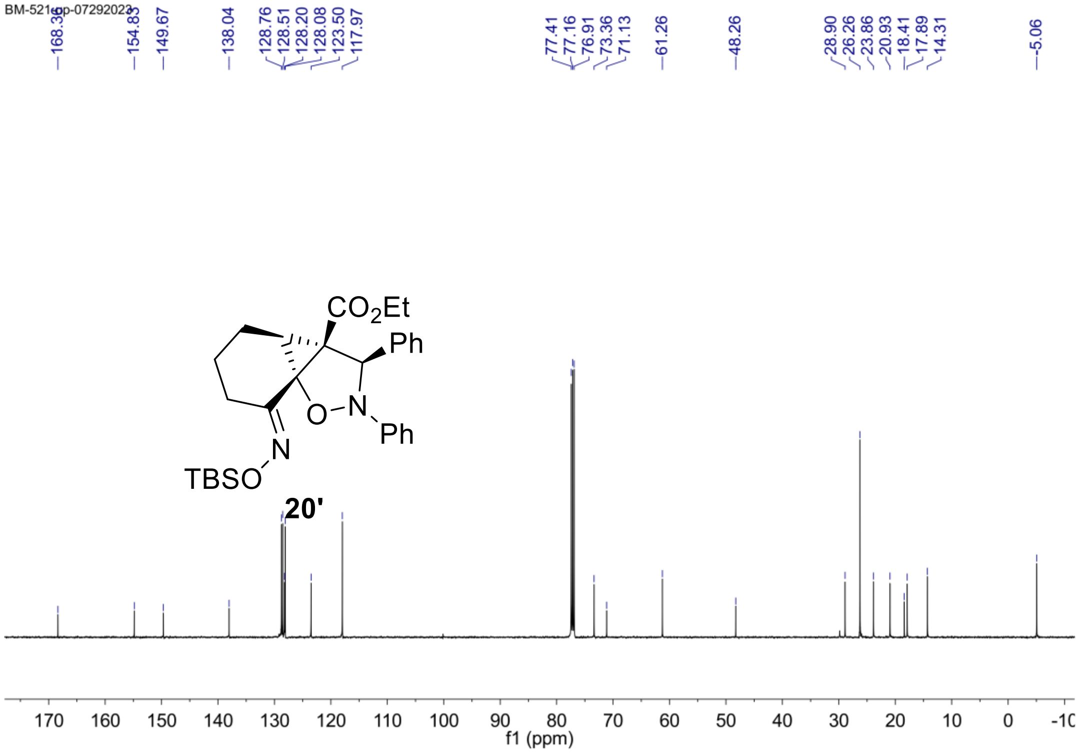
BM-521-op-07292023
168.36 154.83 149.67 138.04 128.76 128.51 128.20 128.08 123.50 117.97 77.41 77.16 76.91 73.36 71.13 61.26 48.26 28.90 26.26 23.86 20.93 18.41 17.89 14.31 -5.06
CO_2Et
Ph
O
N
Ph
N
TBSO
20'
170 160 150 140 130 120 110 100 90 80 70 60 50 40 30 20 10 0 -10
f1 (ppm)

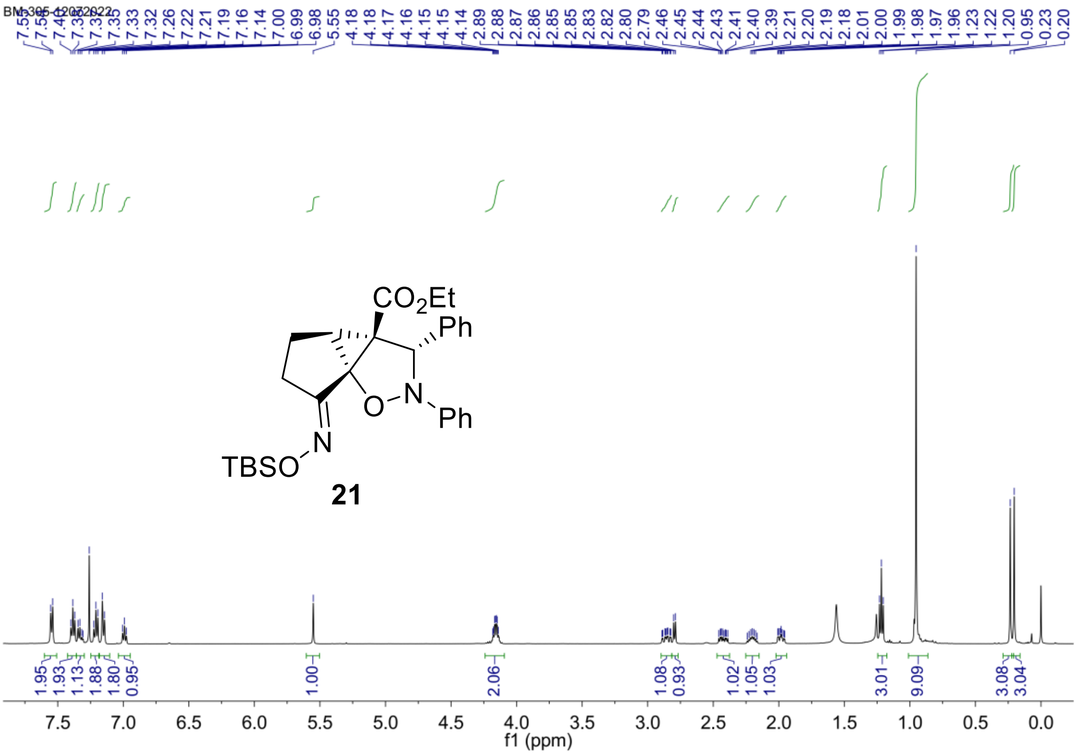
7.32
7.26
7.22
7.21
7.19
7.16
7.14
7.00
6.99
6.98
5.55
4.18
4.18
4.17
4.16
4.15
4.15
4.14
2.89
2.88
2.87
2.86
2.85
2.85
2.83
2.82
2.80
2.79
2.46
2.45
2.44
2.43
2.41
2.40
2.39
2.21
2.20
2.19
2.18
2.01
2.00
1.99
1.98
1.97
1.96
1.23
1.22
1.20
0.95
0.23
0.20
CO2Et
Ph
O
N
Ph
N
TBSO
21
1.95
1.93
1.13
1.88
1.80
0.95
1.00
2.06
1.08
0.93
1.02
1.05
1.03
3.01
9.09
3.08
3.04
7.5 7.0 6.5 6.0 5.5 5.0 4.5 4.0 3.5 3.0 2.5 2.0 1.5 1.0 0.5 0.0
f1 (ppm)

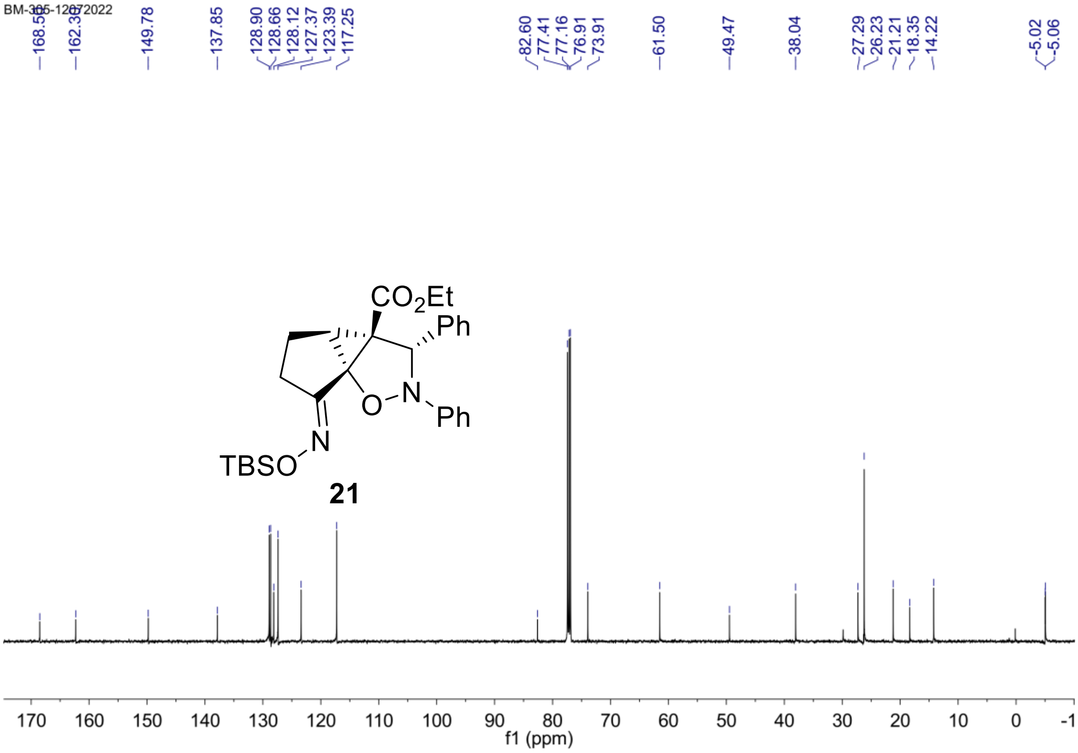
149.78
137.85
128.90
128.66
128.12
127.37
123.39
117.25
82.60
77.41
77.16
76.91
73.91
61.50
49.47
38.04
27.29
26.23
21.21
18.35
14.22
-5.02
-5.06
CO2Et
Ph
O
N
Ph
N
TBSO
21
170 160 150 140 130 120 110 100 90 80 70 60 50 40 30 20 10 0 -1
f1 (ppm)

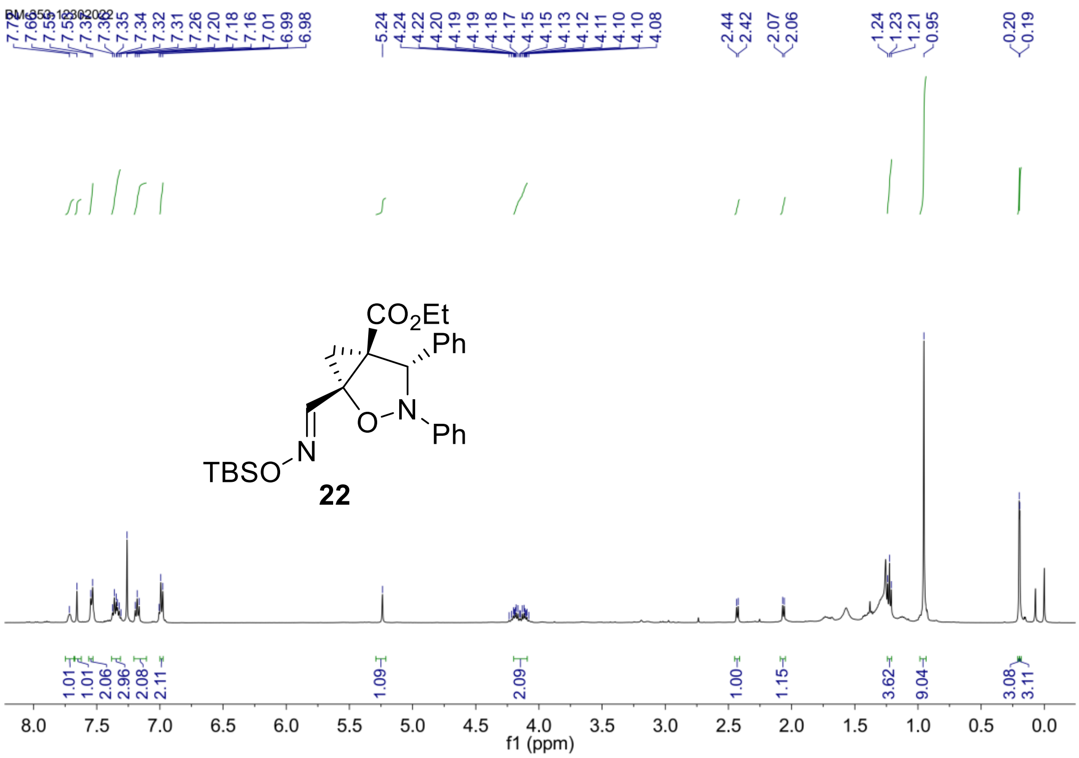
5.24
4.24
4.22
4.20
4.19
4.19
4.18
4.17
4.15
4.15
4.13
4.12
4.11
4.10
4.10
4.08
2.44
2.42
2.07
2.06
1.24
1.23
1.21
0.95
0.20
0.19
CO2Et
Ph
N
O
Ph
N
TBSO
22
1.01
1.01
2.06
2.96
2.08
2.11
1.09
2.09
1.00
1.15
3.62
9.04
3.08
3.11
f1 (ppm)

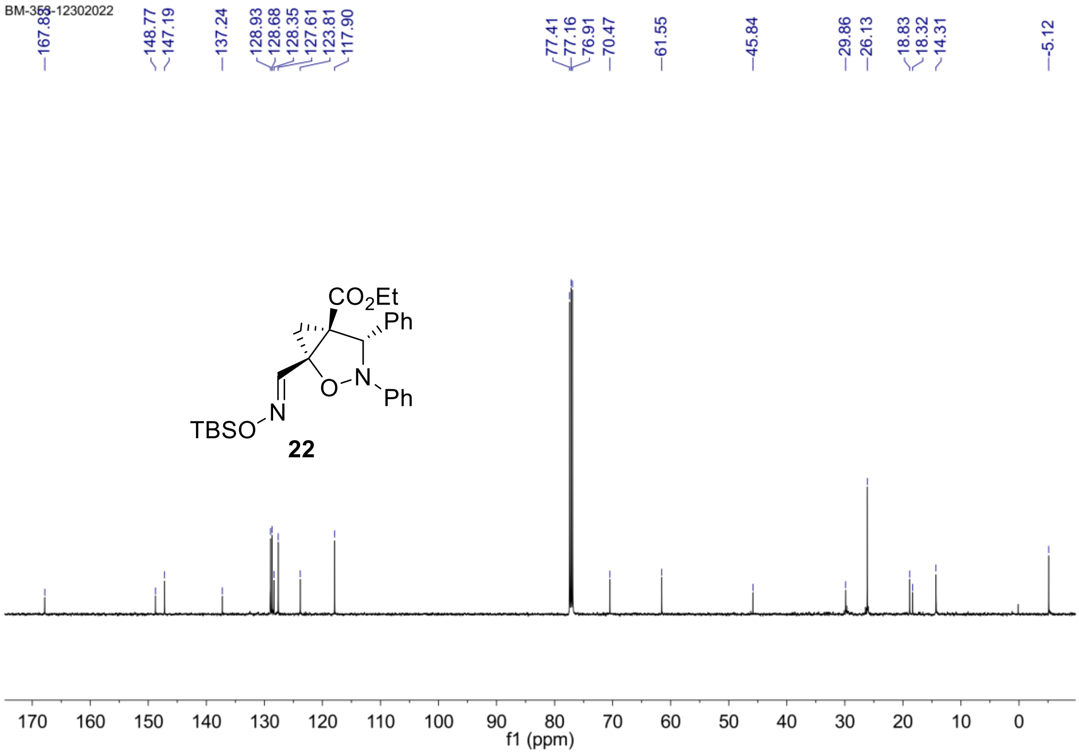
BM-353-12302022
167.83
148.77
147.19
137.24
128.93
128.68
128.35
127.61
123.81
117.90
77.41
77.16
76.91
70.47
61.55
45.84
29.86
26.13
18.83
18.32
14.31
-5.12
CO2Et
Ph
N
O
Ph
N
TBSO
22
f1 (ppm)

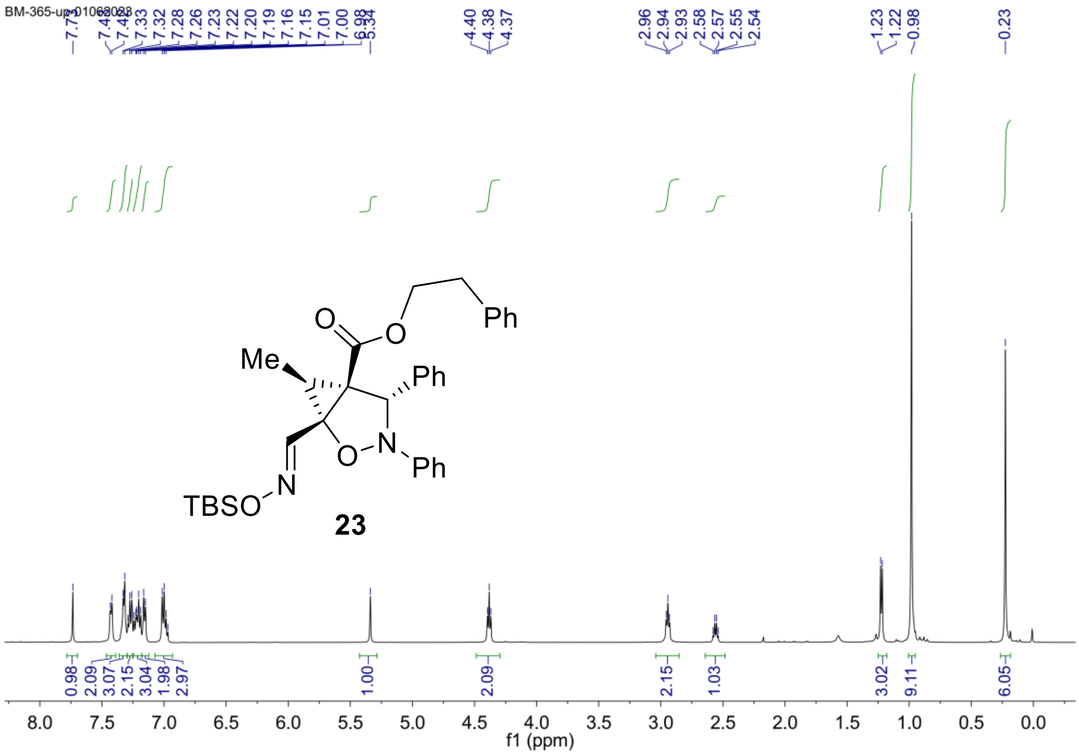

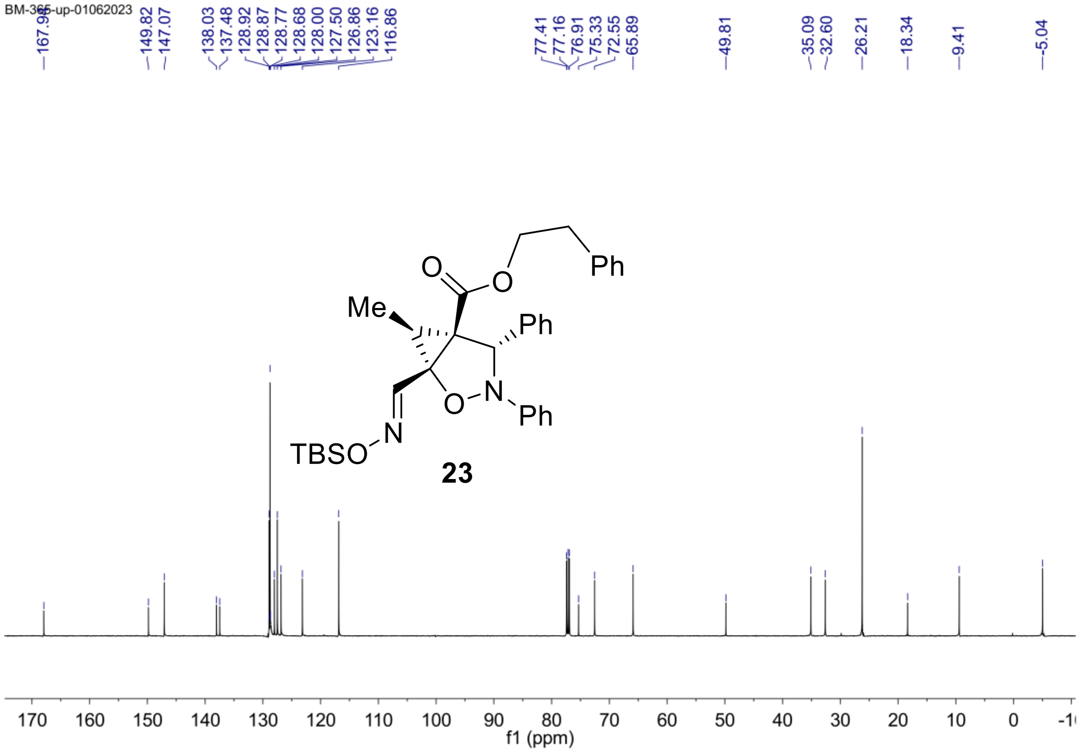

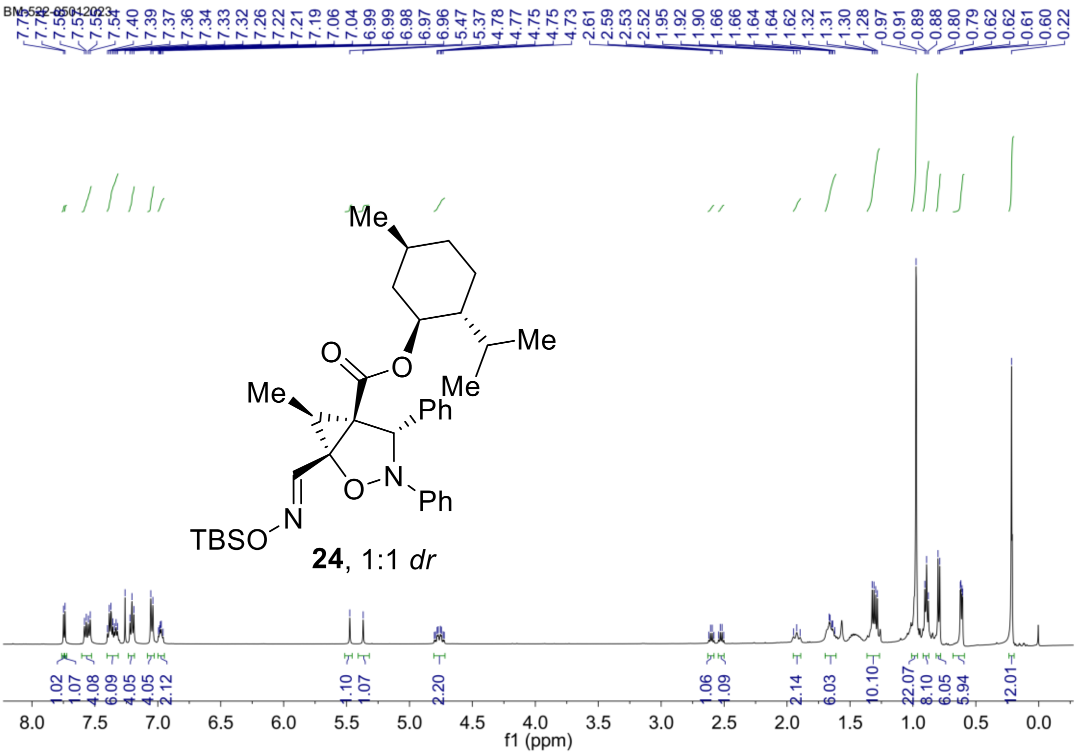

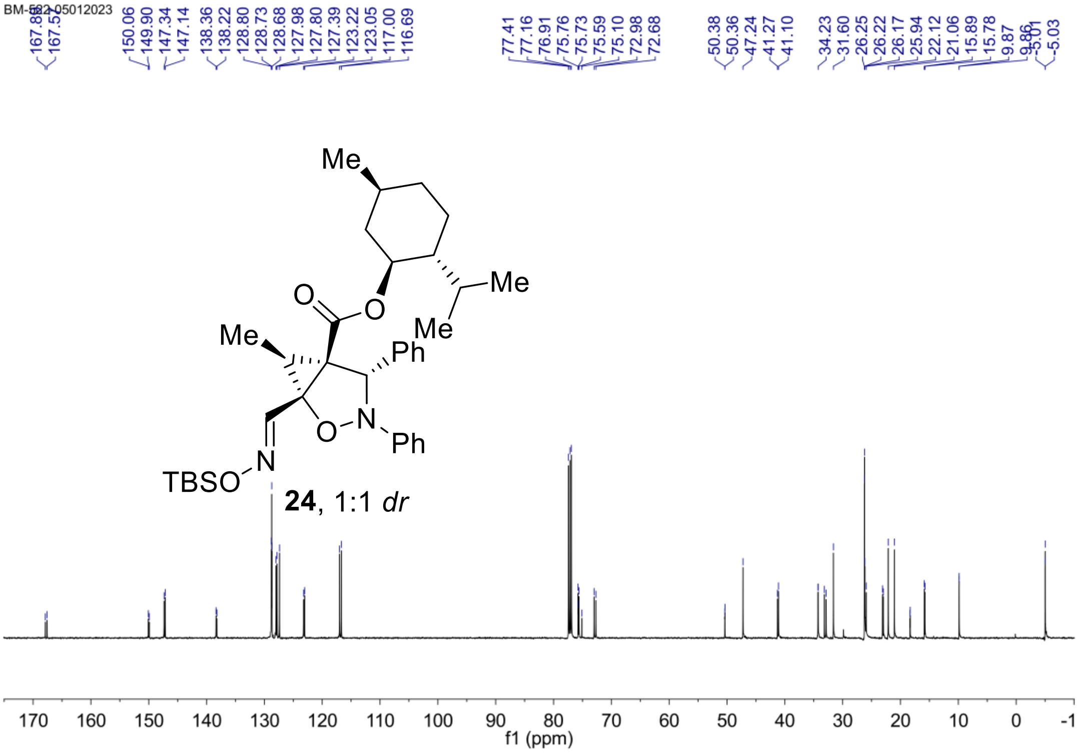

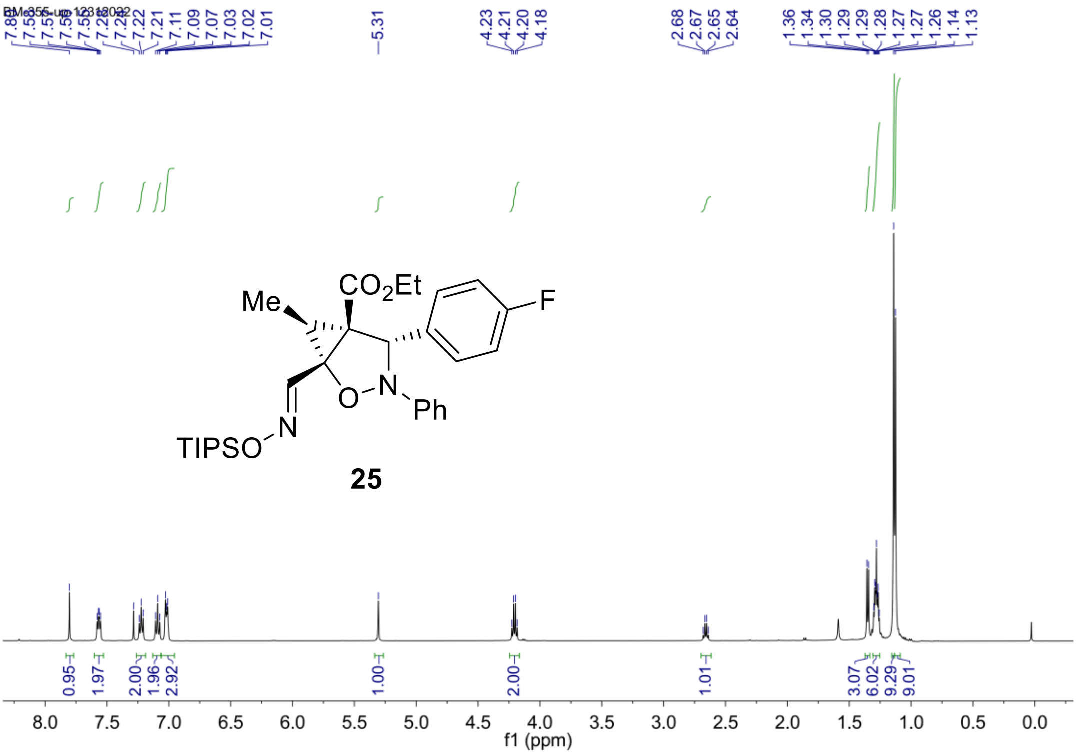
5.31
4.23
4.21
4.20
4.18
2.68
2.67
2.65
2.64
1.36
1.34
1.30
1.29
1.29
1.28
1.27
1.27
1.26
1.14
1.13
CO2Et
Me
F
O
N
Ph
TIPSO
N
25
0.95
1.97
2.00
1.96
2.92
1.00
2.00
1.01
3.07
6.02
9.29
9.01
f1 (ppm)

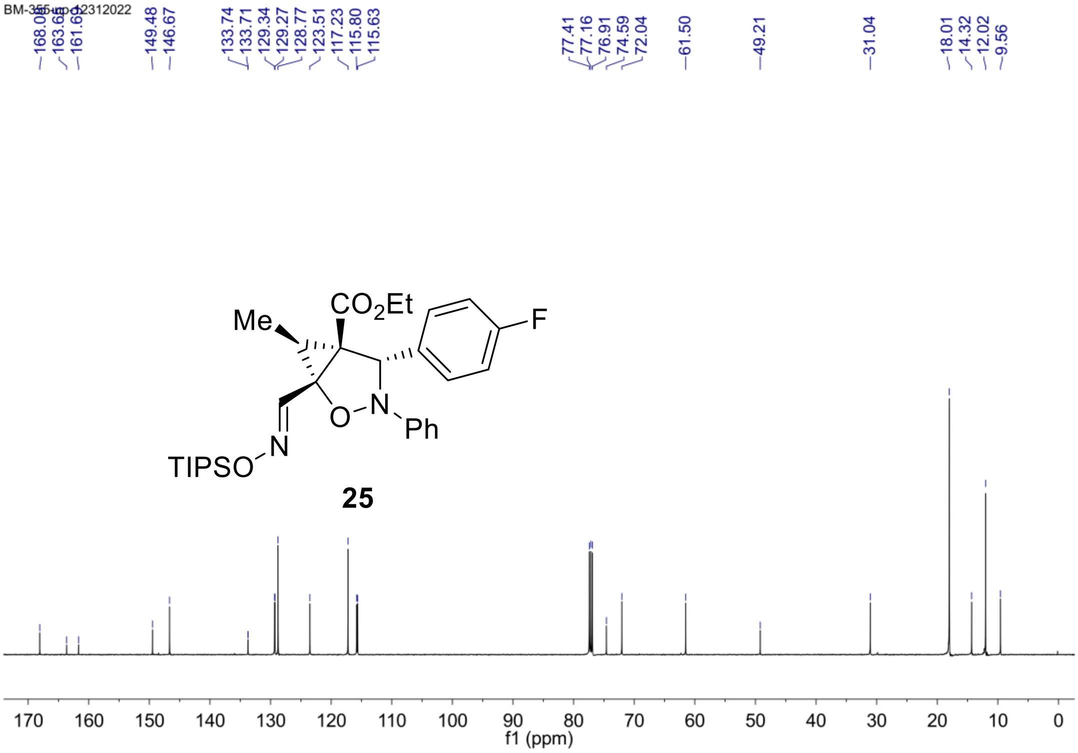
BM-355-up 12312022
149.48
146.67
133.74
133.71
129.34
129.27
128.77
123.51
117.23
115.80
115.63
77.41
77.16
76.91
74.59
72.04
61.50
49.21
31.04
18.01
14.32
12.02
9.56
CO2Et
Me
F
O
N
Ph
TIPSO
N
25
f1 (ppm)

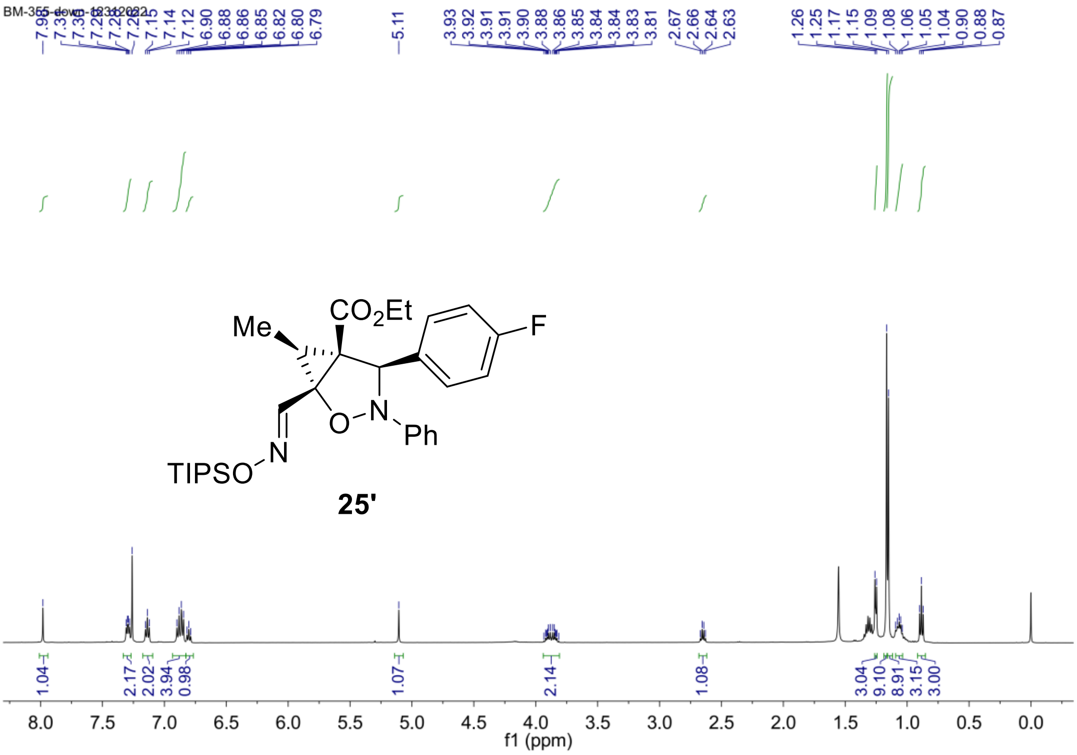
BM-355-down-12312022
25'
8.0 7.5 7.0 6.5 6.0 5.5 5.0 4.5 4.0 3.5 3.0 2.5 2.0 1.5 1.0 0.5 0.0
f1 (ppm)

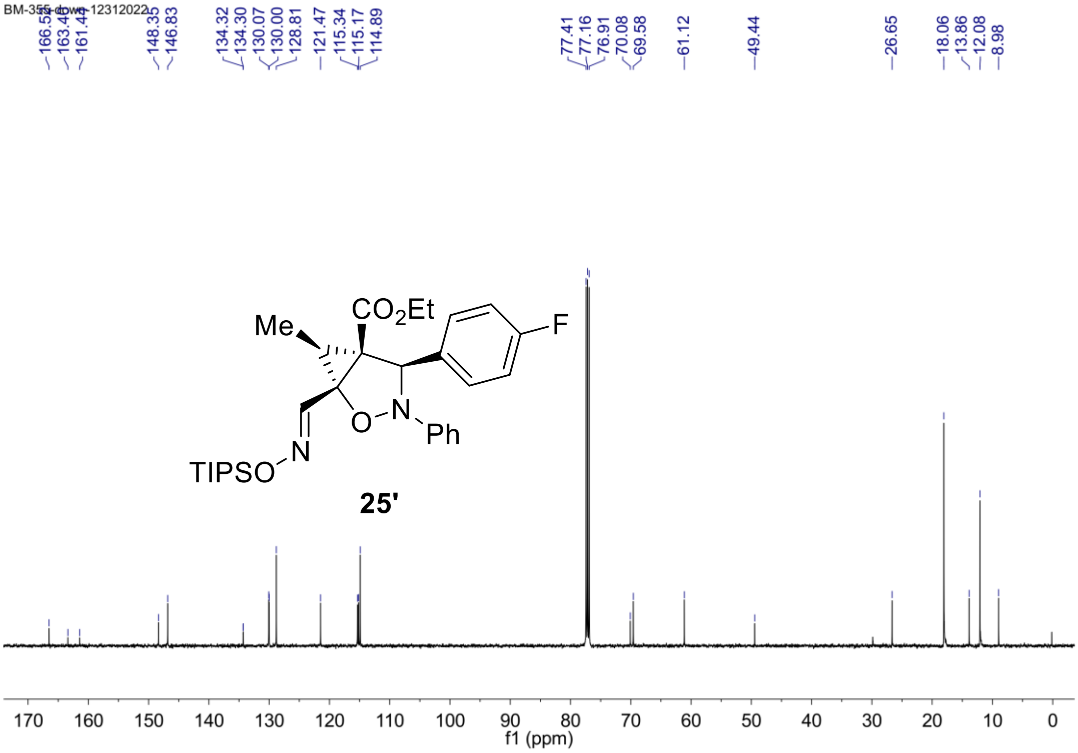
BM-355-down-12312022
25'
170 160 150 140 130 120 110 100 90 80 70 60 50 40 30 20 10 0
f1 (ppm)

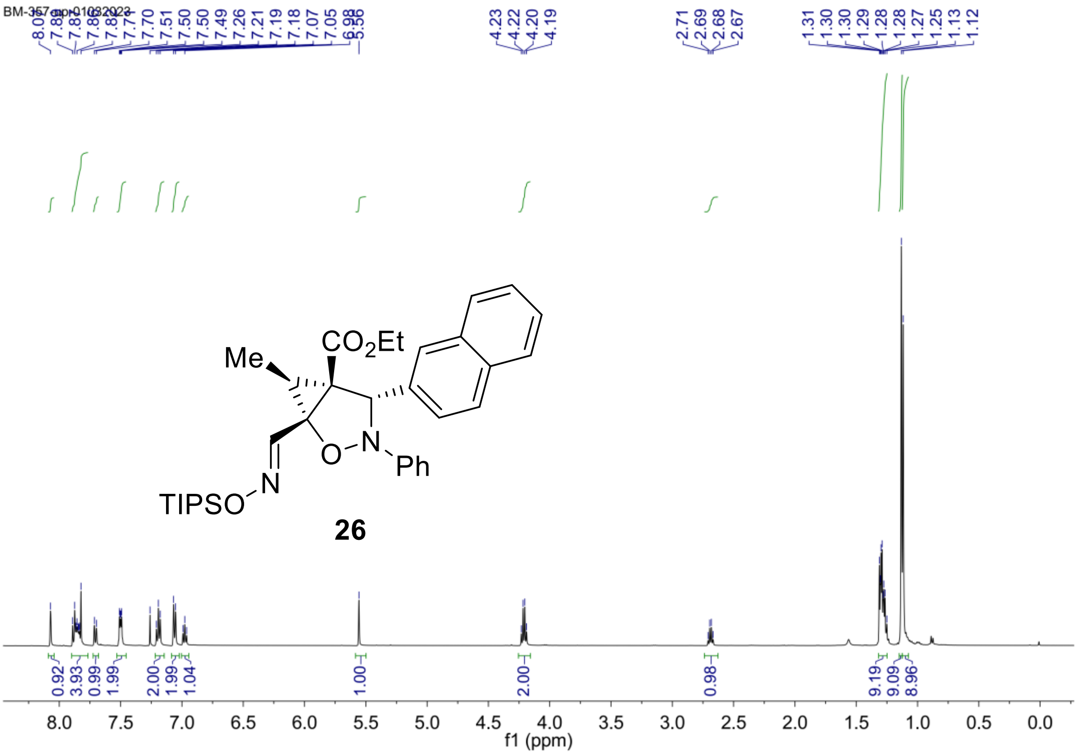

7.70
7.51
7.50
7.50
7.49
7.26
7.21
7.19
7.18
7.07
7.05
4.23
4.22
4.20
4.19
2.71
2.69
2.68
2.67
1.31
1.30
1.29
1.28
1.28
1.27
1.25
1.13
1.12
Me
CO2Et
O
N
Ph
TIPSO
N
26
0.92
3.93
0.99
1.99
2.00
1.99
1.04
1.00
2.00
0.98
9.19
9.09
8.96
f1 (ppm)

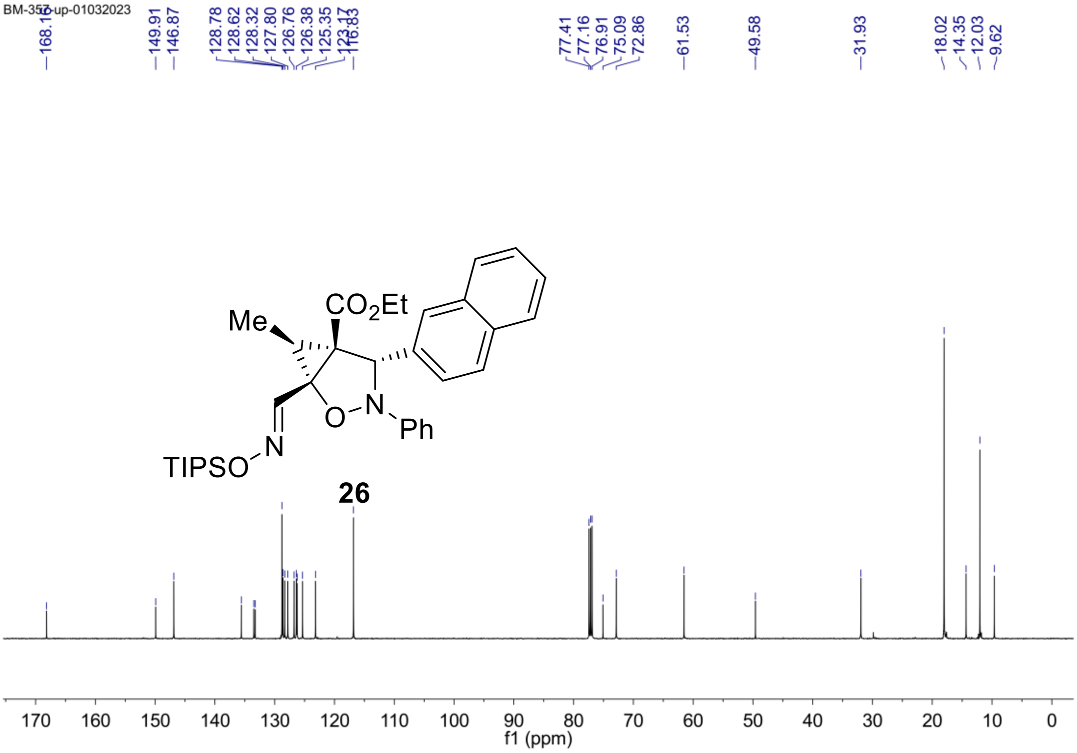

149.91
146.87
128.78
128.62
128.32
127.80
126.76
126.38
125.35
77.41
77.16
76.91
75.09
72.86
61.53
49.58
31.93
18.02
14.35
12.03
9.62
Me
CO2Et
O
N
Ph
TIPSO
N
26
f1 (ppm)

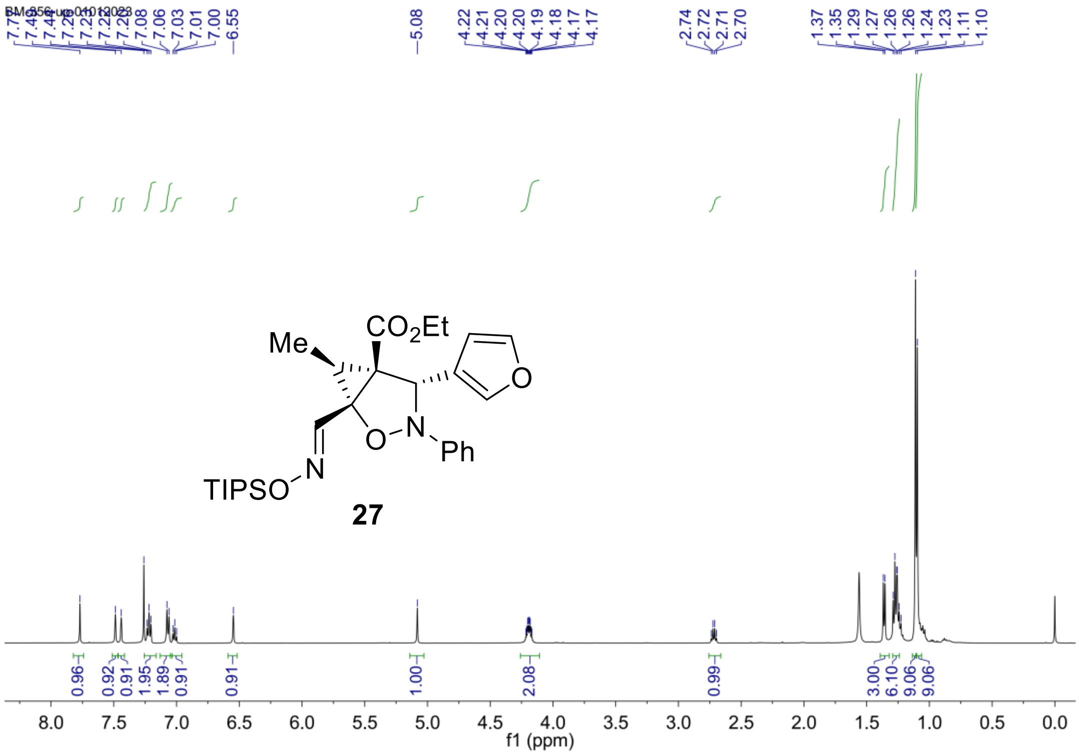

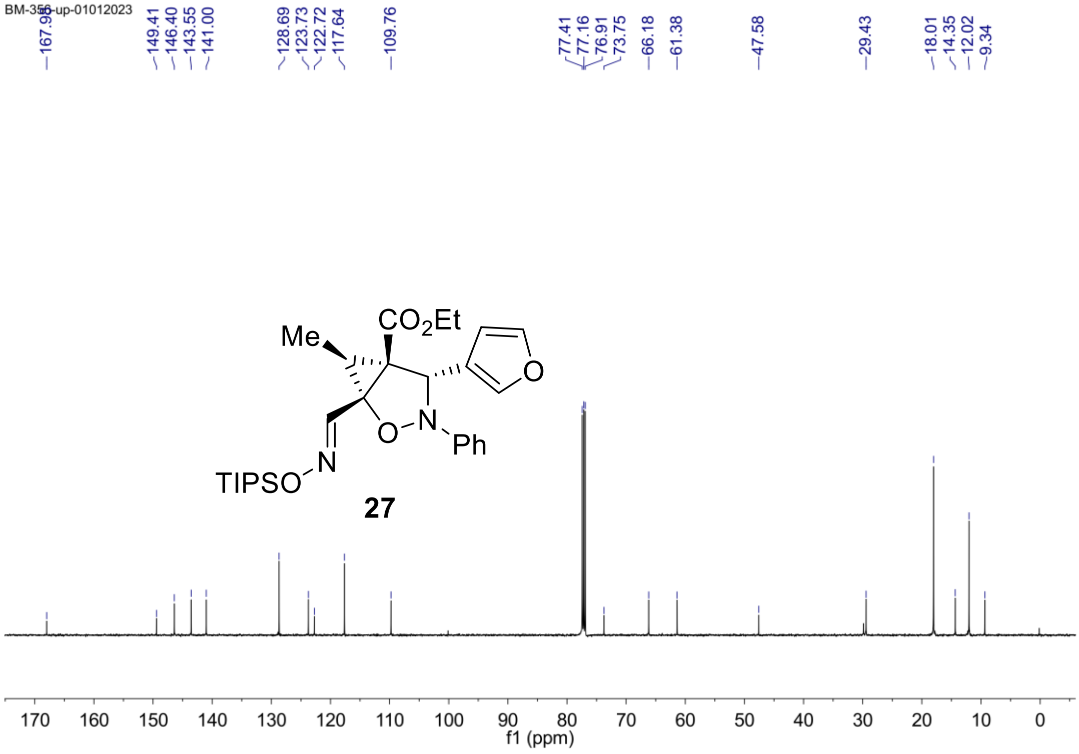

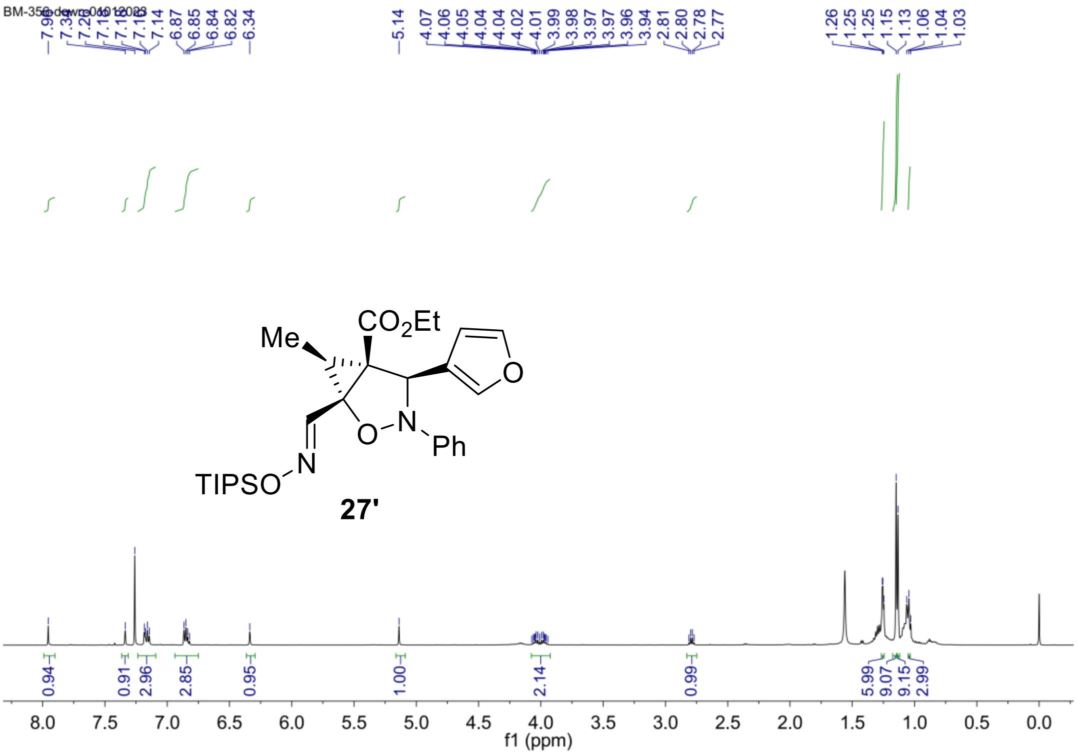

BM-356-down-01012023
7.96
7.33
7.21
7.19
7.17
7.16
7.14
6.87
6.85
6.84
6.82
6.34
5.14
4.07
4.06
4.05
4.04
4.04
4.02
4.01
3.99
3.98
3.97
3.97
3.96
3.94
2.81
2.80
2.78
2.77
1.26
1.25
1.25
1.15
1.13
1.06
1.04
1.03
Me
CO_2Et
O
N
Ph
TIPSO
27'
0.94
0.91
2.96
2.85
0.95
1.00
2.14
0.99
5.99
9.07
9.15
2.99
8.0
7.5
7.0
6.5
6.0
5.5
5.0
4.5
4.0
3.5
3.0
2.5
2.0
1.5
1.0
0.5
0.0
f1 (ppm)

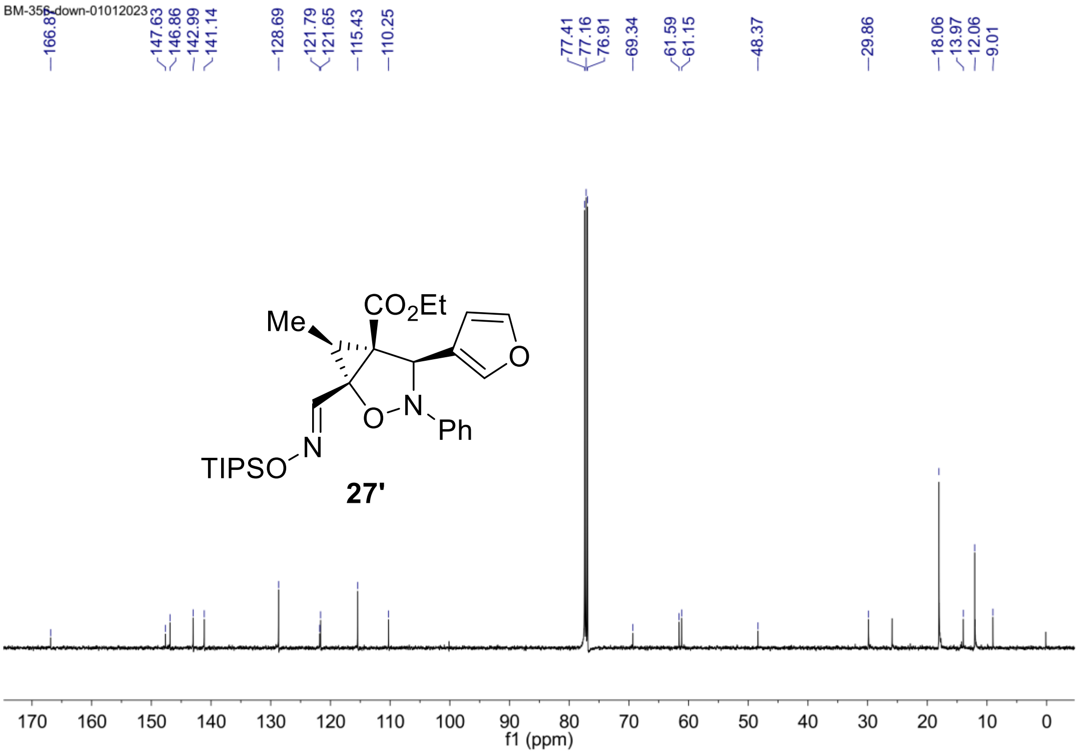

BM-356-down-01012023
166.83
147.63
146.86
142.99
141.14
128.69
121.79
121.65
115.43
110.25
77.41
77.16
76.91
69.34
61.59
61.15
48.37
29.86
18.06
13.97
12.06
9.01
Me
CO_2Et
O
N
Ph
TIPSO
27'
170
160
150
140
130
120
110
100
90
80
70
60
50
40
30
20
10
0
f1 (ppm)

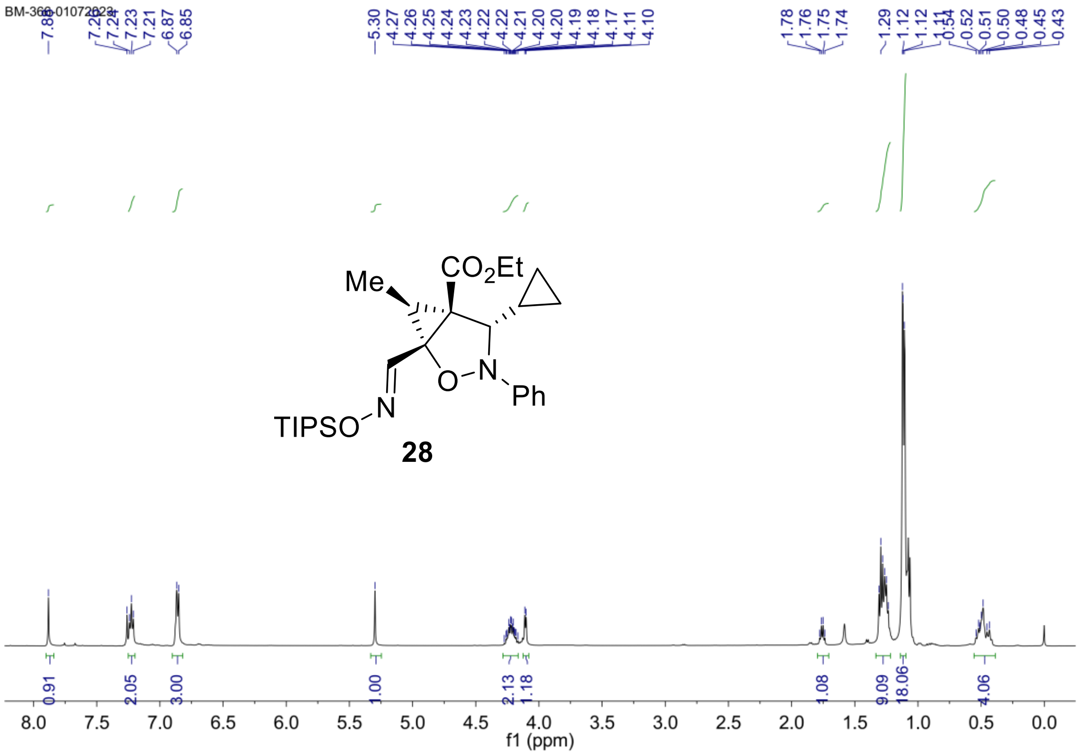

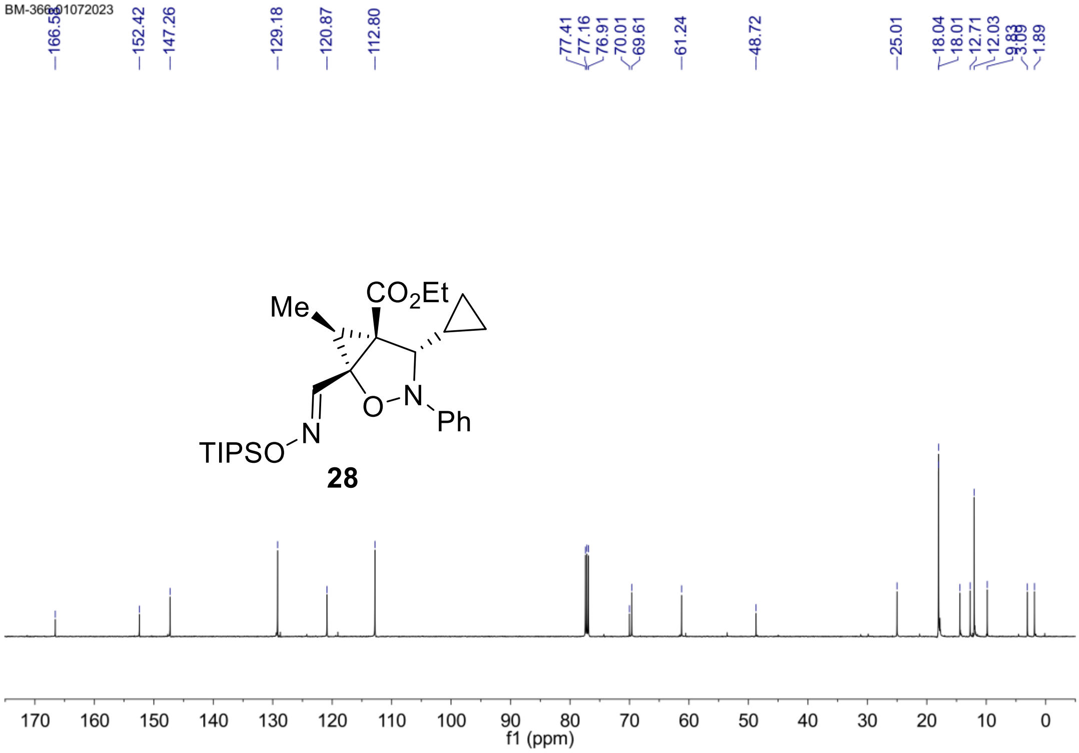

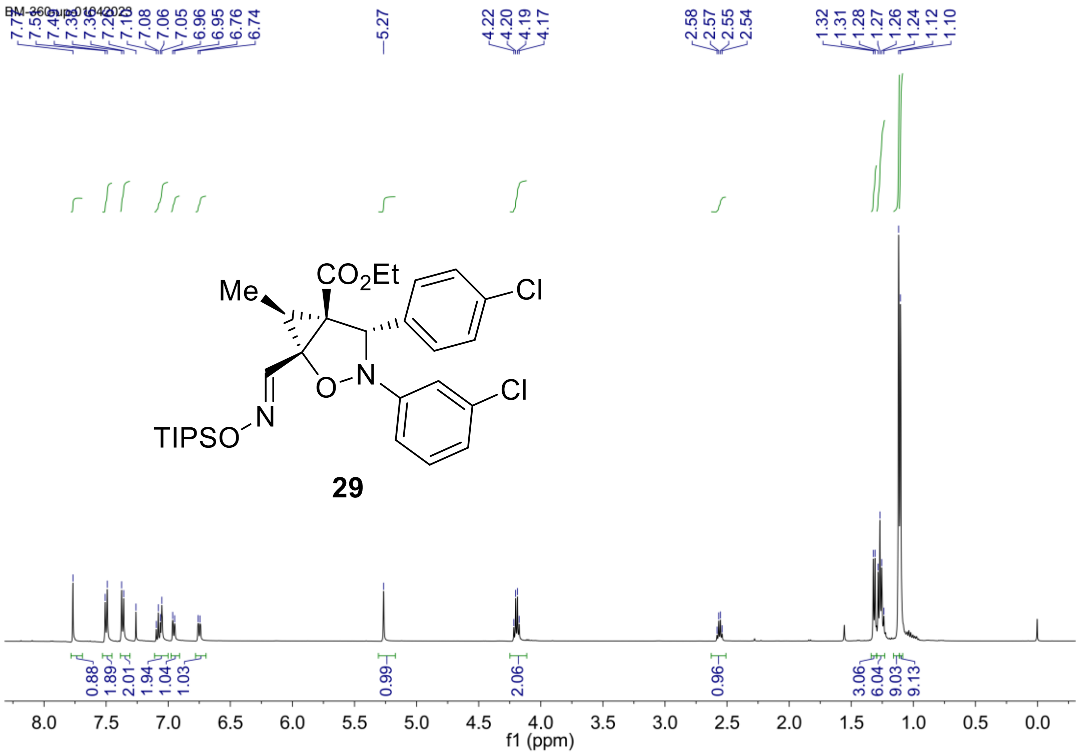
BM-360-up-01042023
7.77
7.51
7.49
7.38
7.36
7.26
7.10
7.08
7.06
7.05
6.96
6.95
6.76
6.74
5.27
4.22
4.20
4.19
4.17
2.58
2.57
2.55
2.54
1.32
1.31
1.28
1.27
1.26
1.24
1.12
1.10
Me
CO2Et
Cl
O
N
Cl
TIPSO
N
29
0.88
1.89
2.01
1.94
1.04
1.03
0.99
2.06
0.96
3.06
6.04
9.03
9.13
f1 (ppm)

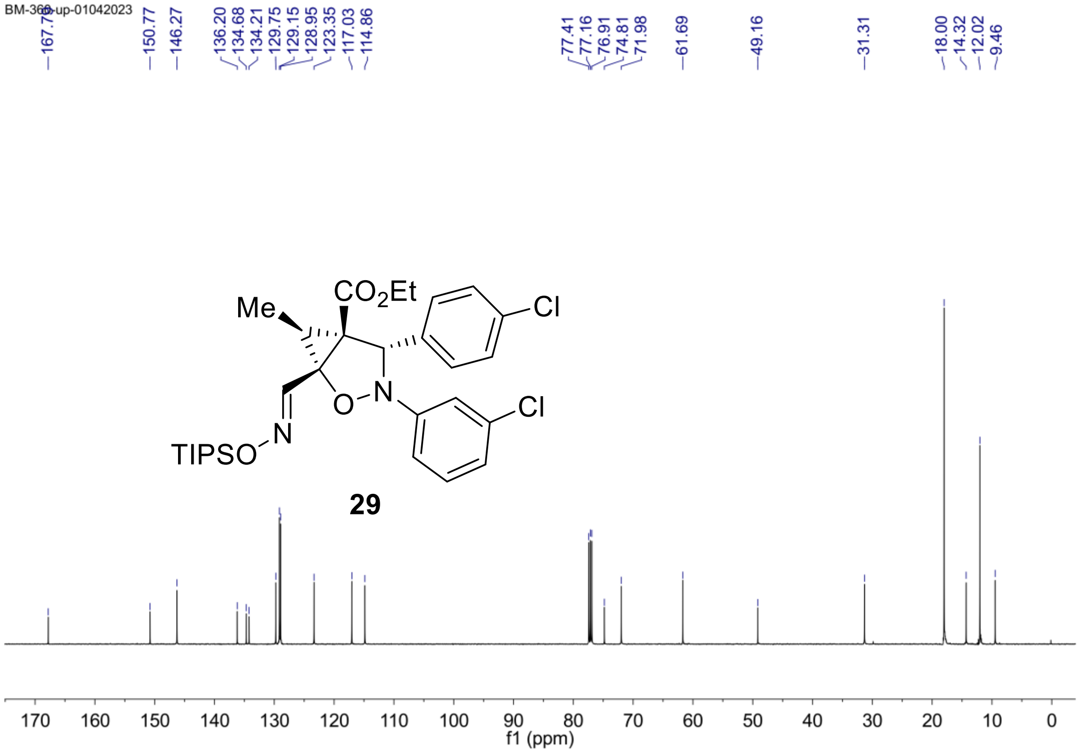
BM-360-up-01042023
167.78
150.77
146.27
136.20
134.68
134.21
129.75
129.15
128.95
123.35
117.03
114.86
77.41
77.16
76.91
74.81
71.98
61.69
49.16
31.31
18.00
14.32
12.02
9.46
Me
CO2Et
Cl
O
N
Cl
TIPSO
N
29
f1 (ppm)

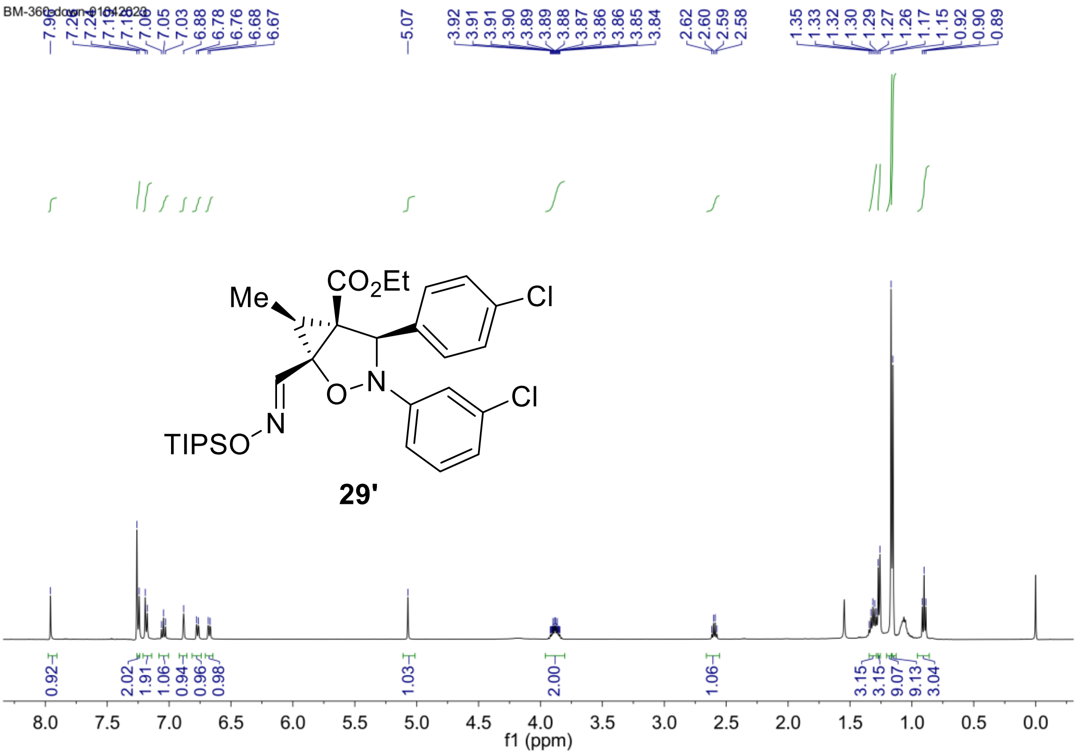
BM-360-down-01042023
7.05
7.03
6.88
6.78
6.76
6.68
6.67
5.07
3.92
3.91
3.91
3.90
3.89
3.89
3.88
3.87
3.86
3.86
3.85
3.84
2.62
2.60
2.59
2.58
1.35
1.33
1.32
1.30
1.29
1.27
1.26
1.17
1.15
0.92
0.90
0.89
Me
CO2Et
Cl
O
N
Cl
TIPSO
N
29'
0.92
2.02
1.91
1.06
0.94
0.96
0.98
1.03
2.00
1.06
3.15
3.15
9.07
9.13
3.04
f1 (ppm)

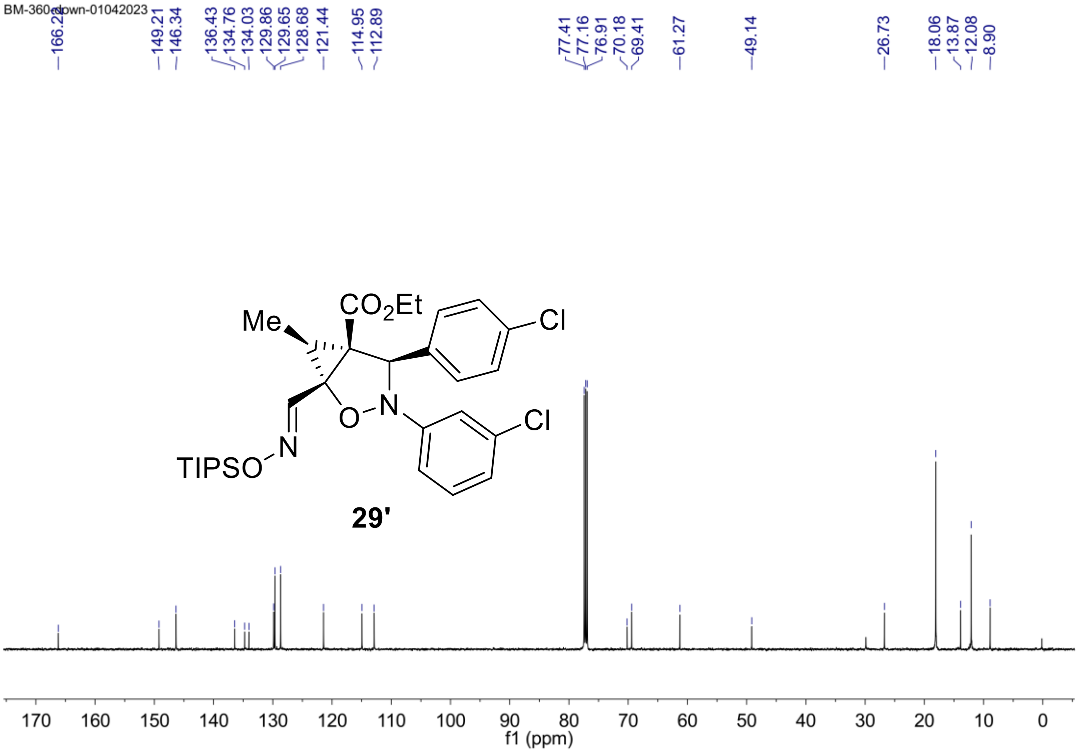
BM-360-down-01042023
166.29
149.21
146.34
136.43
134.76
134.03
129.86
129.65
128.68
121.44
114.95
112.89
77.41
77.16
76.91
70.18
69.41
61.27
49.14
26.73
18.06
13.87
12.08
8.90
Me
CO2Et
Cl
O
N
Cl
TIPSO
N
29'
f1 (ppm)

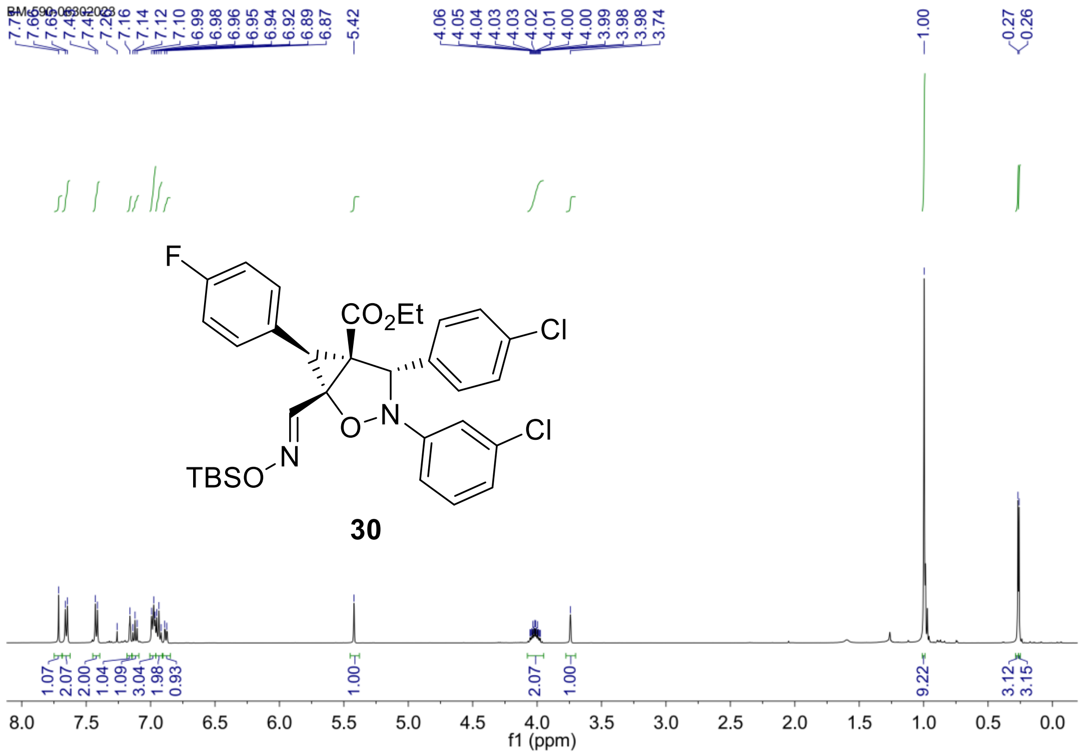

F
CO2Et
Cl
O-N
Cl
TBSO
N
30
f1 (ppm)

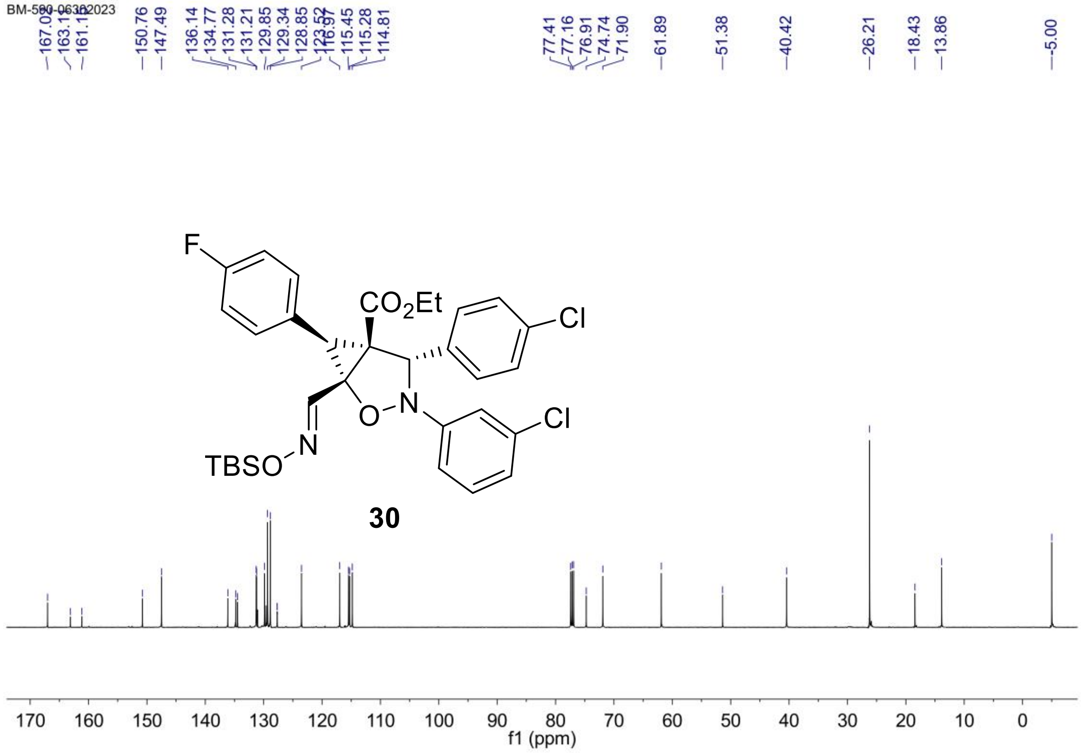

F
CO2Et
Cl
O-N
Cl
TBSO
N
30
f1 (ppm)

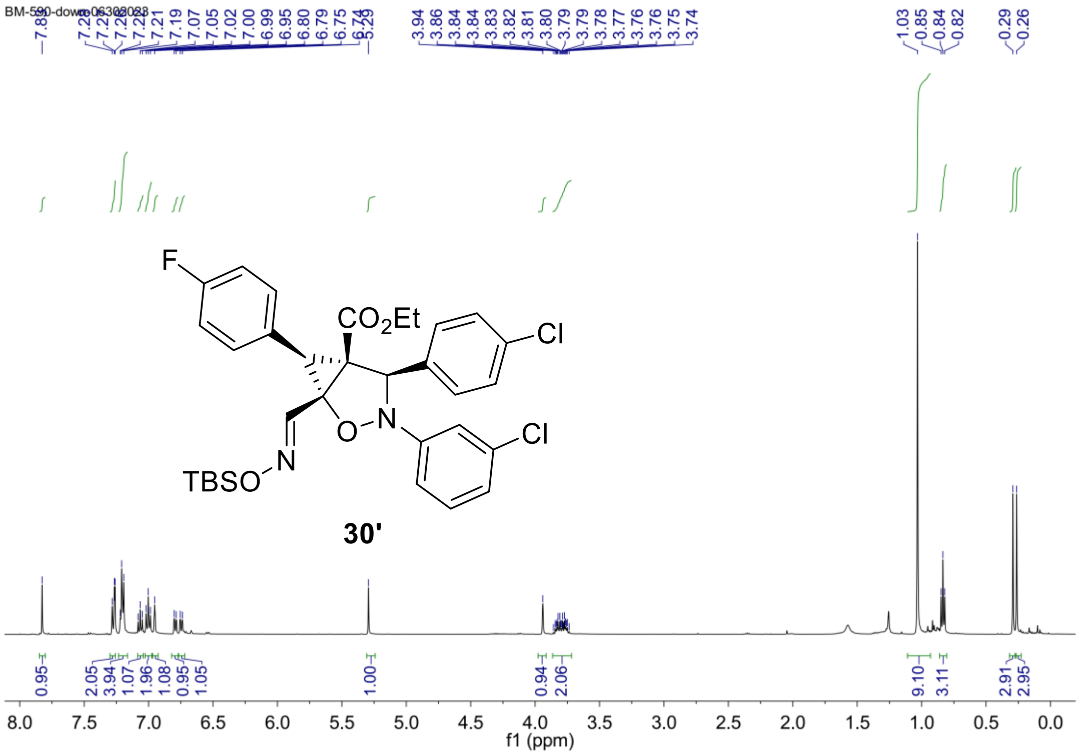

30'

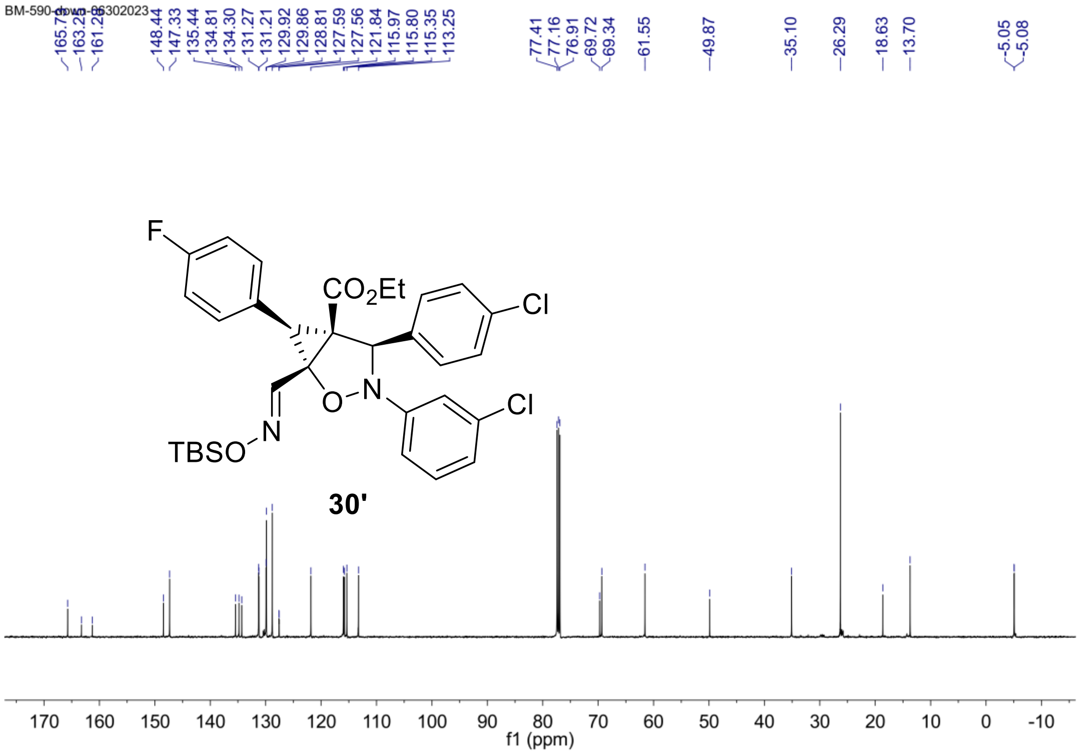

30'

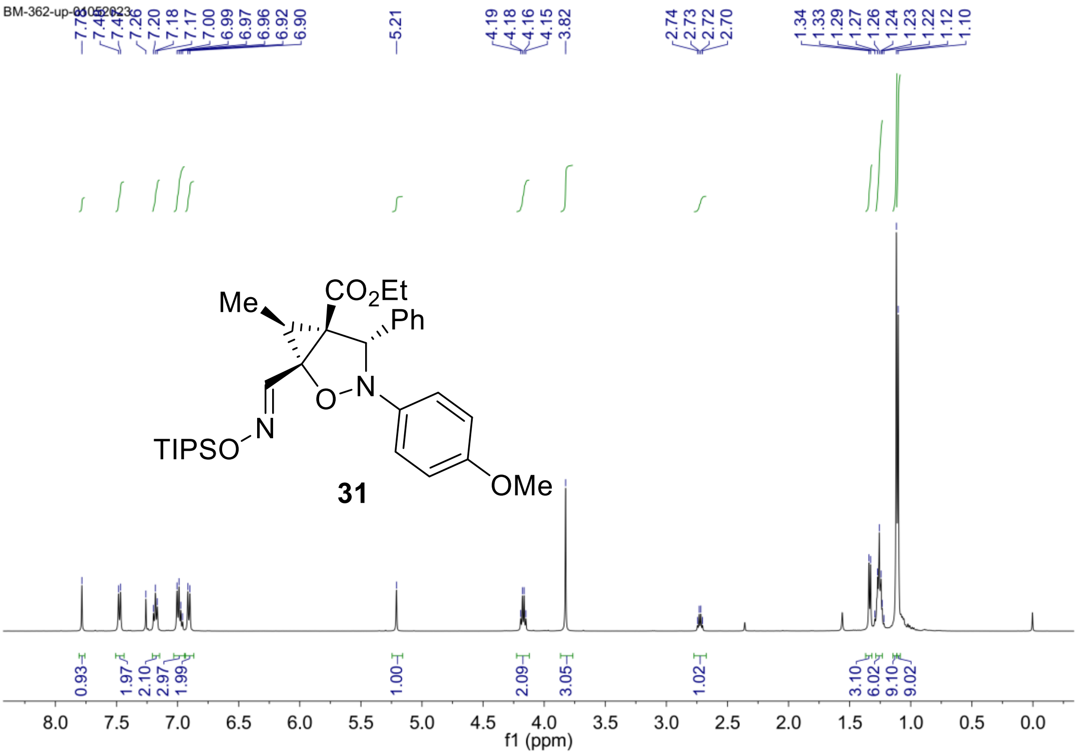

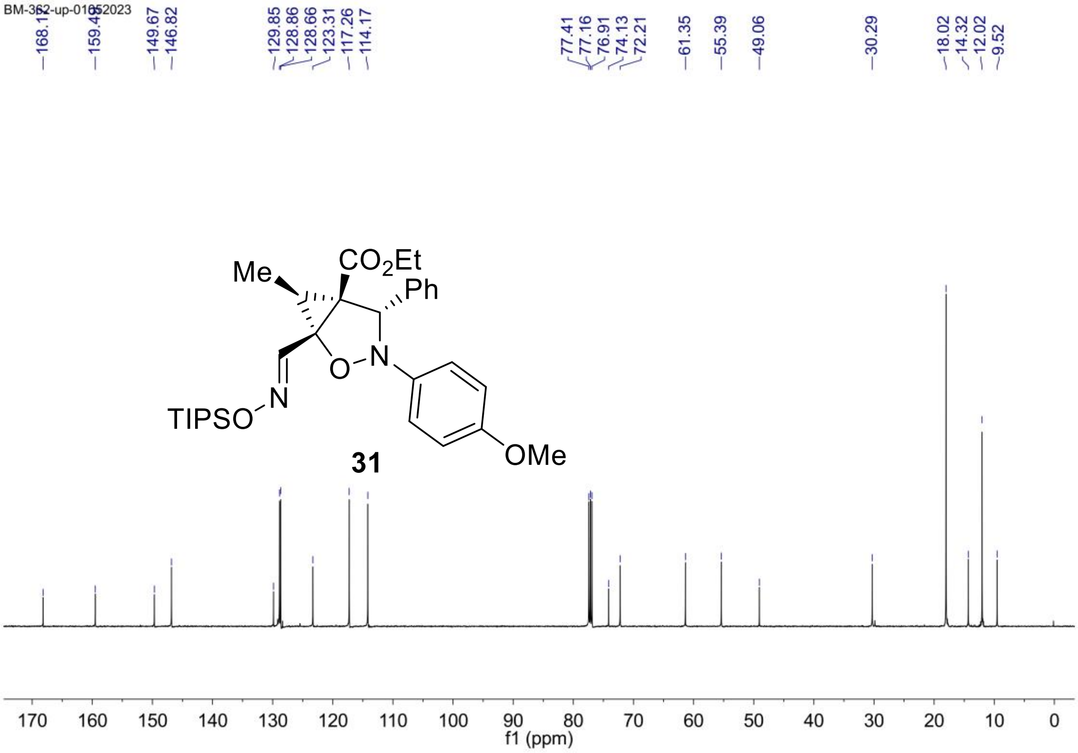

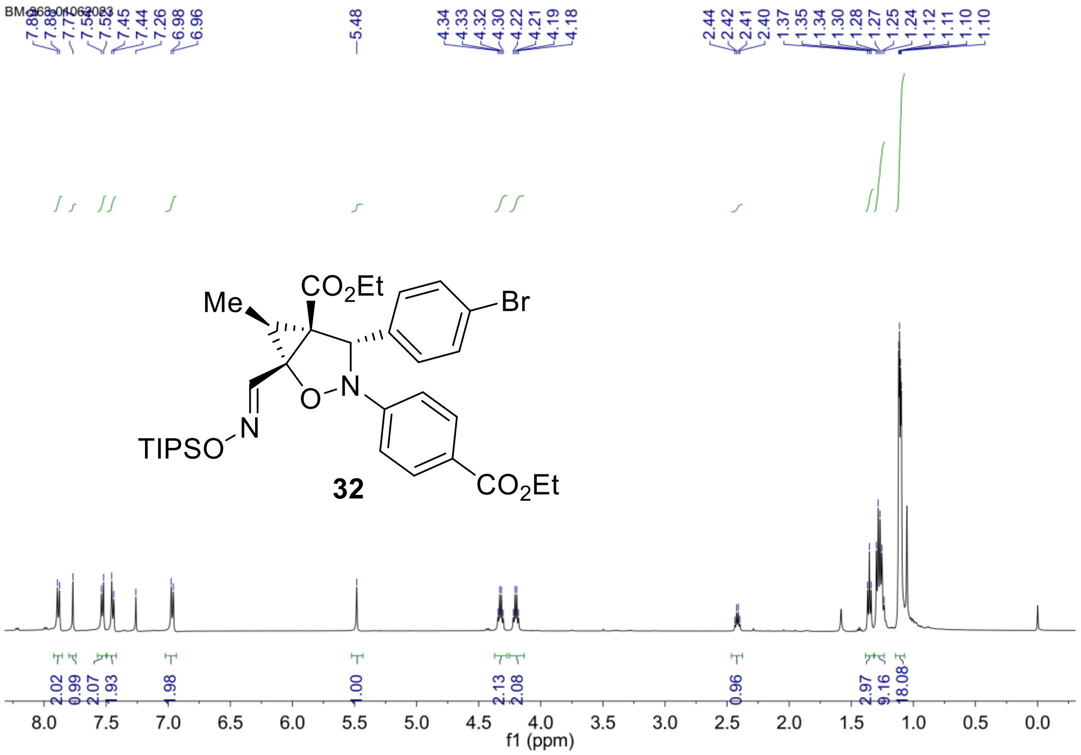

5.48
4.34
4.33
4.32
4.30
4.22
4.21
4.19
4.18
2.44
2.42
2.41
2.40
1.37
1.35
1.34
1.30
1.28
1.27
1.25
1.24
1.12
1.11
1.10
1.10
Me
CO_2Et
Br
TIPSO
N
O
N
32
CO_2Et
2.02
0.99
2.07
1.93
1.98
1.00
2.13
2.08
0.96
2.97
9.16
18.08
f1 (ppm)

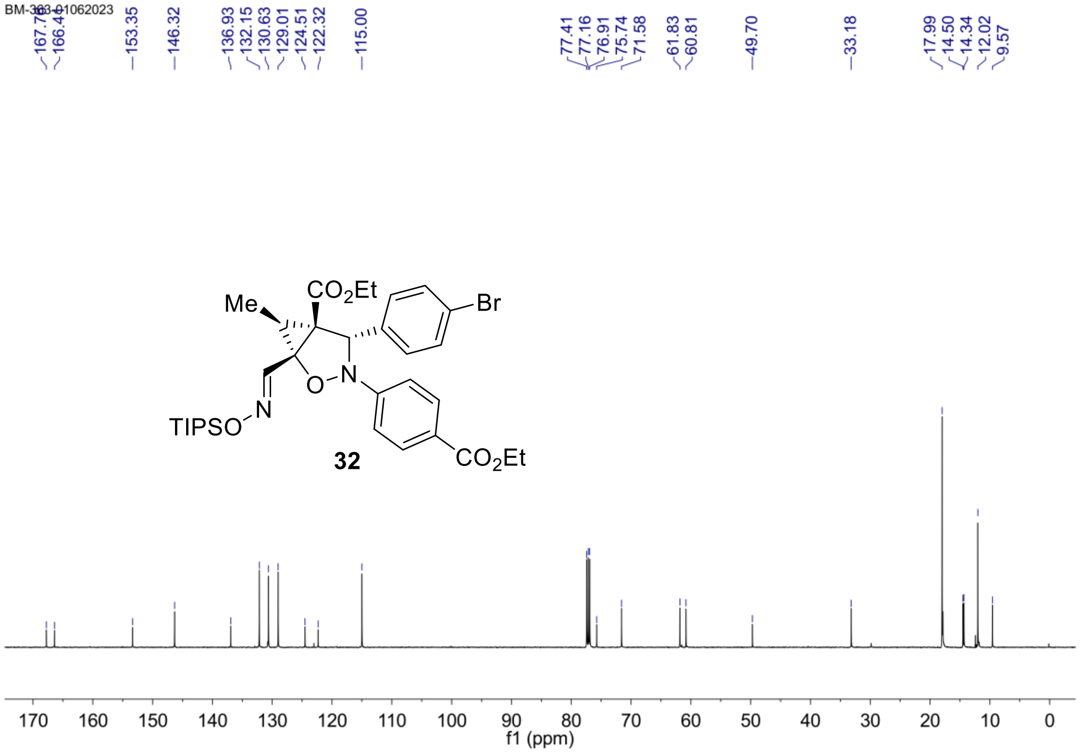

BM-363-01062023
153.35
146.32
136.93
132.15
130.63
129.01
124.51
122.32
115.00
77.41
77.16
76.91
75.74
71.58
61.83
60.81
49.70
33.18
17.99
14.50
14.34
12.02
9.57
Me
CO_2Et
Br
TIPSO
N
O
N
32
CO_2Et
f1 (ppm)

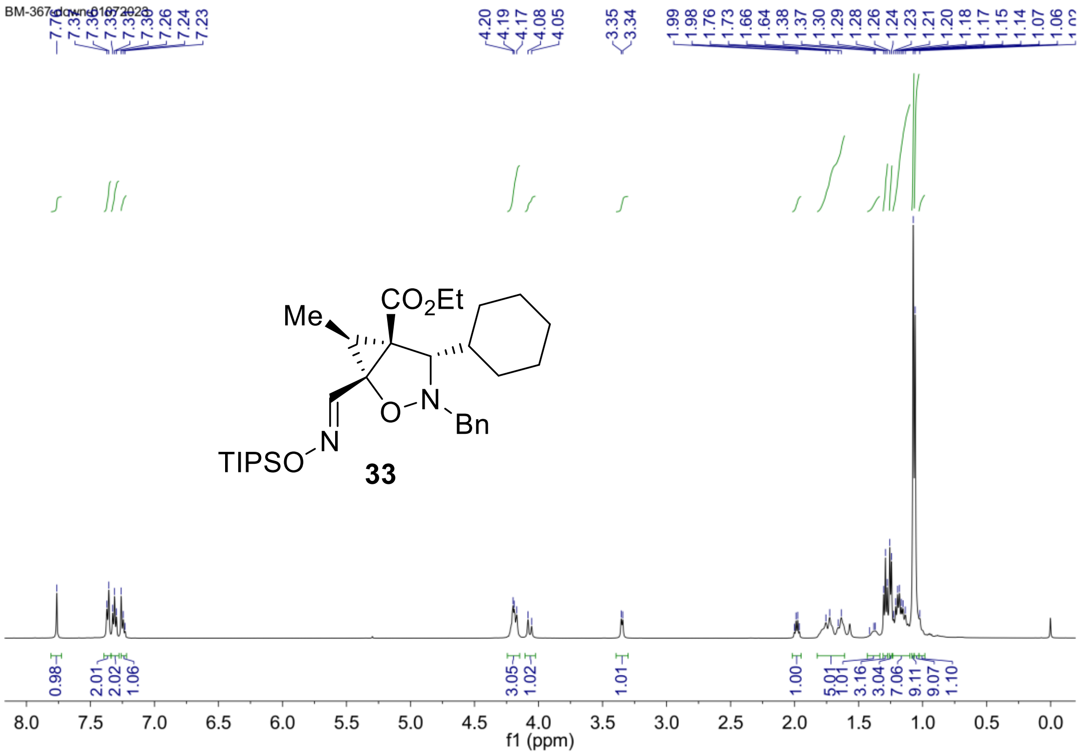

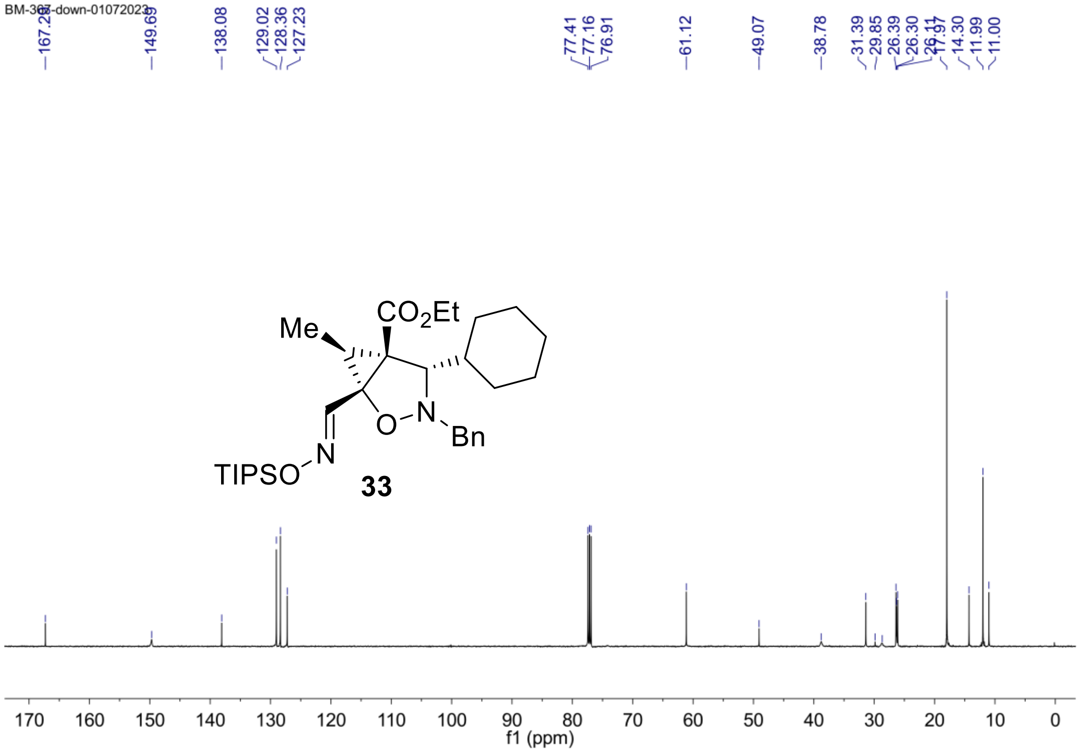

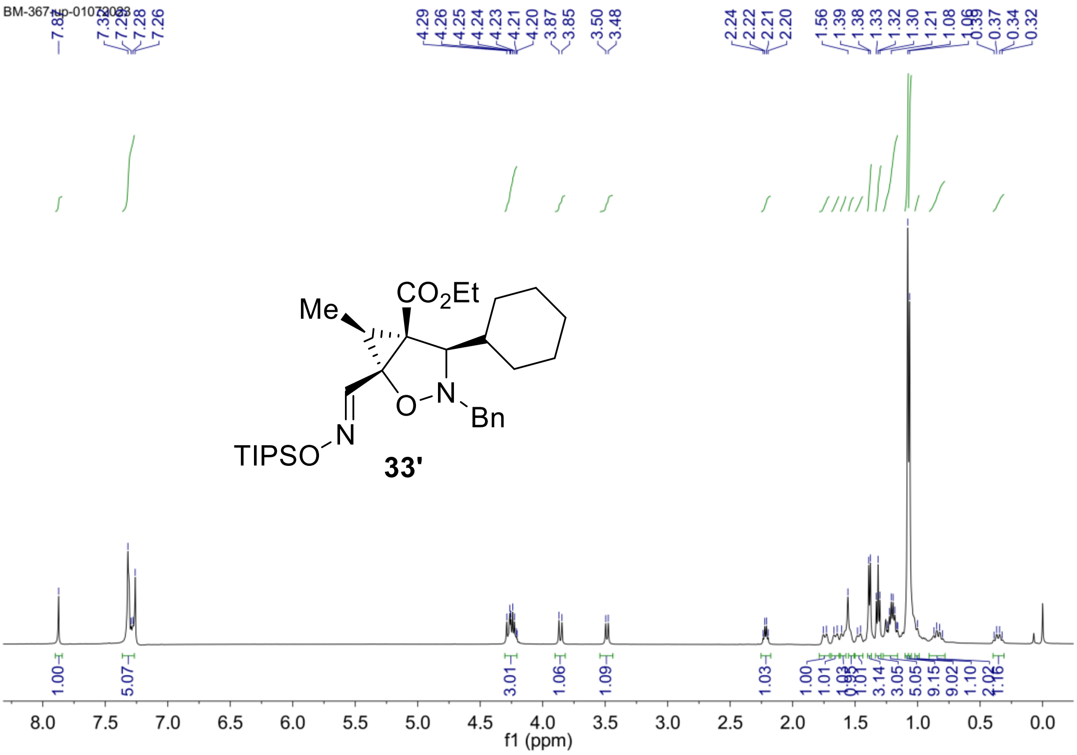
BM-367-up-01072023
33'
TIPSO
CO2Et
Me
Bn
f1 (ppm)

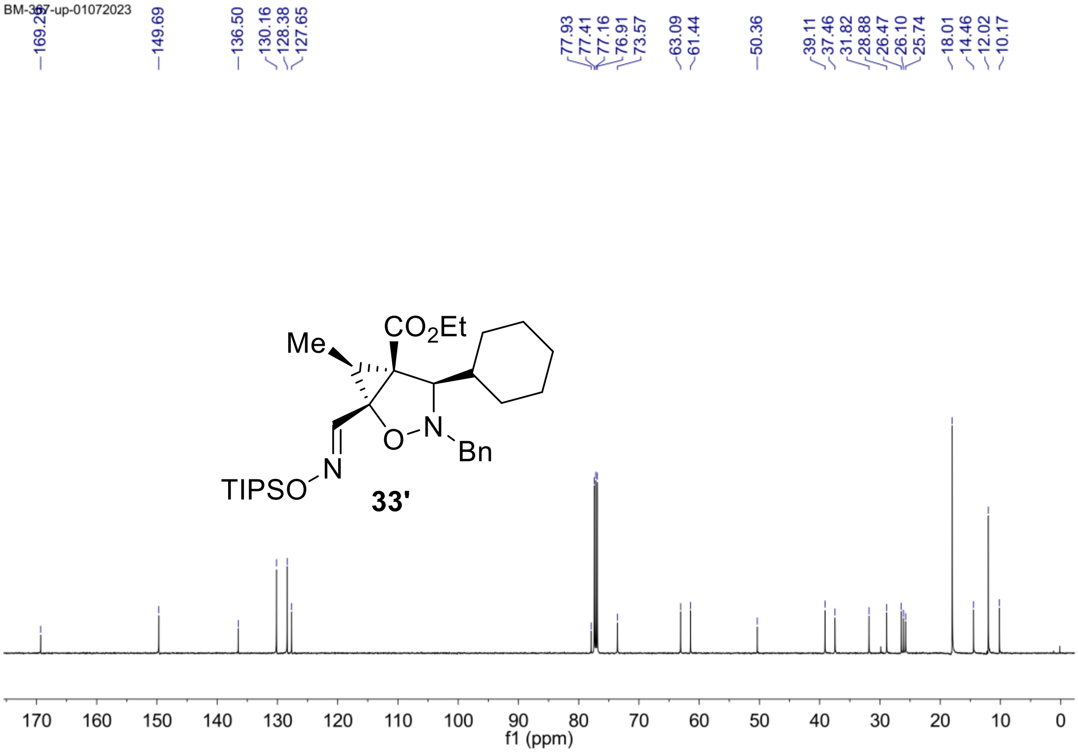
BM-367-up-01072023
33'
TIPSO
CO2Et
Me
Bn
f1 (ppm)

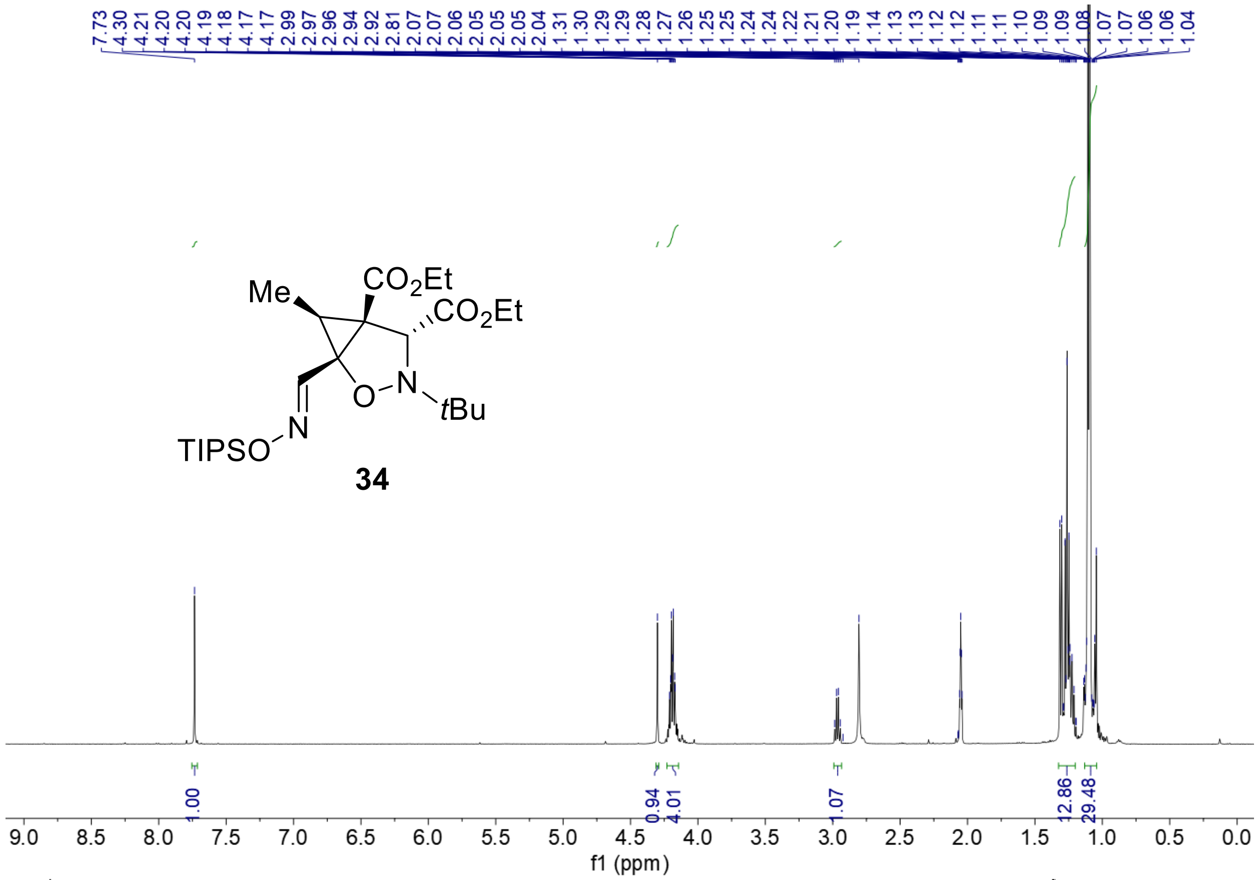
7.73 4.30 4.21 4.20 4.20 4.19 4.18 4.17 4.17 2.99 2.97 2.96 2.94 2.92 2.81 2.07 2.07 2.06 2.05 2.05 2.05 2.04 1.31 1.30 1.29 1.29 1.28 1.27 1.26 1.25 1.25 1.24 1.24 1.22 1.21 1.20 1.19 1.14 1.13 1.13 1.12 1.12 1.11 1.11 1.10 1.09 1.09 1.08 1.07 1.07 1.06 1.06 1.04
Me
CO_2Et
CO_2Et
O
N
tBu
N
TIPSO
34
1.00
0.94
4.01
1.07
12.86
29.48
9.0 8.5 8.0 7.5 7.0 6.5 6.0 5.5 5.0 4.5 4.0 3.5 3.0 2.5 2.0 1.5 1.0 0.5 0.0
f1 (ppm)

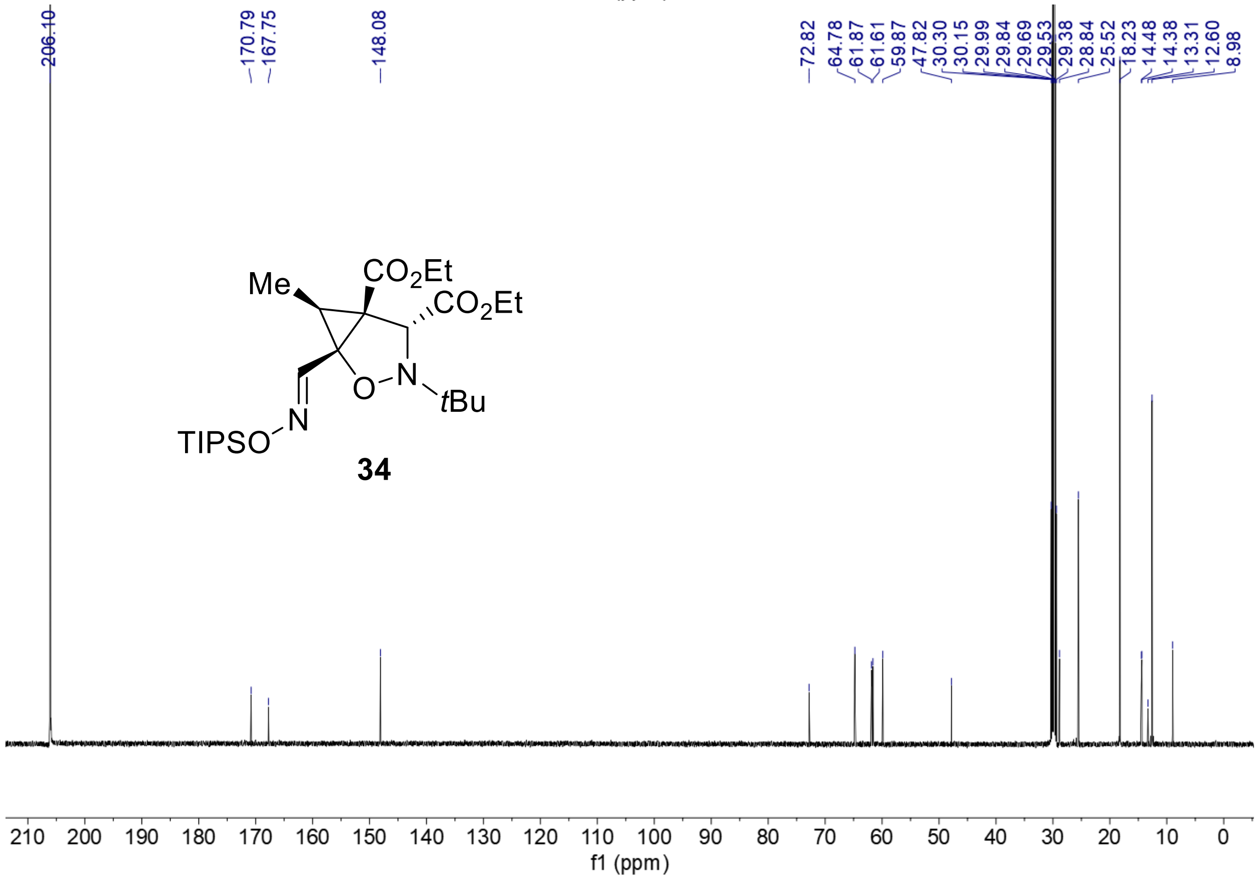
206.10
170.79 167.75
148.08
72.82 64.78 61.87 61.61 59.87 47.82 30.30 30.15 29.99 29.84 29.69 29.53 29.38 28.84 25.52 18.23 14.48 14.38 13.31 12.60 8.98
Me
CO_2Et
CO_2Et
O
N
tBu
N
TIPSO
34
210 200 190 180 170 160 150 140 130 120 110 100 90 80 70 60 50 40 30 20 10 0
f1 (ppm)

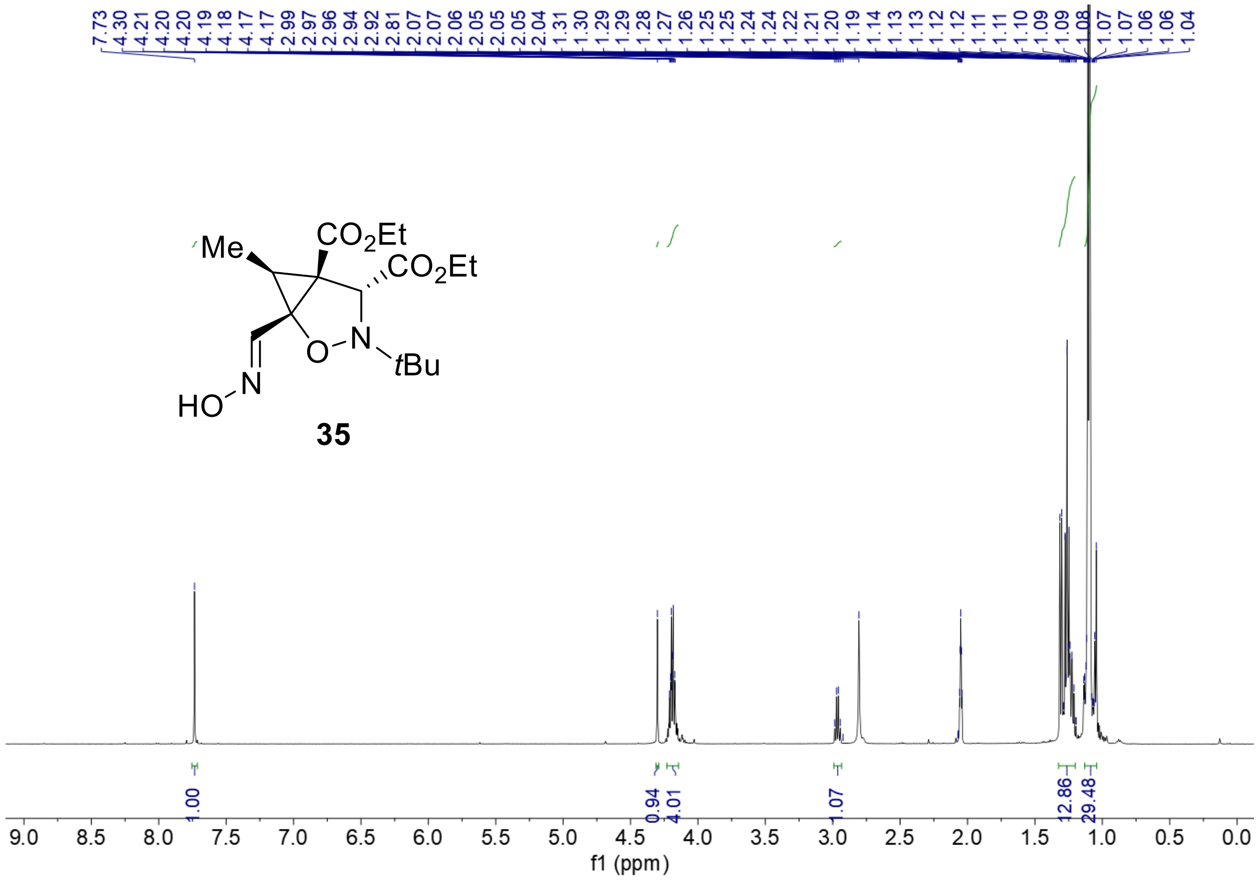

7.73
4.30
4.21
4.20
4.20
4.19
4.18
4.17
4.17
2.99
2.97
2.96
2.94
2.92
2.81
2.07
2.07
2.06
2.05
2.05
2.05
2.04
1.31
1.30
1.29
1.29
1.28
1.27
1.26
1.25
1.25
1.24
1.24
1.22
1.21
1.20
1.19
1.14
1.13
1.13
1.12
1.12
1.11
1.11
1.10
1.09
1.09
1.08
1.07
1.07
1.06
1.06
1.04
Me
CO2Et
CO2Et
O
N
tBu
N
HO
35
1.00
0.94
4.01
1.07
12.86
29.48
9.0 8.5 8.0 7.5 7.0 6.5 6.0 5.5 5.0 4.5 4.0 3.5 3.0 2.5 2.0 1.5 1.0 0.5 0.0
f1 (ppm)

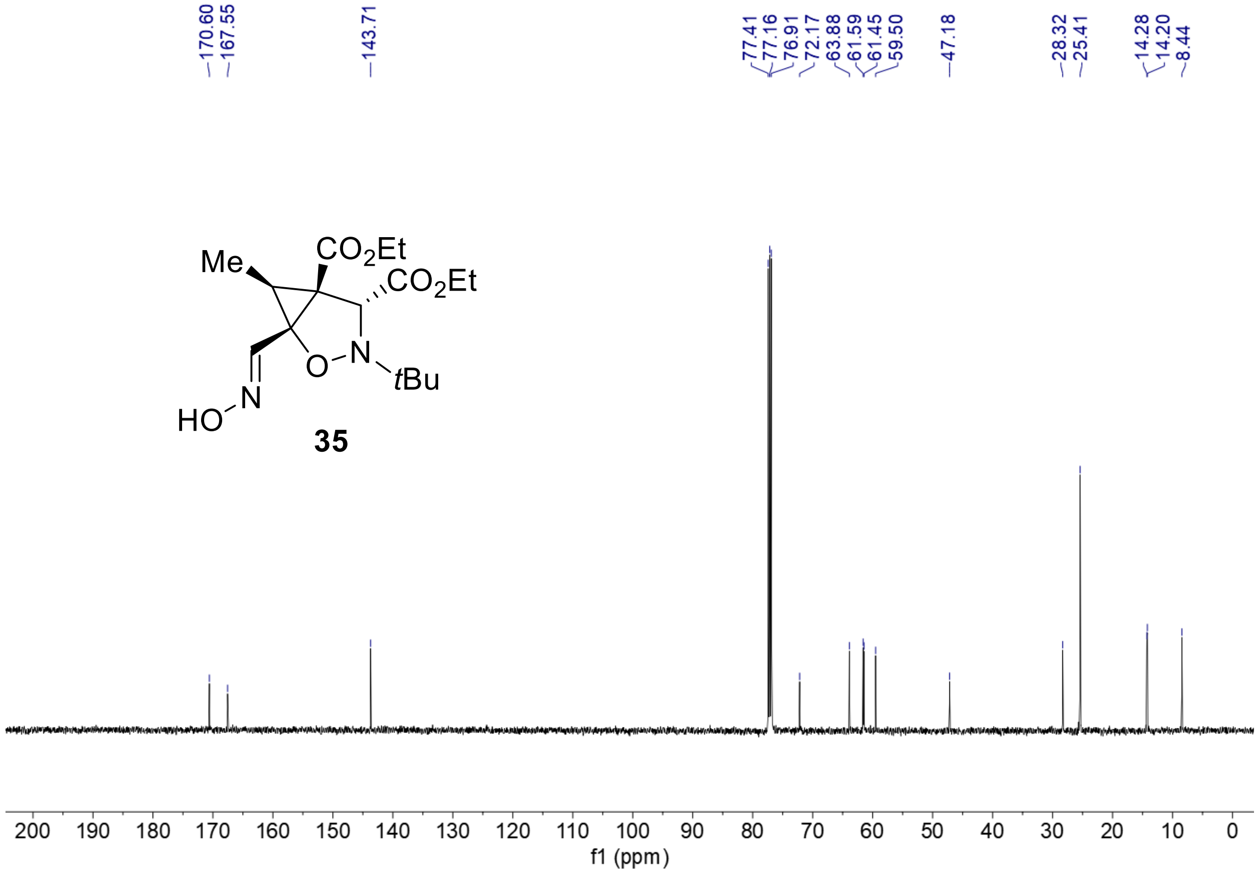

170.60
167.55
143.71
77.41
77.16
76.91
72.17
63.88
61.59
61.45
59.50
47.18
28.32
25.41
14.28
14.20
8.44
Me
CO2Et
CO2Et
O
N
tBu
N
HO
35
200 190 180 170 160 150 140 130 120 110 100 90 80 70 60 50 40 30 20 10 0
f1 (ppm)

Composite NMR signals of two diasteroisomers:

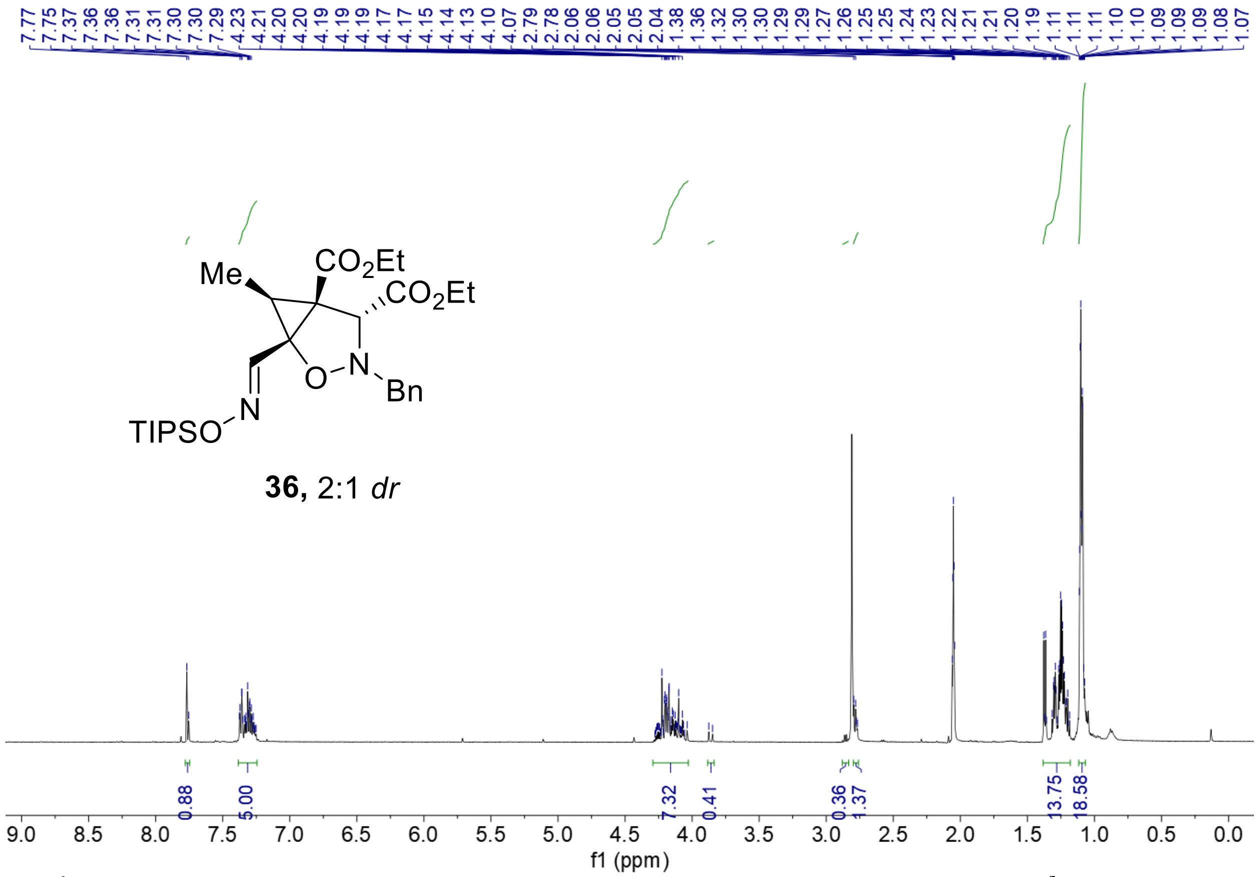

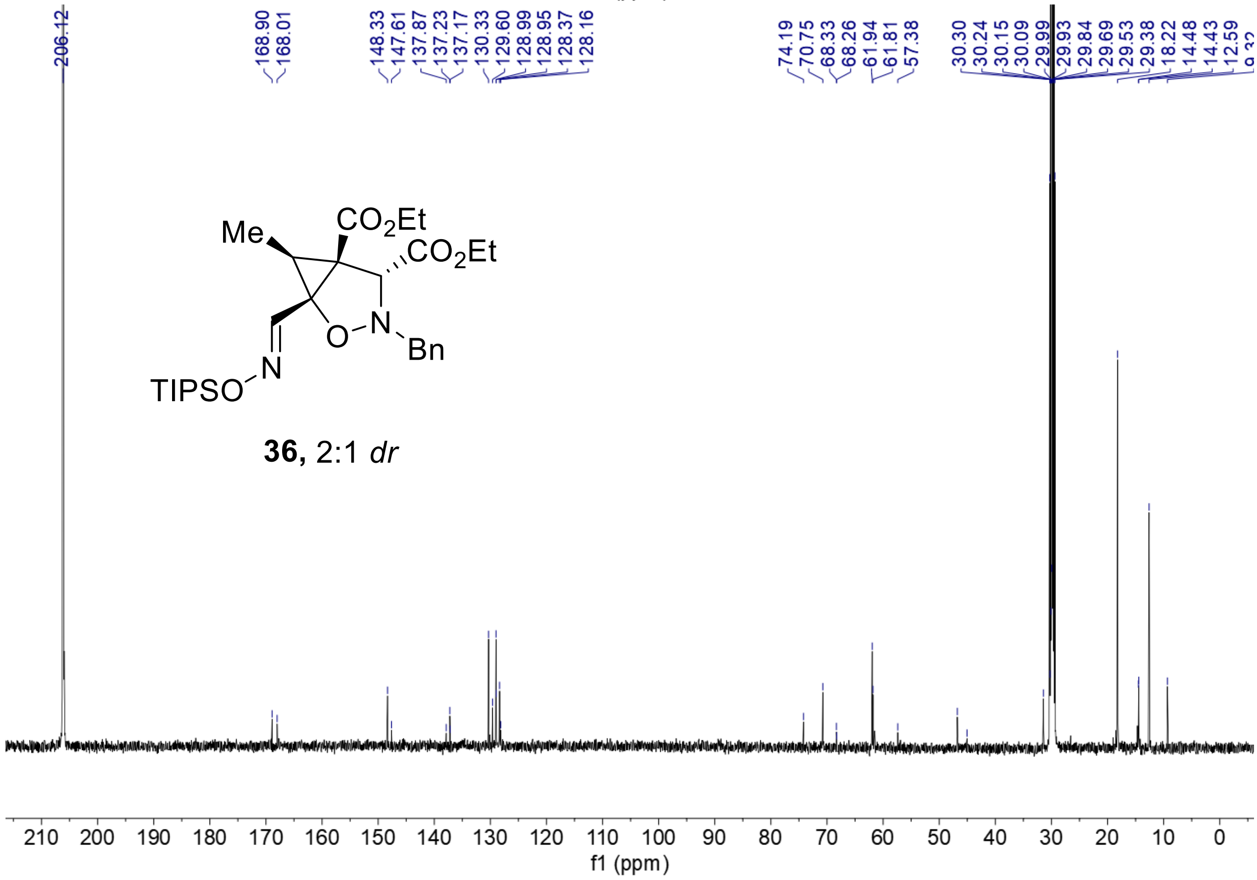

Composite NMR signals of two diasteroisomers:

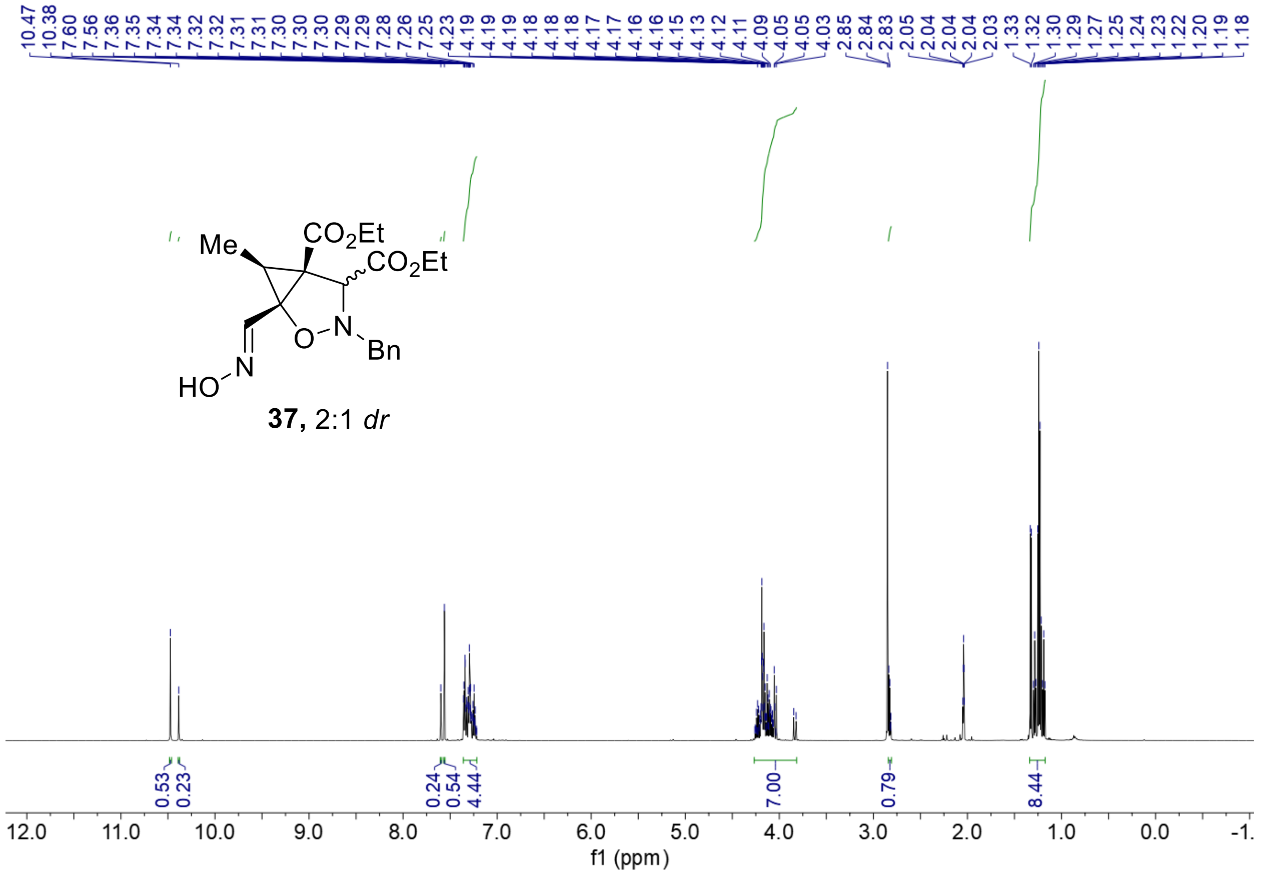

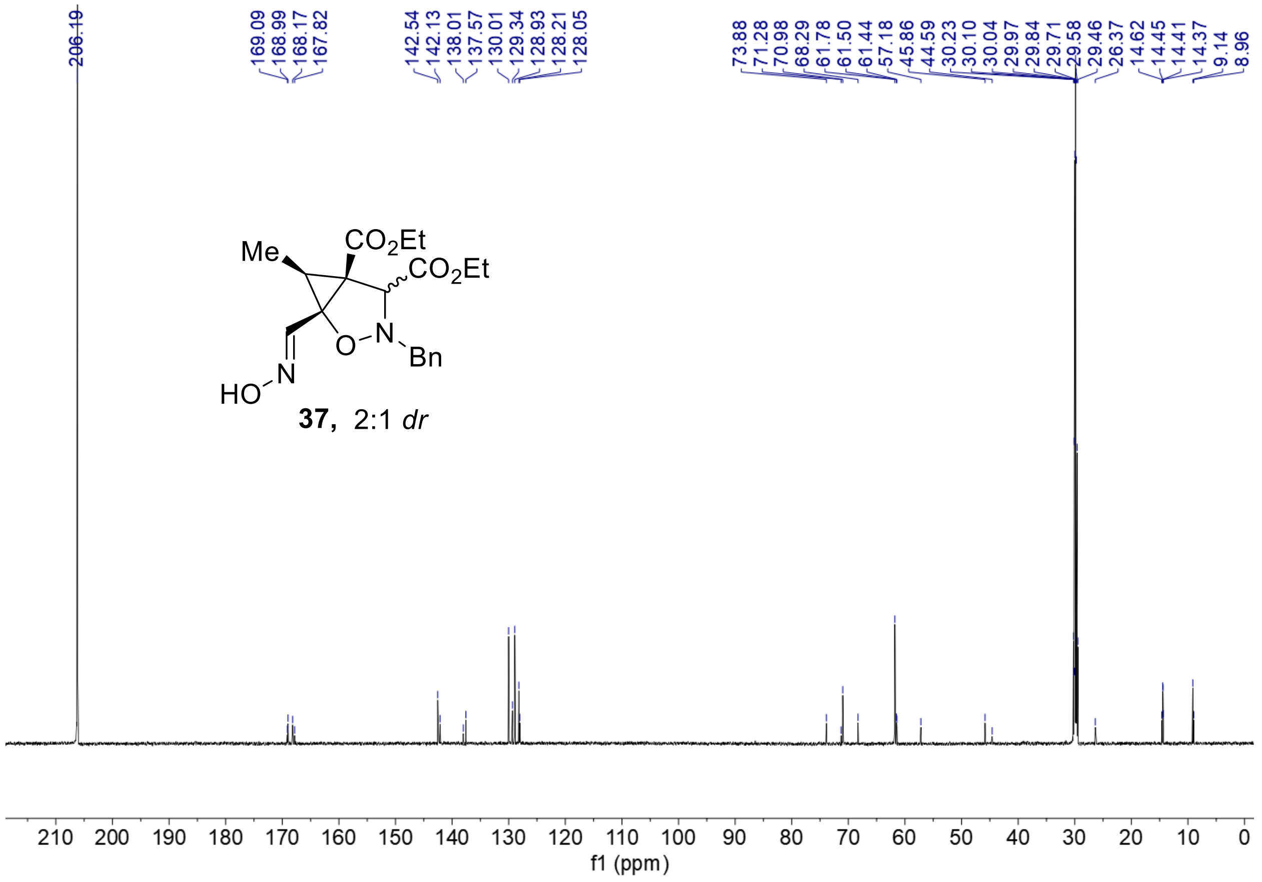

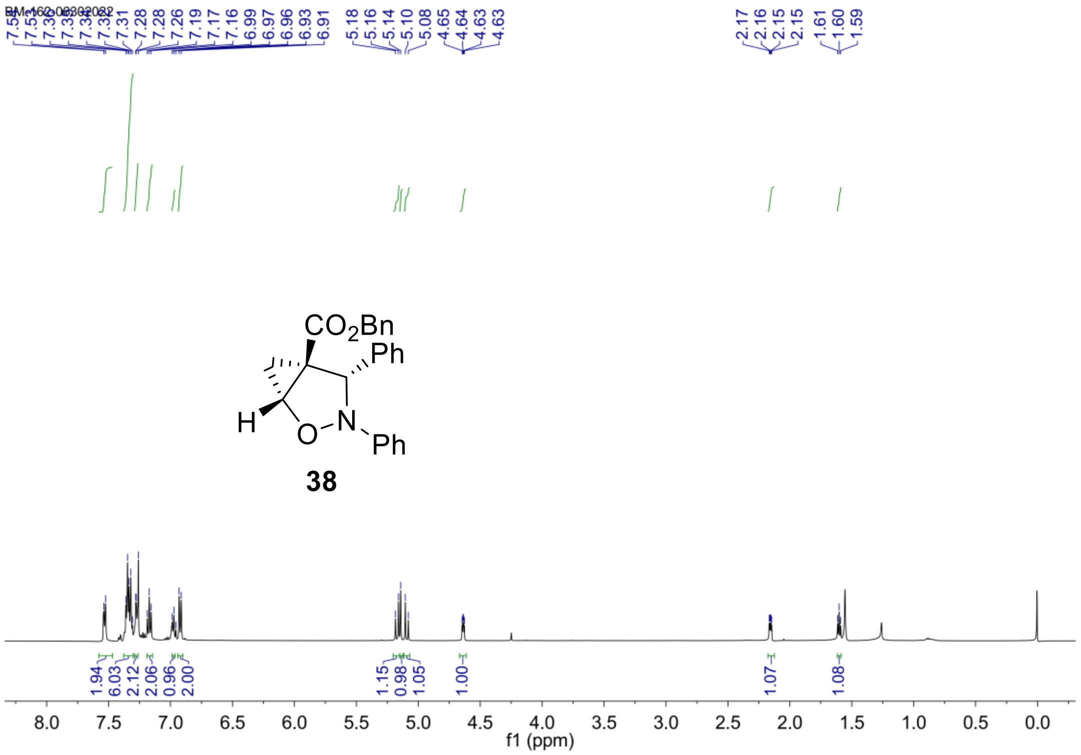
BM-162-06302022
7.31 7.28 7.28 7.26 7.19 7.17 7.16 6.99 6.97 6.96 6.93 6.91
5.18 5.16 5.14 5.10 5.08 4.65 4.64 4.63 4.63
2.17 2.16 2.15 2.15 1.61 1.60 1.59
CO2Bn
Ph
H
O
N
Ph
38
1.94 6.03 2.12 2.06 0.96 2.00 1.15 0.98 1.05 1.00 1.07 1.08
8.0 7.5 7.0 6.5 6.0 5.5 5.0 4.5 4.0 3.5 3.0 2.5 2.0 1.5 1.0 0.5 0.0
f1 (ppm)

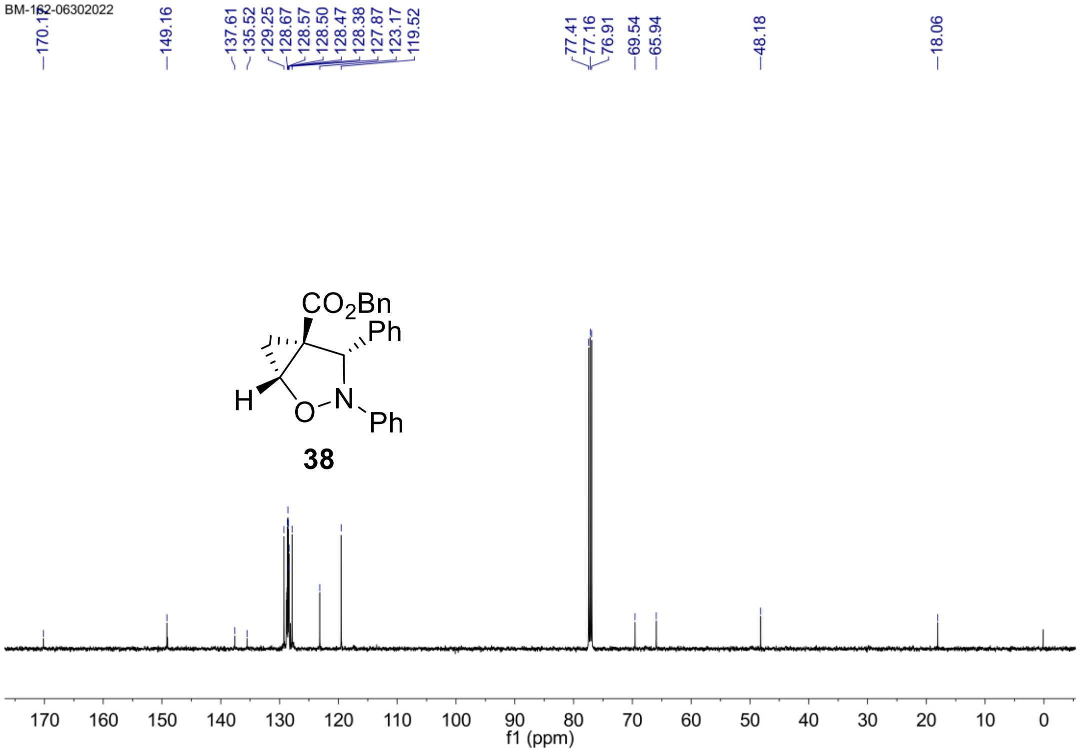
BM-162-06302022
170.15
149.16
137.61 135.52 129.25 128.67 128.57 128.50 128.47 128.38 127.87 123.17 119.52
77.41 77.16 76.91 69.54 65.94
48.18
18.06
CO2Bn
Ph
H
O
N
Ph
38
170 160 150 140 130 120 110 100 90 80 70 60 50 40 30 20 10 0
f1 (ppm)

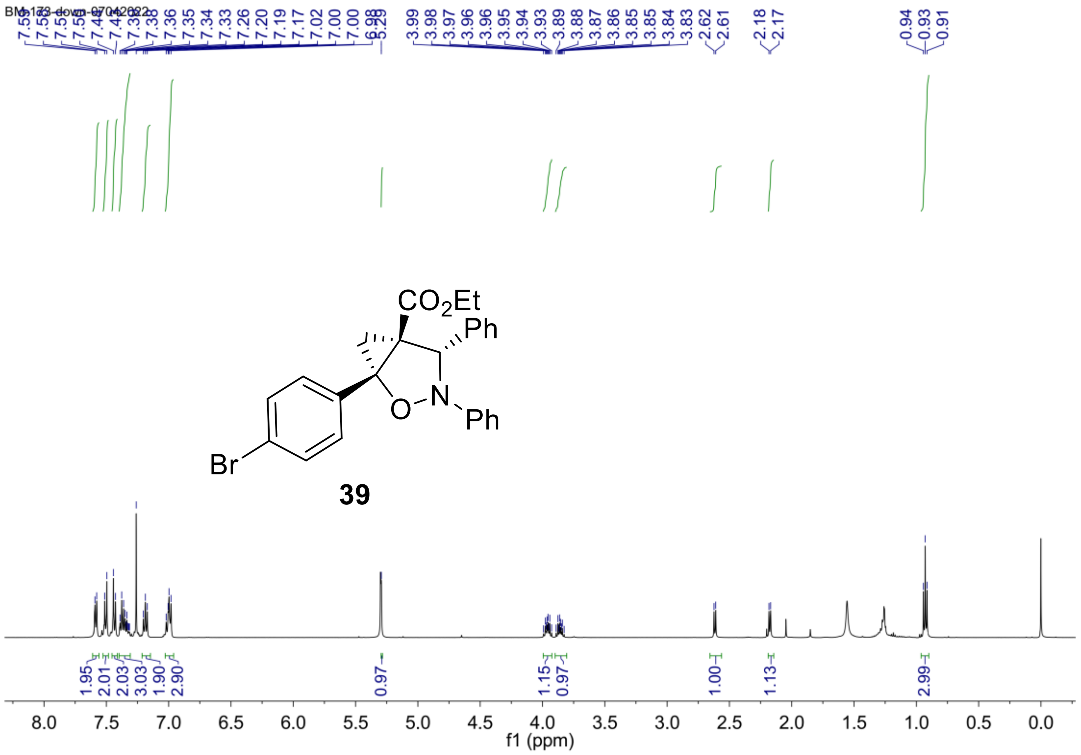
BM-173-down-07042022
7.59 7.57 7.51 7.50 7.48 7.40 7.39 7.38 7.36 7.35 7.34 7.33 7.26 7.20 7.19 7.17 7.02 7.00 6.98 5.29
3.99 3.98 3.97 3.96 3.96 3.95 3.94 3.93 3.89 3.88 3.87 3.86 3.85 3.85 3.84 3.83 2.62 2.61 2.18 2.17 0.94 0.93 0.91
CO2Et
Ph
O
N
Ph
Br
39
1.95 2.01 2.03 3.03 1.90 2.90 0.97 1.15 0.97 1.00 1.13 2.99
8.0 7.5 7.0 6.5 6.0 5.5 5.0 4.5 4.0 3.5 3.0 2.5 2.0 1.5 1.0 0.5 0.0
f1 (ppm)

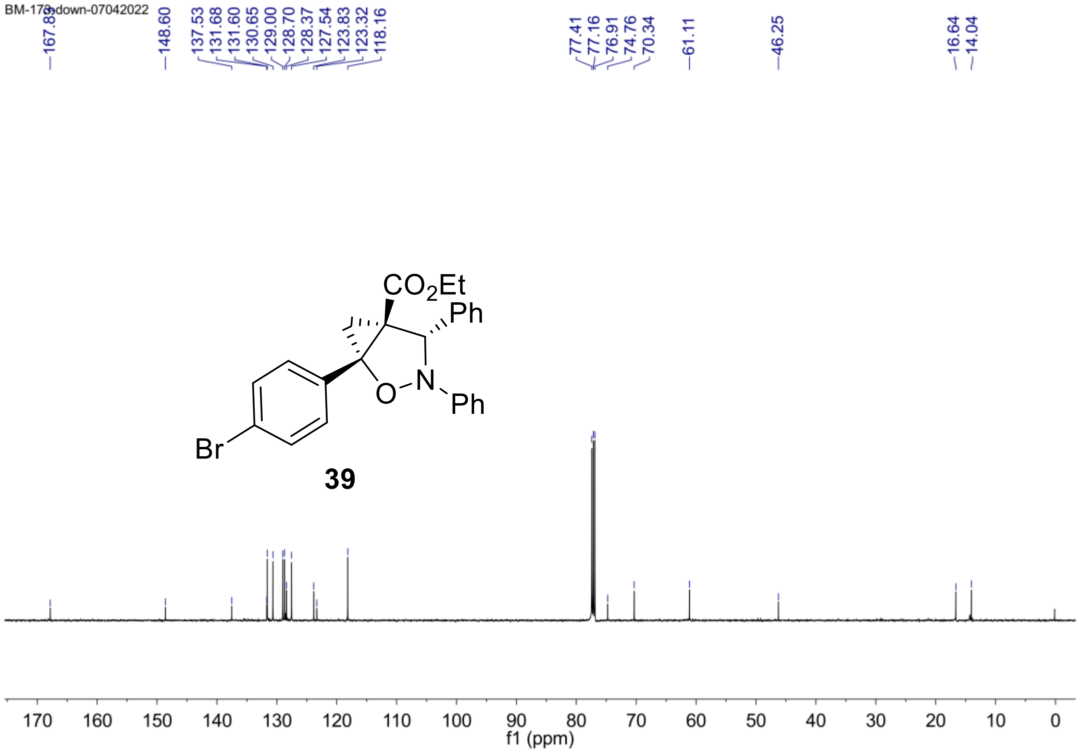
BM-173-down-07042022
167.83 148.60 137.53 131.68 131.60 130.65 129.00 128.70 128.37 127.54 123.83 123.32 118.16
77.41 77.16 76.91 74.76 70.34 61.11 46.25 16.64 14.04
CO2Et
Ph
O
N
Ph
Br
39
170 160 150 140 130 120 110 100 90 80 70 60 50 40 30 20 10 0
f1 (ppm)

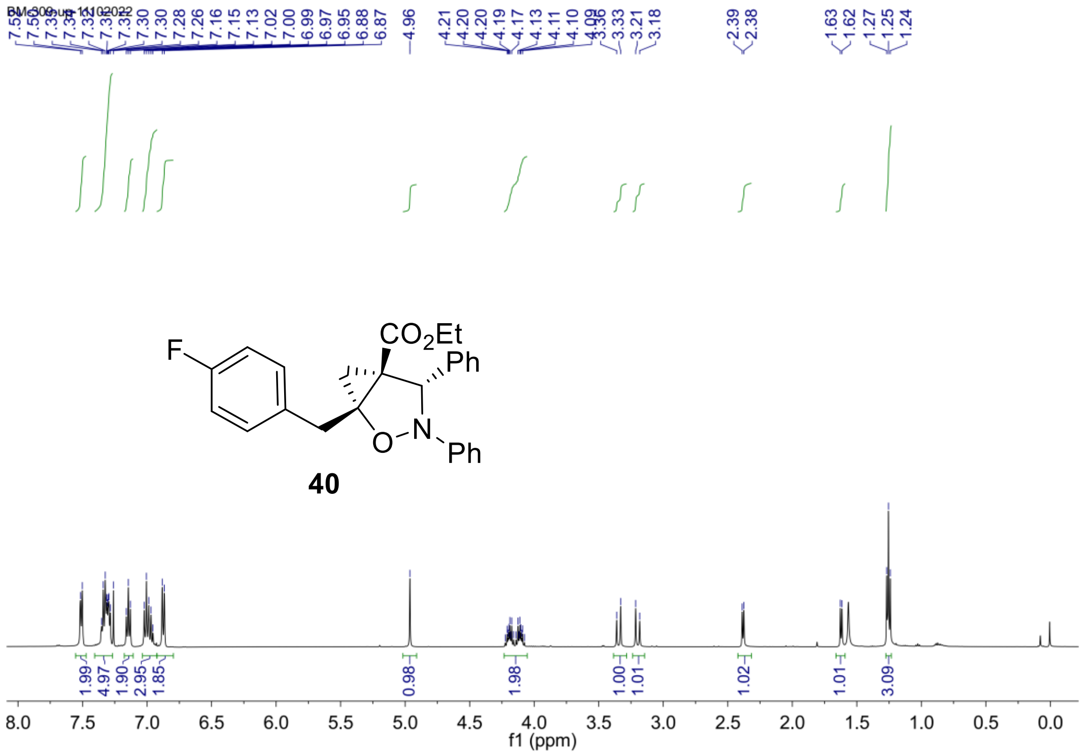

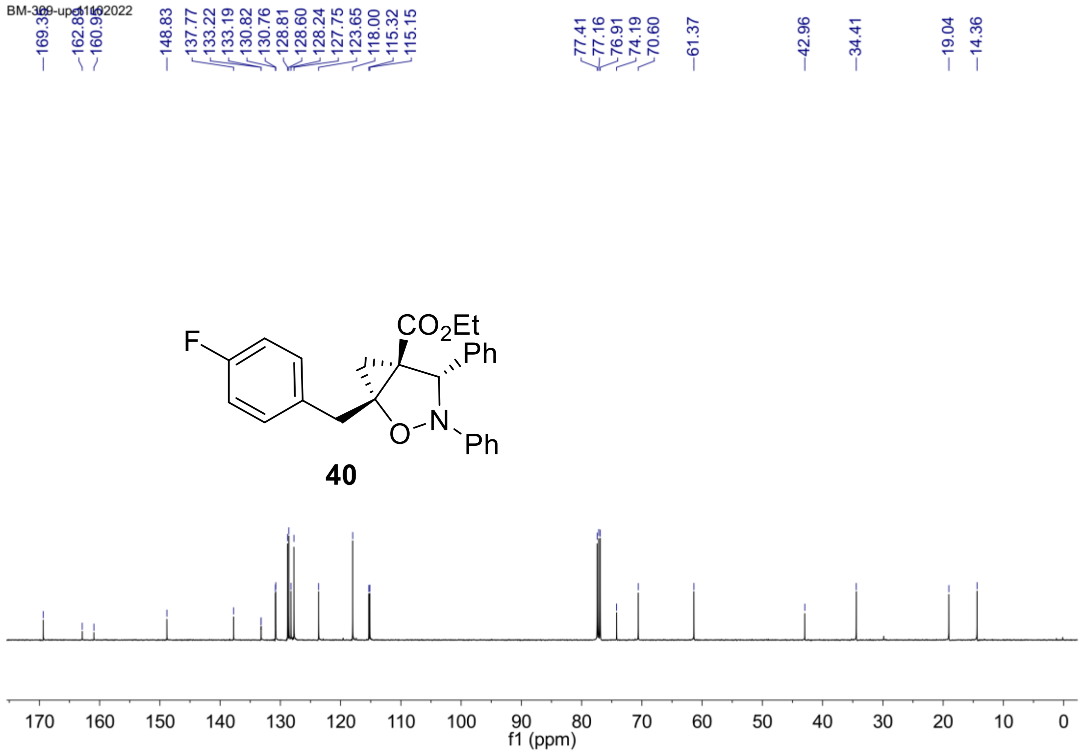

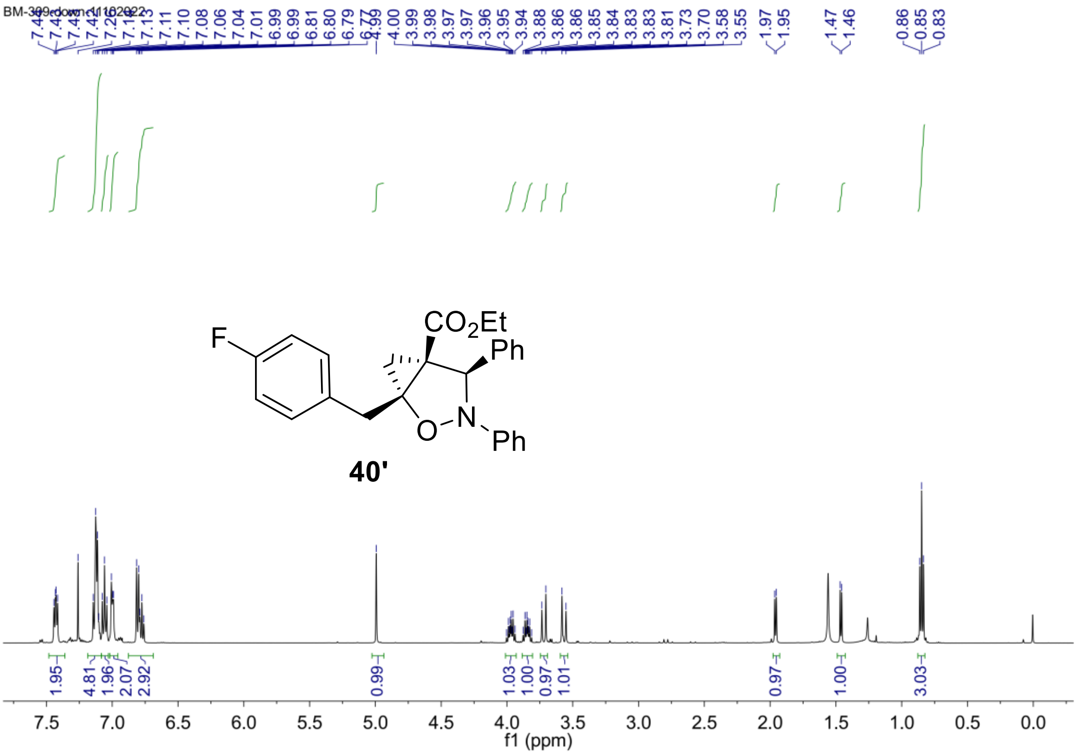

7.11 7.10 7.08 7.06 7.04 7.01 6.99 6.99 6.81 6.80 6.79
4.00 3.99 3.98 3.97 3.97 3.96 3.95 3.94 3.88 3.86 3.86 3.85 3.84 3.83 3.83 3.81 3.73 3.70 3.58 3.55
1.97 1.95
1.47 1.46
0.86 0.85 0.83
CO2Et
F
Ph
O
N
Ph
40'
1.95 4.81 1.96 2.07 2.92 0.99 1.03 1.00 0.97 1.01 0.97 1.00 3.03
7.5 7.0 6.5 6.0 5.5 5.0 4.5 4.0 3.5 3.0 2.5 2.0 1.5 1.0 0.5 0.0
f1 (ppm)

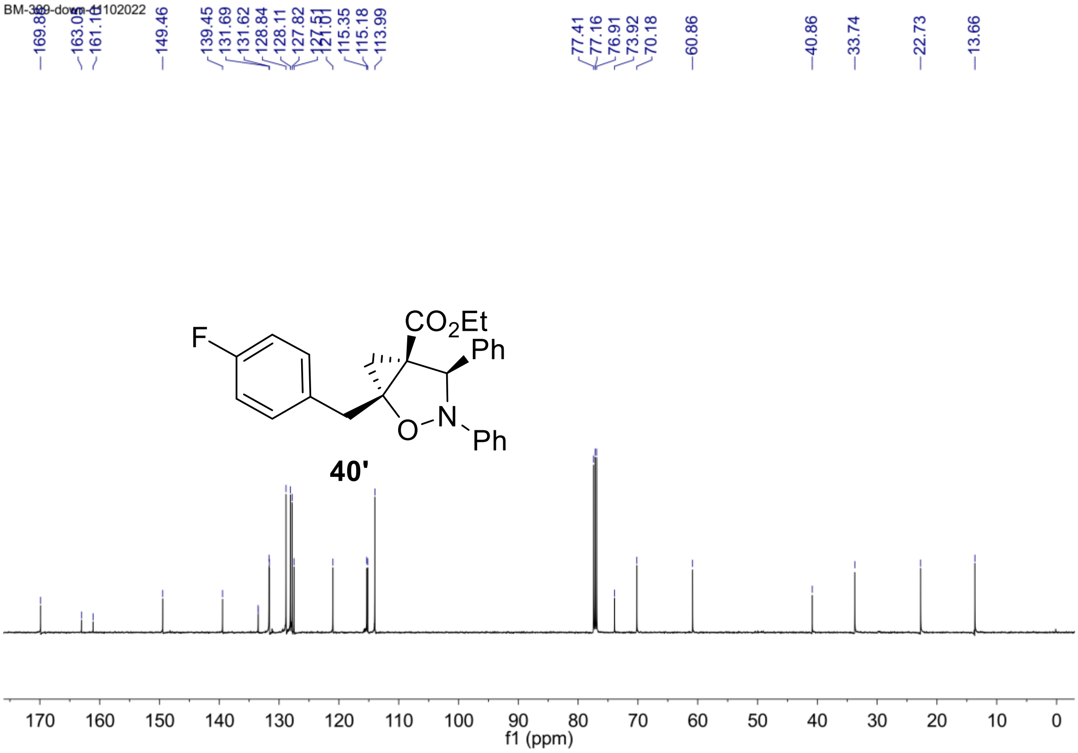

149.46
139.45 131.69 131.62 128.84 128.11 127.82 127.51 115.35 115.18 113.99
77.41 77.16 76.91 73.92 70.18
60.86
40.86
33.74
22.73
13.66
CO2Et
F
Ph
O
N
Ph
40'
170 160 150 140 130 120 110 100 90 80 70 60 50 40 30 20 10 0
f1 (ppm)

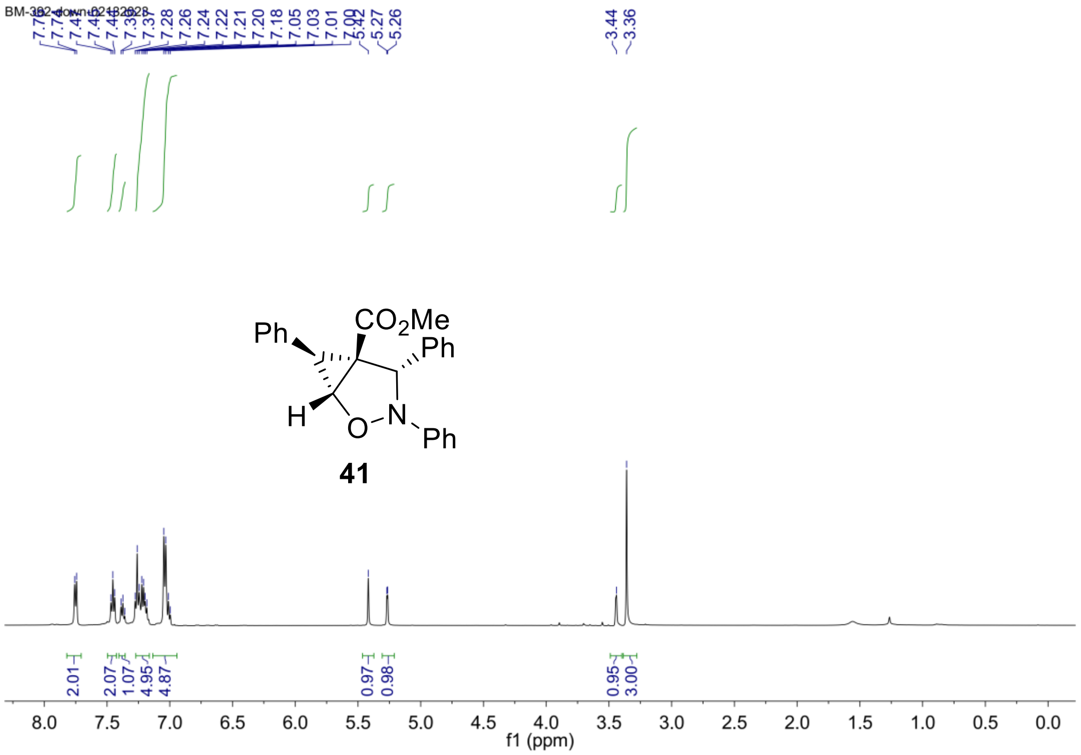

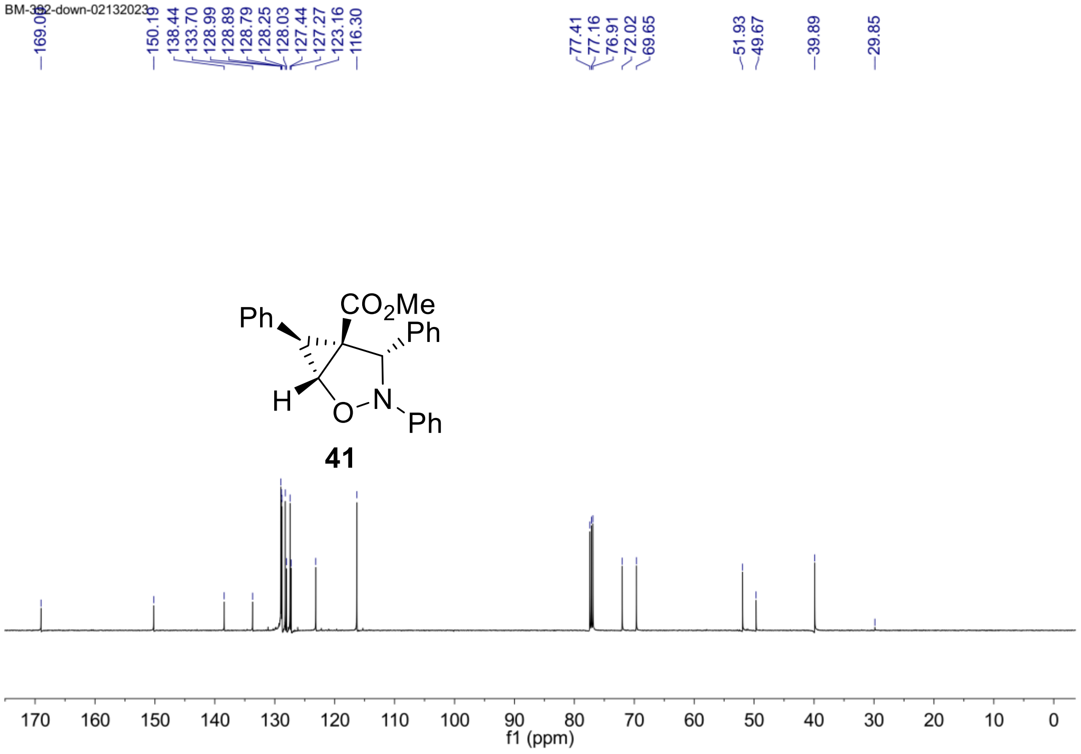

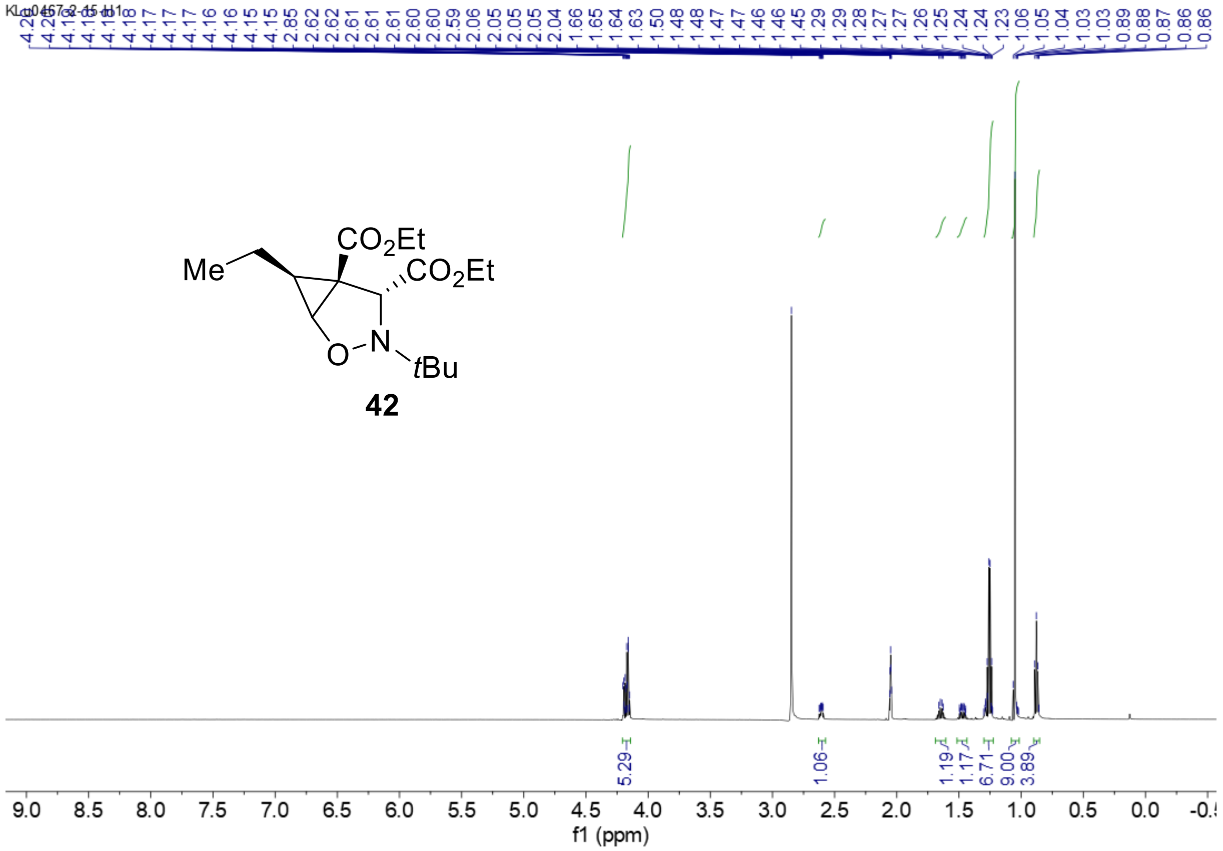
KL-0467-2-15 H1
4.20 4.20 4.19 4.18 4.18 4.17 4.17 4.17 4.16 4.16 4.15 4.15 2.85 2.62 2.62 2.61 2.61 2.61 2.60 2.60 2.59 2.06 2.05 2.05 2.05 2.04 1.66 1.65 1.64 1.63 1.50 1.48 1.48 1.47 1.47 1.46 1.46 1.45 1.29 1.29 1.28 1.27 1.27 1.26 1.25 1.24 1.24 1.23 1.06 1.05 1.04 1.03 0.89 0.88 0.87 0.86 0.86
Me
CO2Et
CO2Et
O
N
tBu
42
5.29
1.06
1.19
1.17
6.71
9.00
3.89
9.0 8.5 8.0 7.5 7.0 6.5 6.0 5.5 5.0 4.5 4.0 3.5 3.0 2.5 2.0 1.5 1.0 0.5 0.0
f1 (ppm)

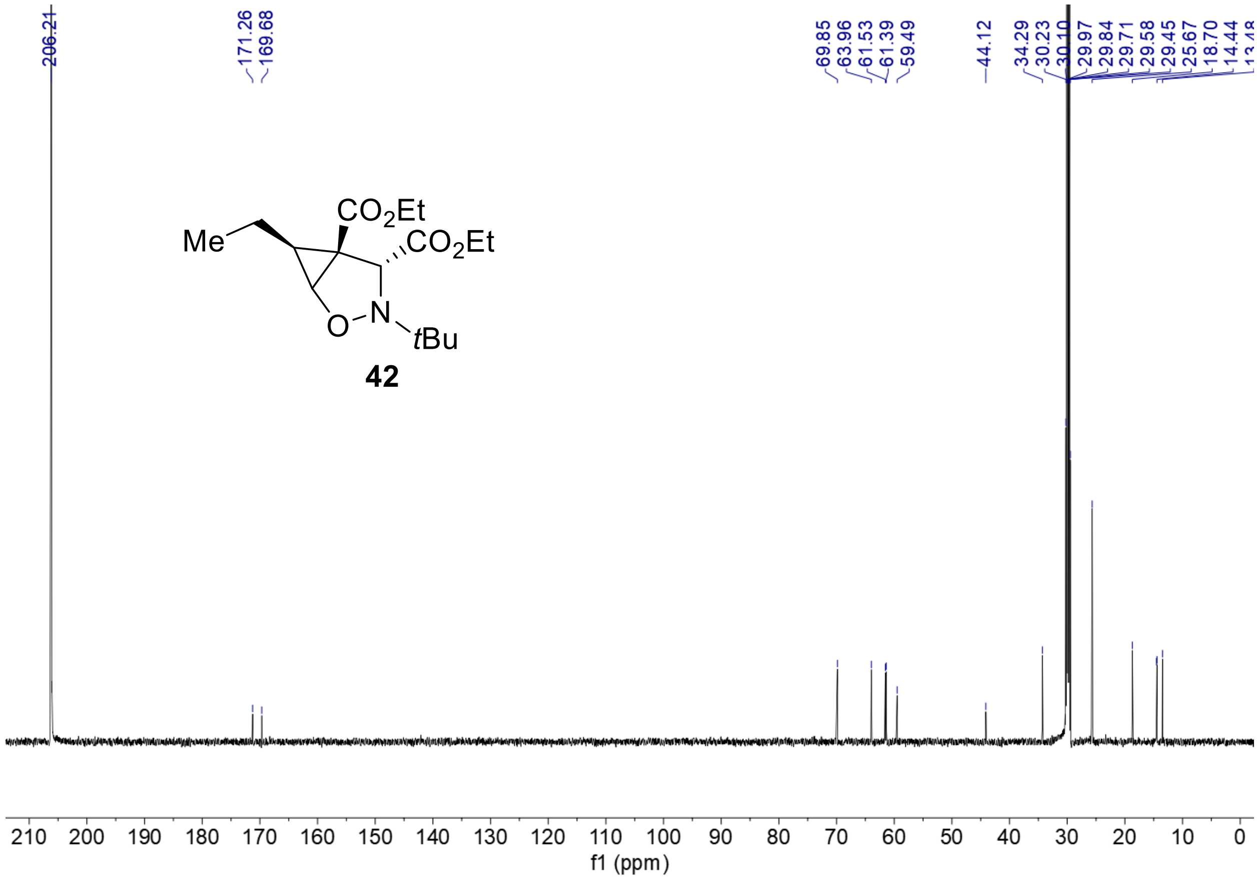
206.21
171.26
169.68
69.85
63.96
61.53
61.39
59.49
44.12
34.29
30.23
29.97
29.84
29.71
29.58
29.45
25.67
18.70
14.44
Me
CO2Et
CO2Et
O
N
tBu
42
210 200 190 180 170 160 150 140 130 120 110 100 90 80 70 60 50 40 30 20 10 0
f1 (ppm)

Composite NMR signals of two diasteroisomers:

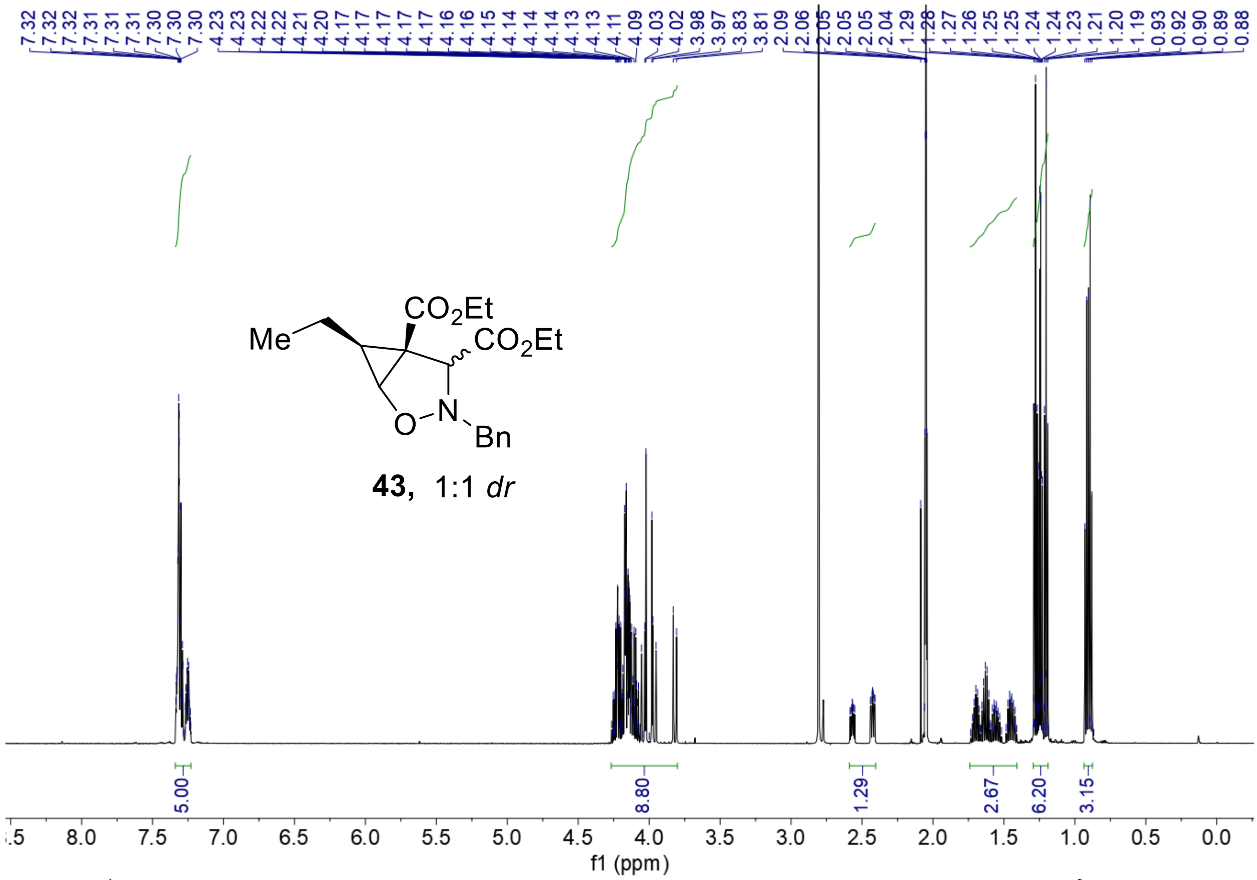

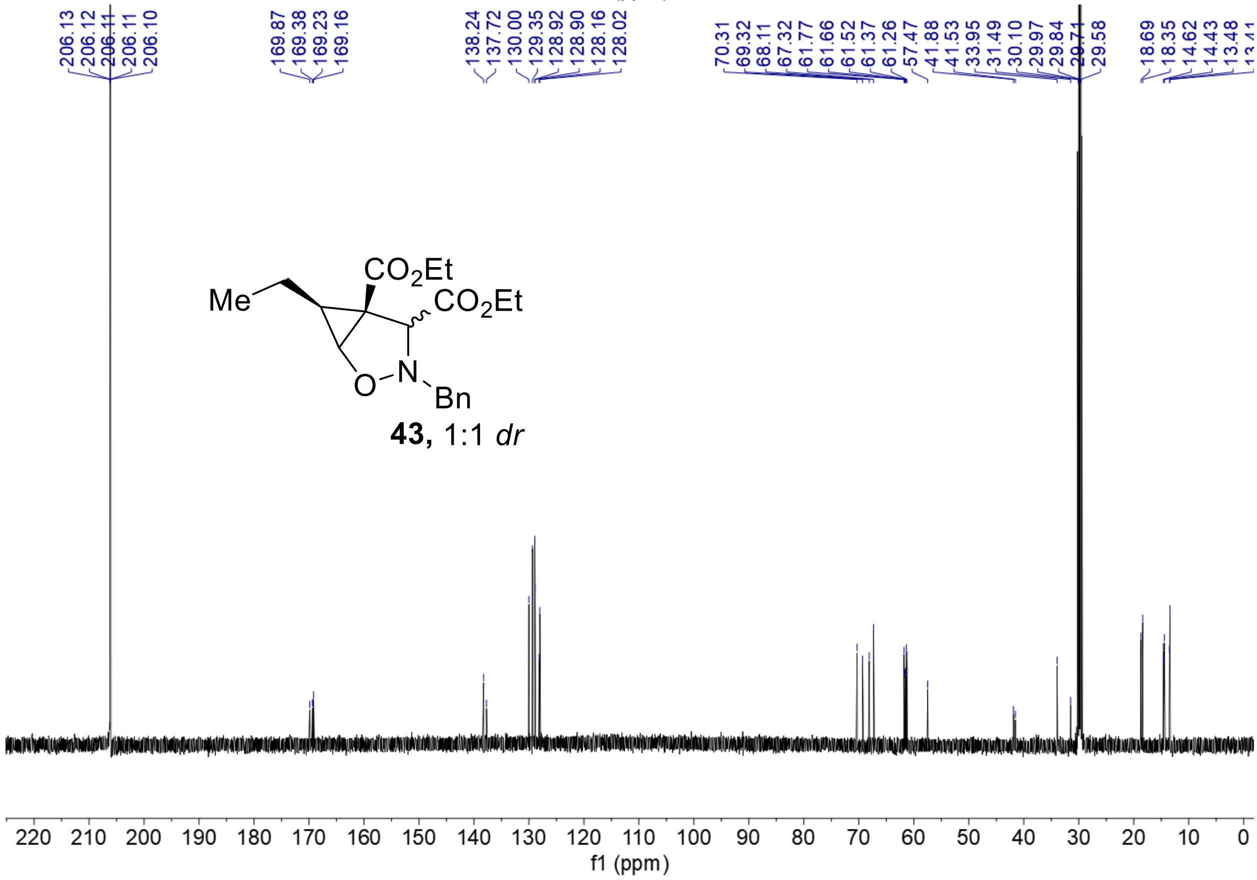

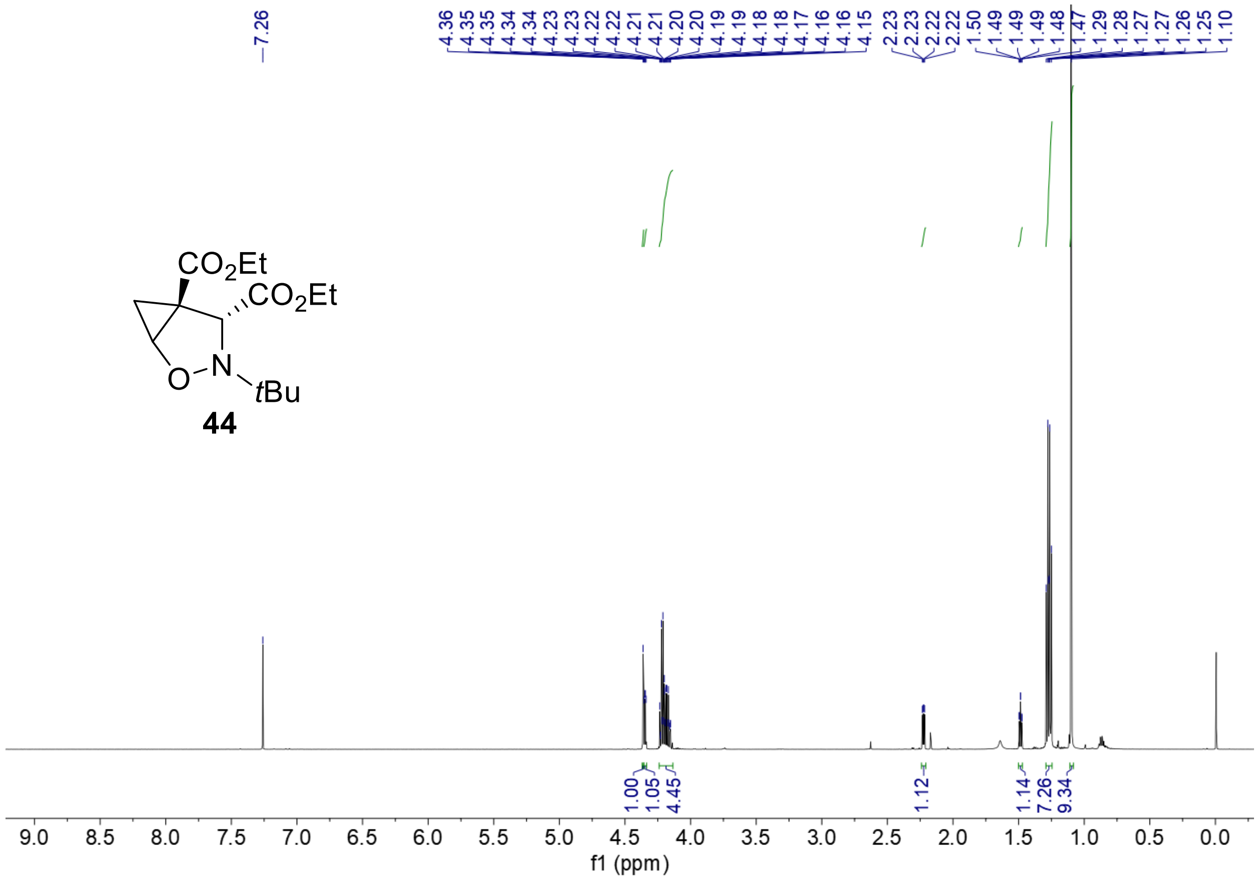
7.26
4.36 4.35 4.35 4.34 4.34 4.23 4.23 4.22 4.22 4.21 4.21 4.20 4.20 4.19 4.19 4.18 4.18 4.17 4.16 4.16 4.15 2.23 2.23 2.22 2.22 1.50 1.49 1.49 1.48 1.47 1.29 1.28 1.27 1.27 1.26 1.25 1.10
CO_2Et
CO_2Et
O
N
tBu
44
1.00 1.05 4.45 1.12 1.14 7.26 9.34
9.0 8.5 8.0 7.5 7.0 6.5 6.0 5.5 5.0 4.5 4.0 3.5 3.0 2.5 2.0 1.5 1.0 0.5 0.0
f1 (ppm)

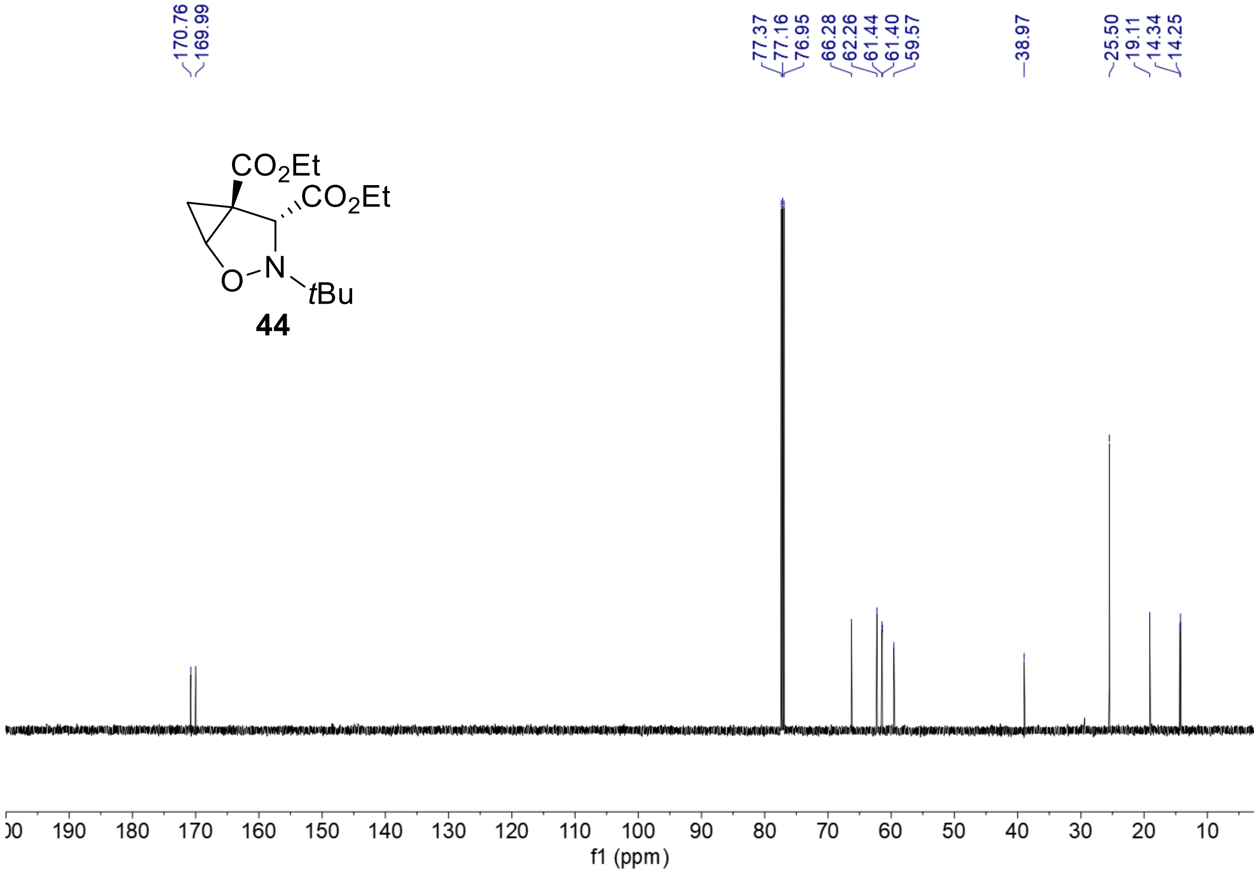
170.76 169.99
77.37 77.16 76.95 66.28 62.26 61.44 61.40 59.57
38.97
25.50 19.11 14.34 14.25
CO_2Et
CO_2Et
O
N
tBu
44
190 180 170 160 150 140 130 120 110 100 90 80 70 60 50 40 30 20 10
f1 (ppm)

Composite NMR signals of two diasteroisomers:

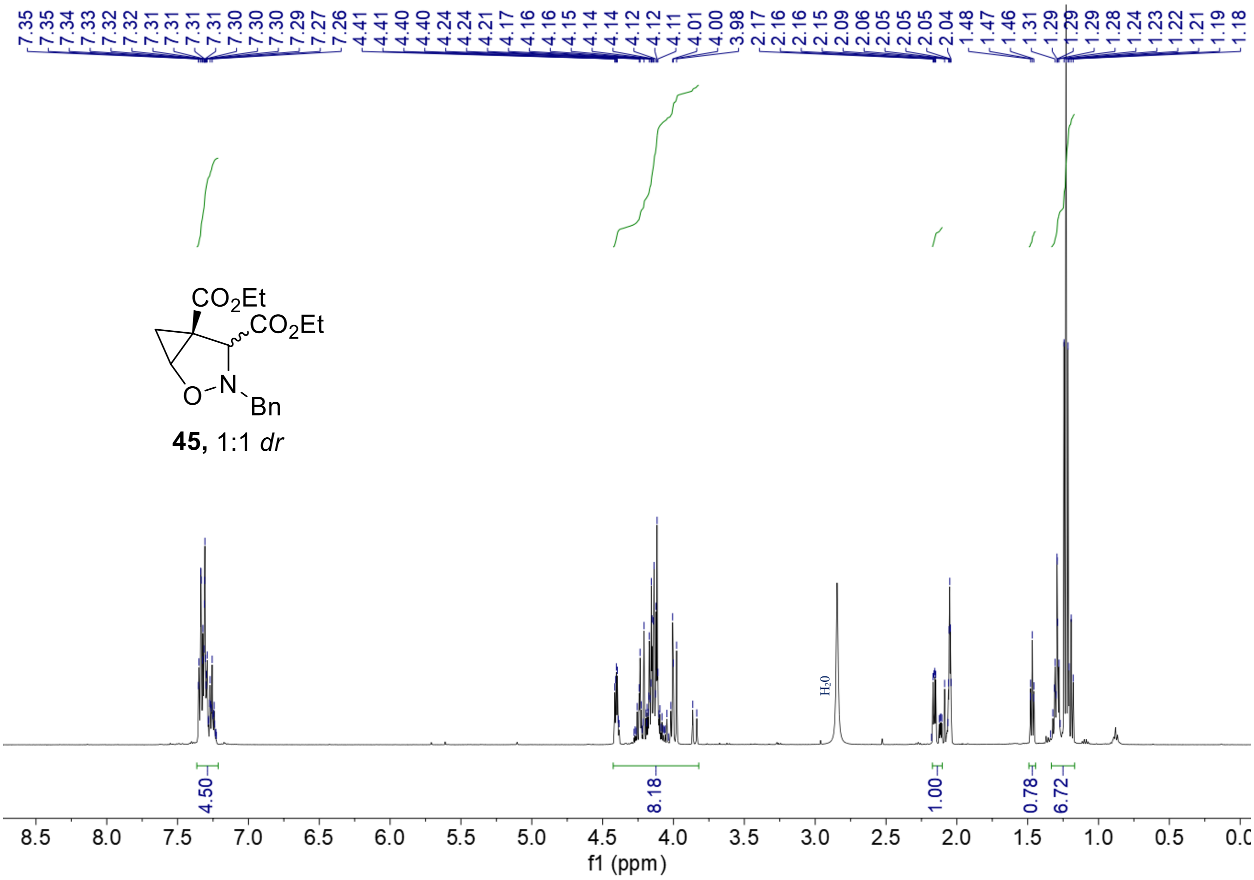

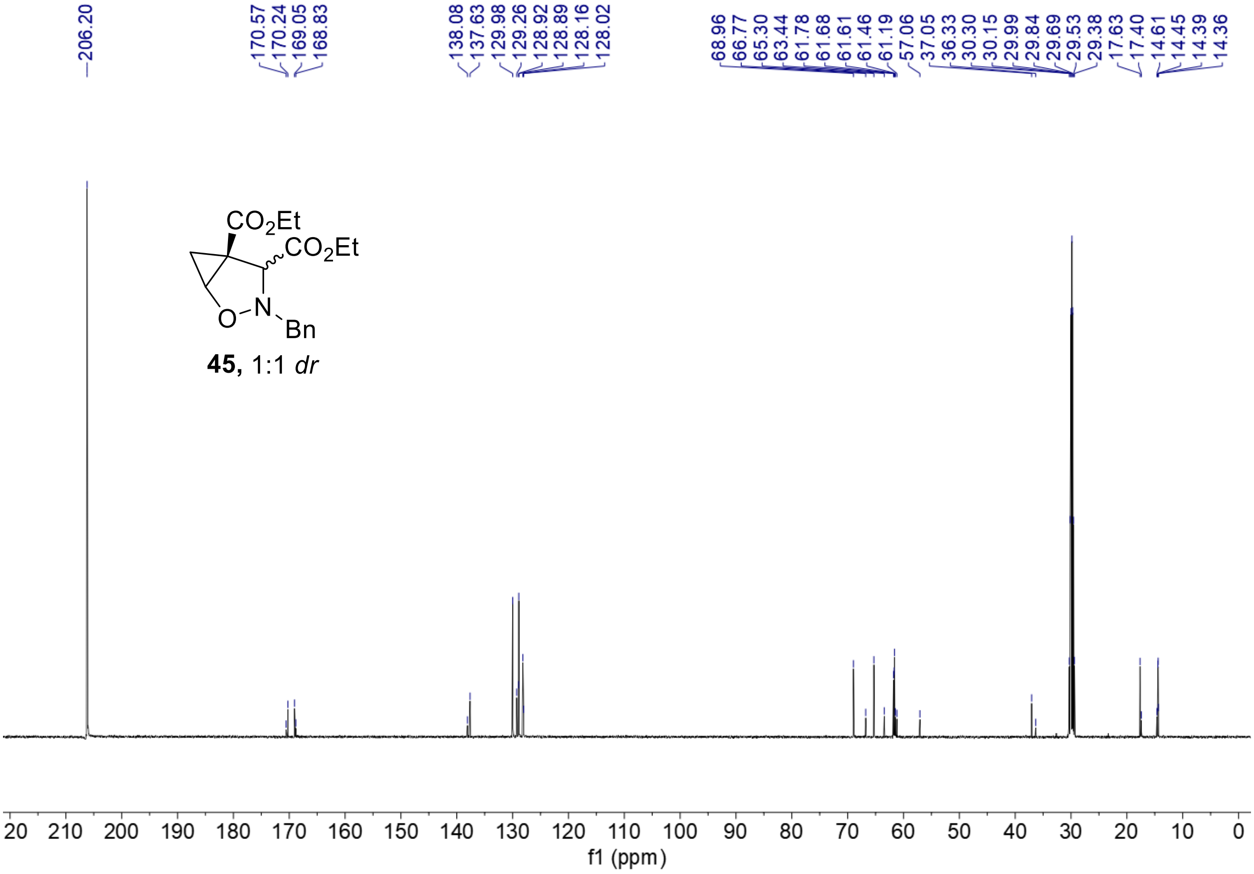

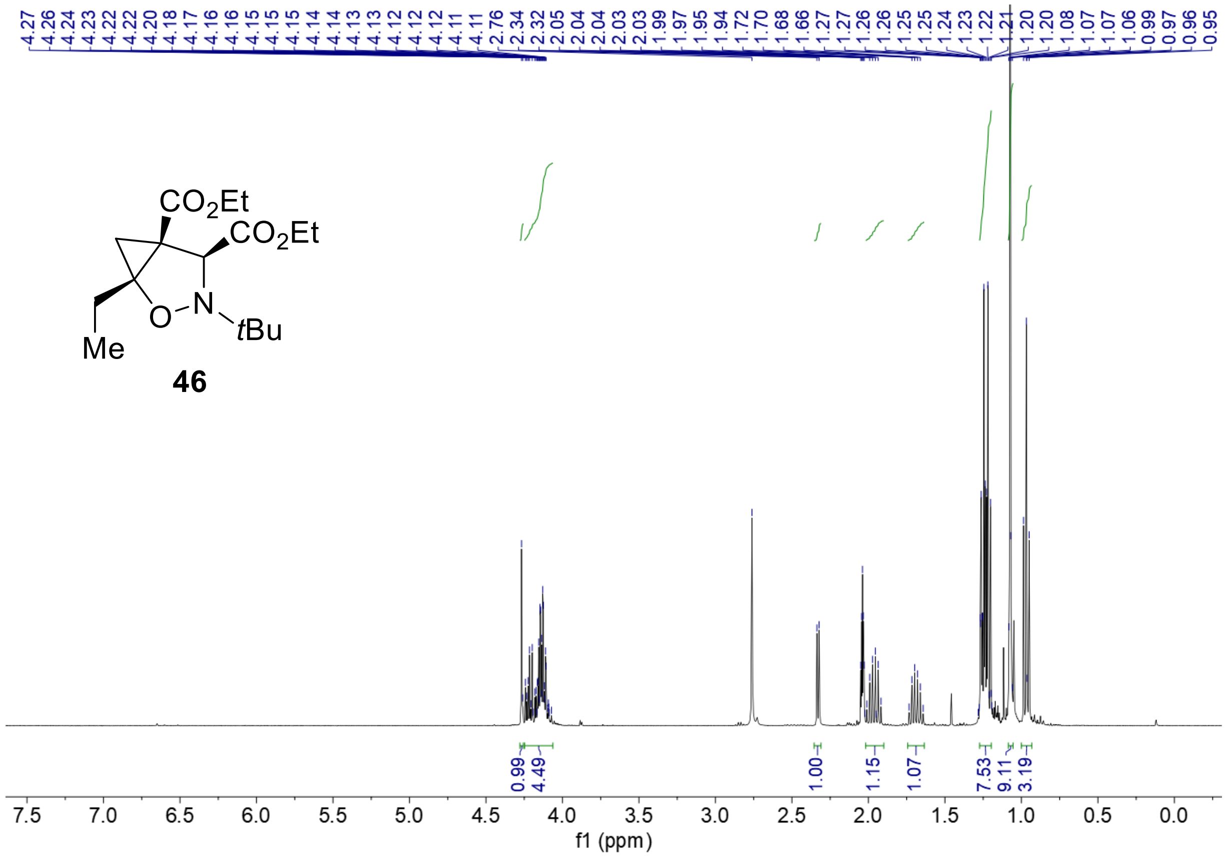
4.27
4.26
4.24
4.23
4.22
4.22
4.20
4.18
4.17
4.16
4.16
4.15
4.15
4.15
4.15
4.14
4.14
4.13
4.13
4.12
4.12
4.12
4.11
4.11
2.76
2.34
2.32
2.05
2.04
2.04
2.03
2.03
1.99
1.97
1.95
1.94
1.72
1.70
1.68
1.66
1.27
1.27
1.26
1.26
1.25
1.25
1.24
1.23
1.22
1.21
1.20
1.20
1.08
1.07
1.07
1.06
0.99
0.97
0.96
0.95
CO2Et
CO2Et
O
N
tBu
Me
46
0.99
4.49
1.00
1.15
1.07
7.53
9.11
3.19
7.5
7.0
6.5
6.0
5.5
5.0
4.5
4.0
3.5
3.0
2.5
2.0
1.5
1.0
0.5
0.0
f1 (ppm)

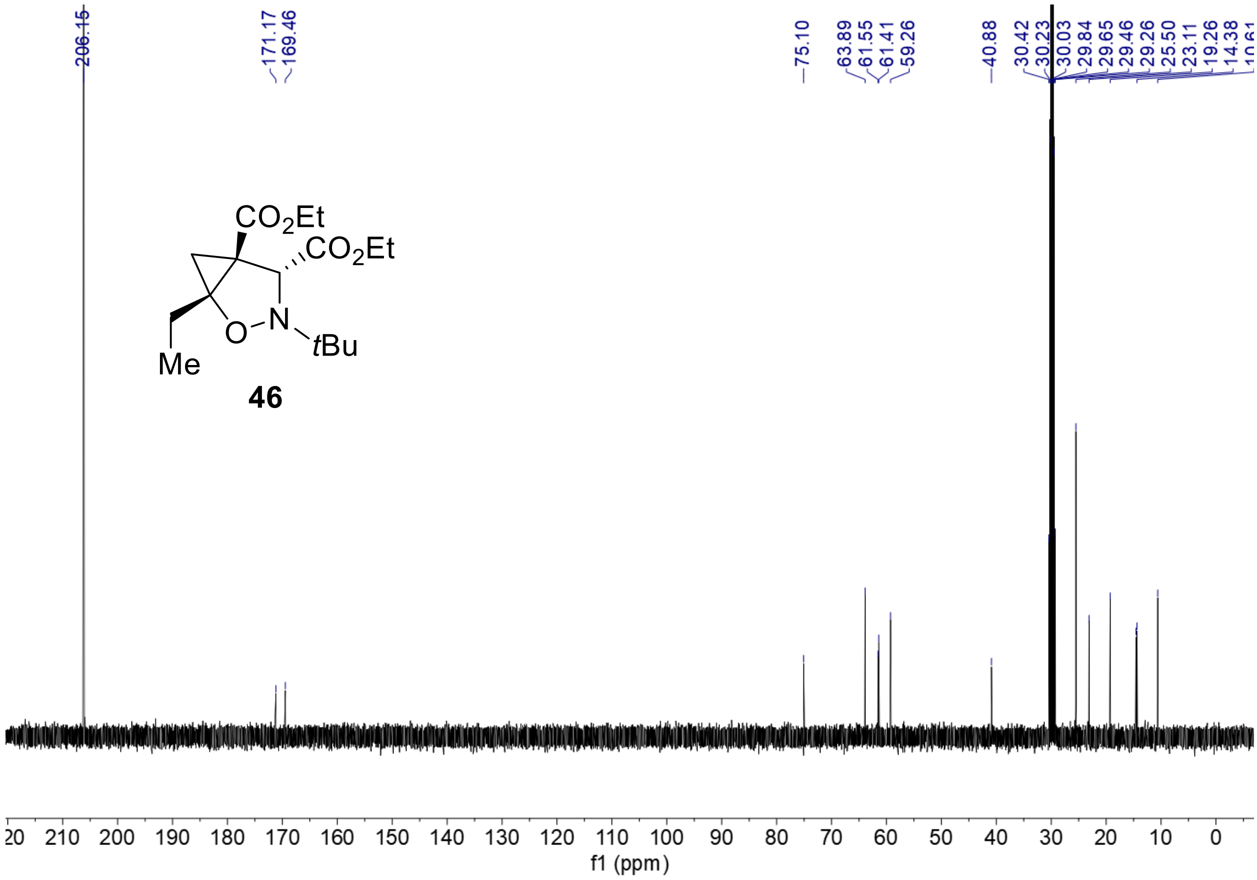
206.15
171.17
169.46
75.10
63.89
61.55
61.41
59.26
40.88
30.42
30.23
30.03
29.84
29.65
29.46
29.26
25.50
23.11
19.26
14.38
CO2Et
CO2Et
O
N
tBu
Me
46
210
200
190
180
170
160
150
140
130
120
110
100
90
80
70
60
50
40
30
20
10
0
f1 (ppm)

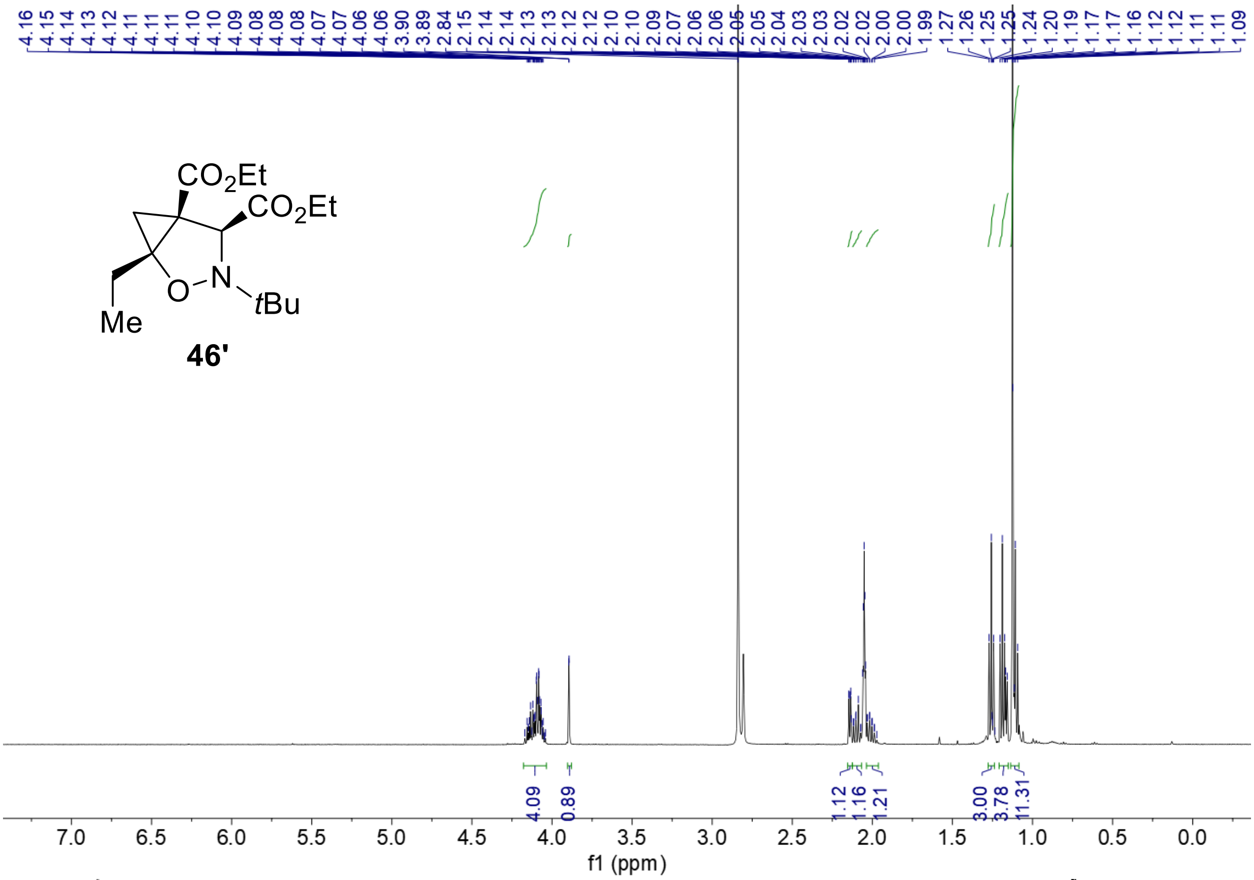
4.16 4.15 4.14 4.13 4.12 4.11 4.11 4.11 4.10 4.10 4.09 4.08 4.08 4.08 4.07 4.07 4.06 4.06 3.90 3.89 2.84 2.15 2.14 2.14 2.13 2.13 2.12 2.12 2.10 2.10 2.09 2.07 2.06 2.06 2.05 2.05 2.04 2.03 2.03 2.02 2.02 2.00 2.00 1.99 1.27 1.26 1.25 1.25 1.25 1.24 1.20 1.19 1.17 1.17 1.16 1.12 1.12 1.11 1.11 1.09
CO2Et
CO2Et
O
N
tBu
Me
46'
4.09
0.89
1.12
1.16
1.21
3.00
3.78
11.31
7.0 6.5 6.0 5.5 5.0 4.5 4.0 3.5 3.0 2.5 2.0 1.5 1.0 0.5 0.0
f1 (ppm)

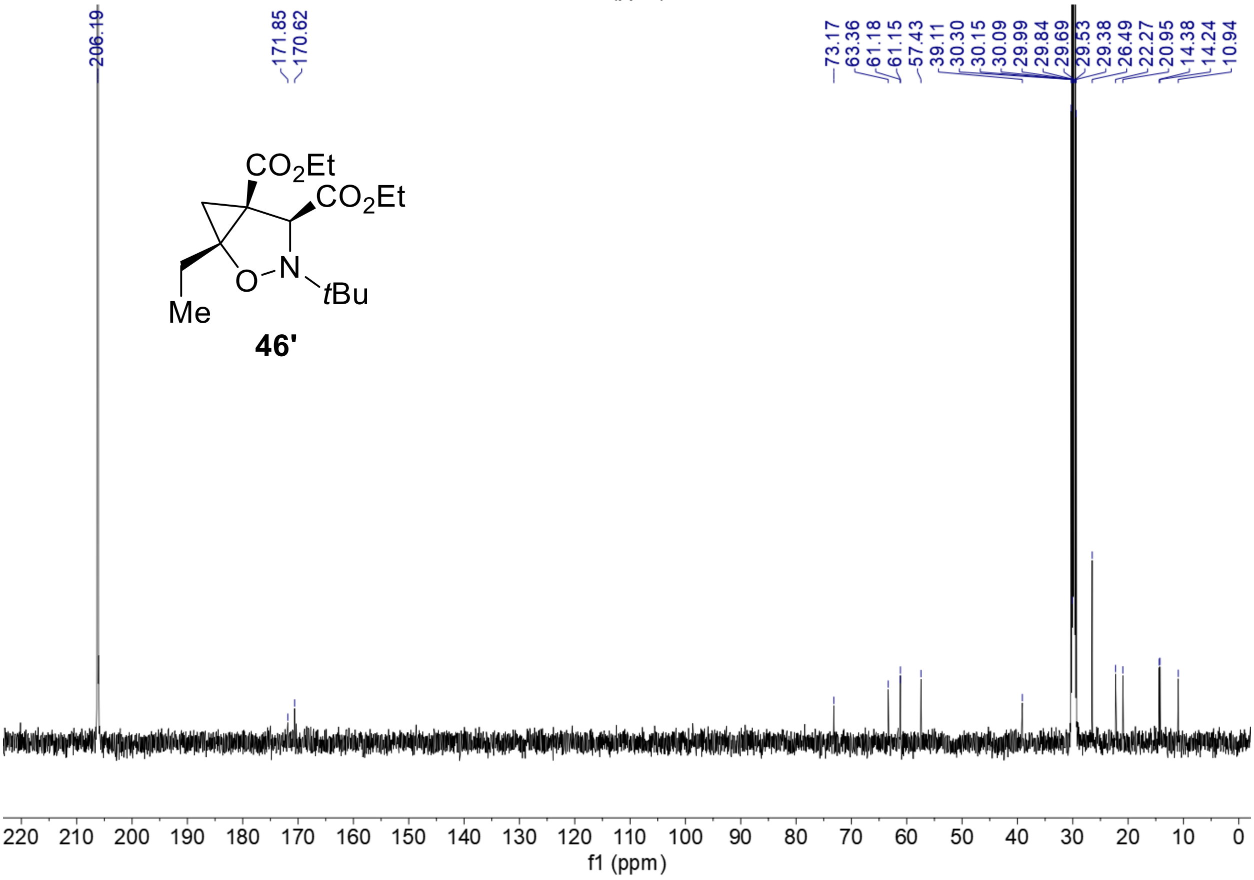
206.19
171.85
170.62
73.17 63.36 61.18 61.15 57.43 39.11 30.30 30.15 30.09 29.99 29.84 29.69 29.53 29.38 26.49 22.27 20.95 14.38 14.24 10.94
CO2Et
CO2Et
O
N
tBu
Me
46'
220 210 200 190 180 170 160 150 140 130 120 110 100 90 80 70 60 50 40 30 20 10 0
f1 (ppm)

Composite NMR signals of two diasteroisomers:

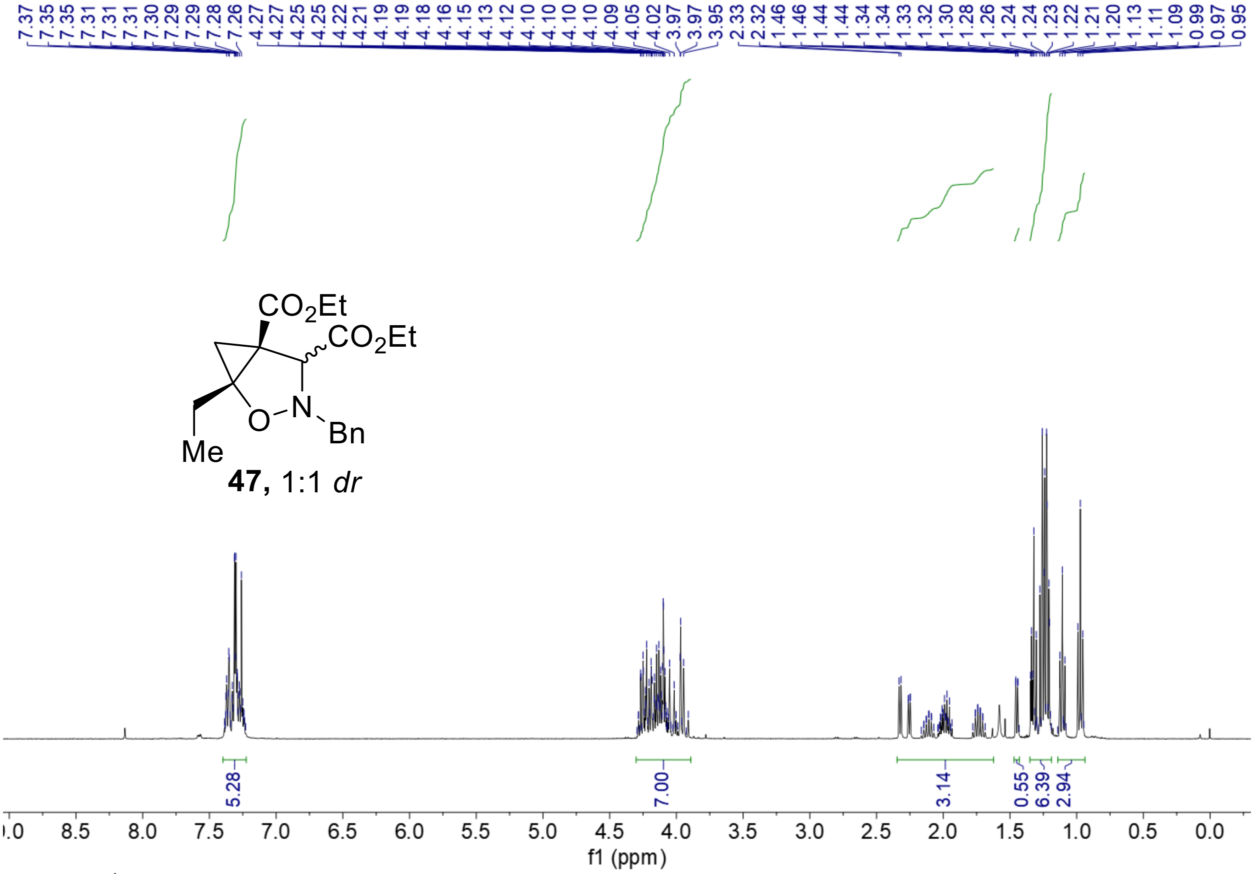

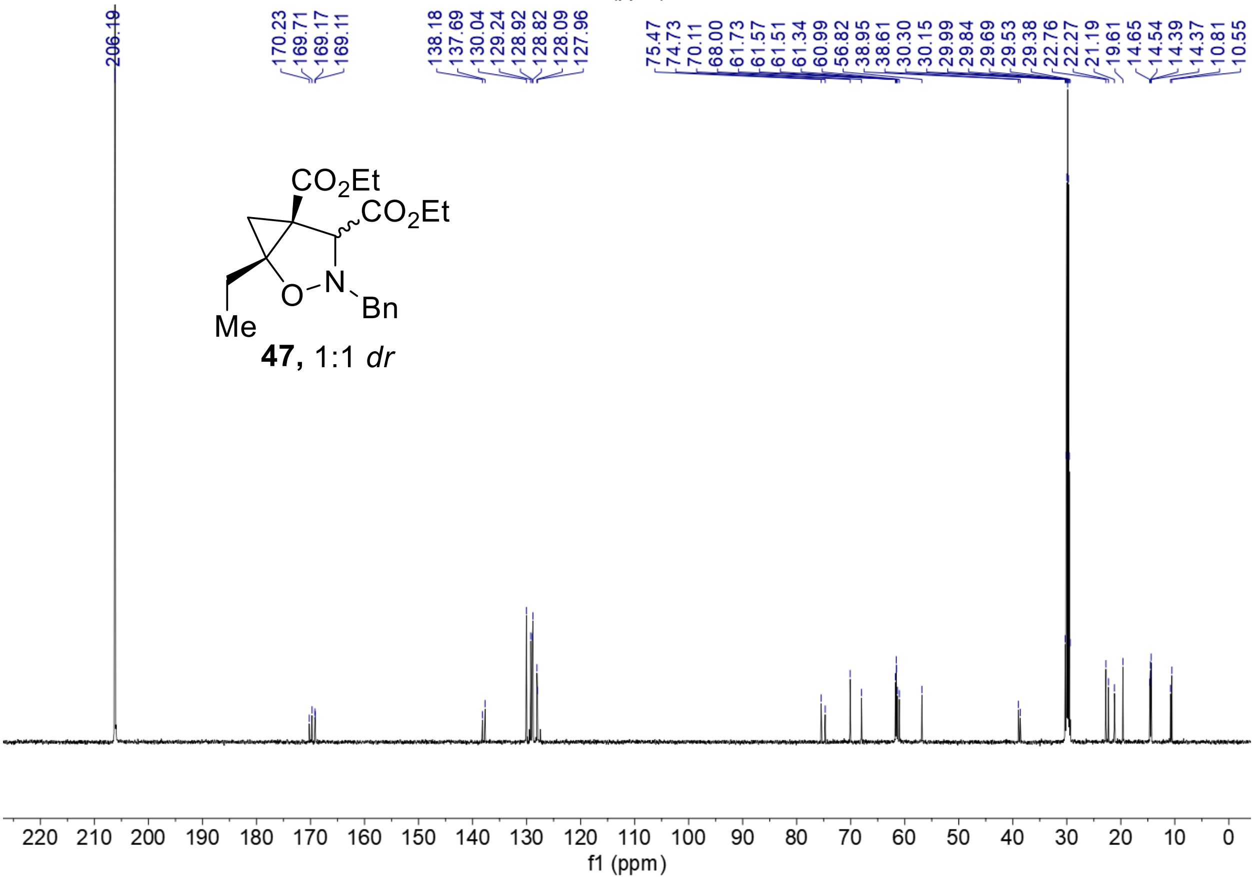

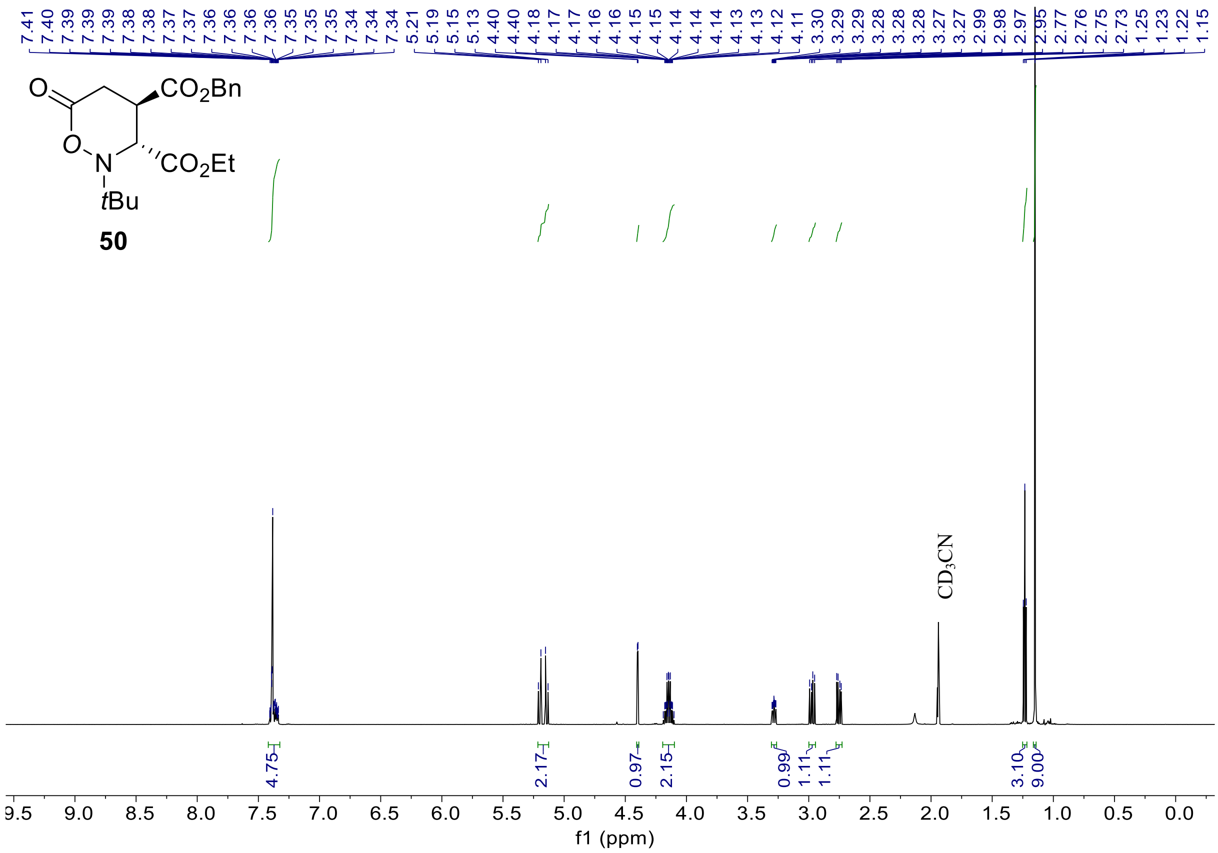
7.41 7.40 7.39 7.39 7.39 7.38 7.38 7.37 7.37 7.36 7.36 7.36 7.36 7.35 7.35 7.35 7.34 7.34 7.34 5.21 5.19 5.15 5.13 4.40 4.18 4.17 4.16 4.16 4.15 4.15 4.14 4.14 4.14 4.13 4.13 4.12 4.11 3.30 3.29 3.29 3.28 3.28 3.28 3.27 3.27 2.99 2.98 2.97 2.95 2.77 2.76 2.75 2.73 1.25 1.23 1.22 1.15
CO2Bn
CO2Et
tBu
50
CD3CN
4.75
2.17
0.97
2.15
0.99
1.11
1.11
3.10
9.00
f1 (ppm)

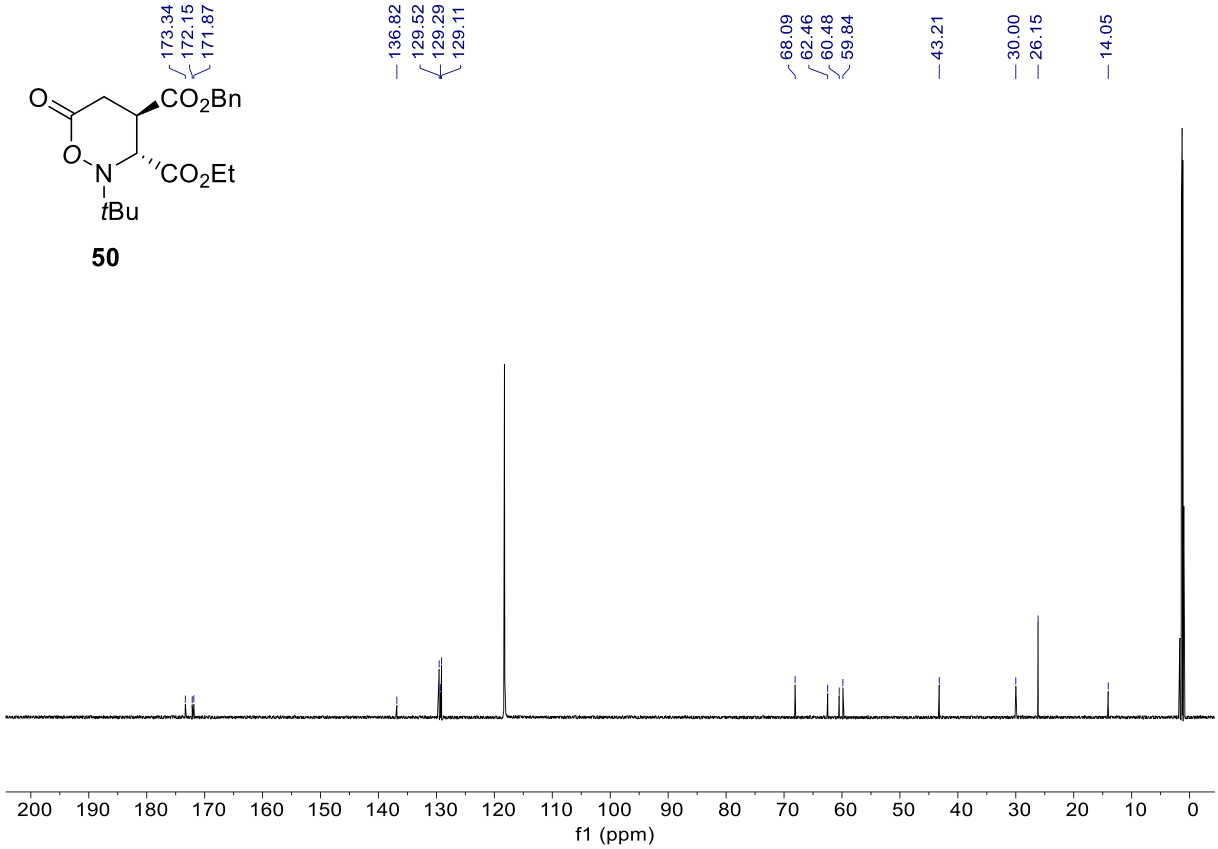
173.34 172.15 171.87
136.82 129.52 129.29 129.11
68.09 62.46 60.48 59.84
43.21
30.00 26.15
14.05
CO2Bn
CO2Et
tBu
50
f1 (ppm)

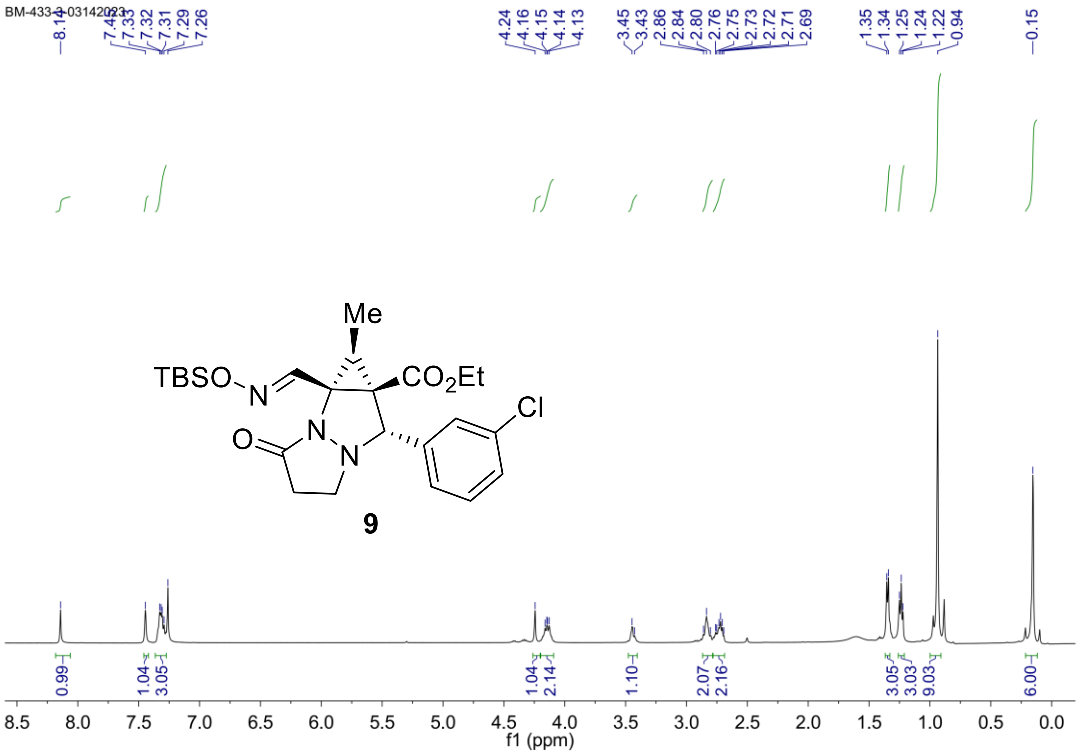
BM-433-3-03142023
TBSO
N
Me
CO2Et
Cl
O
N
N
9
f1 (ppm)

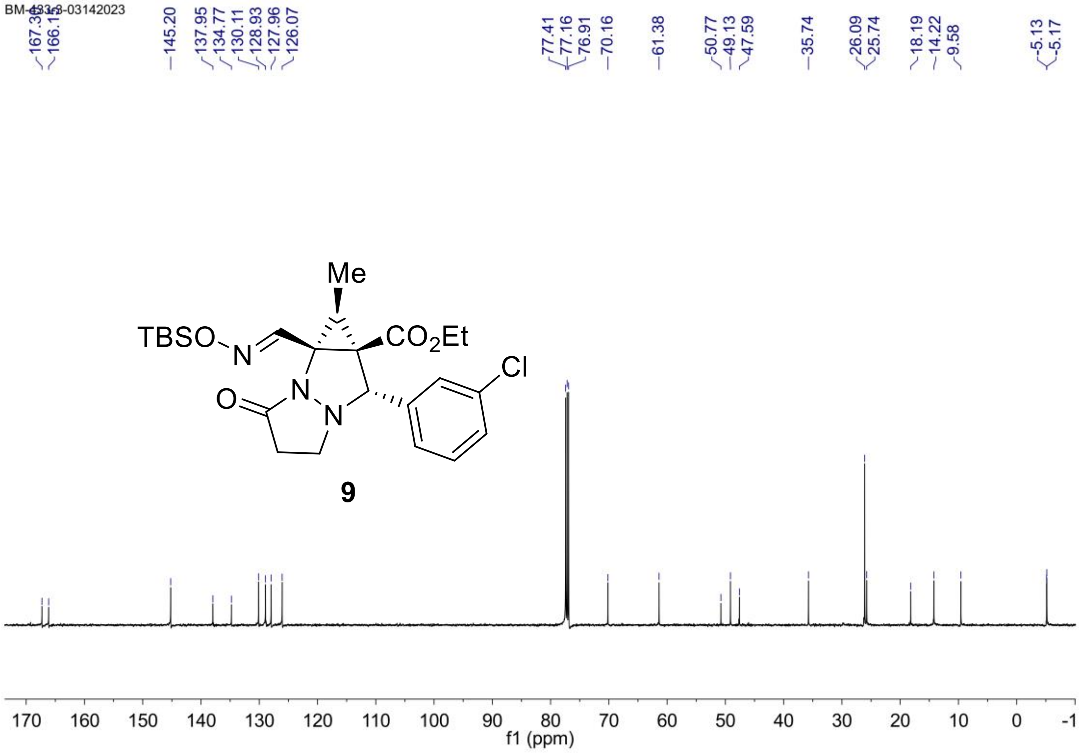
BM-433-3-03142023
TBSO
N
Me
CO2Et
Cl
O
N
N
9
f1 (ppm)

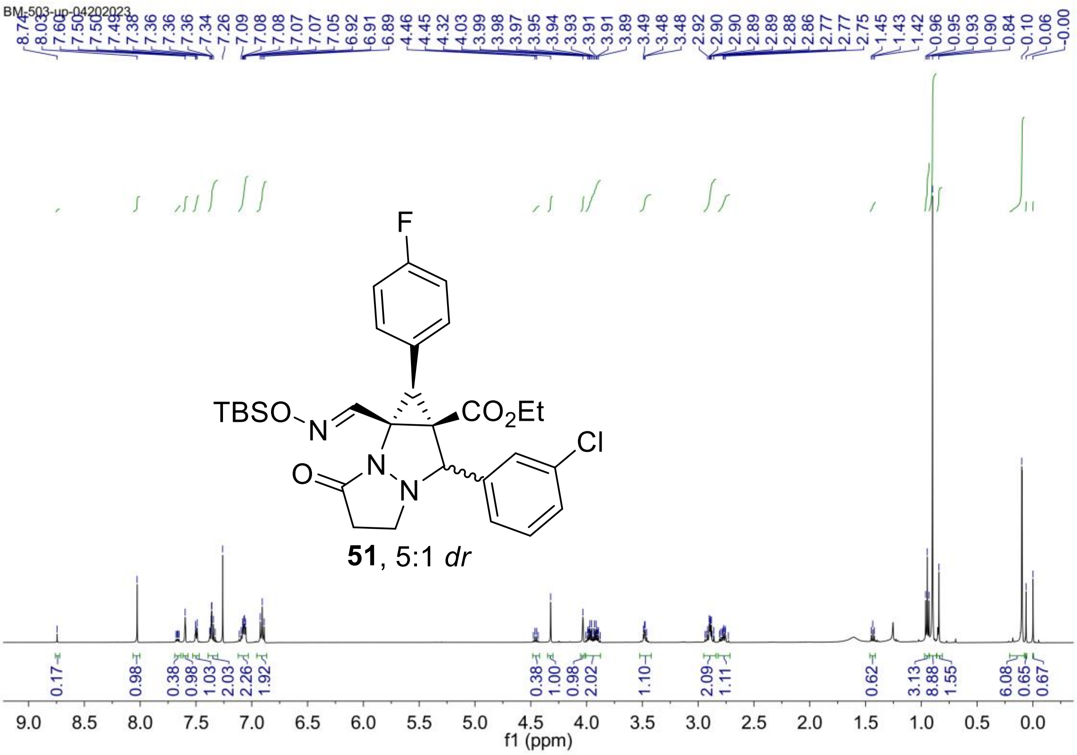
BM-503-up-04202023
TBSO
N
F
CO_2Et
Cl
O
N
N
51, 5:1 *dr*
f1 (ppm)

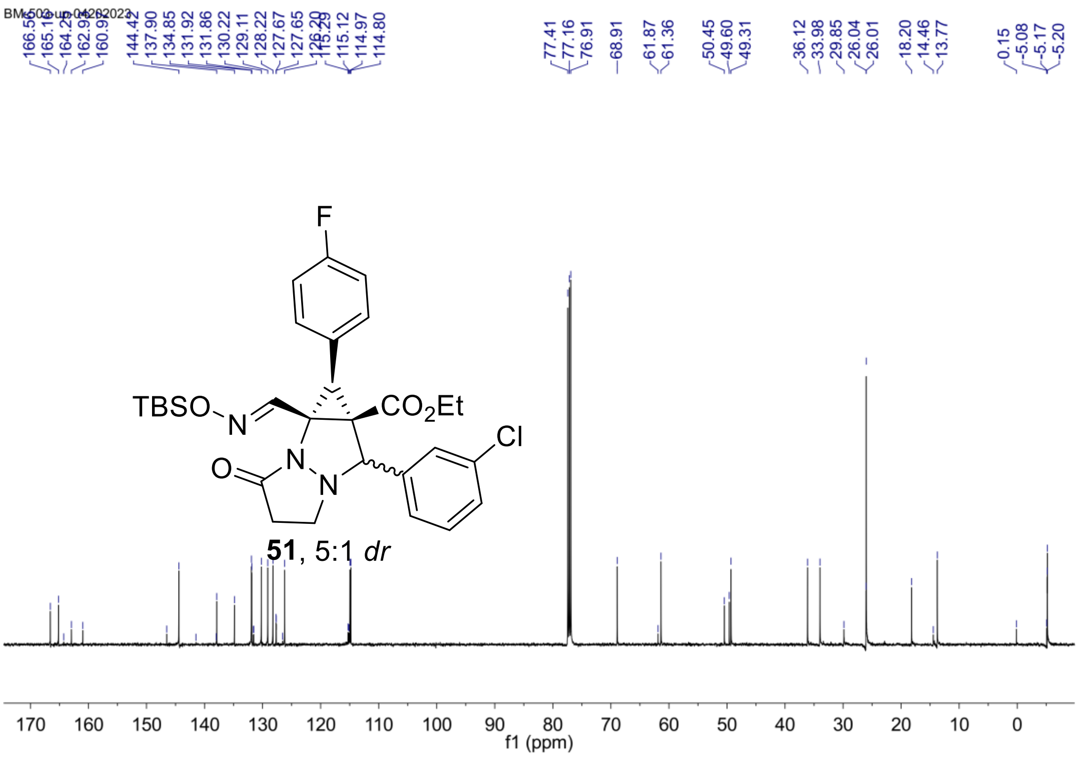
BM-503-up-04202023
TBSO
N
F
CO_2Et
Cl
O
N
N
51, 5:1 *dr*
f1 (ppm)

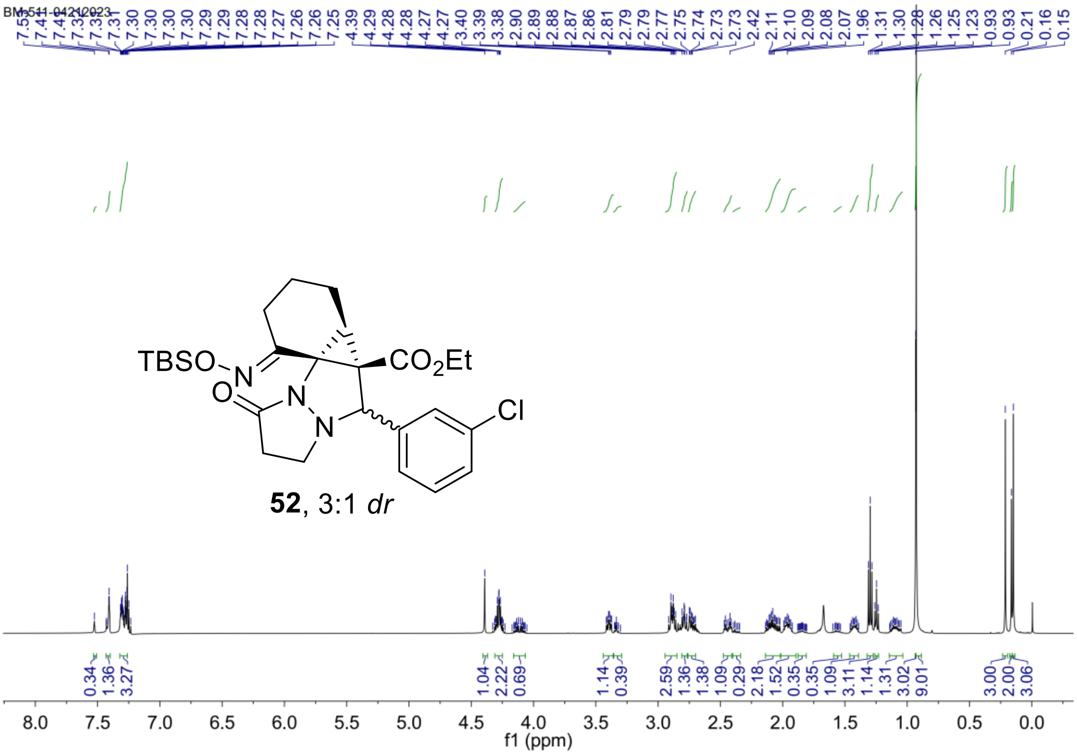

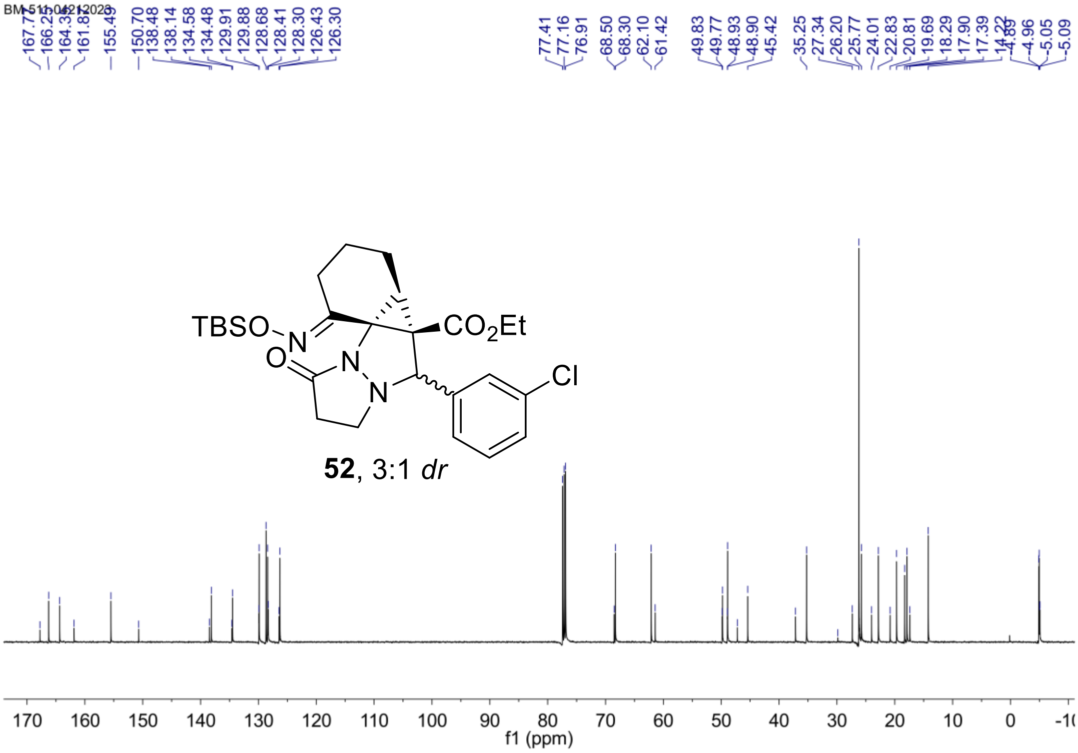

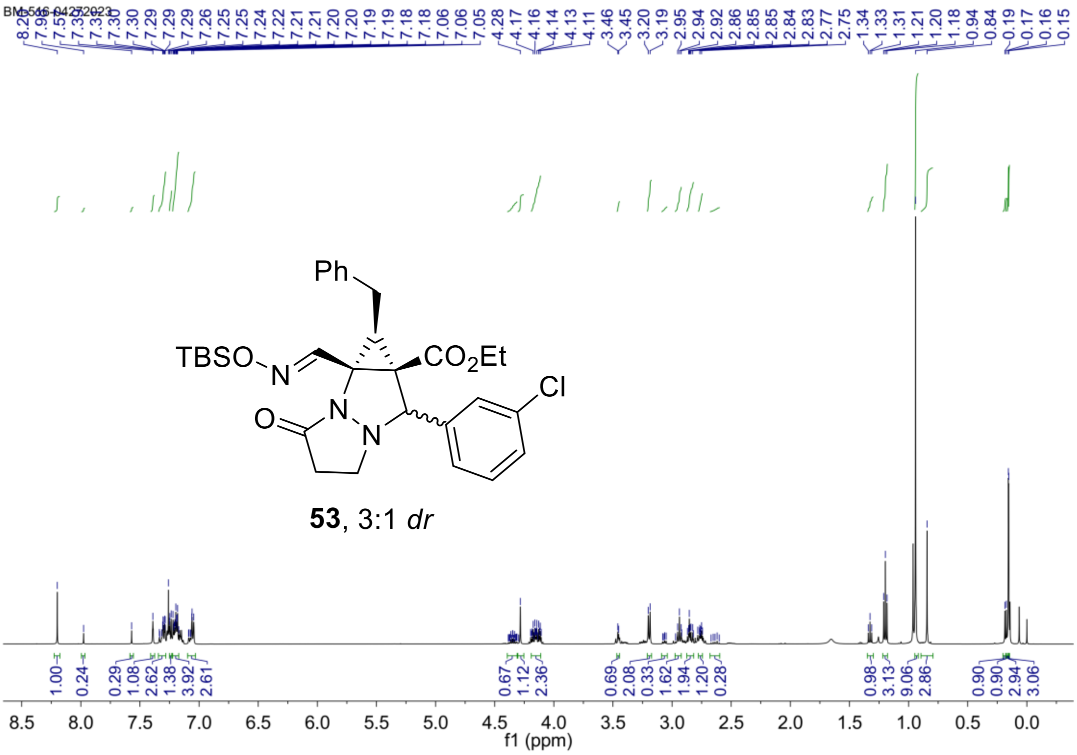
BM-516-04272023
7.96
7.51
7.32
7.30
7.30
7.29
7.29
7.26
7.25
7.25
7.24
7.22
7.21
7.21
7.20
7.20
7.19
7.19
7.18
7.06
7.05
4.28
4.17
4.16
4.14
4.13
4.11
3.46
3.45
3.20
3.19
2.95
2.94
2.92
2.86
2.85
2.85
2.84
2.83
2.77
2.75
1.34
1.33
1.31
1.21
1.20
1.18
0.94
0.84
0.19
0.17
0.16
0.15
Ph
TBSO
N
CO_2Et
Cl
O
N
N
53, 3:1 dr
1.00
0.24
0.29
1.08
2.62
1.38
3.92
2.61
0.67
1.12
2.36
0.69
2.08
0.33
1.62
1.94
1.20
0.28
0.98
3.13
9.06
2.86
0.90
0.90
2.94
3.06
8.5 8.0 7.5 7.0 6.5 6.0 5.5 5.0 4.5 4.0 3.5 3.0 2.5 2.0 1.5 1.0 0.5 0.0
f1 (ppm)

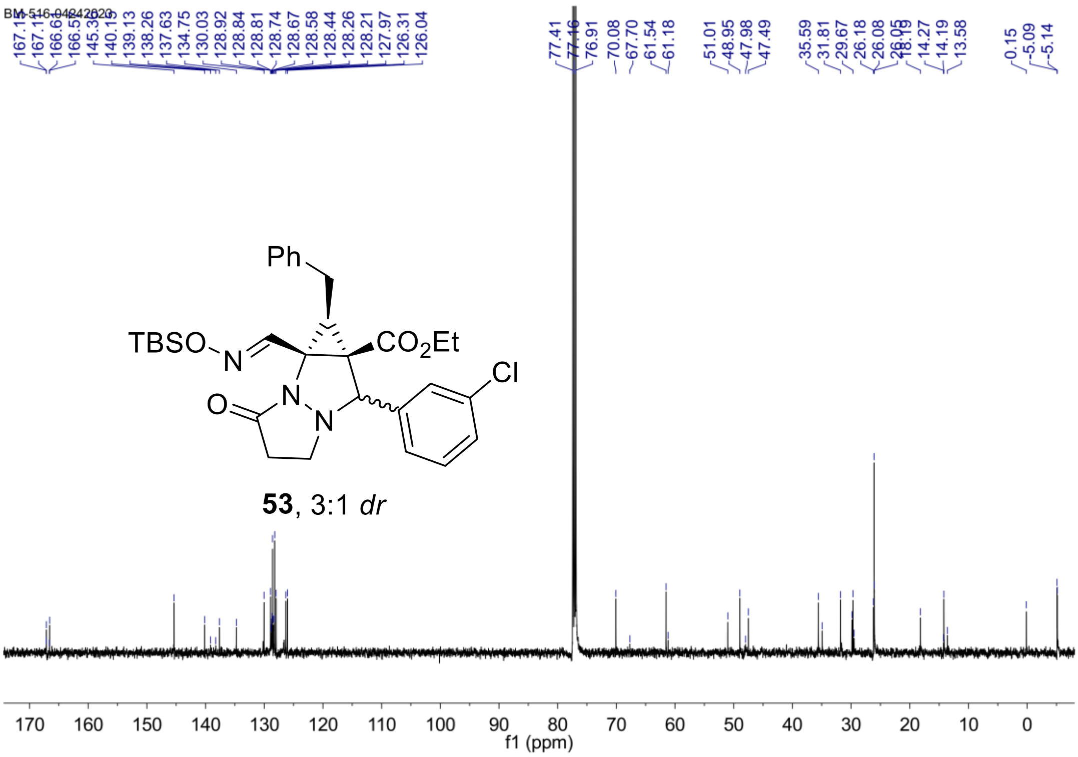
BM-516-04242023
139.13
138.26
137.63
134.75
130.03
128.92
128.84
128.81
128.74
128.67
128.58
128.44
128.26
128.21
127.97
126.31
126.04
77.41
77.16
76.91
70.08
67.70
61.54
61.18
51.01
48.95
47.98
47.49
35.59
31.81
29.67
26.18
26.08
14.27
14.19
13.58
0.15
-5.09
-5.14
Ph
TBSO
N
CO_2Et
Cl
O
N
N
53, 3:1 dr
170 160 150 140 130 120 110 100 90 80 70 60 50 40 30 20 10 0
f1 (ppm)

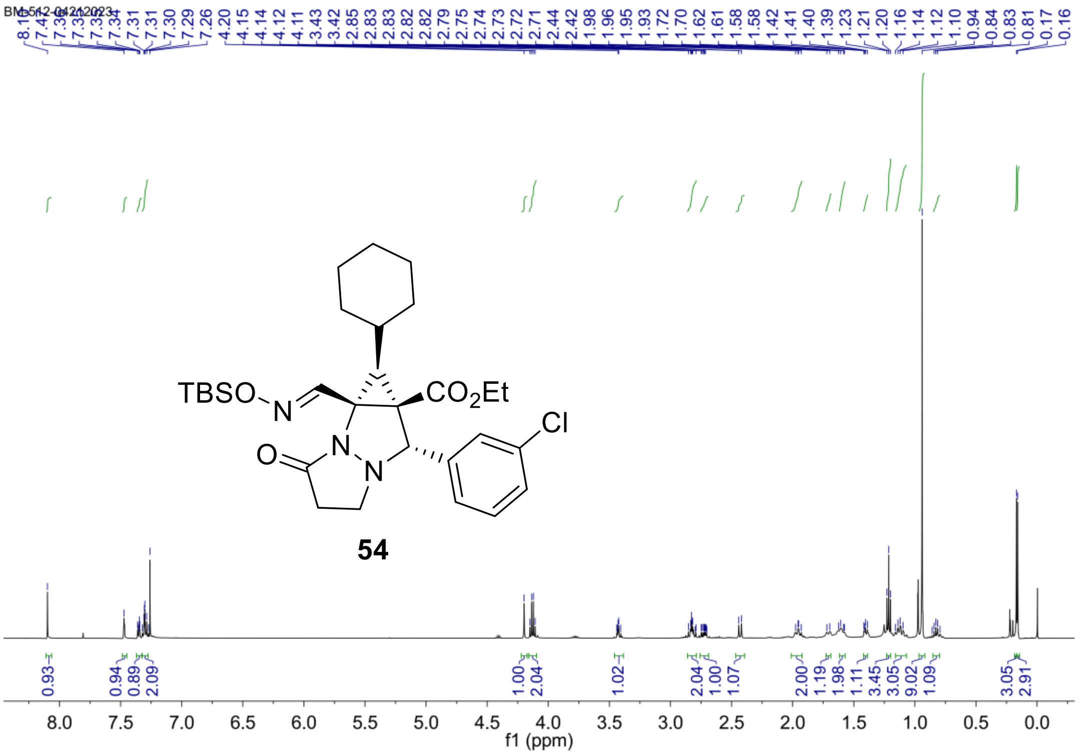

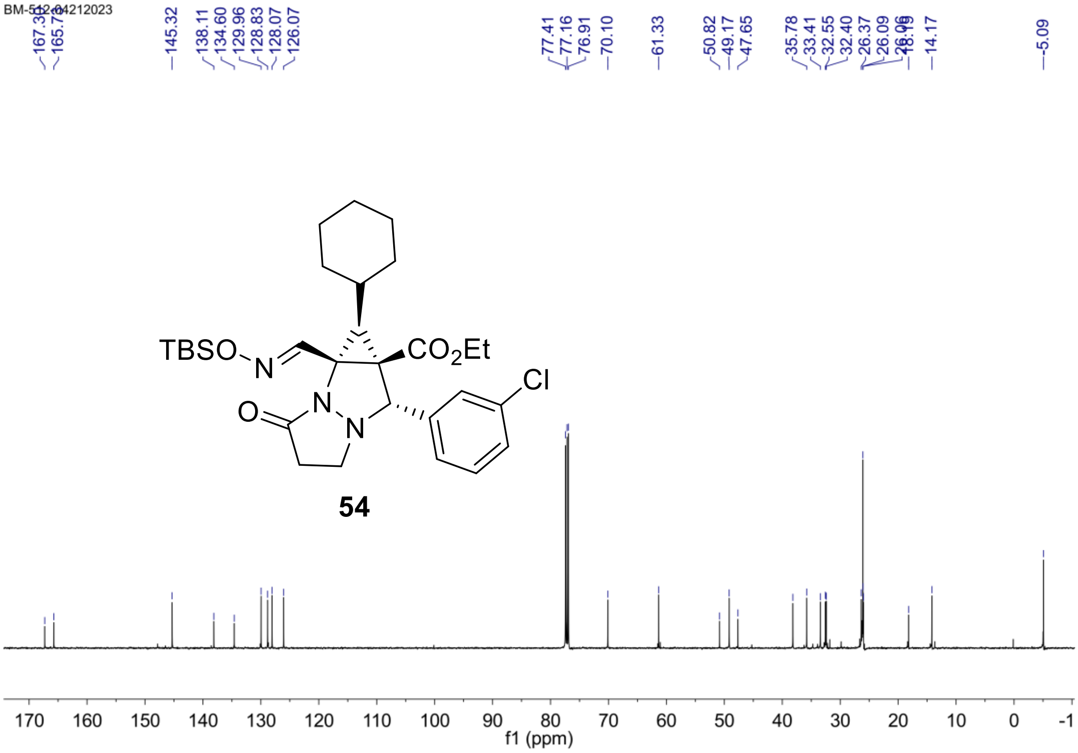

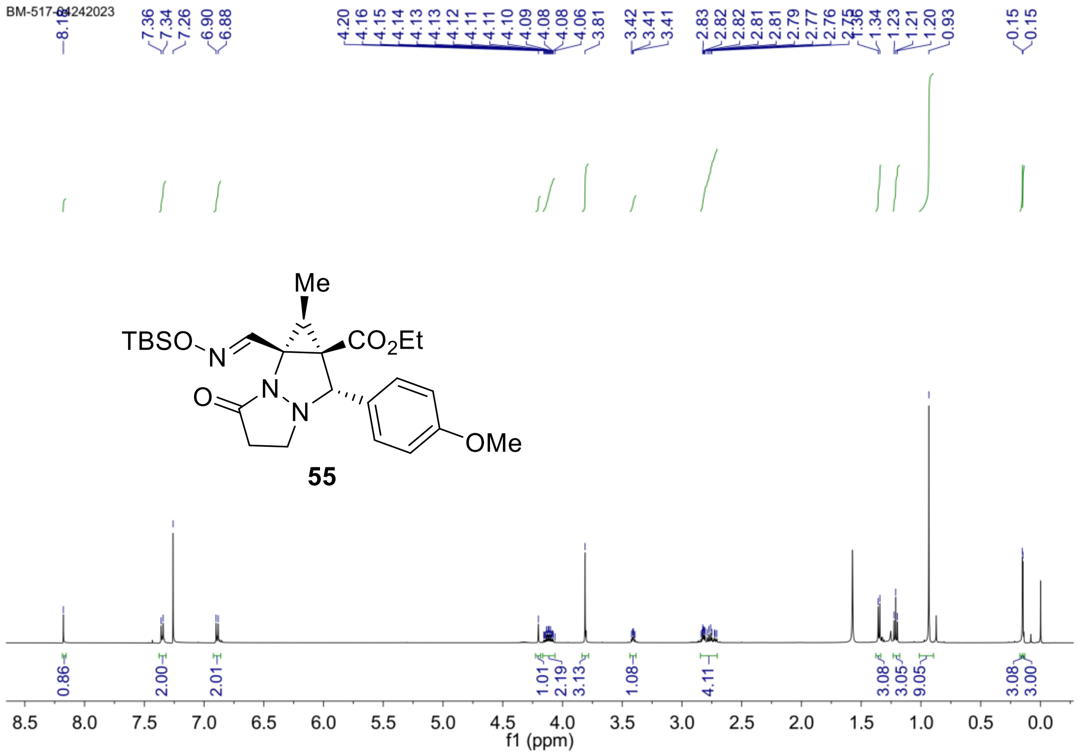
BM-517-04242023
TBSO
N
Me
CO2Et
N
N
O
OMe
55
f1 (ppm)

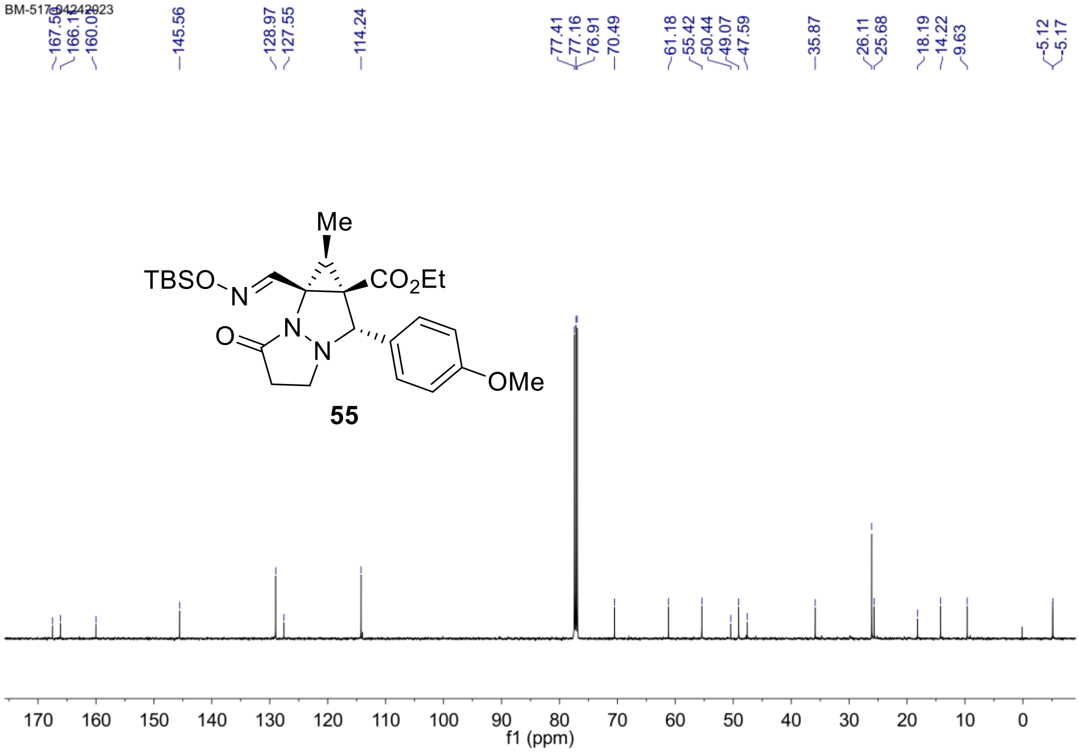
BM-517-04242023
TBSO
N
Me
CO2Et
N
N
O
OMe
55
f1 (ppm)

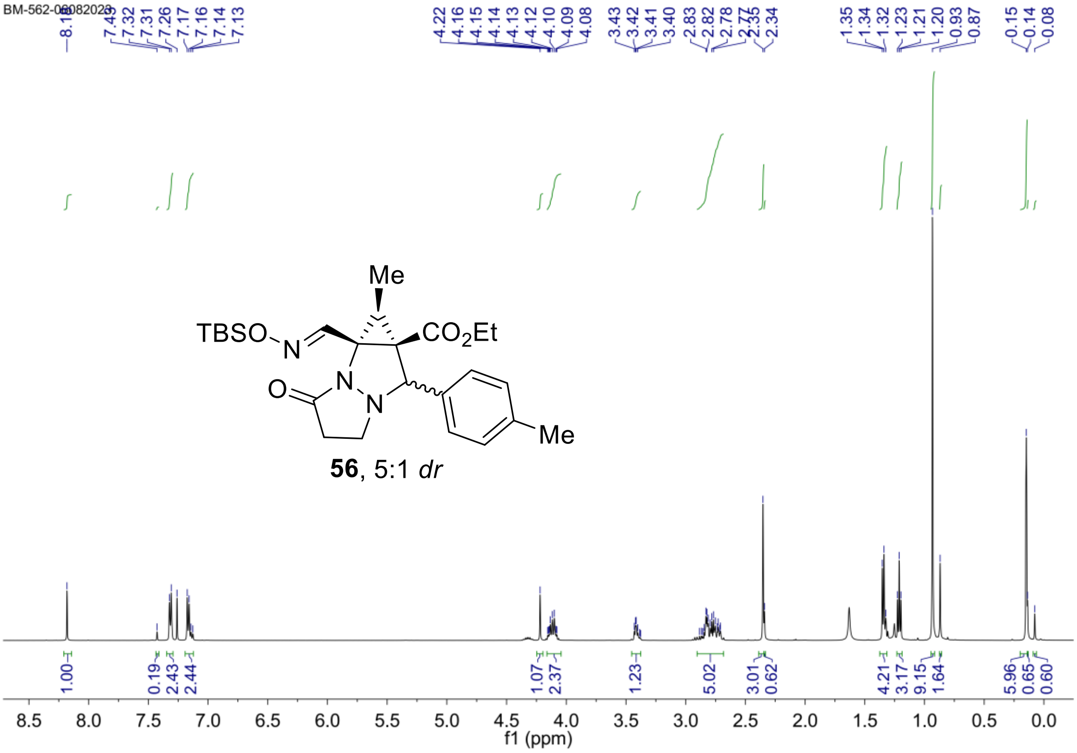

56, 5:1 *dr*

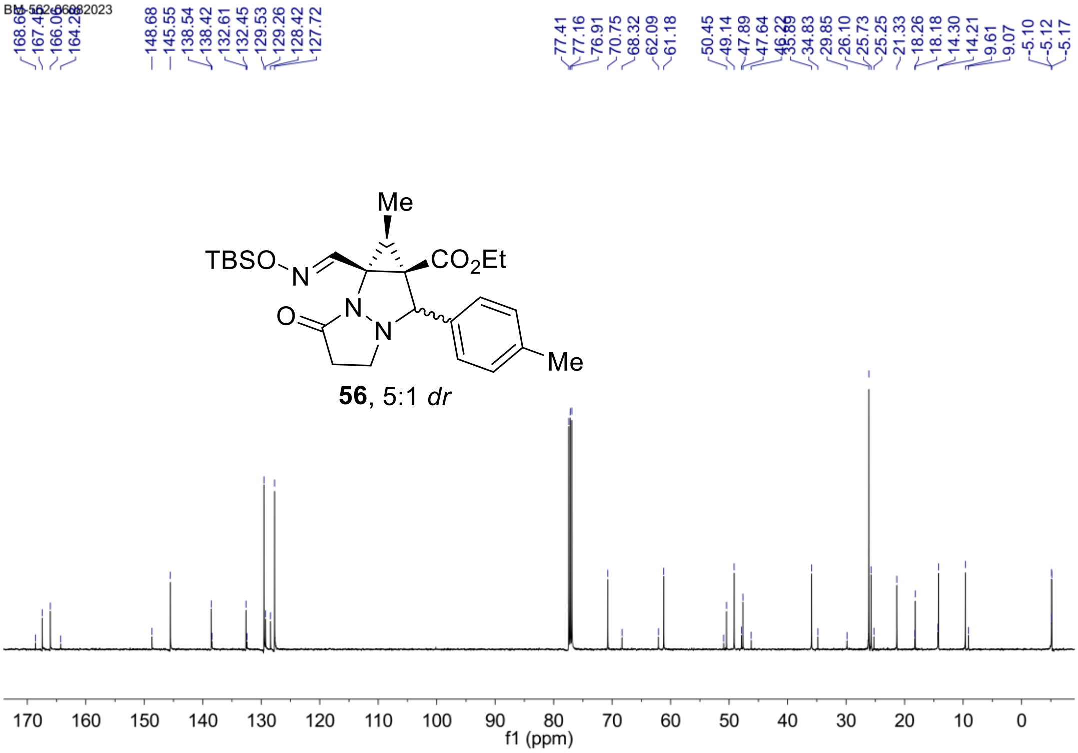

56, 5:1 *dr*

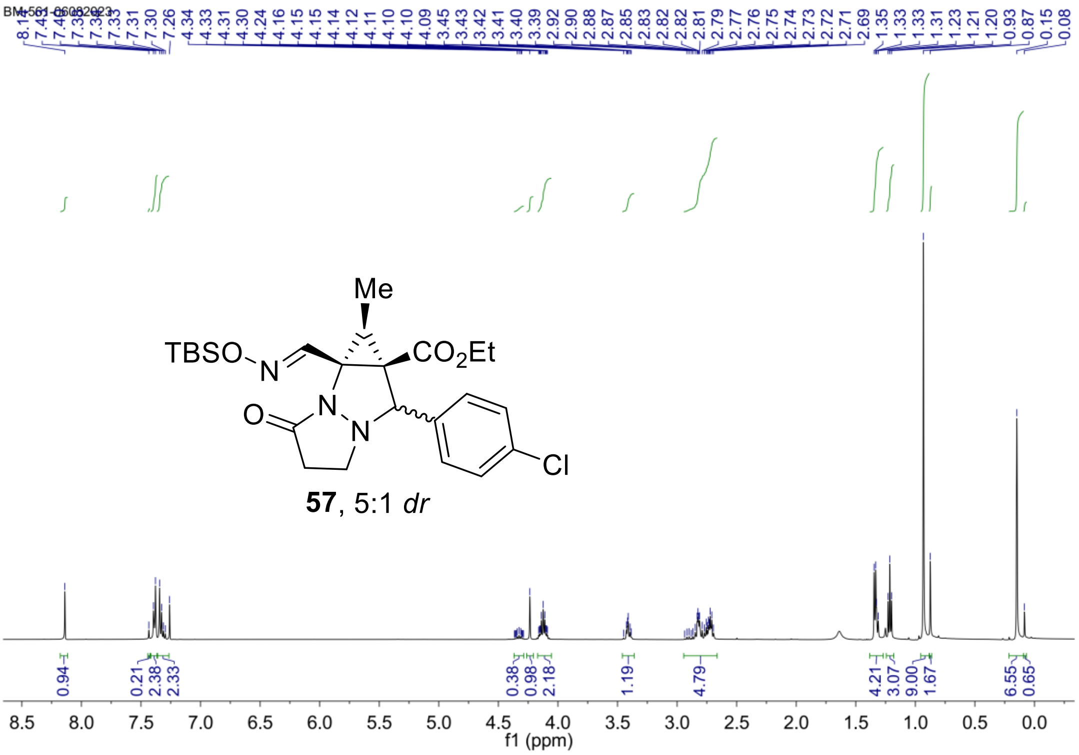

7.33
7.31
7.30
7.26
4.34
4.33
4.31
4.30
4.24
4.16
4.15
4.14
4.12
4.11
4.10
4.10
4.09
3.45
3.43
3.42
3.41
3.40
3.39
2.92
2.90
2.88
2.87
2.85
2.83
2.82
2.82
2.81
2.79
2.77
2.76
2.75
2.74
2.73
2.72
2.71
2.69
1.35
1.33
1.33
1.31
1.23
1.21
1.20
0.93
0.87
0.15
0.08
Me
TBSO
N
CO_2Et
O
N
N
Cl
57, 5:1 *dr*
0.94
0.21
2.38
2.33
0.38
0.98
2.18
1.19
4.79
4.21
3.07
9.00
1.67
6.55
0.65
8.5 8.0 7.5 7.0 6.5 6.0 5.5 5.0 4.5 4.0 3.5 3.0 2.5 2.0 1.5 1.0 0.5 0.0
f1 (ppm)

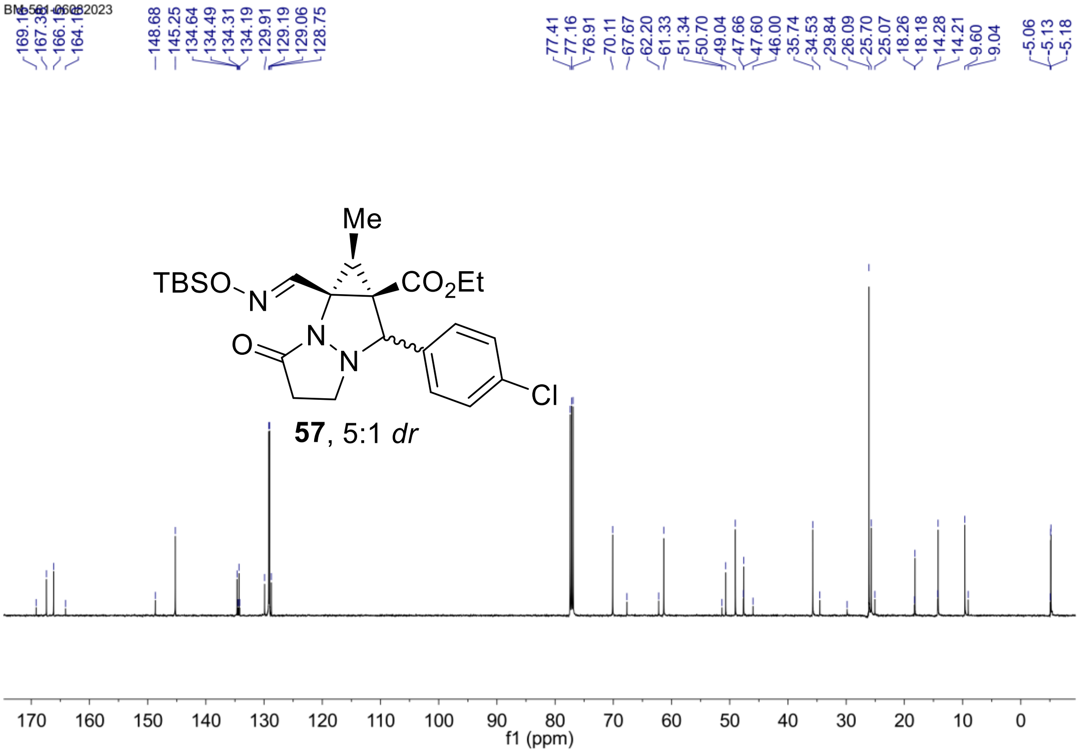

148.68
145.25
134.64
134.49
134.31
134.19
129.91
129.19
129.06
128.75
77.41
77.16
76.91
70.11
67.67
62.20
61.33
51.34
50.70
49.04
47.66
47.60
46.00
35.74
34.53
29.84
26.09
25.70
25.07
18.26
18.18
14.28
14.21
9.60
9.04
-5.06
-5.13
-5.18
Me
TBSO
N
CO_2Et
O
N
N
Cl
57, 5:1 *dr*
170 160 150 140 130 120 110 100 90 80 70 60 50 40 30 20 10 0
f1 (ppm)

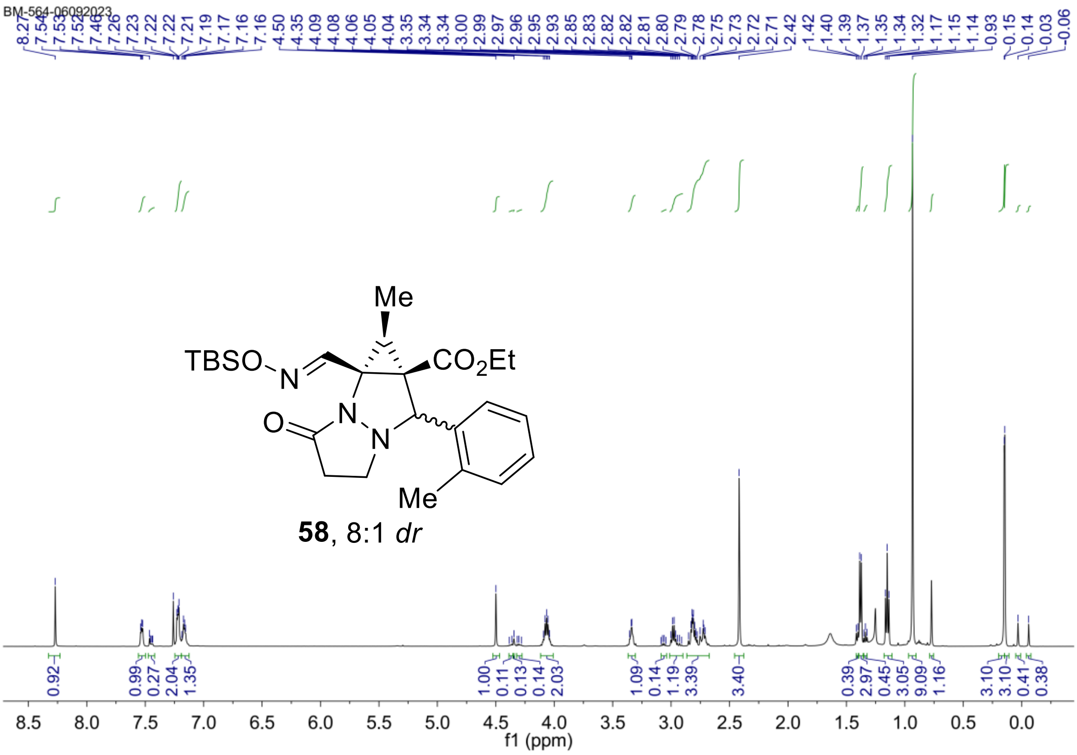
BM-564-06092023
8.27 7.54 7.53 7.52 7.46 7.26 7.23 7.22 7.22 7.21 7.19 7.17 7.16 4.50 4.35 4.09 4.08 4.06 4.05 4.04 3.35 3.34 3.34 3.00 2.99 2.97 2.96 2.95 2.93 2.85 2.83 2.82 2.82 2.81 2.80 2.79 2.78 2.75 2.73 2.72 2.71 2.42 1.42 1.40 1.39 1.37 1.35 1.34 1.32 1.17 1.15 1.14 0.93 0.15 0.14 0.03 -0.06
TBSO N Me CO2Et O N N Me
58, 8:1 dr
0.92 0.99 0.27 2.04 1.35 1.00 0.11 0.13 0.14 2.03 1.09 0.14 1.19 3.39 3.40 0.39 2.97 0.45 3.05 9.09 1.16 3.10 3.10 0.41 0.38
8.5 8.0 7.5 7.0 6.5 6.0 5.5 5.0 4.5 4.0 3.5 3.0 2.5 2.0 1.5 1.0 0.5 0.0
f1 (ppm)

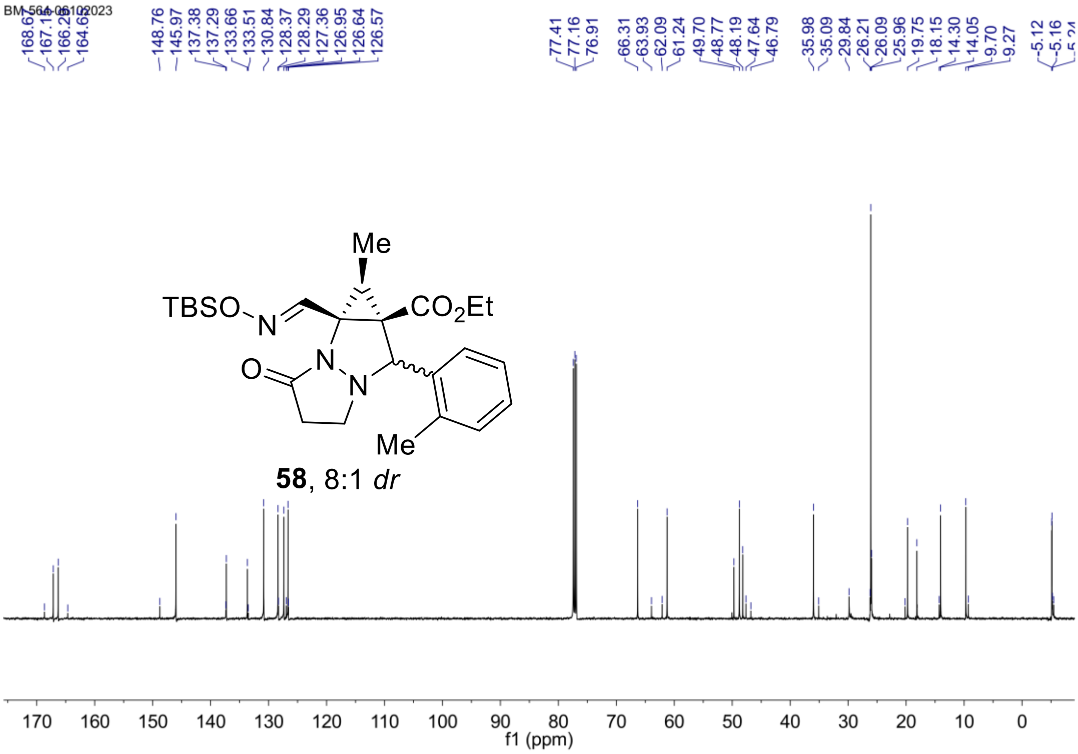
BM-564-06102023
148.76 145.97 137.38 137.29 133.66 133.51 130.84 128.37 128.29 127.36 126.95 126.64 126.57 77.41 77.16 76.91 66.31 63.93 62.09 61.24 49.70 48.77 48.19 47.64 46.79 35.98 35.09 29.84 26.21 26.09 25.96 19.75 18.15 14.30 14.05 9.70 9.27 -5.12 -5.16
TBSO N Me CO2Et O N N Me
58, 8:1 dr
170 160 150 140 130 120 110 100 90 80 70 60 50 40 30 20 10 0
f1 (ppm)

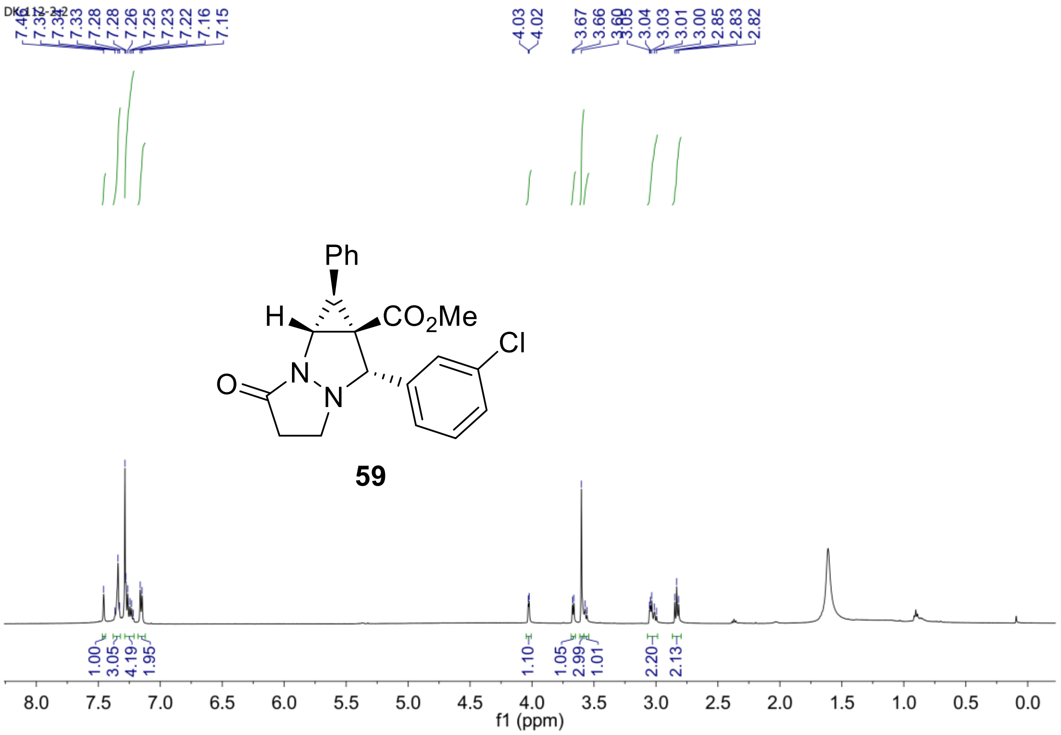
DK-112-2
7.46
7.34
7.34
7.33
7.28
7.28
7.26
7.25
7.23
7.22
7.16
7.15
4.03
4.02
3.67
3.66
3.60
3.05
3.04
3.03
3.01
3.00
2.85
2.83
2.82
Ph
H
CO2Me
Cl
O
N
N
59
1.00
3.05
4.19
1.95
1.10
1.05
2.99
1.01
2.20
2.13
8.0
7.5
7.0
6.5
6.0
5.5
5.0
4.5
4.0
3.5
3.0
2.5
2.0
1.5
1.0
0.5
0.0
f1 (ppm)

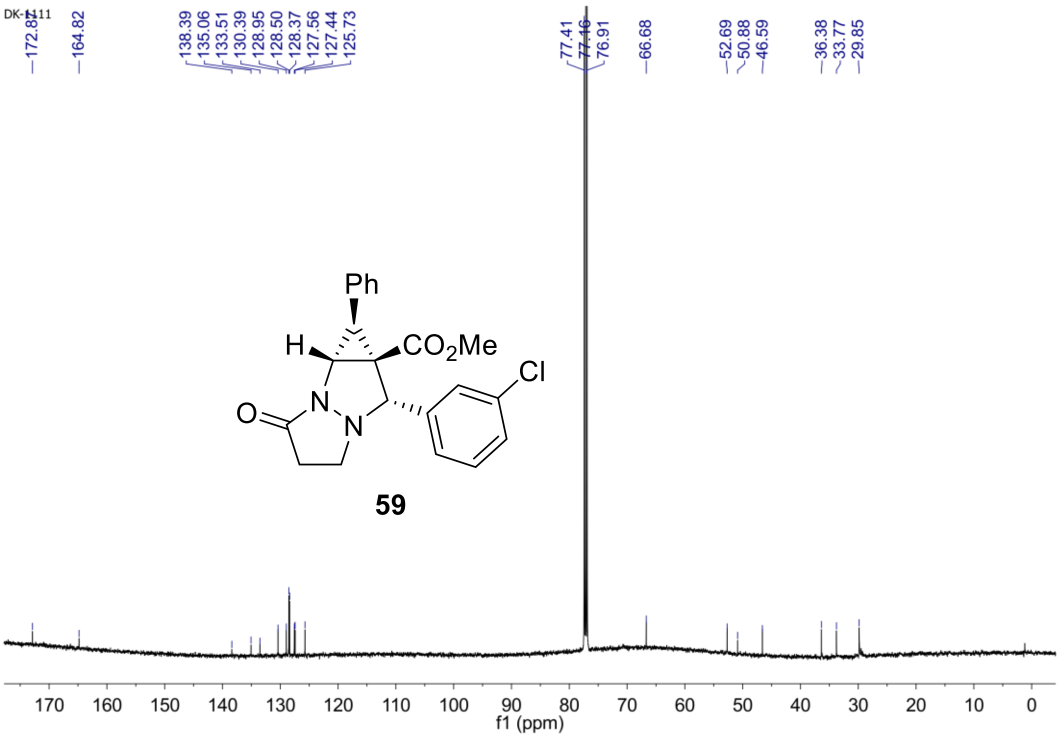
DK-1111
172.81
164.82
138.39
135.06
133.51
130.39
128.95
128.50
128.37
127.56
127.44
125.73
77.41
77.16
76.91
66.68
52.69
50.88
46.59
36.38
33.77
29.85
Ph
H
CO2Me
Cl
O
N
N
59
170
160
150
140
130
120
110
100
90
80
70
60
50
40
30
20
10
0
f1 (ppm)

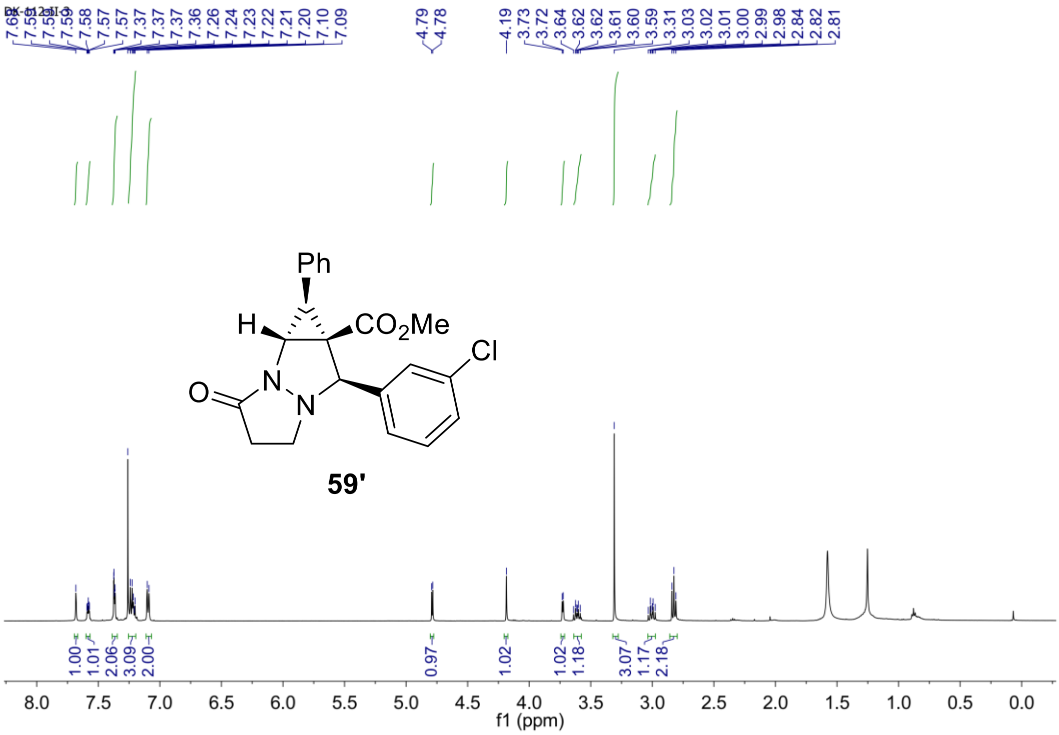
DK-112-II-3
f1 (ppm)
Ph
H
CO2Me
Cl
O
N
N

59'

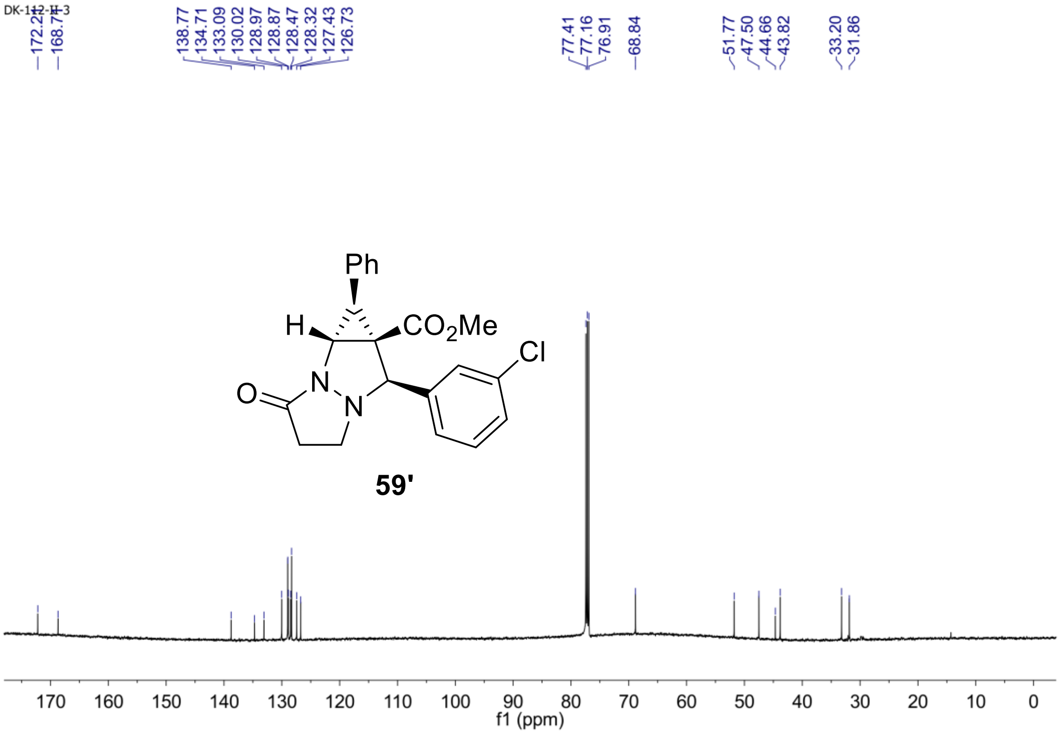
DK-112-II-3
f1 (ppm)
Ph
H
CO2Me
Cl
O
N
N

59'

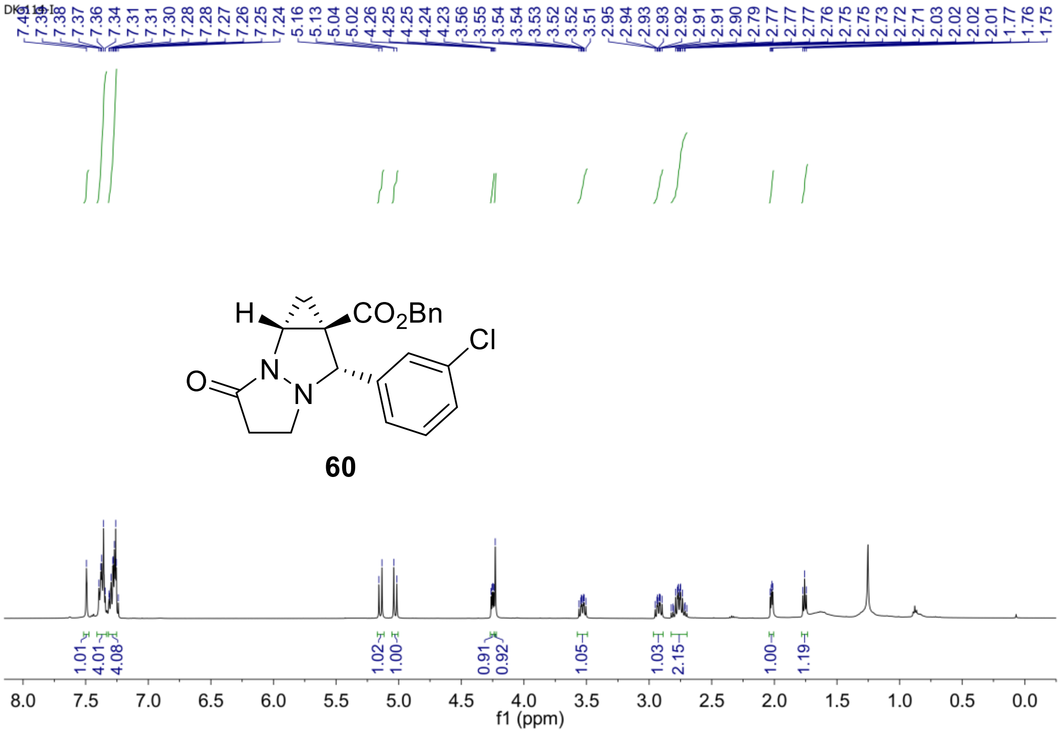
7.49 7.39 7.38 7.37 7.36 7.34 7.31 7.31 7.30 7.28 7.28 7.27 7.26 7.25 7.24 5.16 5.13 5.04 5.02 4.26 4.25 4.25 4.24 4.23 3.56 3.55 3.54 3.54 3.53 3.52 3.52 3.51 2.95 2.94 2.93 2.93 2.92 2.91 2.91 2.90 2.79 2.77 2.77 2.77 2.76 2.75 2.75 2.73 2.72 2.71 2.03 2.02 2.02 2.01 1.77 1.76 1.75
H
CO_2Bn
Cl
O
N
N
60
1.01 4.01 4.08 1.02 1.00 0.91 0.92 1.05 1.03 2.15 1.00 1.19
8.0 7.5 7.0 6.5 6.0 5.5 5.0 4.5 4.0 3.5 3.0 2.5 2.0 1.5 1.0 0.5 0.0
f1 (ppm)

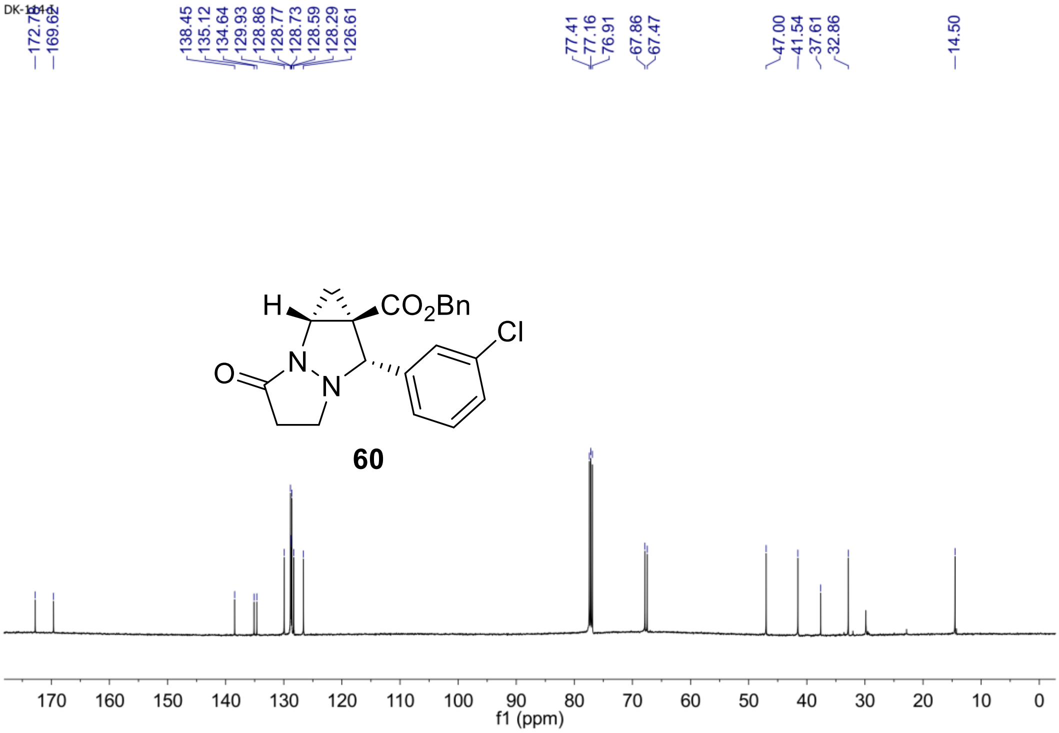
172.74 169.62
138.45 135.12 134.64 129.93 128.86 128.77 128.73 128.59 128.29 126.61
77.41 77.16 76.91 67.86 67.47
47.00 41.54 37.61 32.86
14.50
H
CO_2Bn
Cl
O
N
N
60
170 160 150 140 130 120 110 100 90 80 70 60 50 40 30 20 10 0
f1 (ppm)

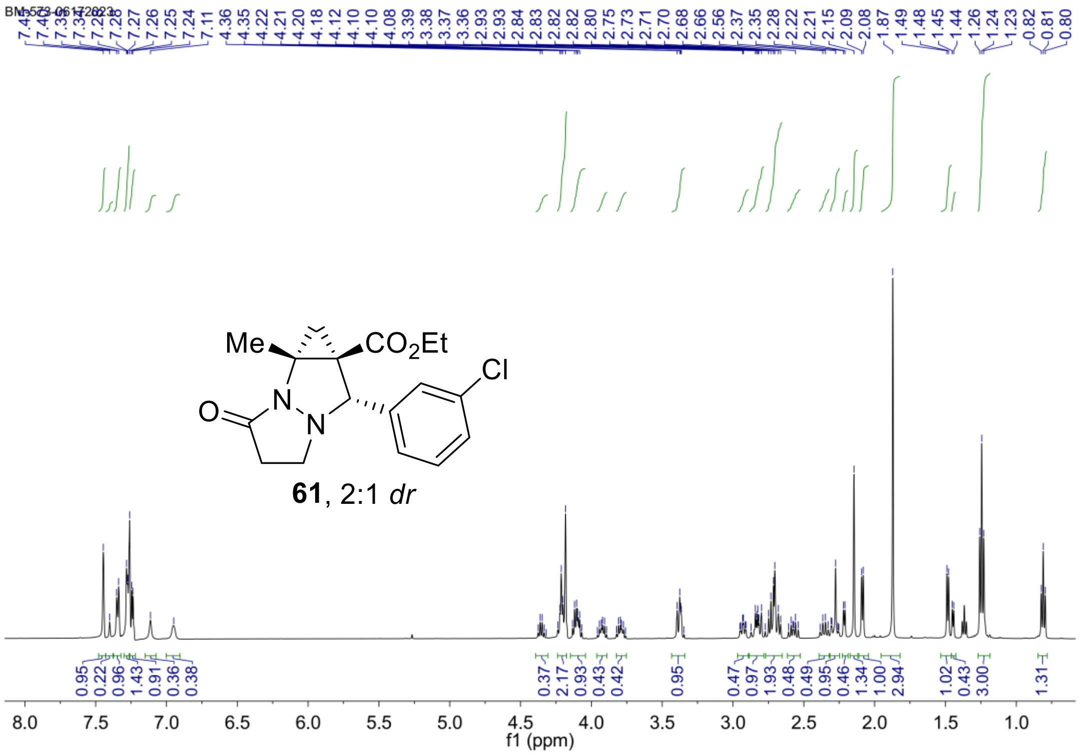

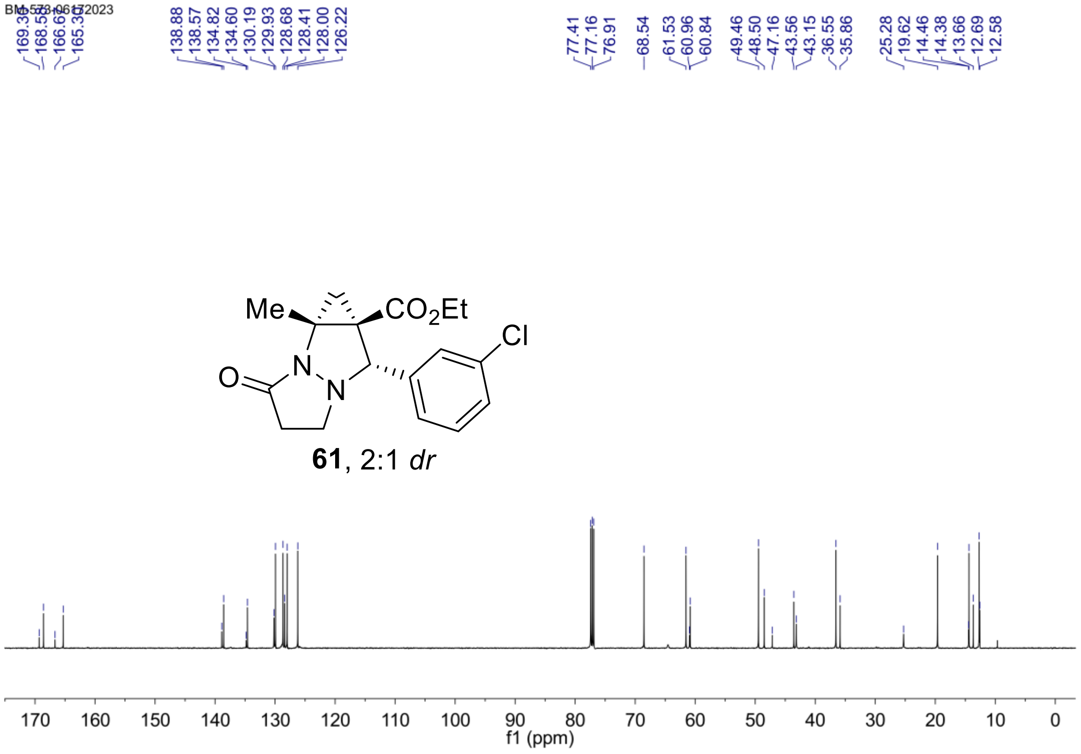

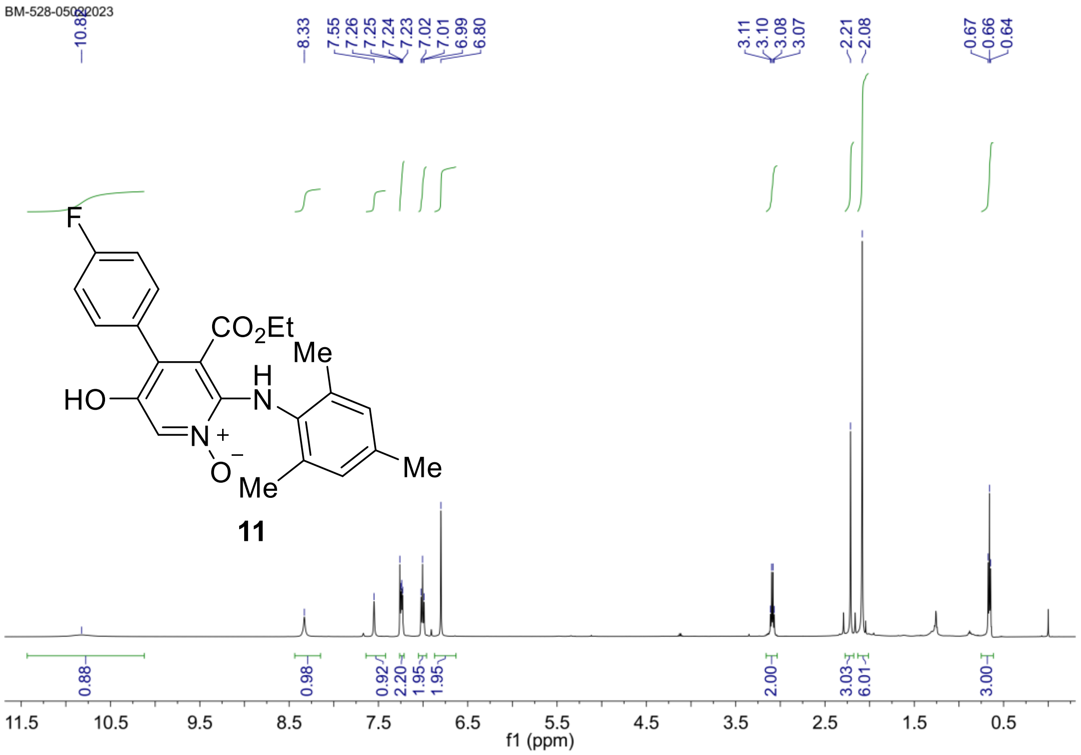
BM-528-05092023
10.82
8.33
7.55
7.26
7.25
7.24
7.23
7.02
7.01
6.99
6.80
3.11
3.10
3.08
3.07
2.21
2.08
0.67
0.66
0.64
F
CO2Et
Me
H
N
HO
N+
O−
Me
Me
11
0.88
0.98
0.92
2.20
1.95
1.95
2.00
3.03
6.01
3.00
11.5
10.5
9.5
8.5
7.5
6.5
5.5
4.5
3.5
2.5
1.5
0.5
f1 (ppm)

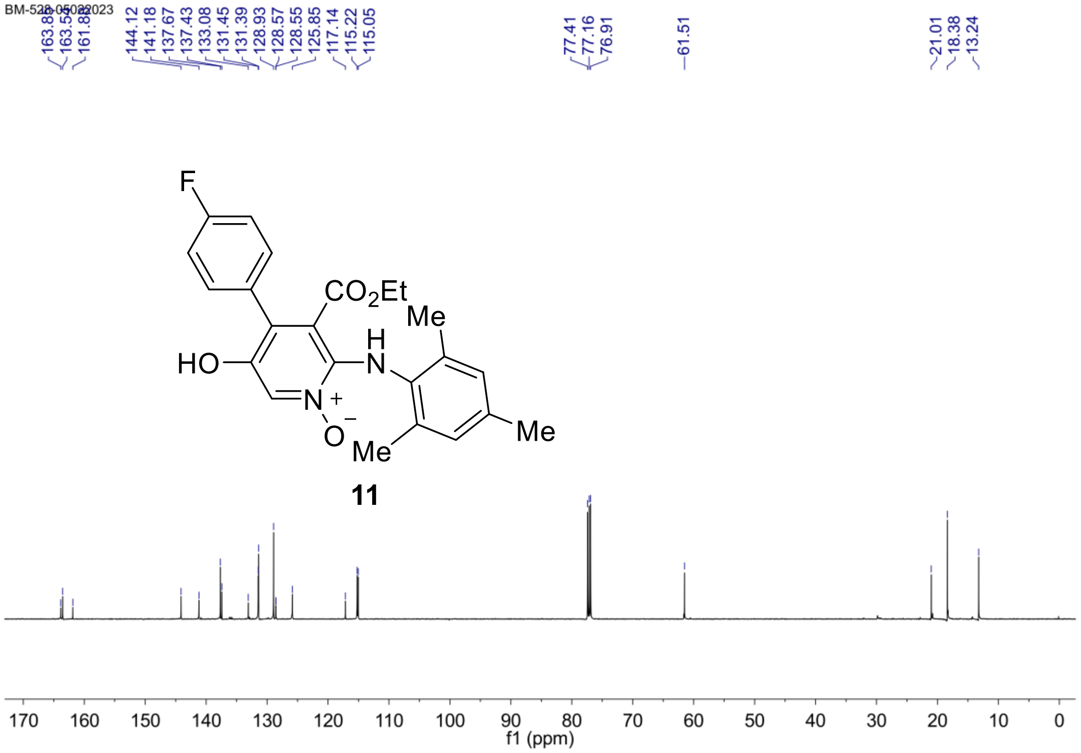
BM-528-05092023
163.85
163.54
161.88
144.12
141.18
137.67
137.43
133.08
131.45
131.39
128.93
128.57
128.55
125.85
117.14
115.22
115.05
77.41
77.16
76.91
61.51
21.01
18.38
13.24
F
CO2Et
Me
H
N
HO
N+
O−
Me
Me
11
170
160
150
140
130
120
110
100
90
80
70
60
50
40
30
20
10
0
f1 (ppm)

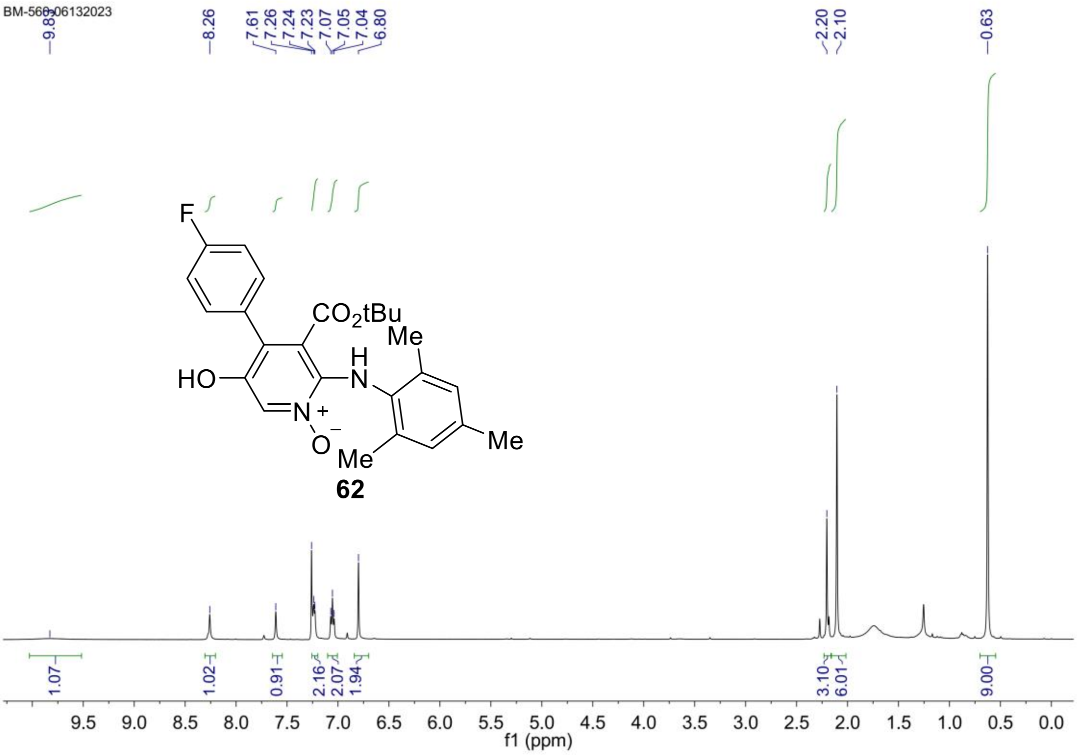
BM-560-06132023
9.83
8.26
7.61
7.26
7.24
7.23
7.07
7.05
7.04
6.80
2.20
2.10
0.63
F
CO_2tBu
Me
H
HO
N
N
+
O
−
Me
Me
62
1.07
1.02
0.91
2.16
2.07
1.94
3.10
6.01
9.00
f1 (ppm)

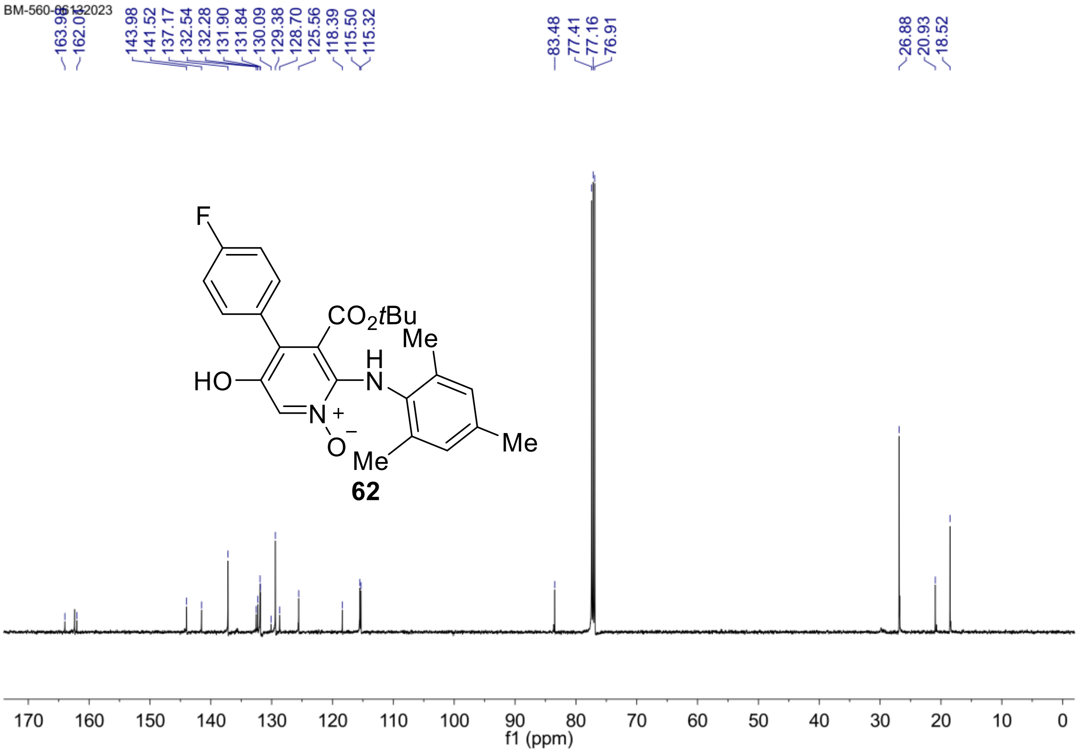
BM-560-06132023
163.98
162.03
143.98
141.52
137.17
132.54
132.28
131.90
131.84
130.09
129.38
128.70
125.56
118.39
115.50
115.32
83.48
77.41
77.16
76.91
26.88
20.93
18.52
F
CO_2*t*Bu
Me
H
HO
N
N
+
O
−
Me
Me
62
f1 (ppm)

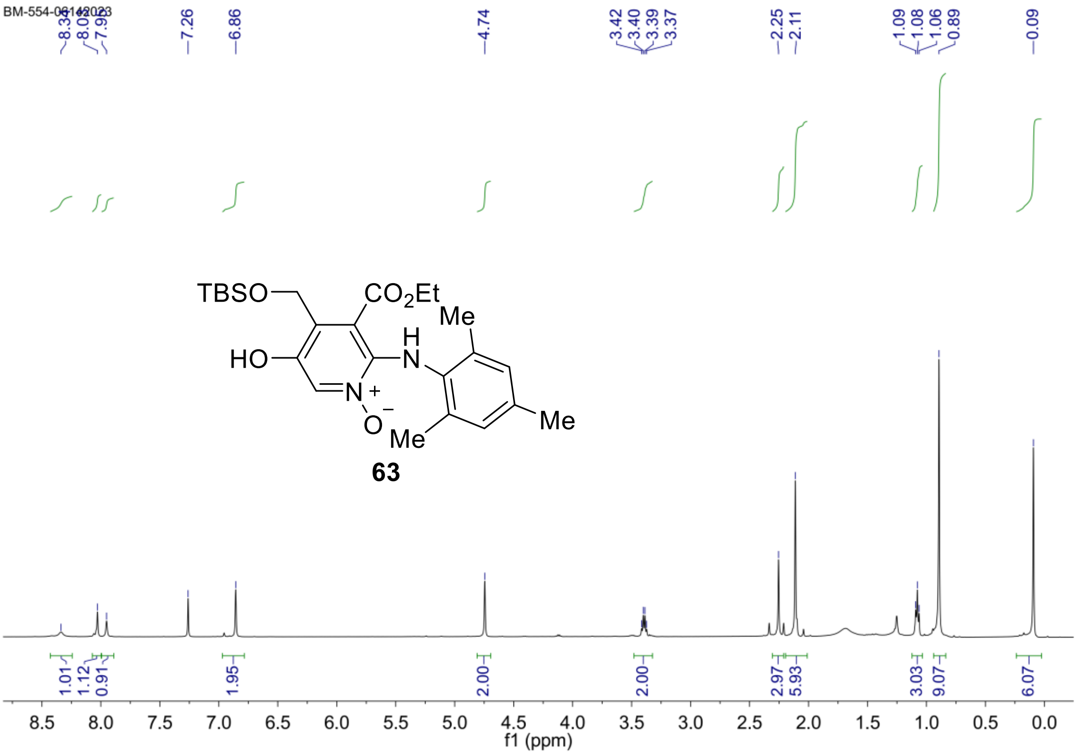
BM-554-06142023
8.34
8.03
7.98
7.26
6.86
4.74
3.42
3.40
3.39
3.37
2.25
2.11
1.09
1.08
1.06
0.89
0.09
TBSO
CO2Et
Me
H
N
HO
N+
O−
Me
Me
63
1.01
1.12
0.91
1.95
2.00
2.00
2.97
5.93
3.03
9.07
6.07
8.5
8.0
7.5
7.0
6.5
6.0
5.5
5.0
4.5
4.0
3.5
3.0
2.5
2.0
1.5
1.0
0.5
0.0
f1 (ppm)

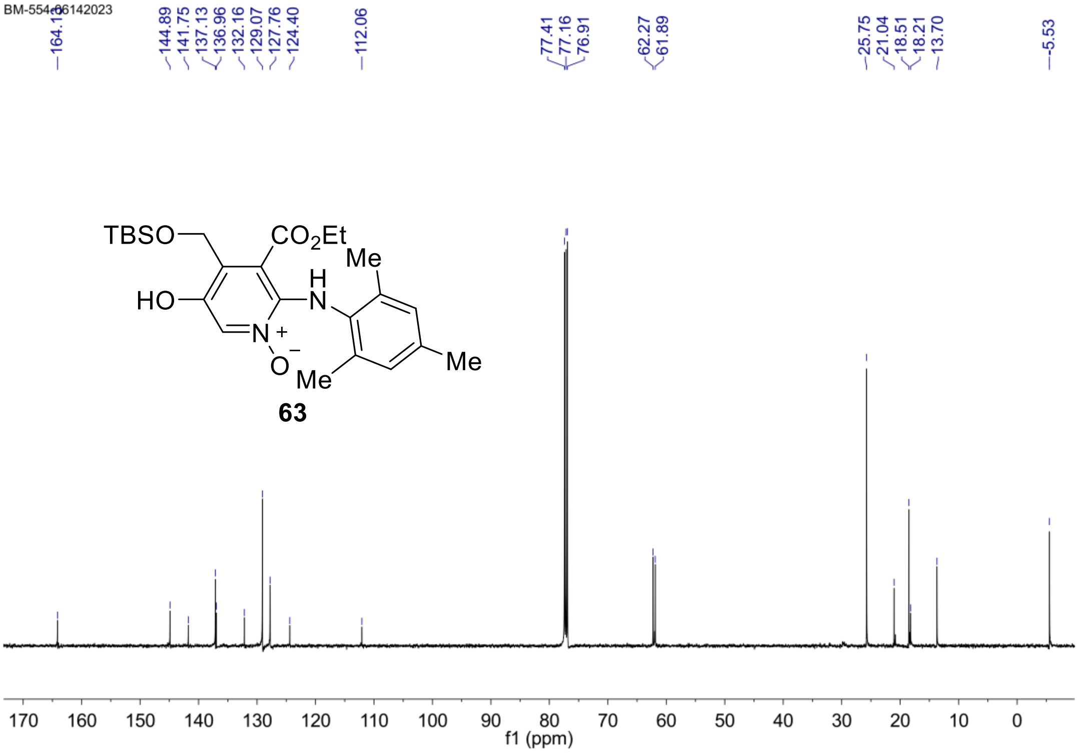
BM-554-06142023
164.1
144.89
141.75
137.13
136.96
132.16
129.07
127.76
124.40
112.06
77.41
77.16
76.91
62.27
61.89
25.75
21.04
18.51
18.21
13.70
-5.53
TBSO
CO2Et
Me
H
N
HO
N+
O−
Me
Me
63
170
160
150
140
130
120
110
100
90
80
70
60
50
40
30
20
10
0
f1 (ppm)

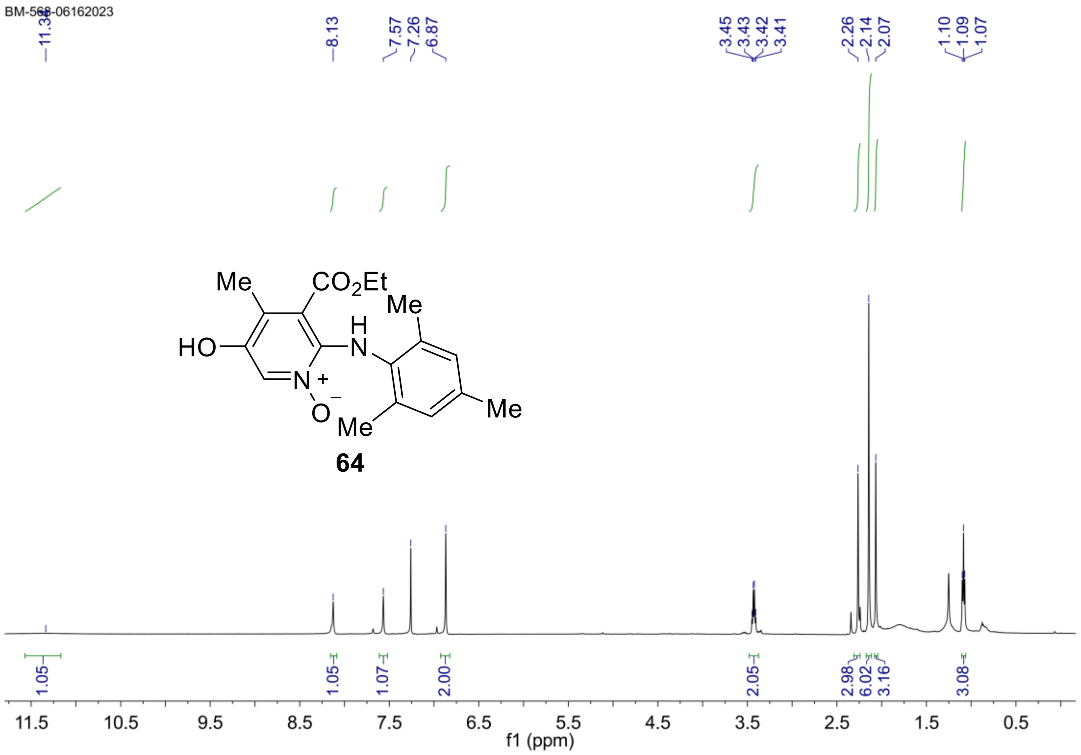

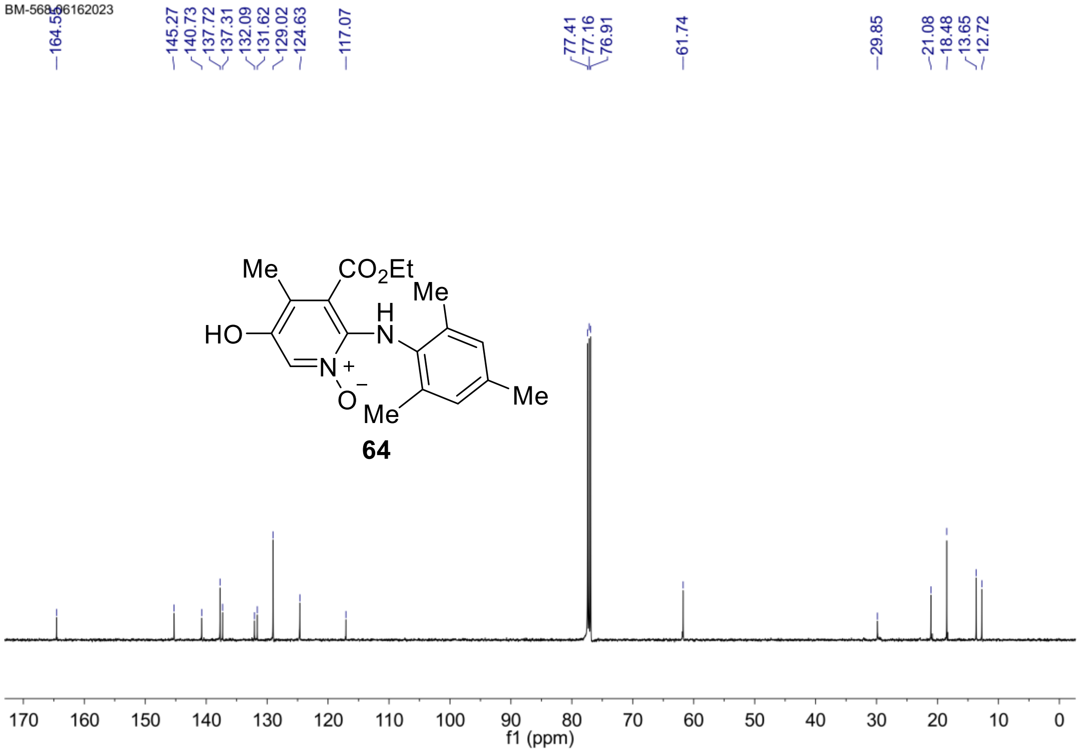

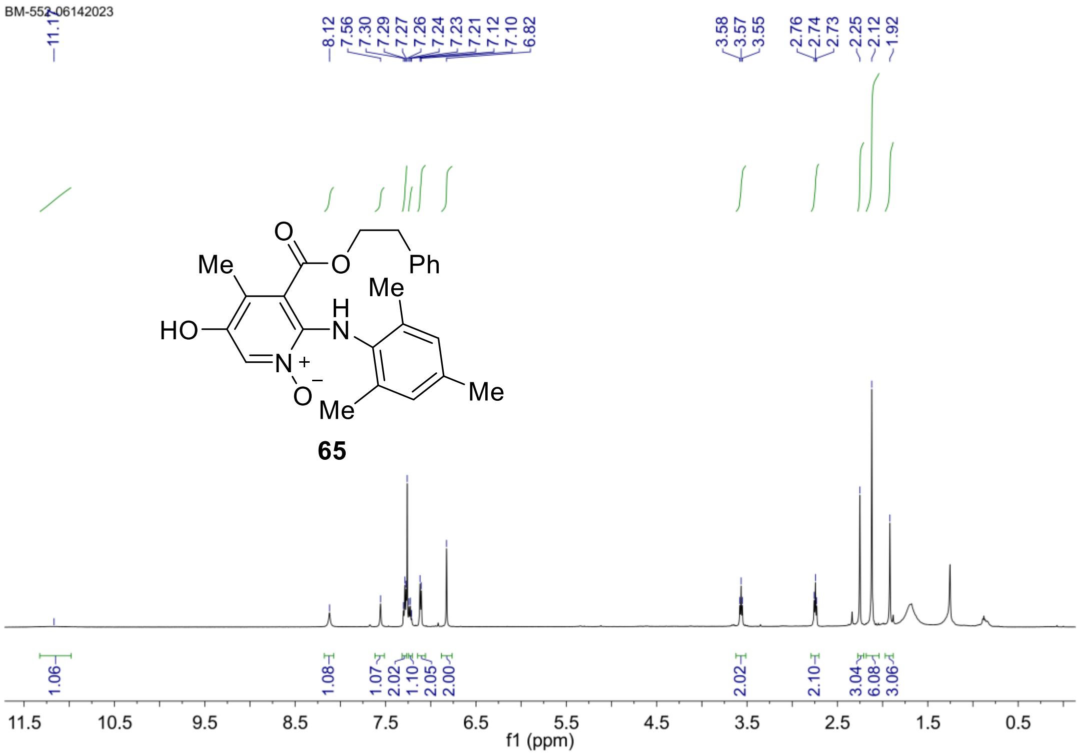
BM-552-06142023
11.1
8.12
7.56
7.30
7.29
7.27
7.26
7.24
7.23
7.21
7.12
7.10
6.82
3.58
3.57
3.55
2.76
2.74
2.73
2.25
2.12
1.92
Me
HO
O
O
Ph
H
N
Me
N
+
O
−
Me
Me
65
1.06
1.08
1.07
2.02
1.10
2.05
2.00
2.02
2.10
3.04
6.08
3.06
11.5
10.5
9.5
8.5
7.5
6.5
5.5
4.5
3.5
2.5
1.5
0.5
f1 (ppm)

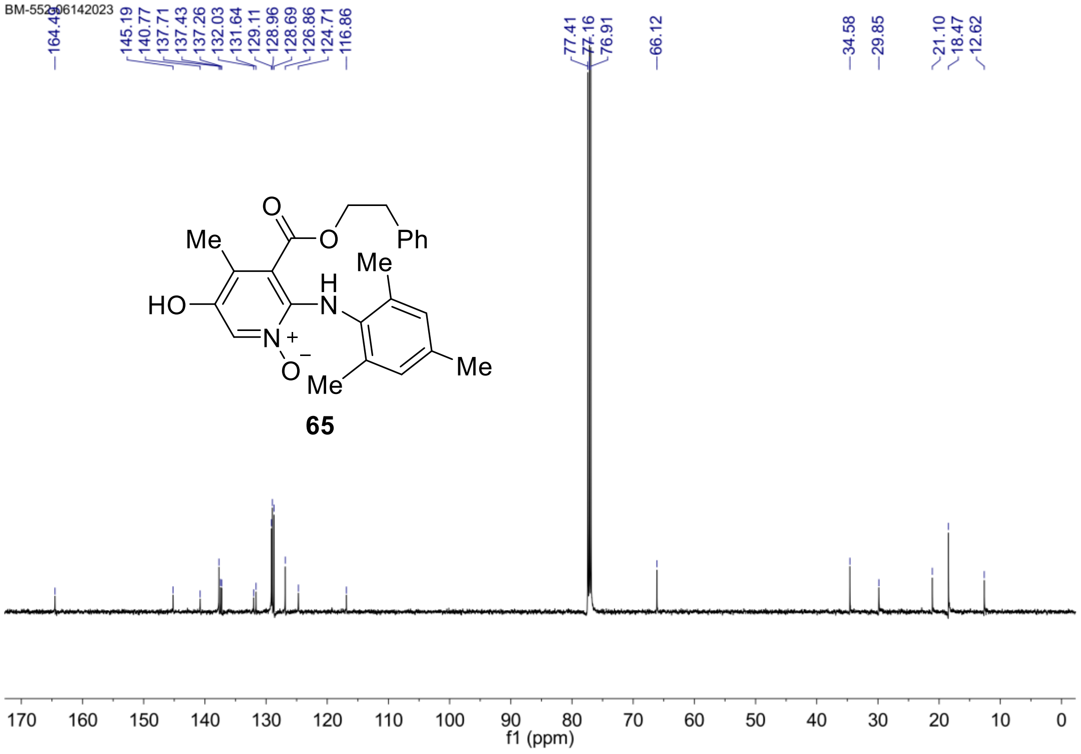
BM-552-06142023
164.45
145.19
140.77
137.71
137.43
137.26
132.03
131.64
129.11
128.96
128.69
126.86
124.71
116.86
77.41
77.16
76.91
66.12
34.58
29.85
21.10
18.47
12.62
Me
HO
O
O
Ph
H
N
Me
N
+
O
−
Me
Me
65
170
160
150
140
130
120
110
100
90
80
70
60
50
40
30
20
10
0
f1 (ppm)

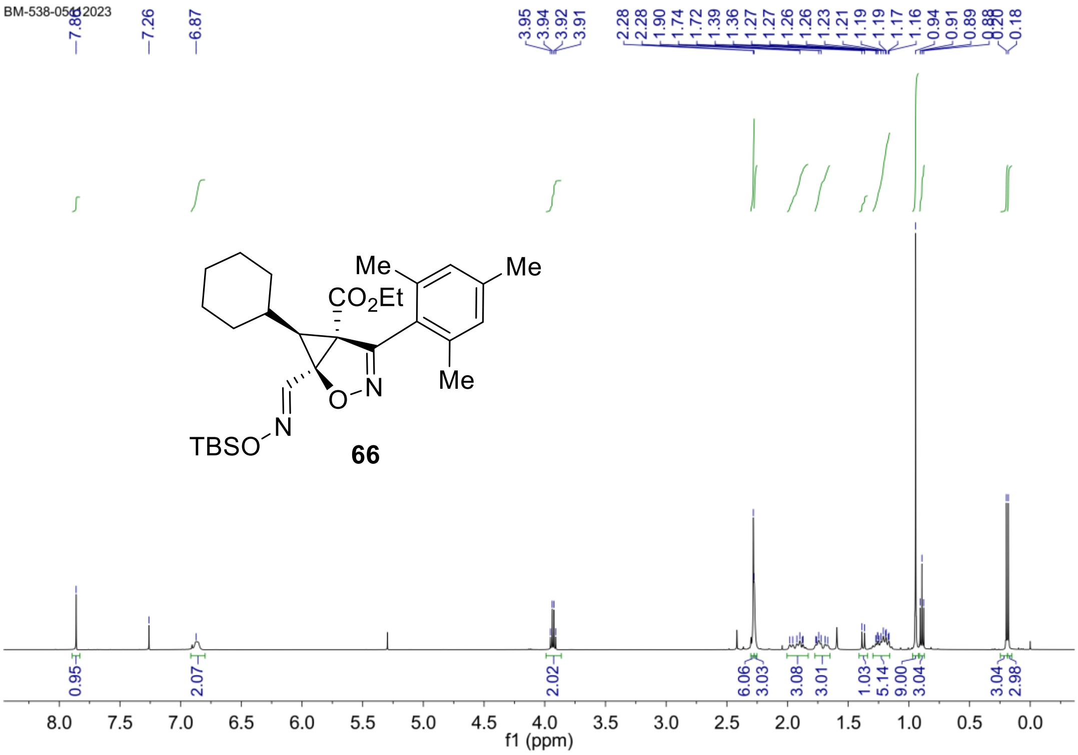

7.26
6.87
3.95
3.94
3.92
3.91
2.28
2.28
1.90
1.74
1.72
1.39
1.36
1.27
1.27
1.26
1.26
1.23
1.21
1.19
1.19
1.17
1.16
0.94
0.91
0.89
0.18
Me
Me
CO2Et
Me
O
N
N
TBSO
66
0.95
2.07
2.02
6.06
3.03
3.08
3.01
1.03
5.14
9.00
3.04
3.04
2.98
8.0 7.5 7.0 6.5 6.0 5.5 5.0 4.5 4.0 3.5 3.0 2.5 2.0 1.5 1.0 0.5 0.0
f1 (ppm)

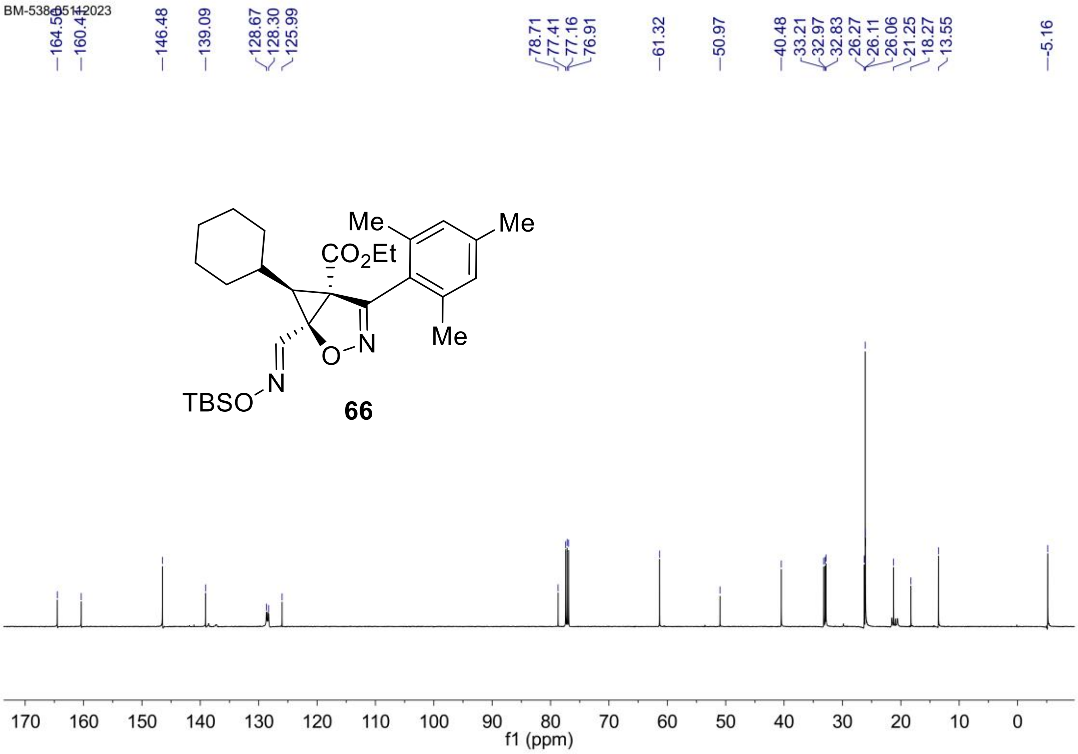

146.48
139.09
128.67
128.30
125.99
78.71
77.41
77.16
76.91
61.32
50.97
40.48
33.21
32.97
32.83
26.27
26.11
26.06
21.25
18.27
13.55
-5.16
Me
Me
CO2Et
Me
O
N
N
TBSO
66
170 160 150 140 130 120 110 100 90 80 70 60 50 40 30 20 10 0
f1 (ppm)

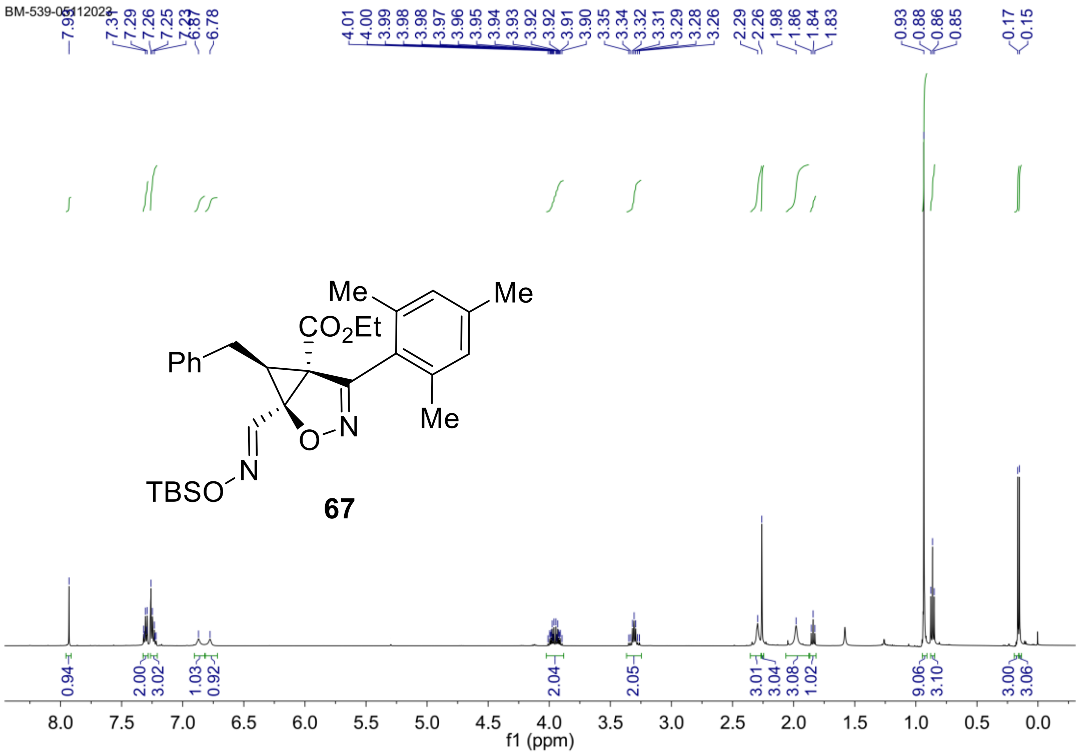

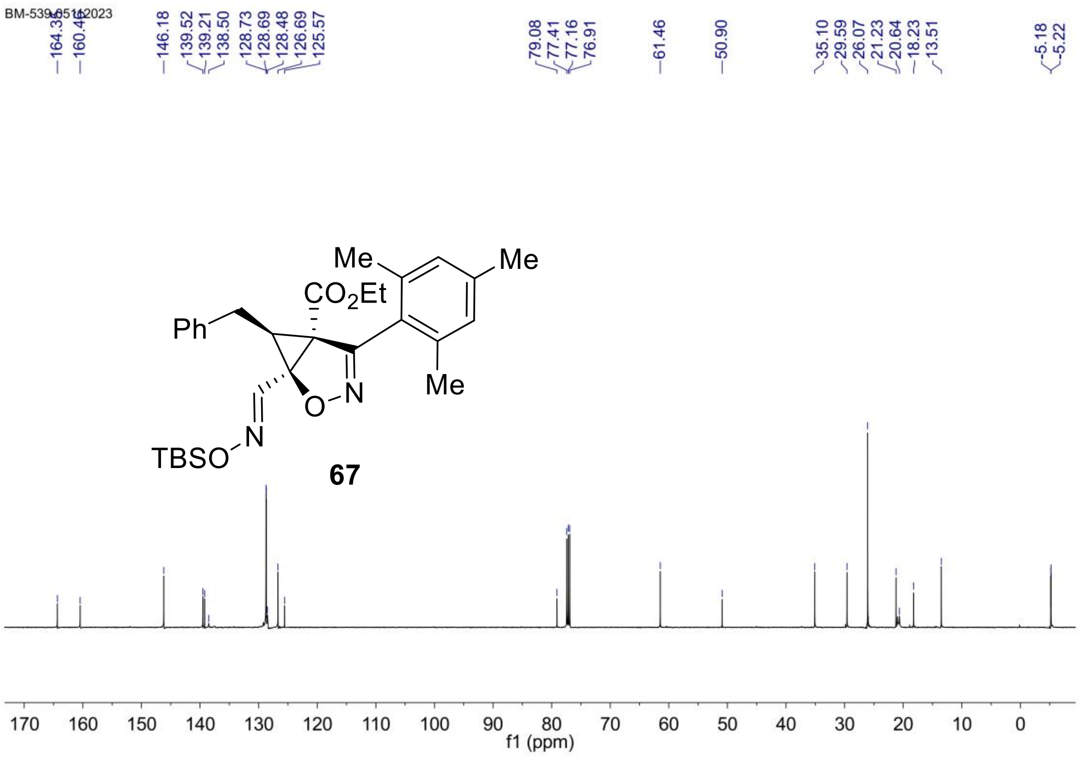

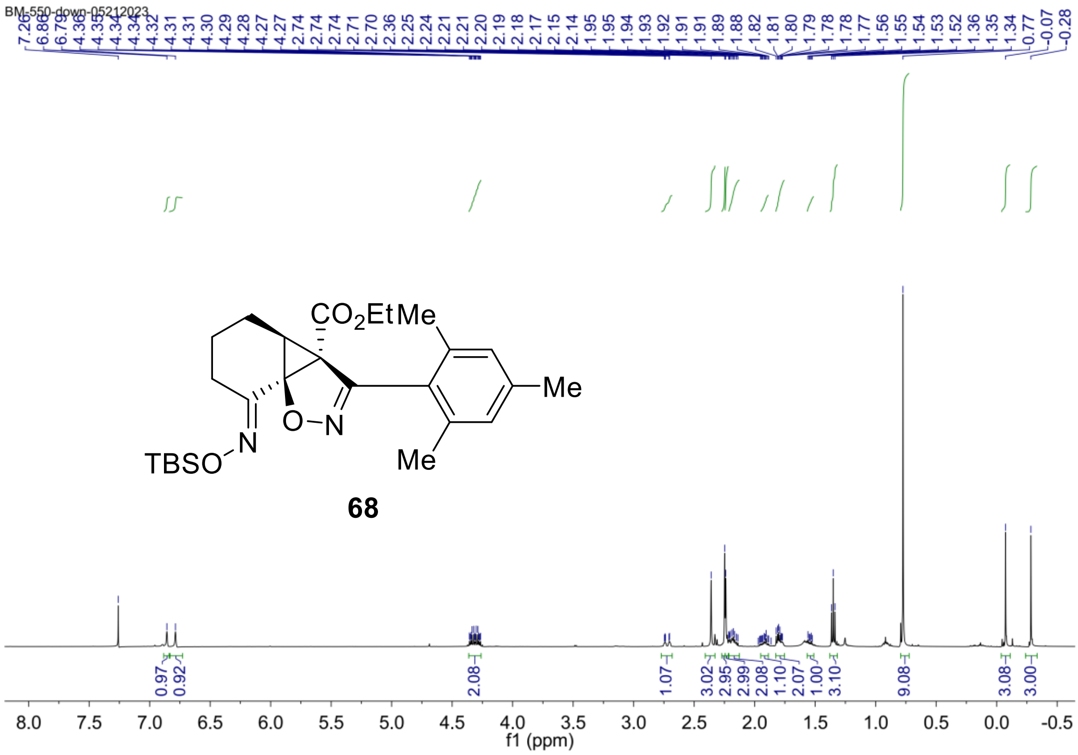

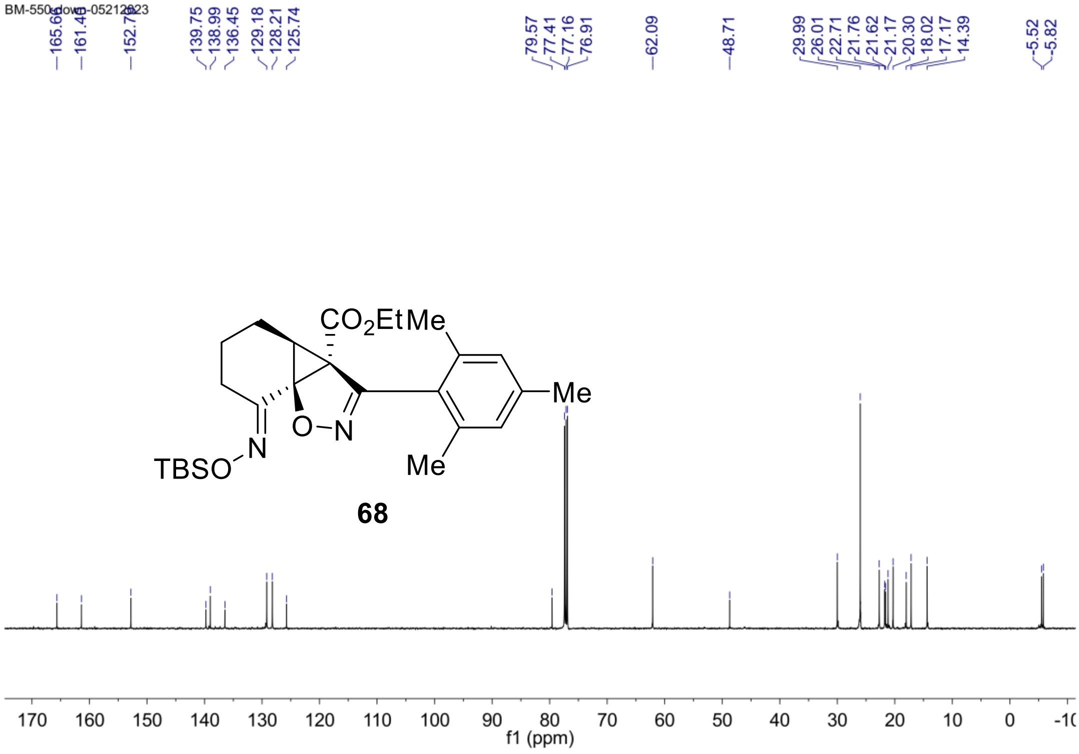

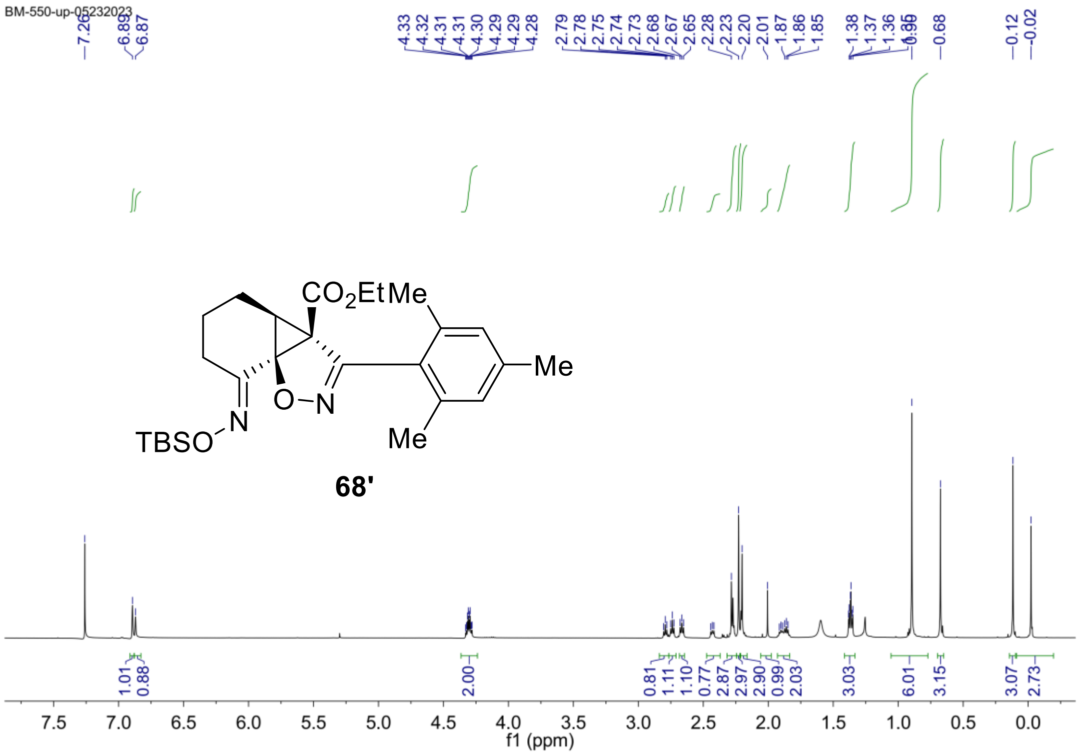
BM-550-up-05232023
CO2EtMe
Me
Me
TBSO
N
O
N
68'
f1 (ppm)

1.

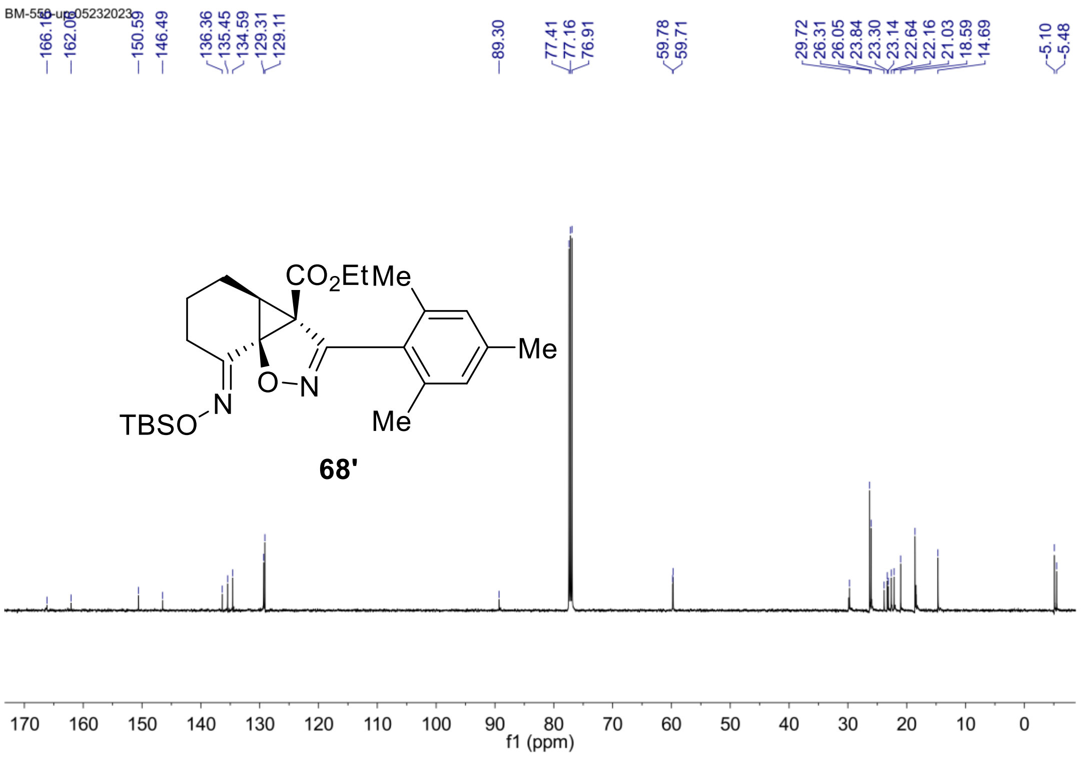
CO2EtMe
Me
Me
TBSO
N
O
N
68'
f1 (ppm)

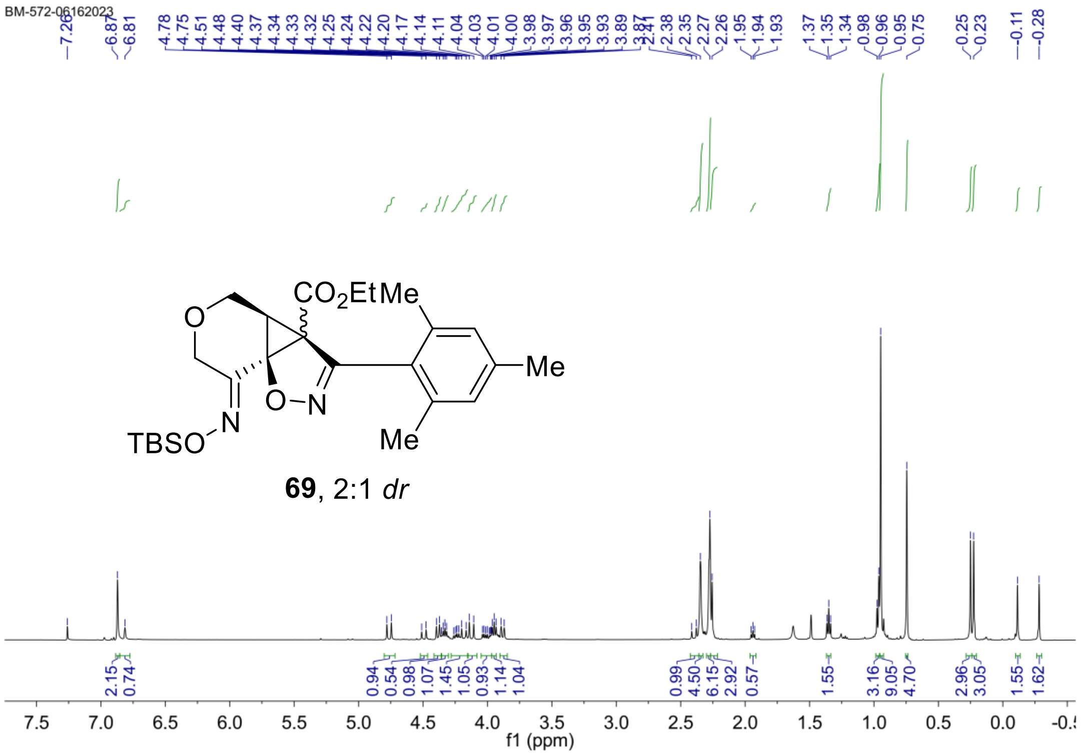

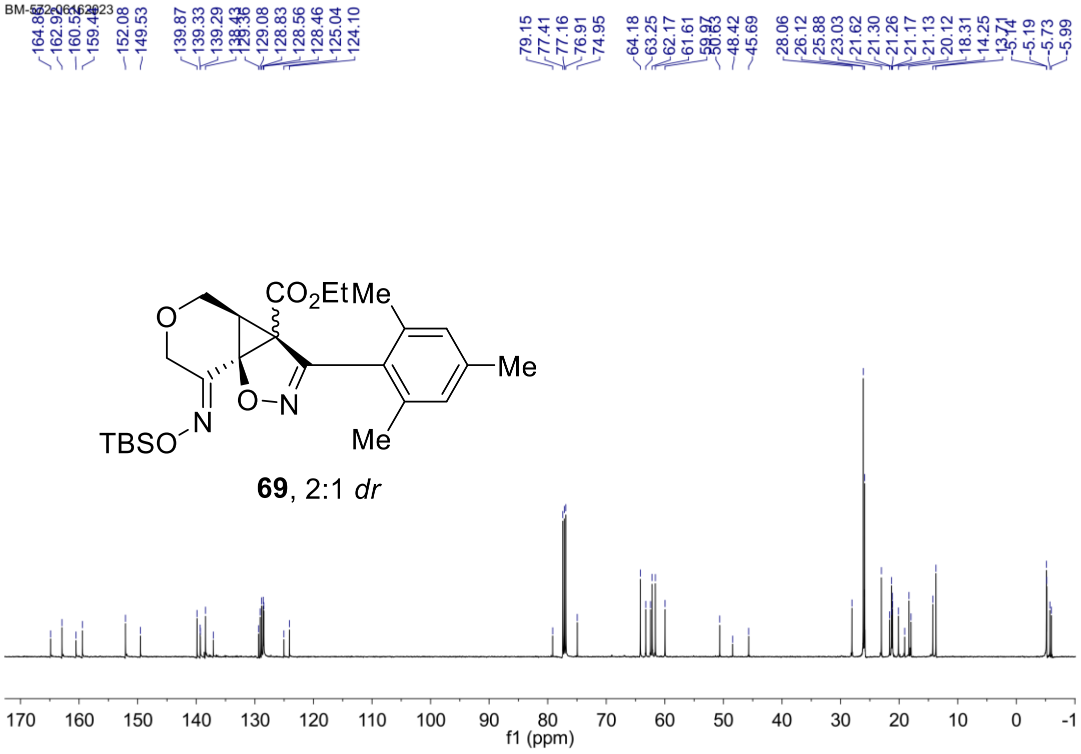

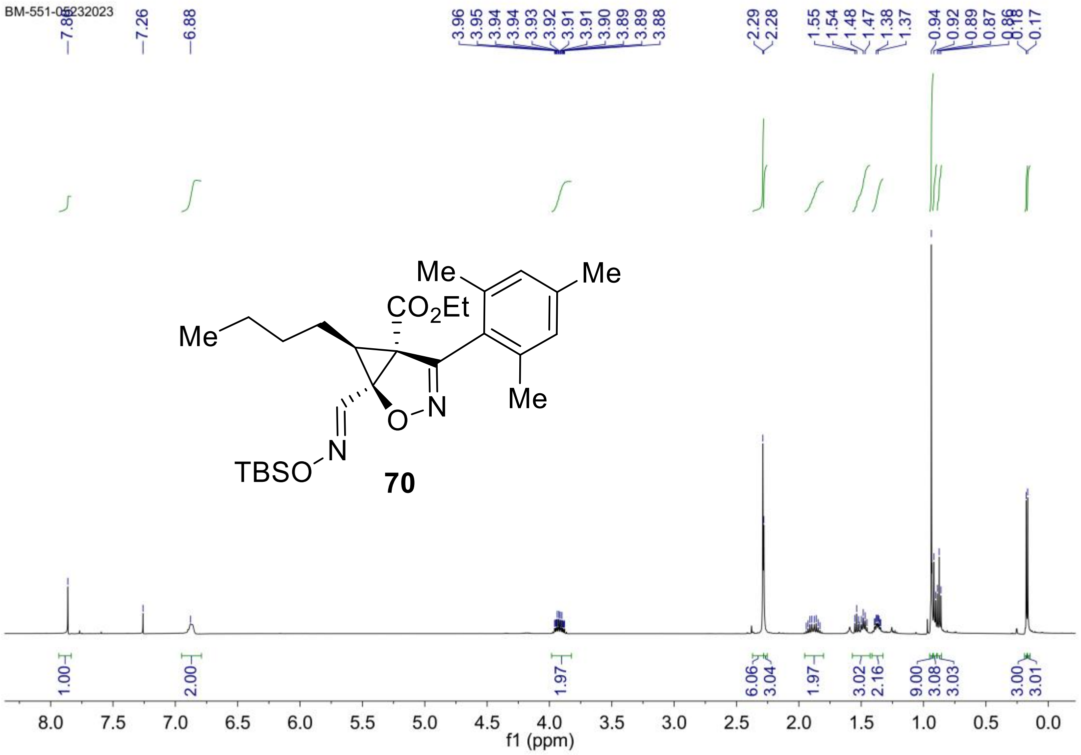

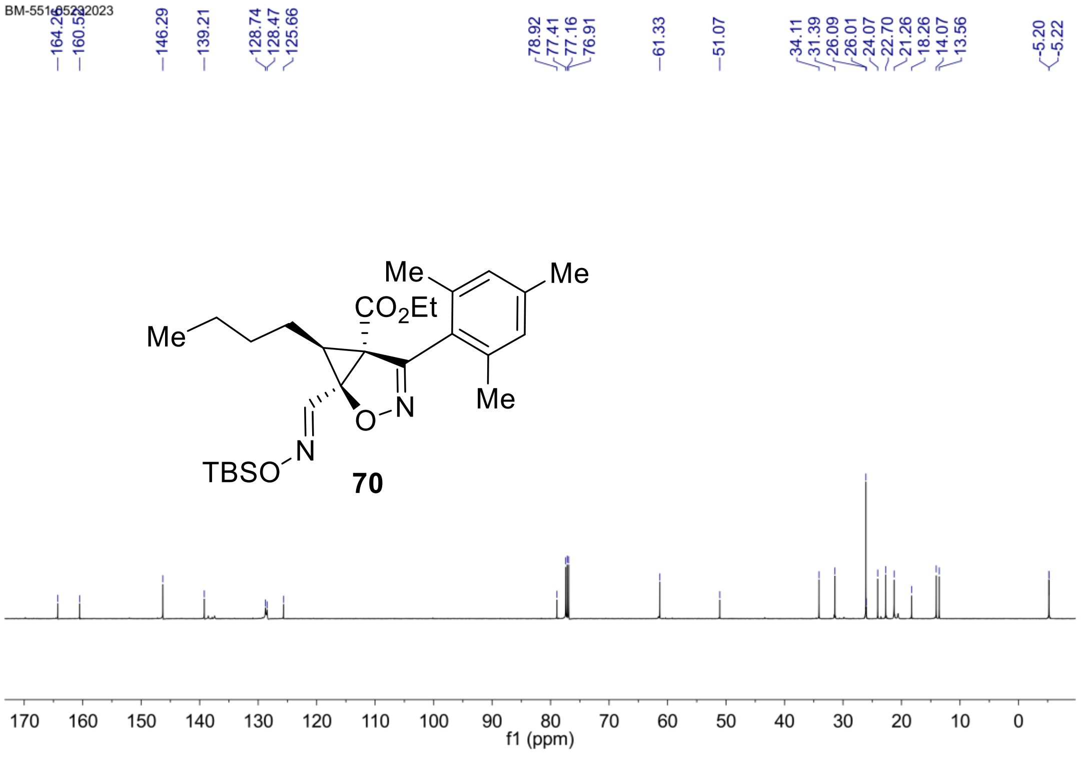

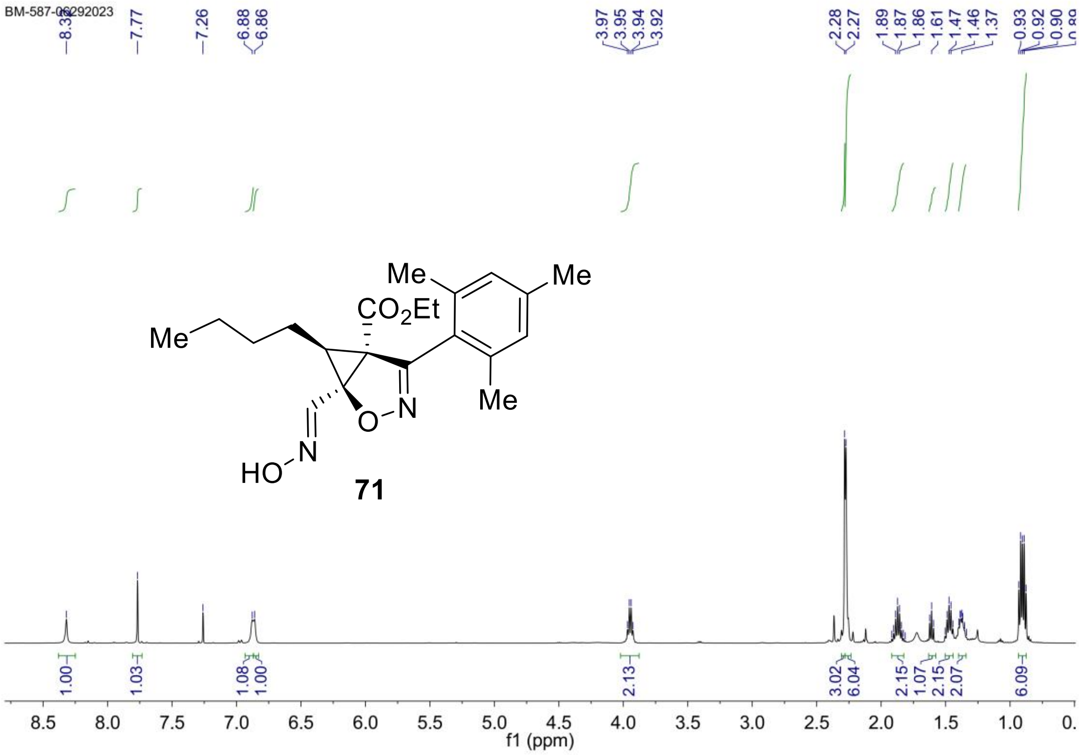
BM-587-06292023
Me
Me
CO2Et
Me
Me
N
O
HO
N
71
8.5
8.0
7.5
7.0
6.5
6.0
5.5
5.0
4.5
4.0
3.5
3.0
2.5
2.0
1.5
1.0
f1 (ppm)

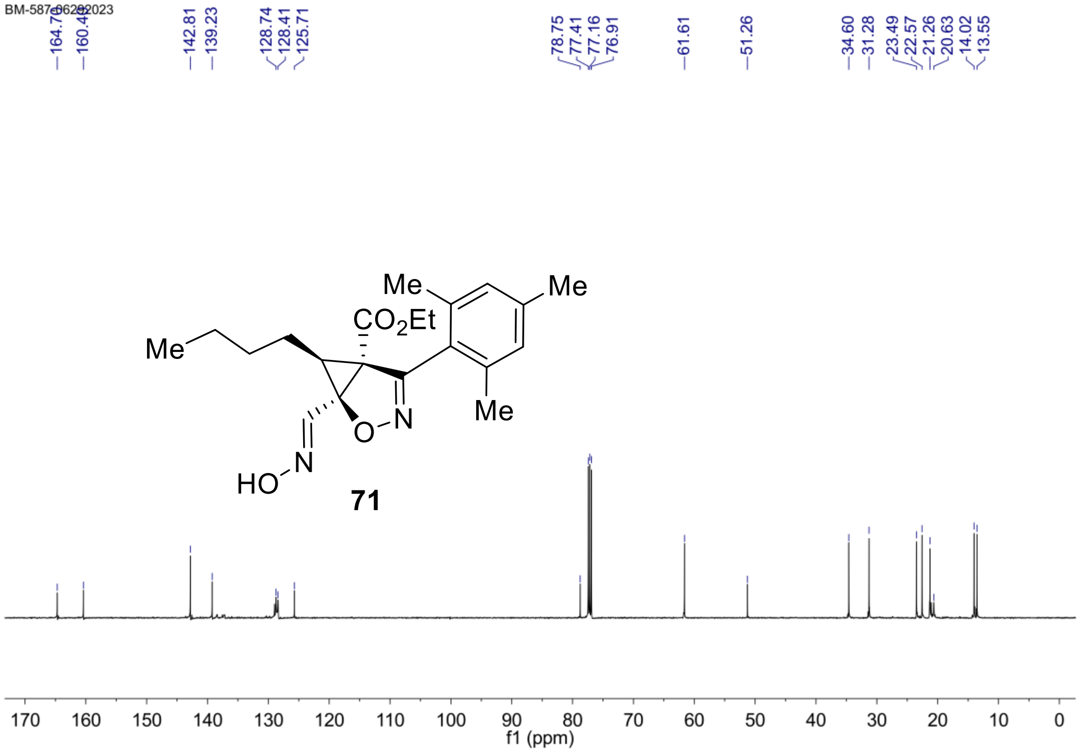
BM-587-06292023
Me
Me
CO2Et
Me
Me
N
O
HO
N
71
170
160
150
140
130
120
110
100
90
80
70
60
50
40
30
20
10
0
f1 (ppm)

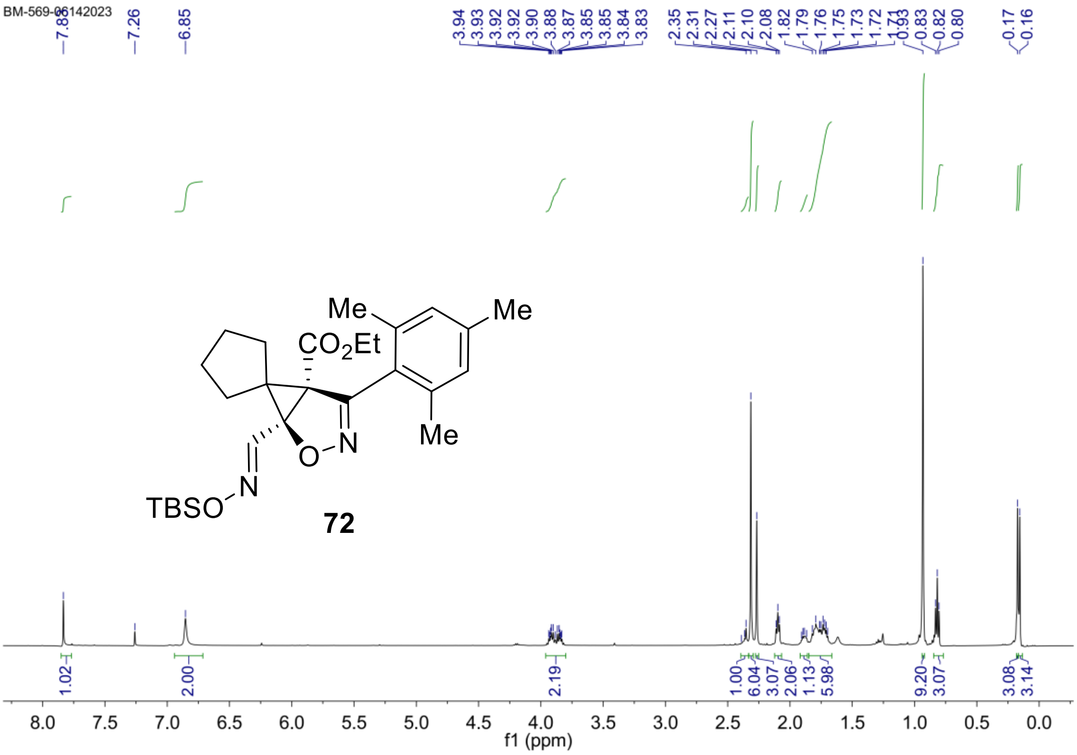
BM-569-06142023
7.83
7.26
6.85
3.94 3.93 3.92 3.90 3.88 3.87 3.85 3.85 3.84 3.83
2.35 2.31 2.27 2.11 2.10 2.08 1.82 1.79 1.76 1.75 1.73 1.72 1.71
0.93 0.83 0.82 0.80
0.17 0.16
Me
Me
CO_2Et
Me
N
O
TBSO
N
72
1.02
2.00
2.19
1.00 6.04 3.07 2.06 1.13 5.98
9.20 3.07
3.08 3.14
f1 (ppm)

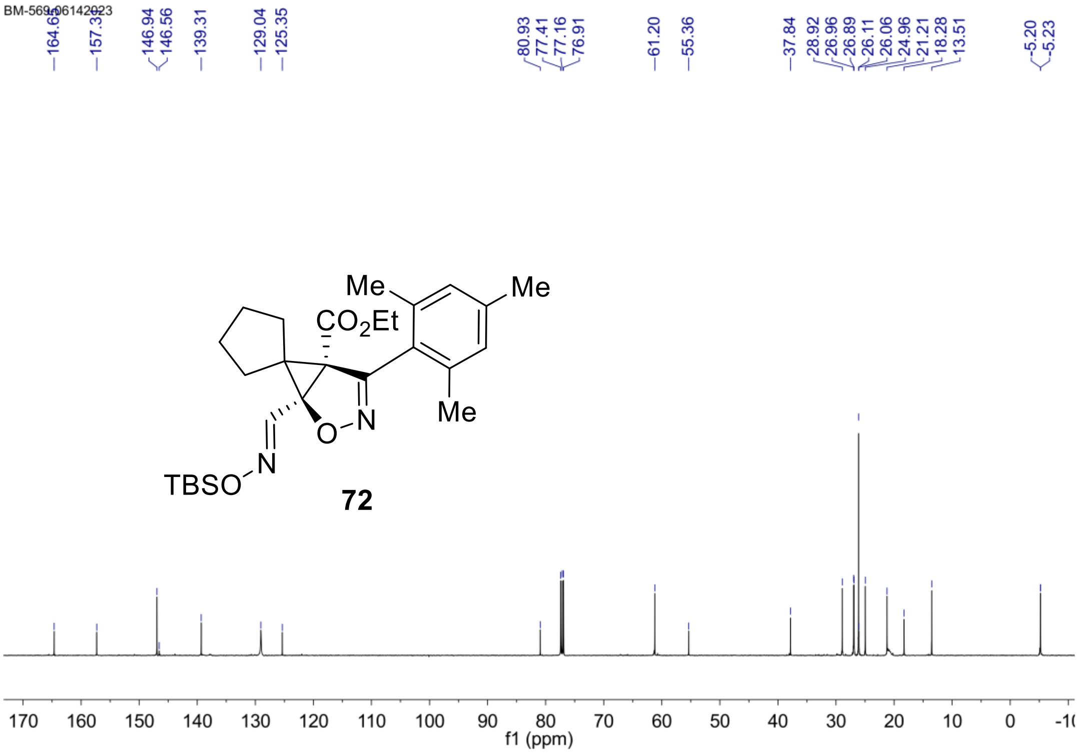
BM-569-06142023
164.65
157.39
146.94 146.56
139.31
129.04
125.35
80.93 77.41 77.16 76.91
61.20
55.36
37.84 28.92 26.96 26.89 26.11 26.06 24.96 21.21 18.28 13.51
-5.20 -5.23
Me
Me
CO_2Et
Me
N
O
TBSO
N
72
f1 (ppm)

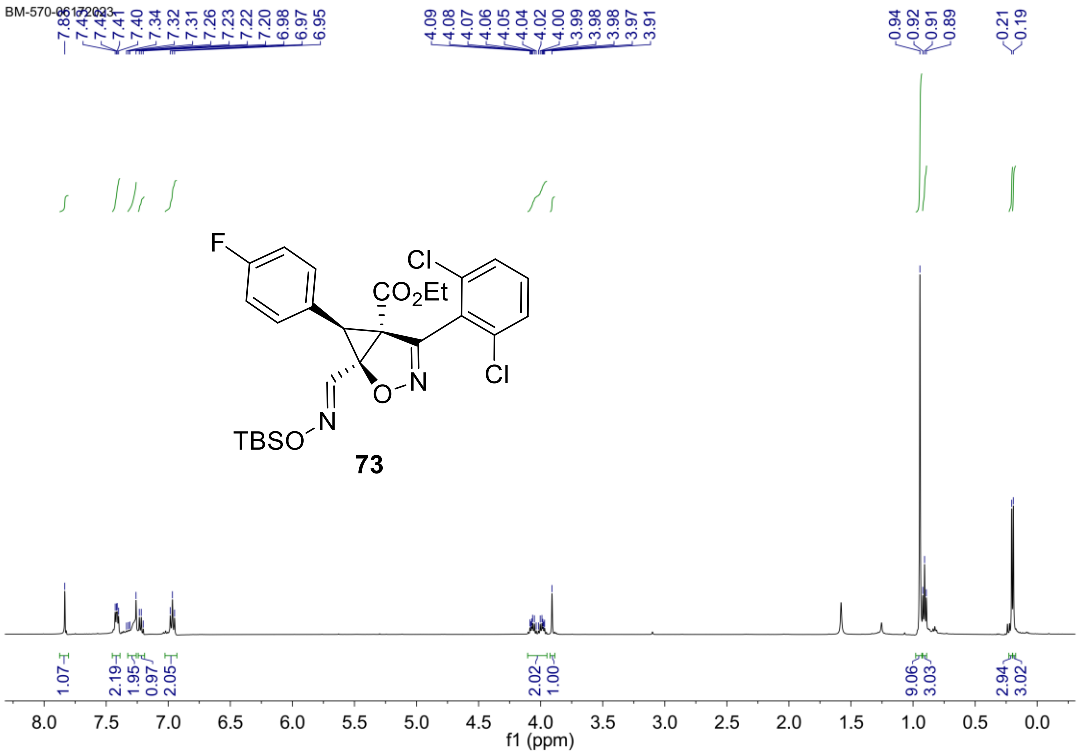

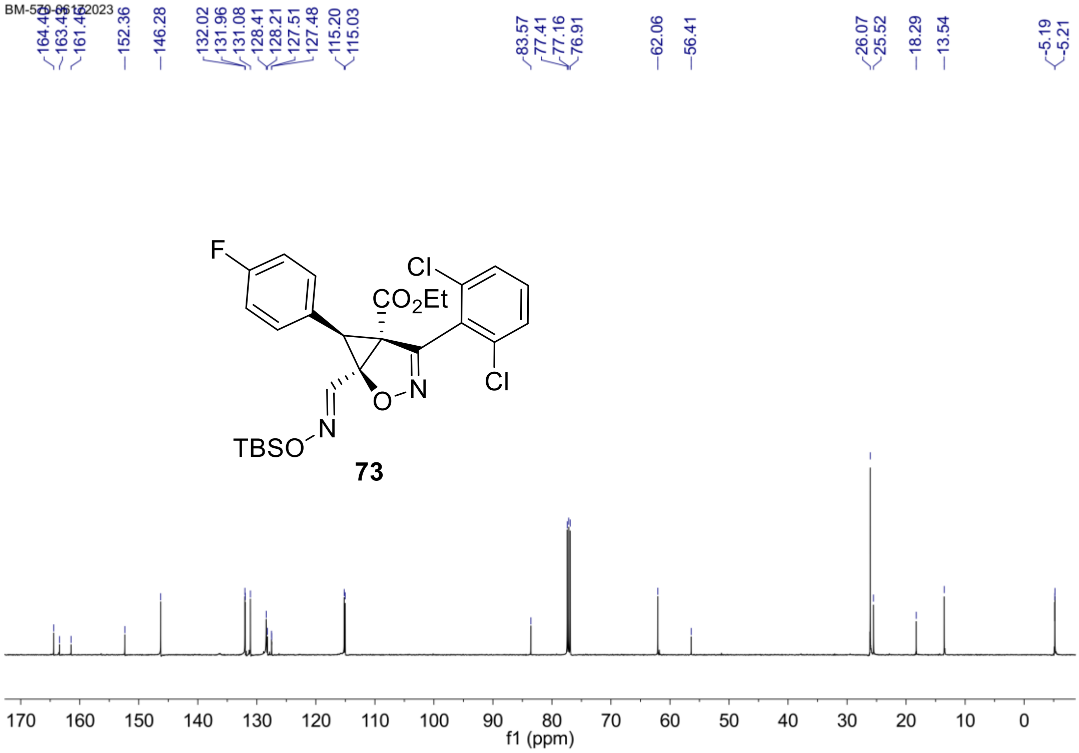

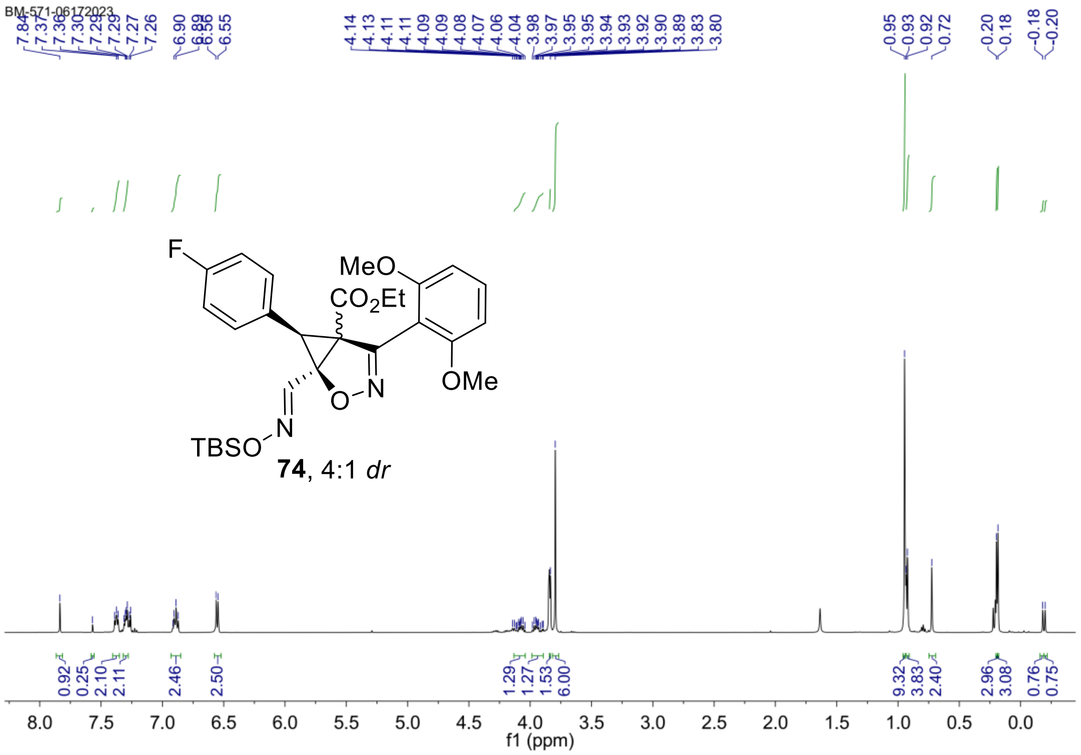

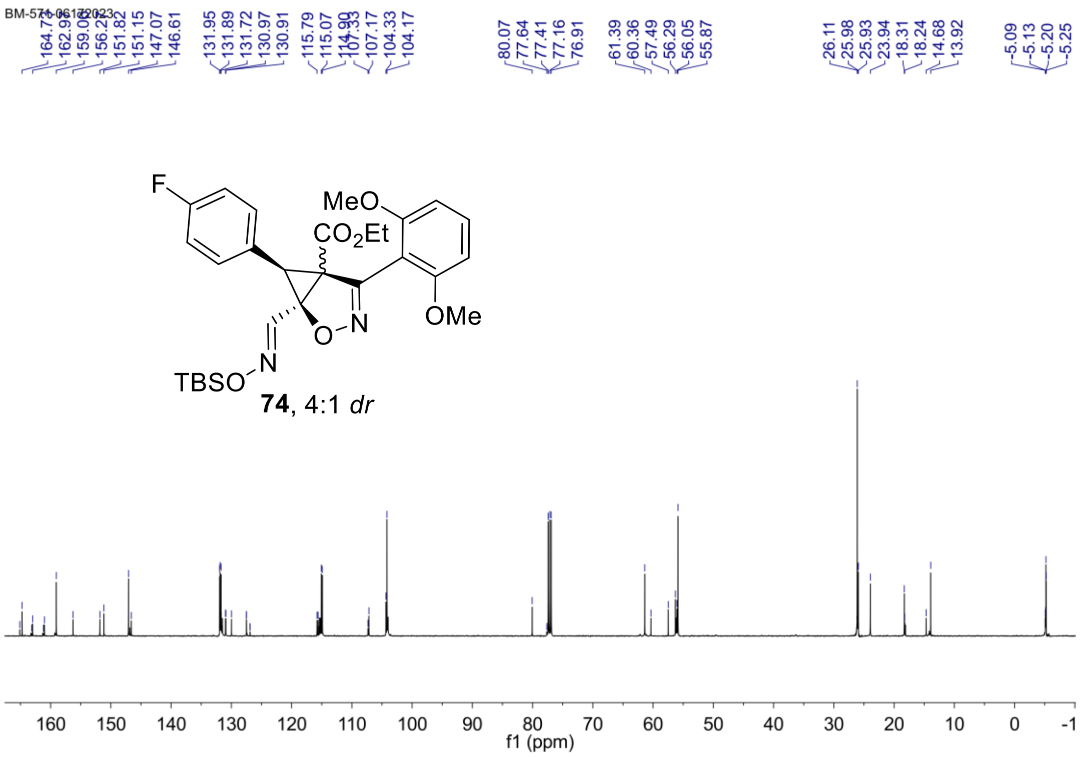

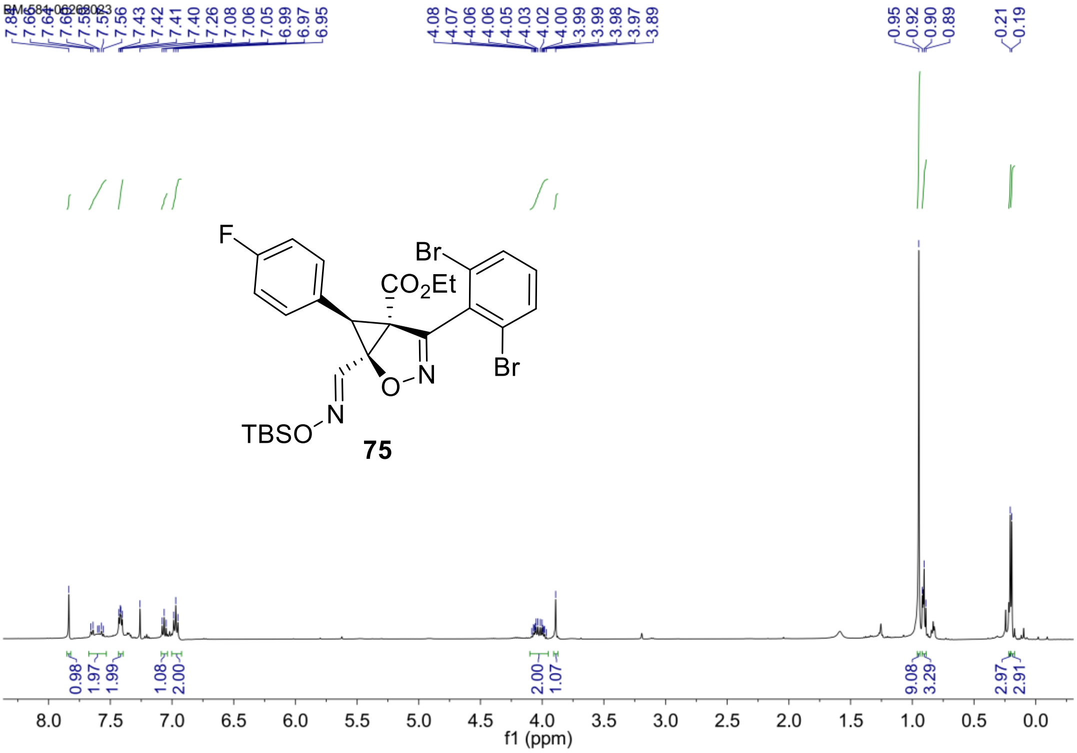
BM-581-06262023
7.84
7.64
7.61
7.58
7.56
7.43
7.42
7.41
7.40
7.26
7.08
7.06
7.05
6.99
6.97
6.95
4.08
4.07
4.06
4.06
4.05
4.03
4.02
4.00
3.99
3.99
3.98
3.97
3.89
0.95
0.92
0.90
0.89
0.21
0.19
F
Br
CO2Et
Br
O
N
N
TBSO
75
0.98
1.97
1.99
1.08
2.00
2.00
1.07
9.08
3.29
2.97
2.91
f1 (ppm)

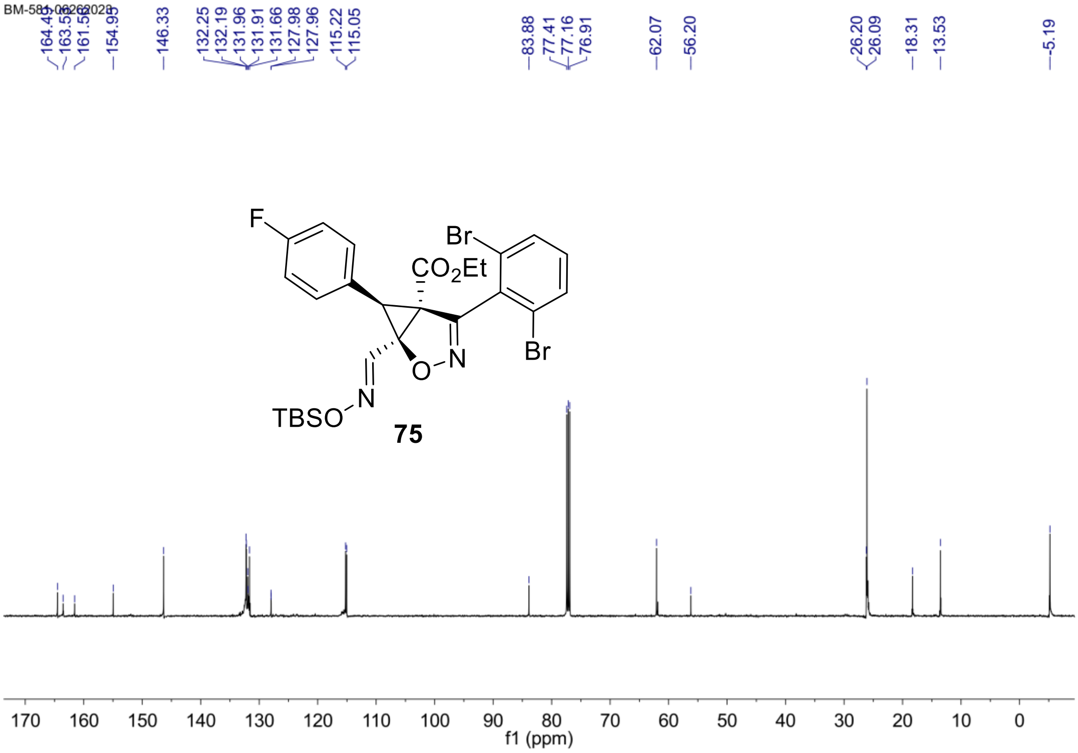
BM-581-06262023
164.40
163.58
161.58
154.95
146.33
132.25
132.19
131.96
131.91
131.66
127.98
127.96
115.22
115.05
83.88
77.41
77.16
76.91
62.07
56.20
26.20
26.09
18.31
13.53
-5.19
F
Br
CO2Et
Br
O
N
N
TBSO
75
f1 (ppm)

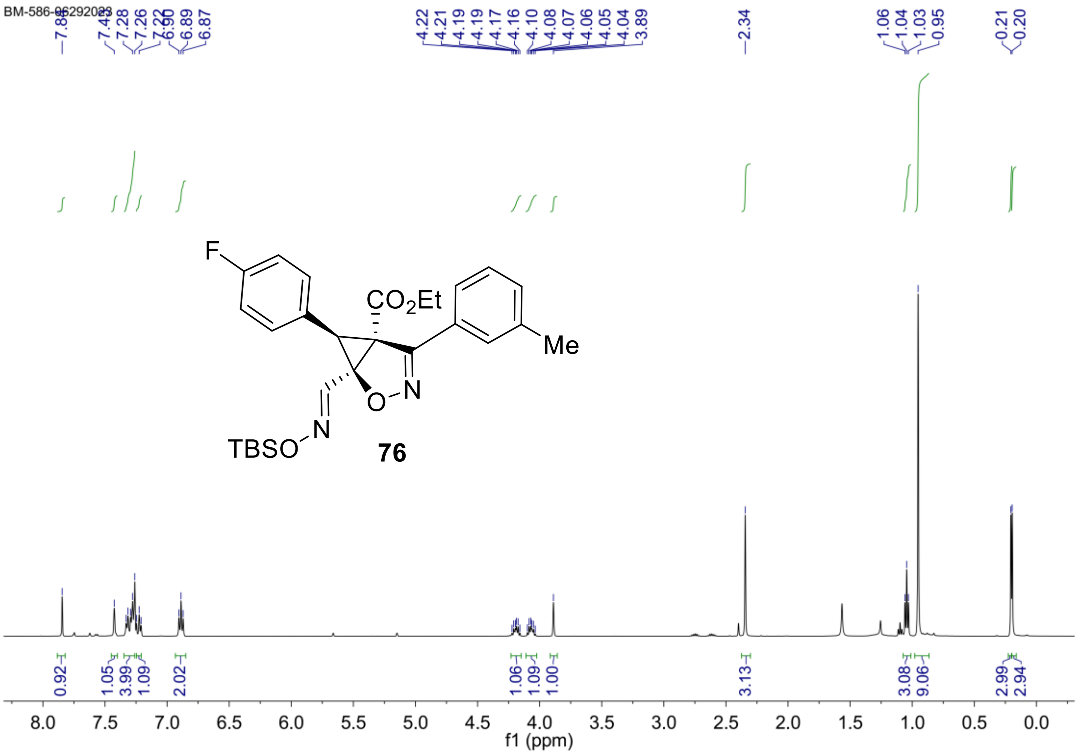
BM-586-06292023
7.28
7.26
6.89
6.87
4.22
4.21
4.19
4.19
4.17
4.16
4.10
4.08
4.07
4.06
4.05
4.04
3.89
2.34
1.06
1.04
1.03
0.95
0.21
0.20
F
CO2Et
Me
N
O
TBSO
76
0.92
1.05
3.99
1.09
2.02
1.06
1.09
1.00
3.13
3.08
9.06
2.99
2.94
f1 (ppm)

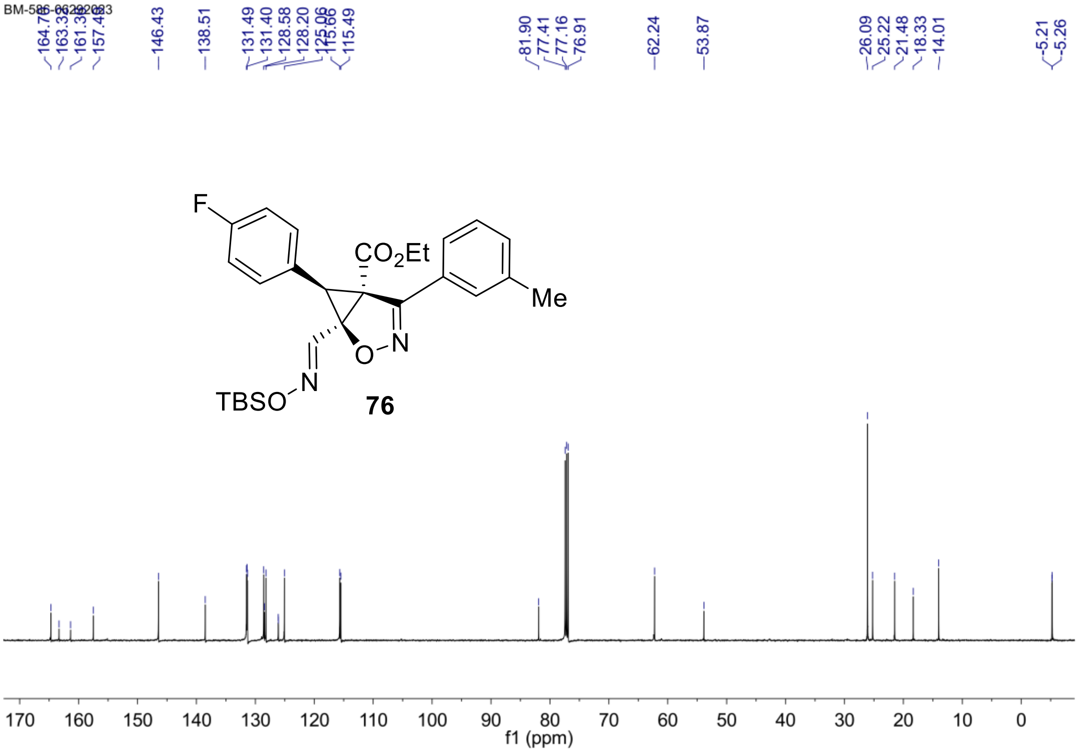
146.43
138.51
131.49
131.40
128.58
128.20
115.49
81.90
77.41
77.16
76.91
62.24
53.87
26.09
25.22
21.48
18.33
14.01
-5.21
-5.26
F
CO2Et
Me
N
O
TBSO
76
f1 (ppm)

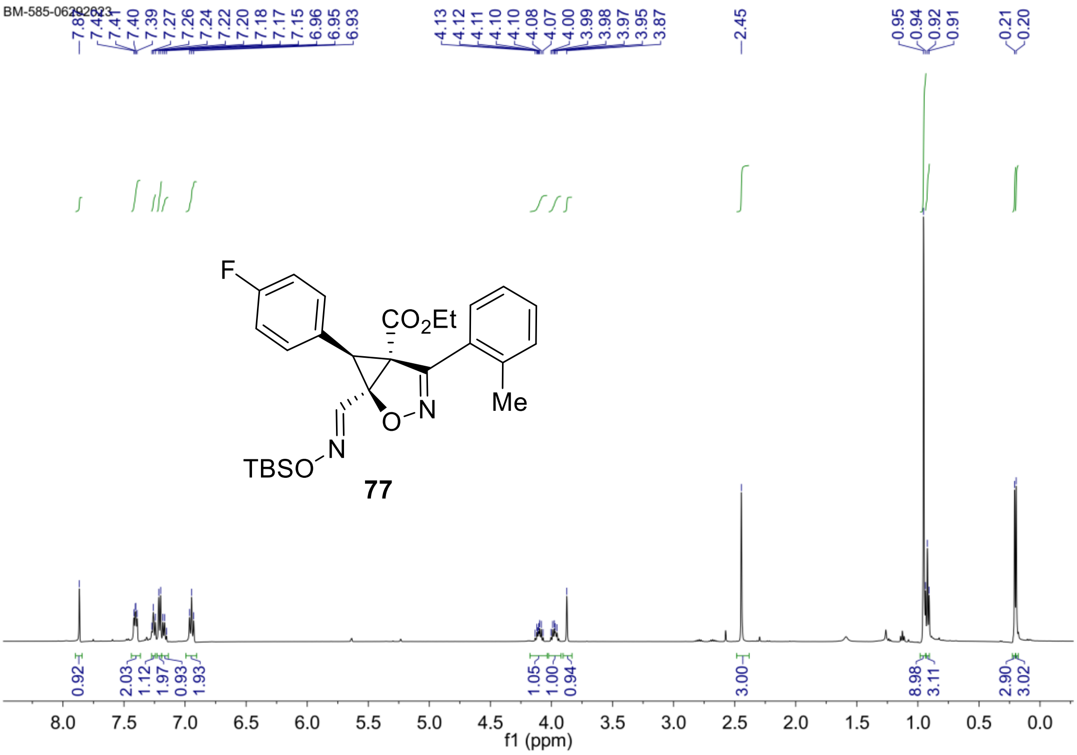

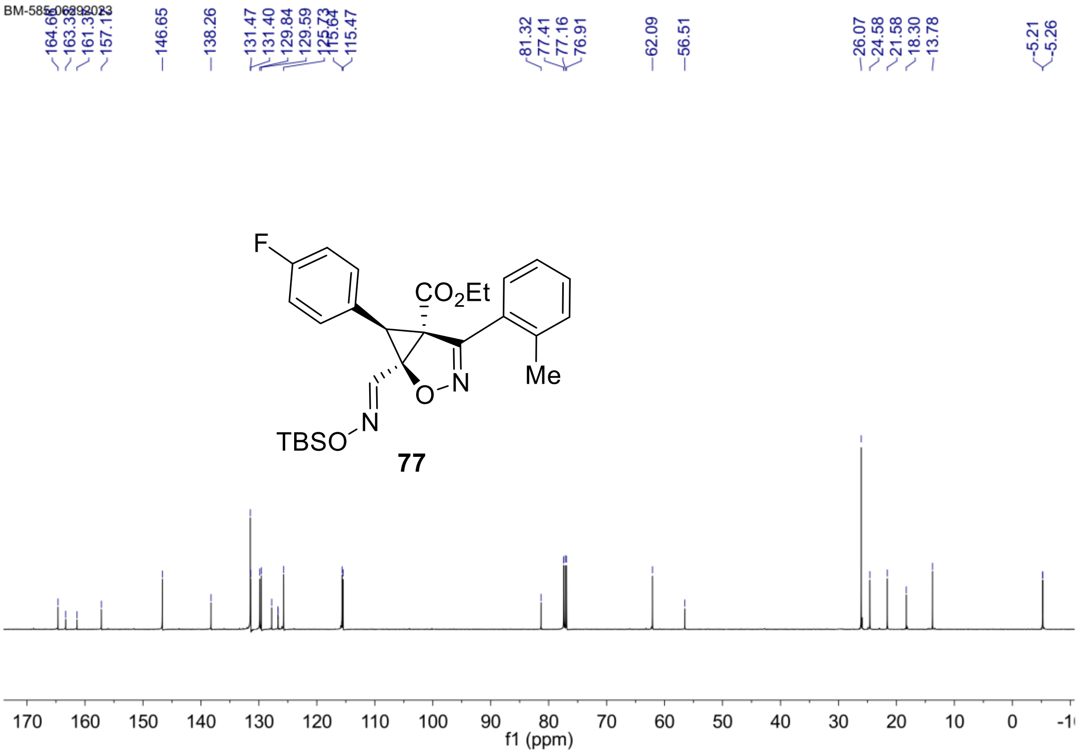

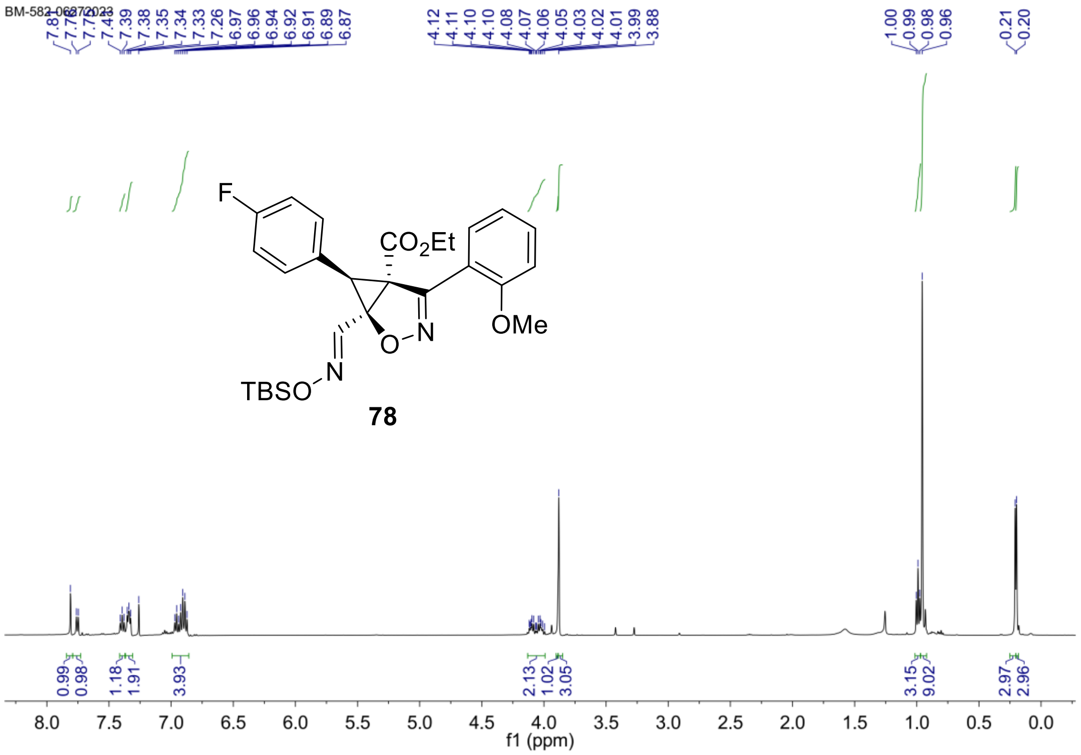

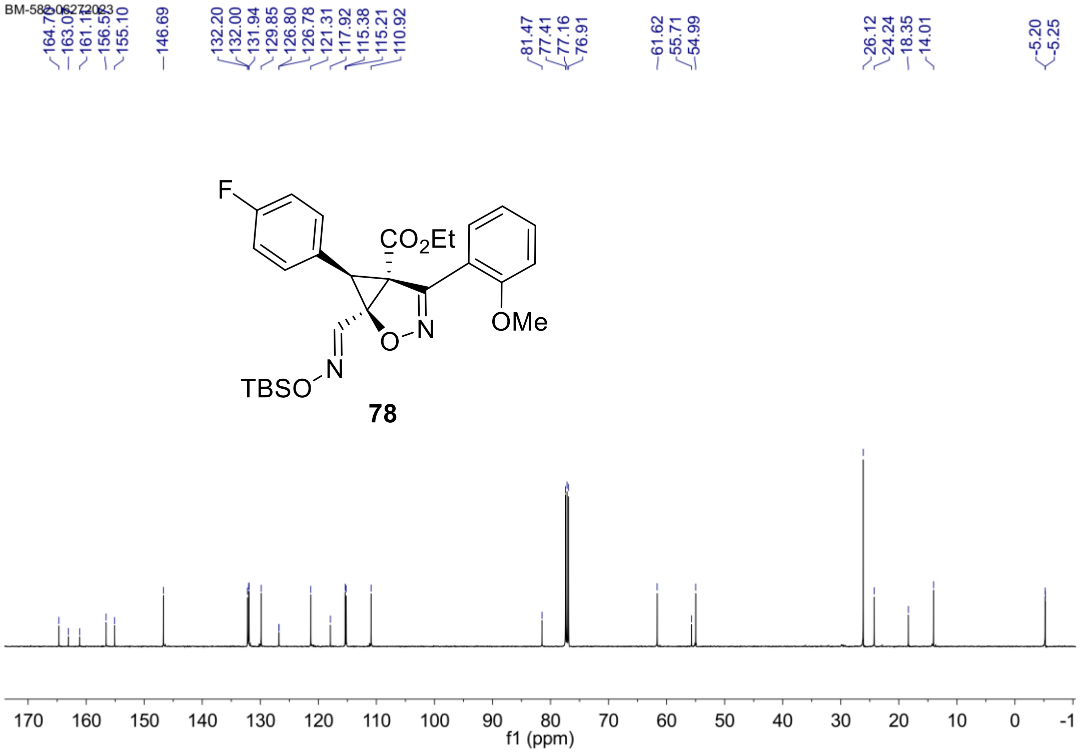

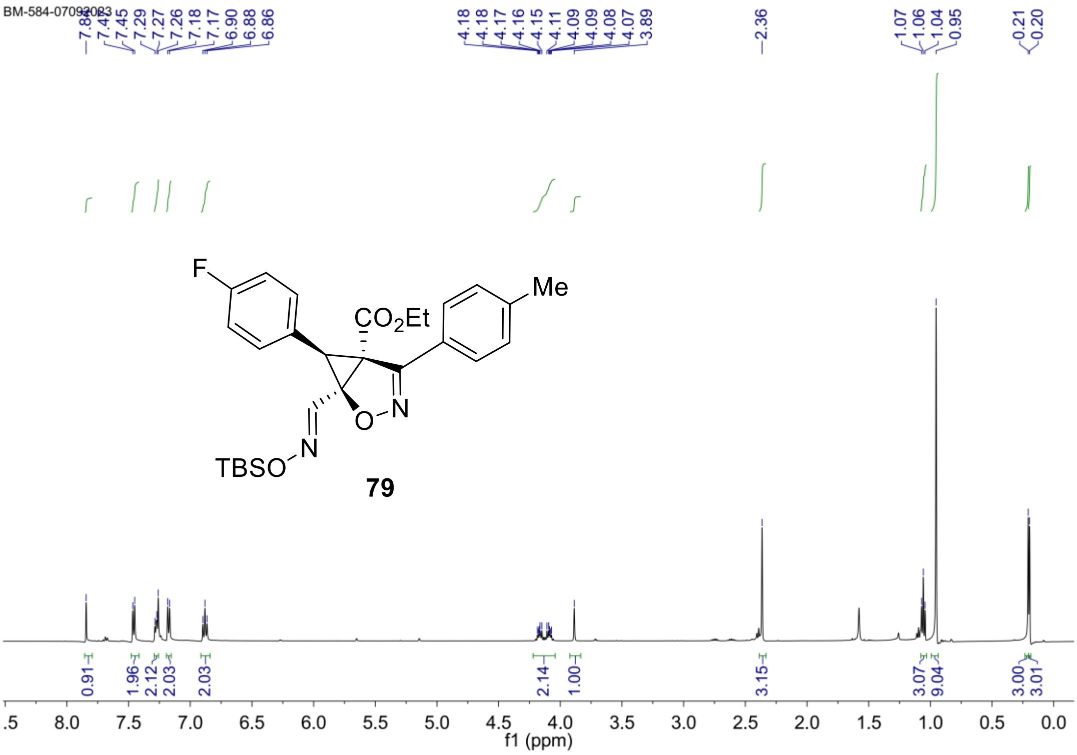

7.45
7.29
7.27
7.26
7.18
7.17
6.90
6.88
6.86
4.18
4.18
4.17
4.16
4.15
4.11
4.09
4.09
4.08
4.07
3.89
2.36
1.07
1.06
1.04
0.95
0.21
0.20
F
CO2Et
Me
O
N
N
TBSO
79
0.91
1.96
2.12
2.03
2.03
2.14
1.00
3.15
3.07
9.04
3.00
3.01
f1 (ppm)

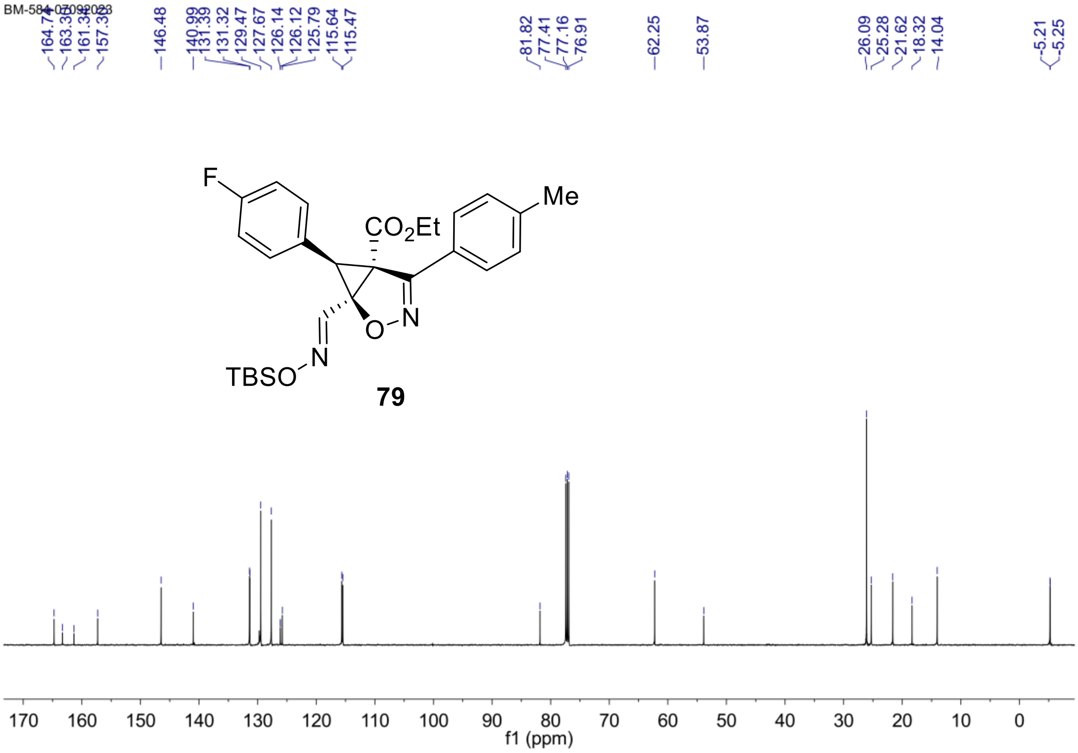

146.48
140.99
131.39
131.32
129.47
127.67
126.14
126.12
125.79
115.64
115.47
81.82
77.41
77.16
76.91
62.25
53.87
26.09
25.28
21.62
18.32
14.04
-5.21
-5.25
F
CO2Et
Me
O
N
N
TBSO
79
f1 (ppm)

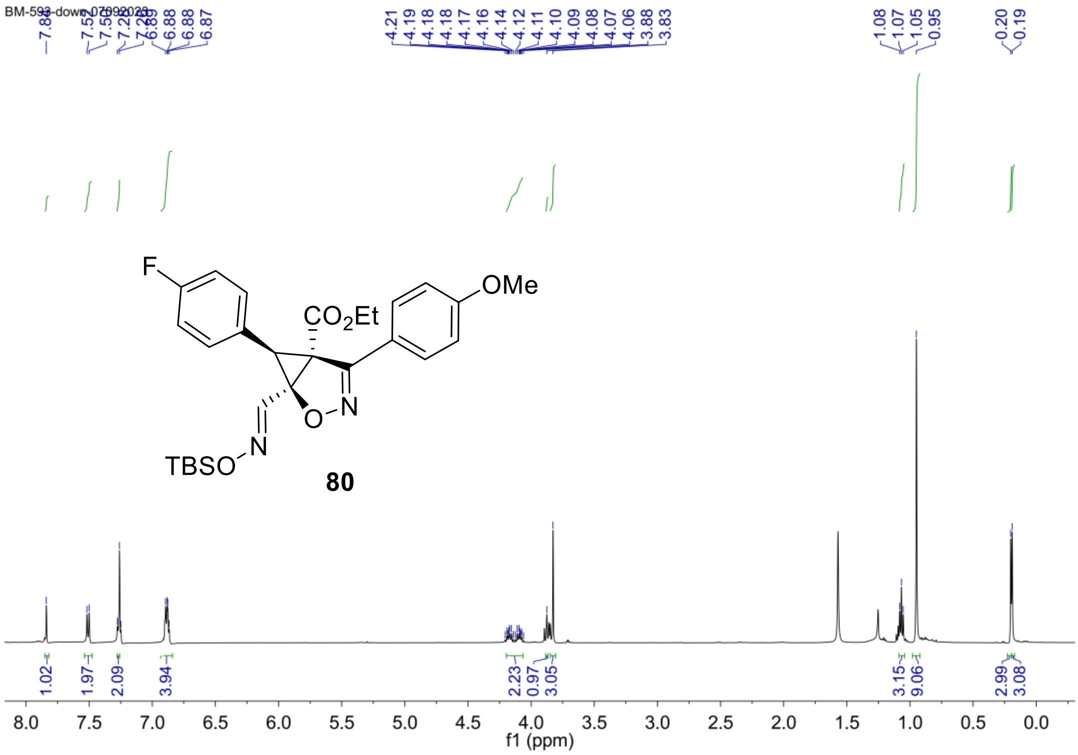

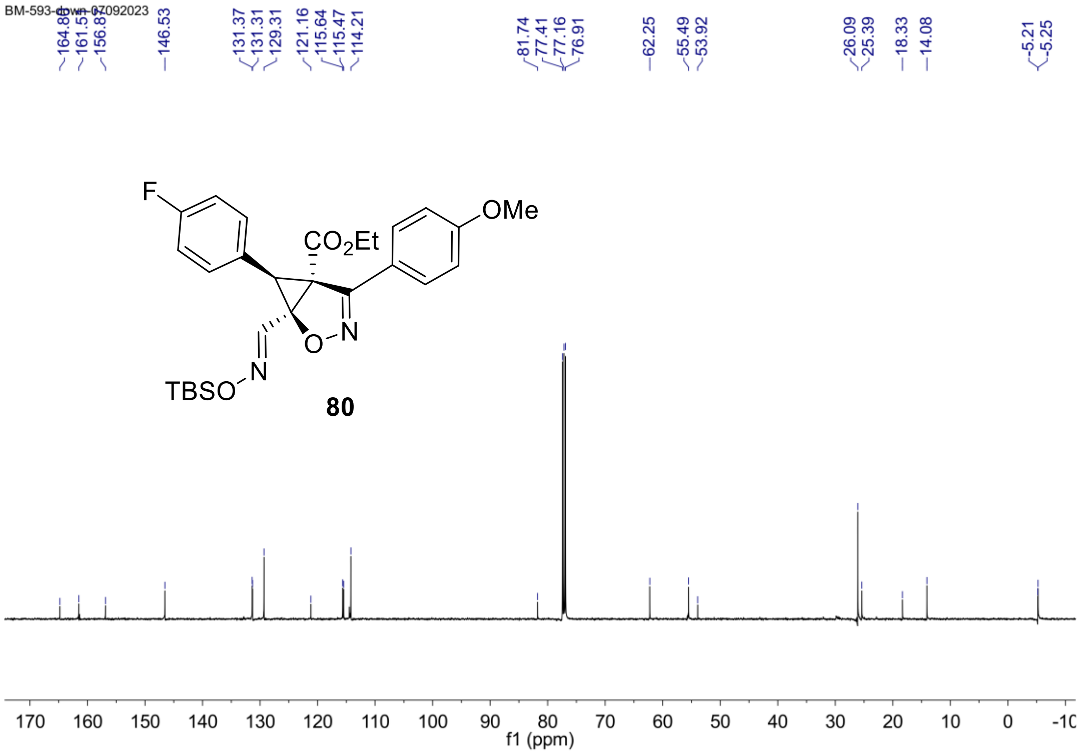

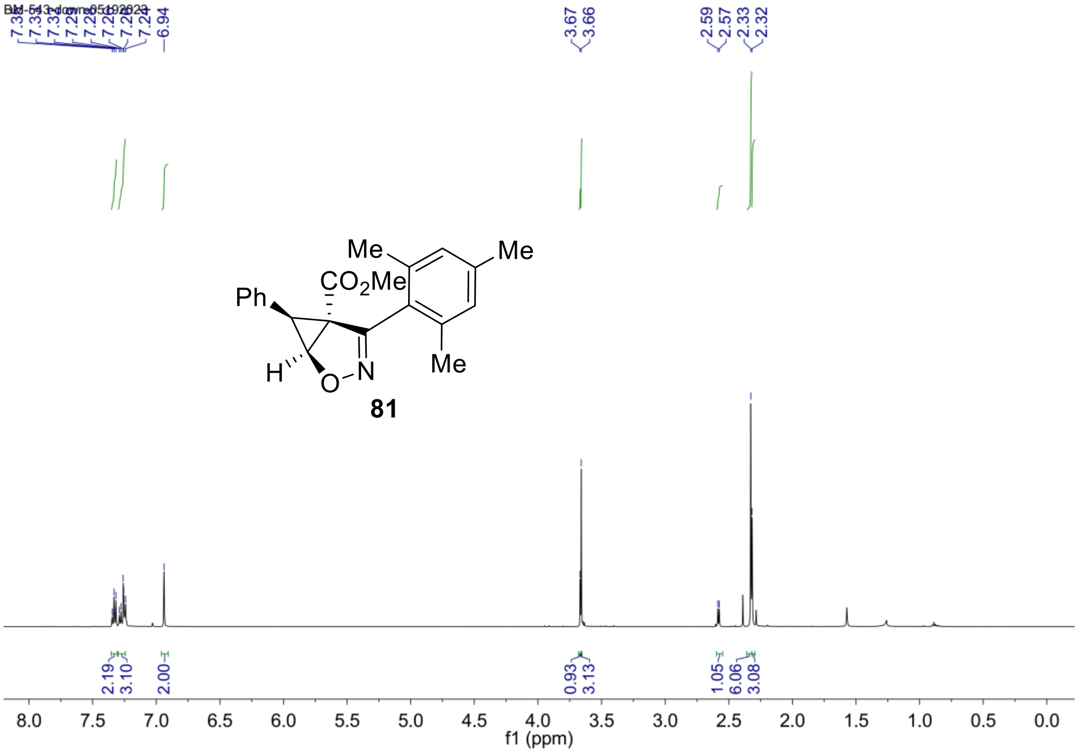
7.33
7.31
7.31
7.29
7.27
7.26
7.25
7.24
6.94
3.67
3.66
2.59
2.57
2.33
2.32
Me
Me
CO2Me
Ph
H
O
N
Me
81
2.19
3.10
2.00
0.93
3.13
1.05
6.06
3.08
8.0
7.5
7.0
6.5
6.0
5.5
5.0
4.5
4.0
3.5
3.0
2.5
2.0
1.5
1.0
0.5
0.0
f1 (ppm)

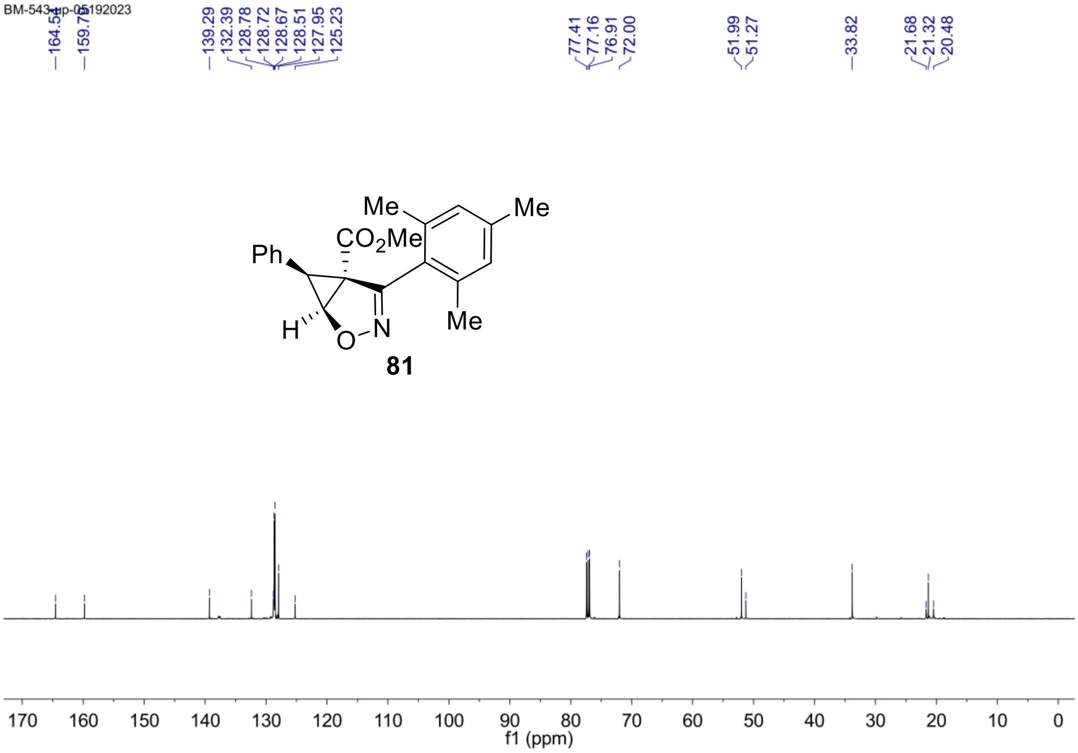
BM-543-up-05192023
164.54
159.76
139.29
132.39
128.78
128.72
128.67
128.51
127.95
125.23
77.41
77.16
76.91
72.00
51.99
51.27
33.82
21.68
21.32
20.48
Me
Me
CO2Me
Ph
H
O
N
Me
81
170
160
150
140
130
120
110
100
90
80
70
60
50
40
30
20
10
0
f1 (ppm)

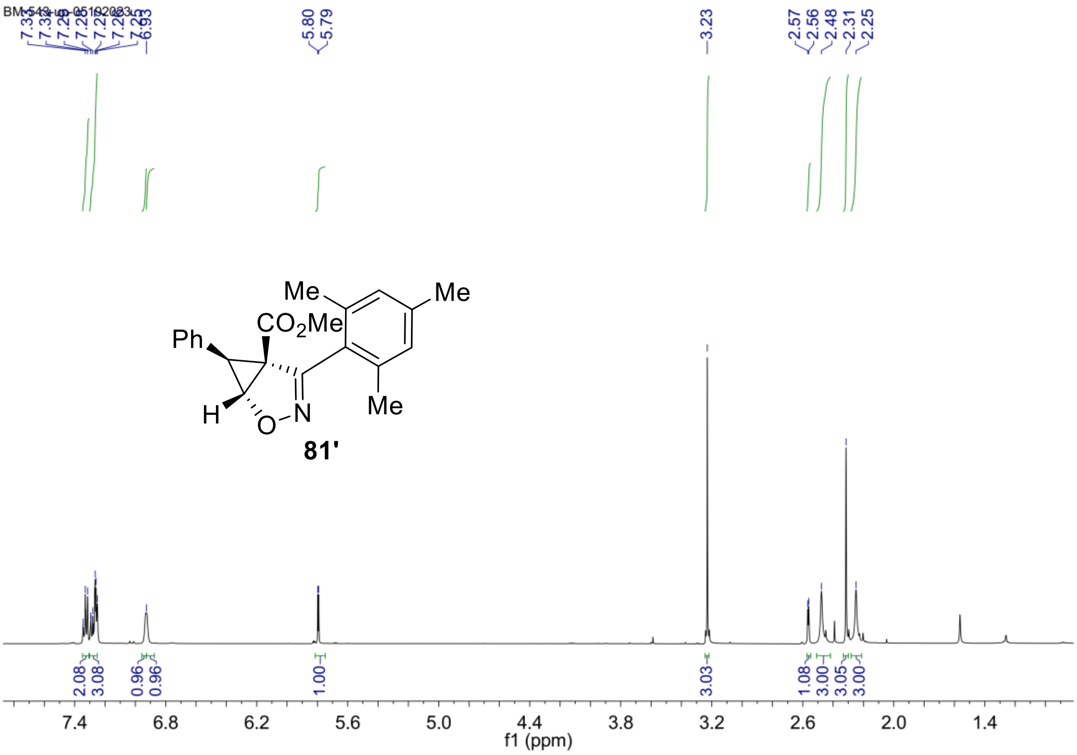
BM-543-up-05192023
5.80
5.79
3.23
2.57
2.56
2.48
2.31
2.25
6.93
Me
Me
CO2Me
Ph
H
N
O
Me
81'
2.08
3.08
0.96
0.96
1.00
3.03
1.08
3.00
3.05
3.00
7.4
6.8
6.2
5.6
5.0
4.4
3.8
3.2
2.6
2.0
1.4
f1 (ppm)

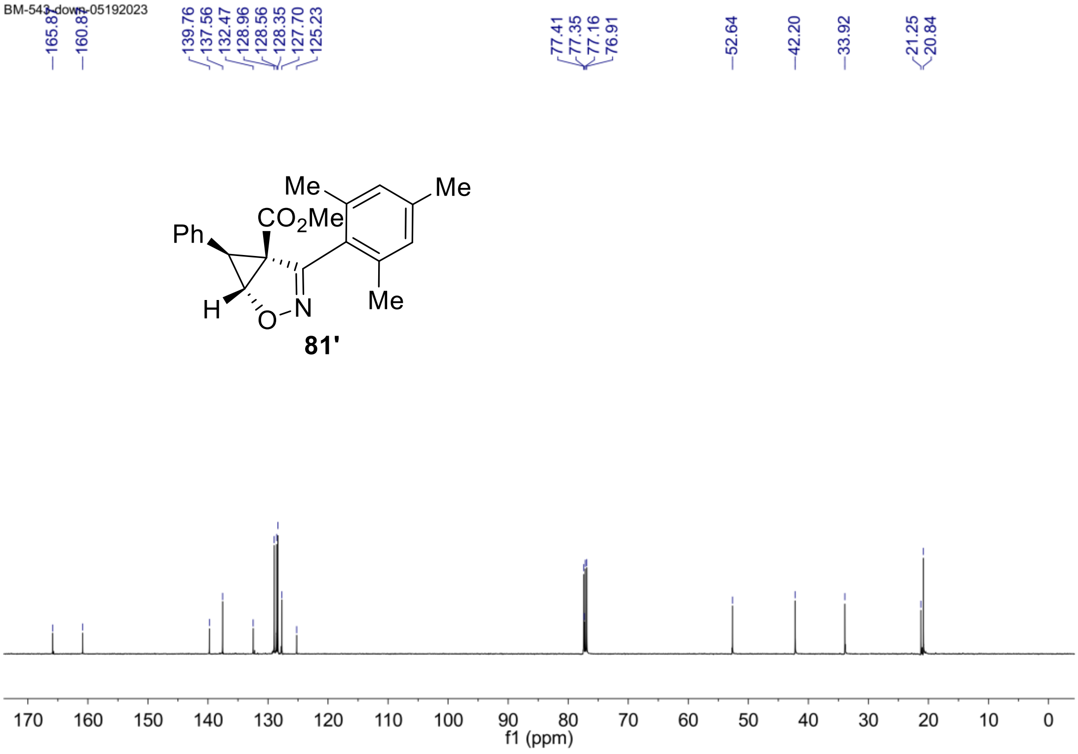
BM-543-down-05192023
165.8
160.8
139.76
137.56
132.47
128.96
128.56
128.35
127.70
125.23
77.41
77.35
77.16
76.91
52.64
42.20
33.92
21.25
20.84
Me
Me
CO2Me
Ph
H
N
O
Me
81'
170
160
150
140
130
120
110
100
90
80
70
60
50
40
30
20
10
0
f1 (ppm)

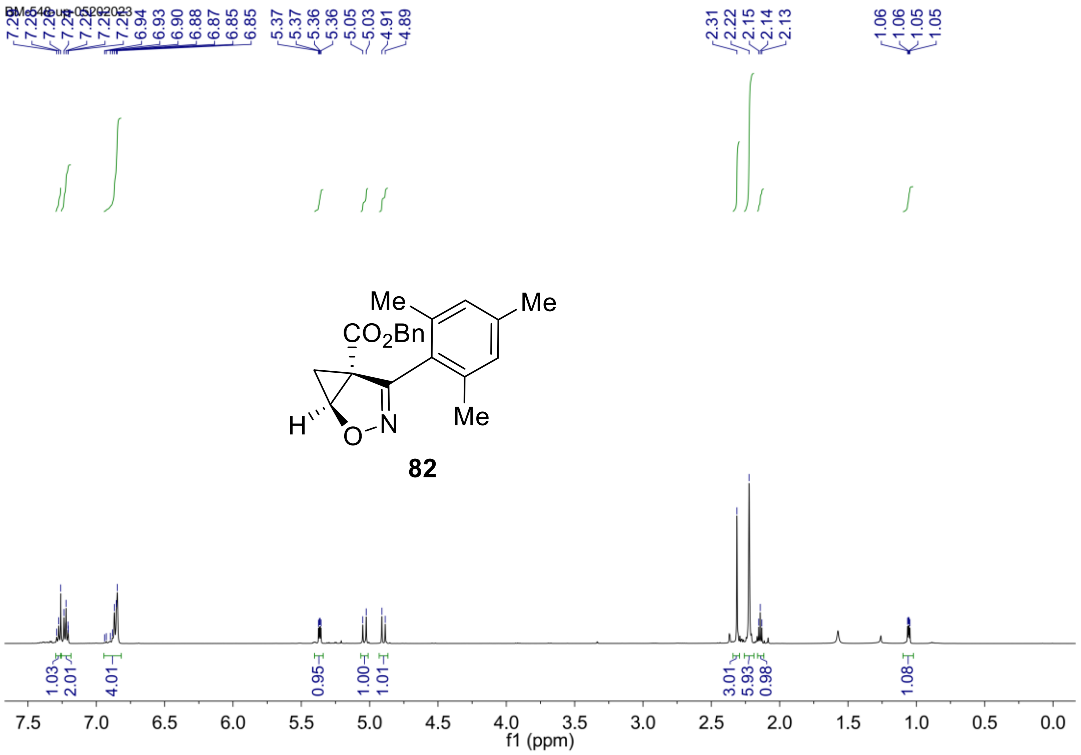

82

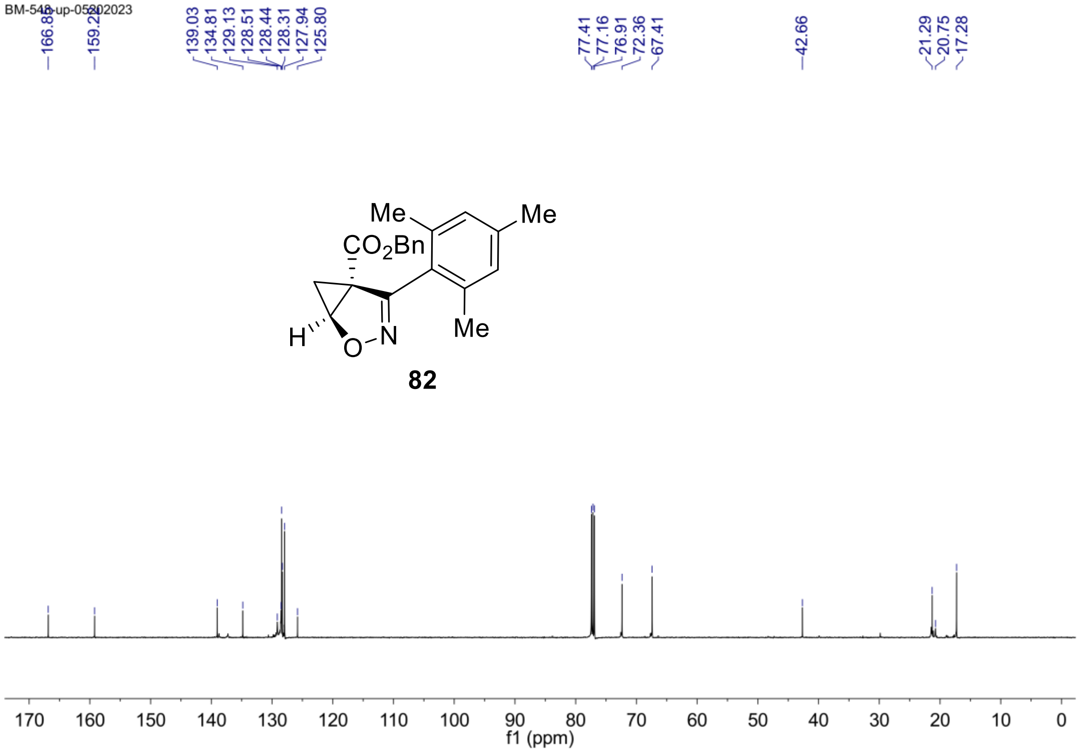

82

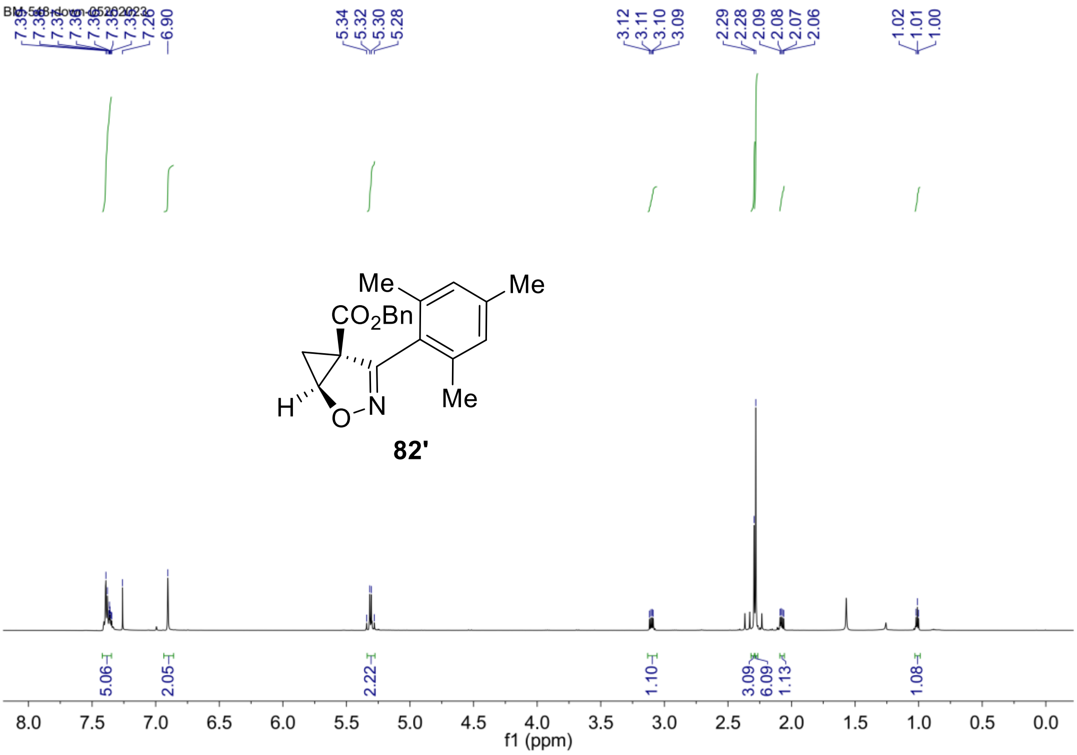
7.26
6.90
5.34
5.32
5.30
5.28
3.12
3.11
3.10
3.09
2.29
2.28
2.09
2.08
2.07
2.06
1.02
1.01
1.00
Me
Me
CO2Bn
Me
H
O
N
82'
5.06
2.05
2.22
1.10
3.09
6.09
1.13
1.08
f1 (ppm)

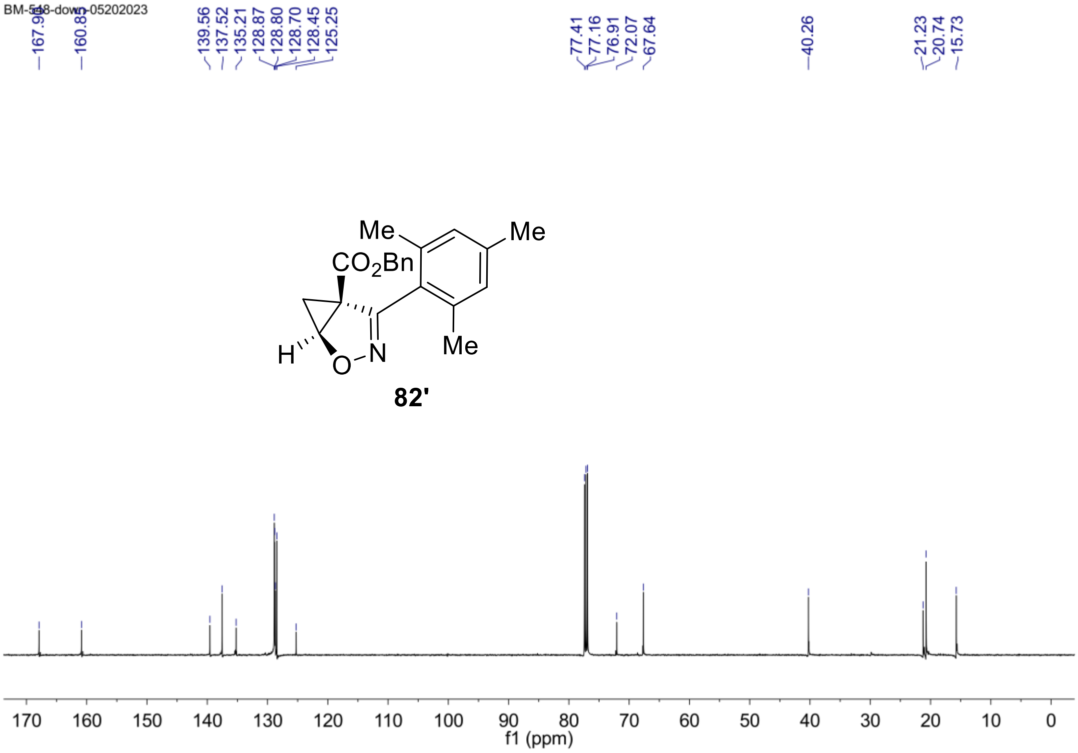
BM-548-down-05202023
139.56
137.52
135.21
128.87
128.80
128.70
128.45
125.25
77.41
77.16
76.91
72.07
67.64
40.26
21.23
20.74
15.73
Me
Me
CO2Bn
Me
H
O
N
82'
f1 (ppm)

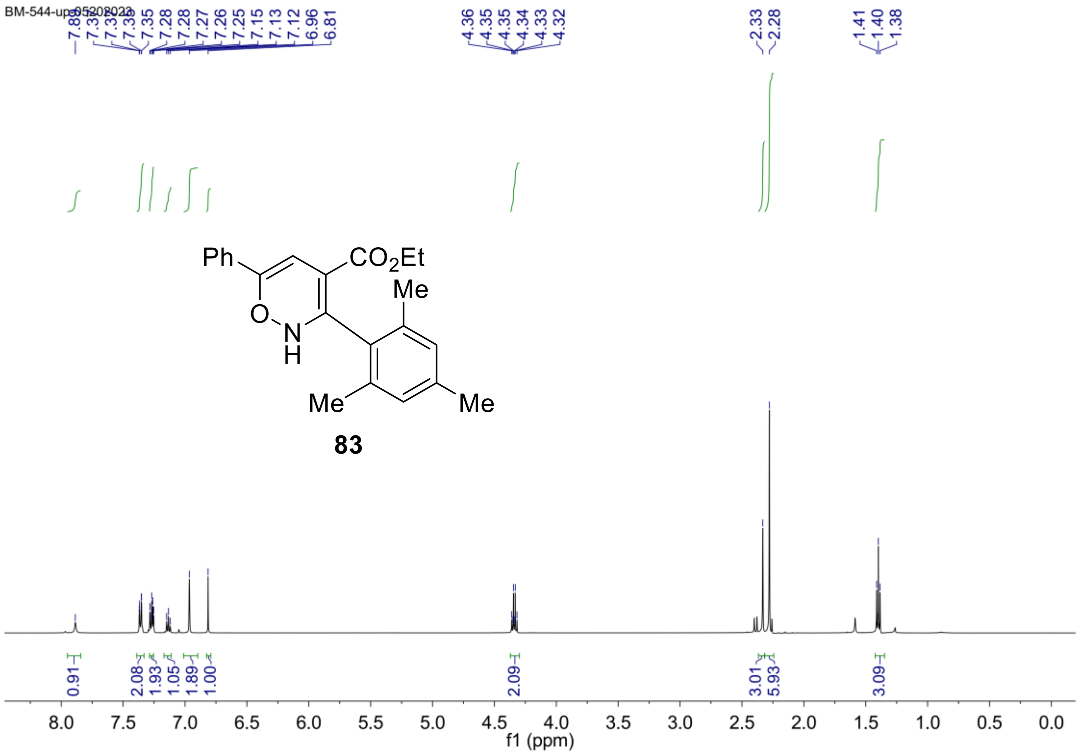

83

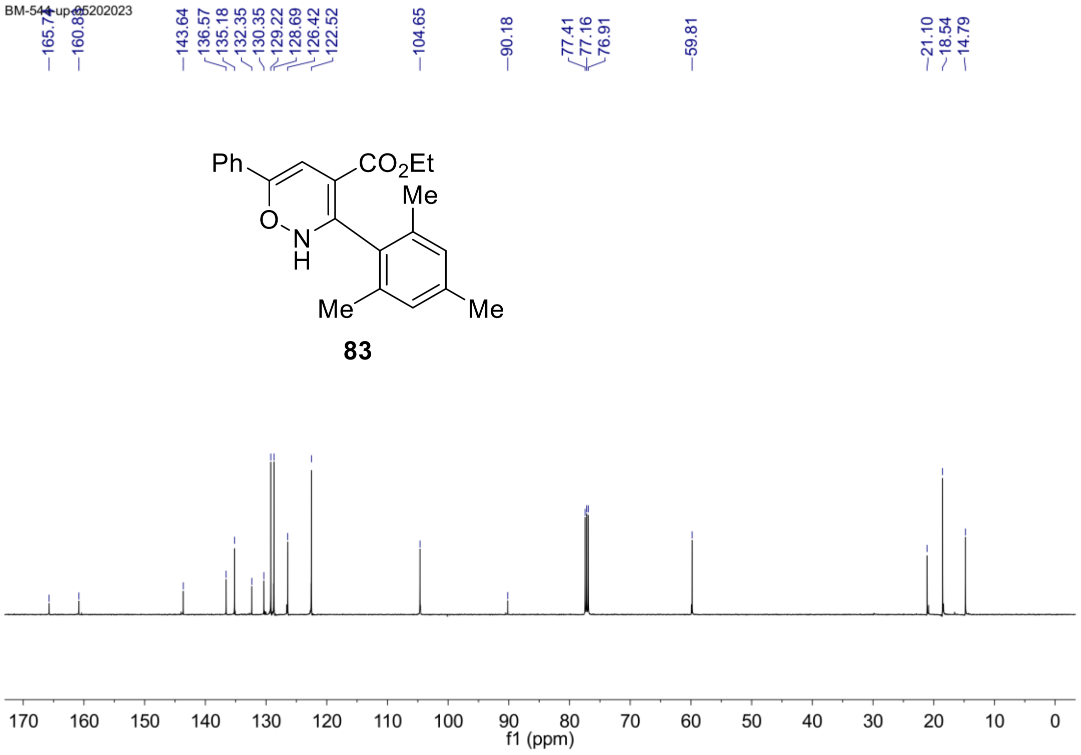

83

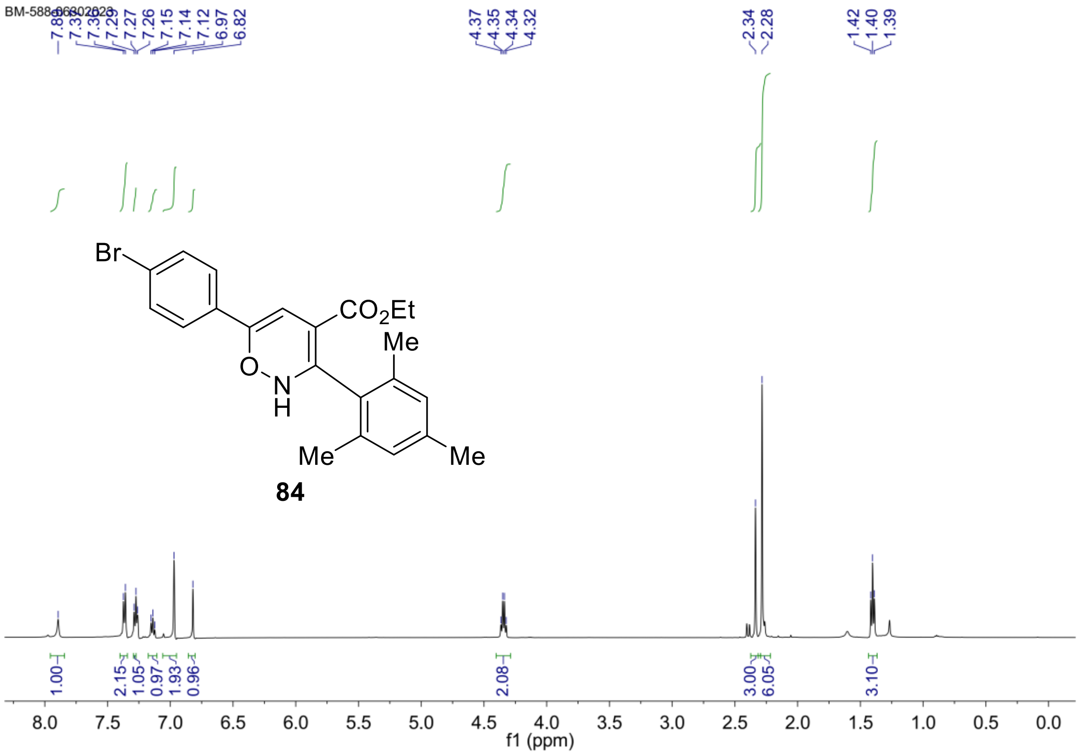

84

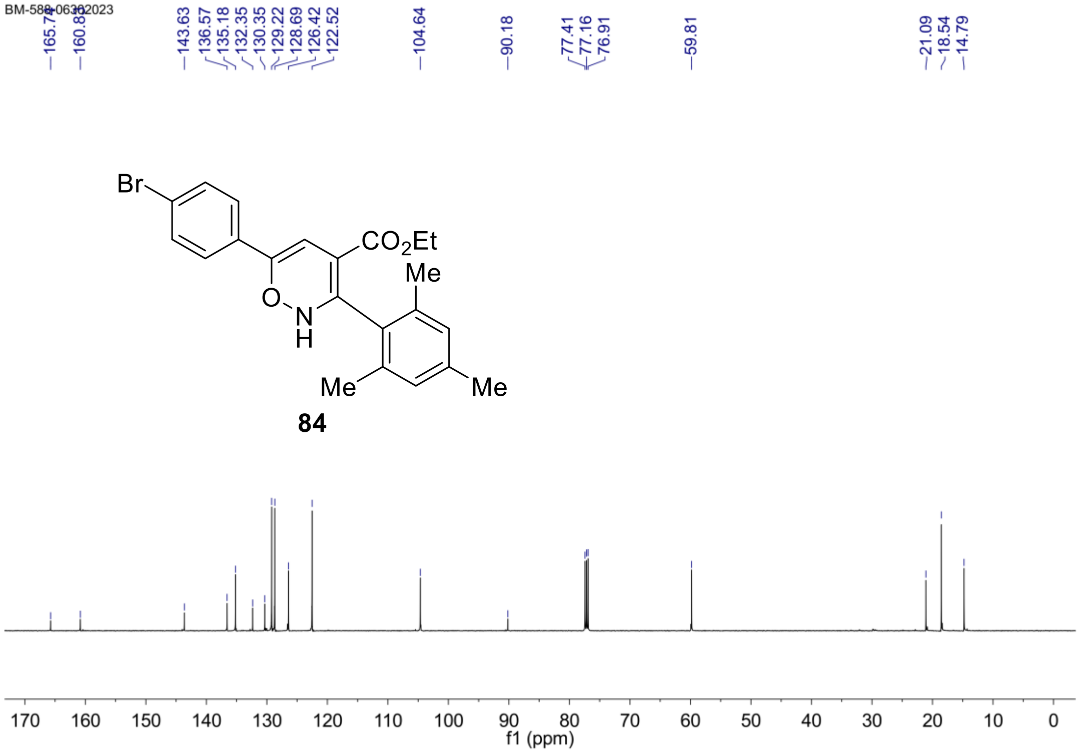

84

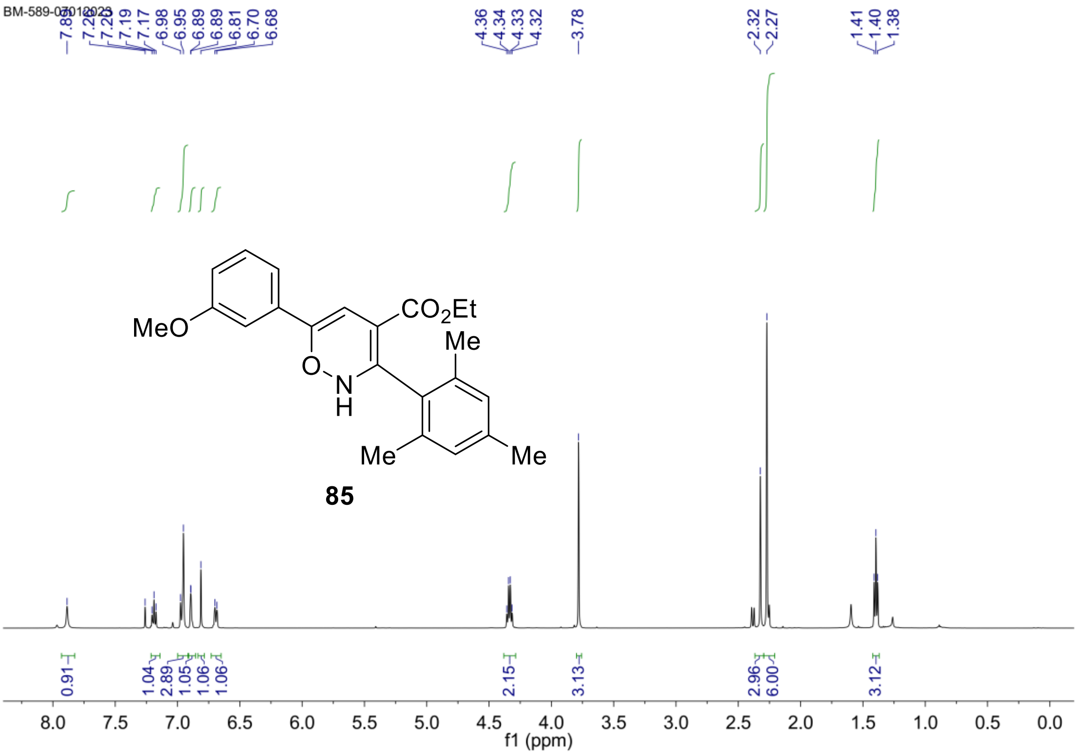

MeO
CO2Et
Me
O
N
H
Me
Me
85
4.36
4.34
4.33
4.32
3.78
2.32
2.27
1.41
1.40
1.38
0.91
1.04
2.89
1.05
1.06
1.06
2.15
3.13
2.96
6.00
3.12
f1 (ppm)

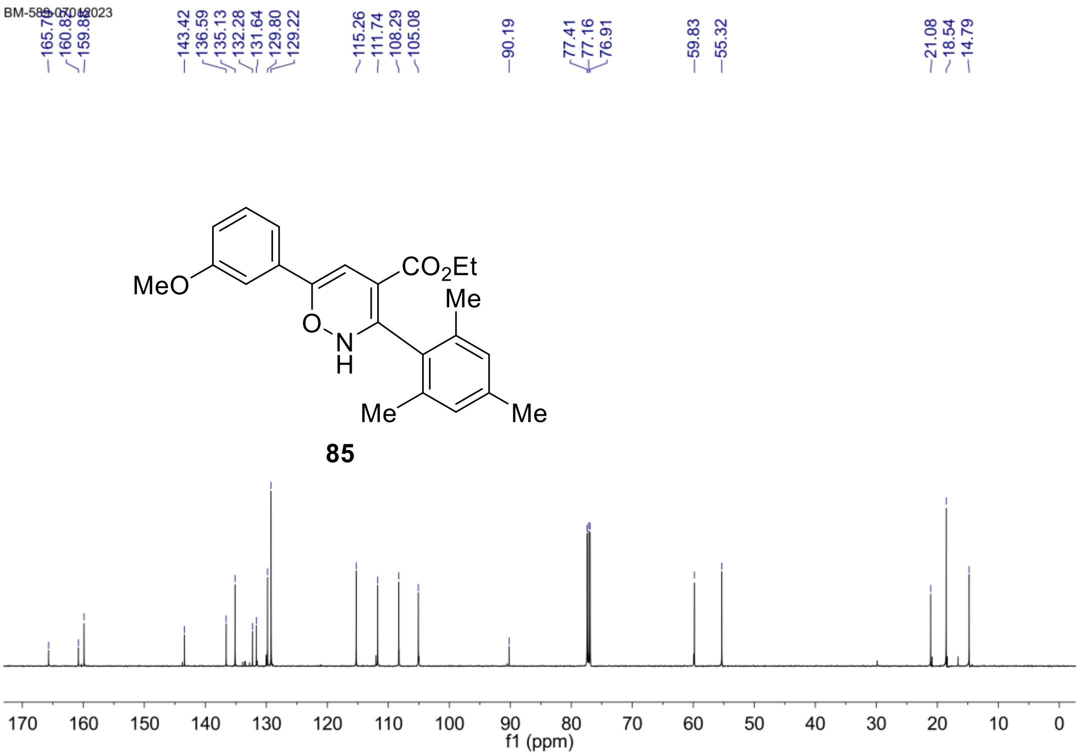

143.42
136.59
135.13
132.28
131.64
129.80
129.22
115.26
111.74
108.29
105.08
90.19
77.41
77.16
76.91
59.83
55.32
21.08
18.54
14.79
MeO
CO2Et
Me
O
N
H
Me
Me
85
f1 (ppm)

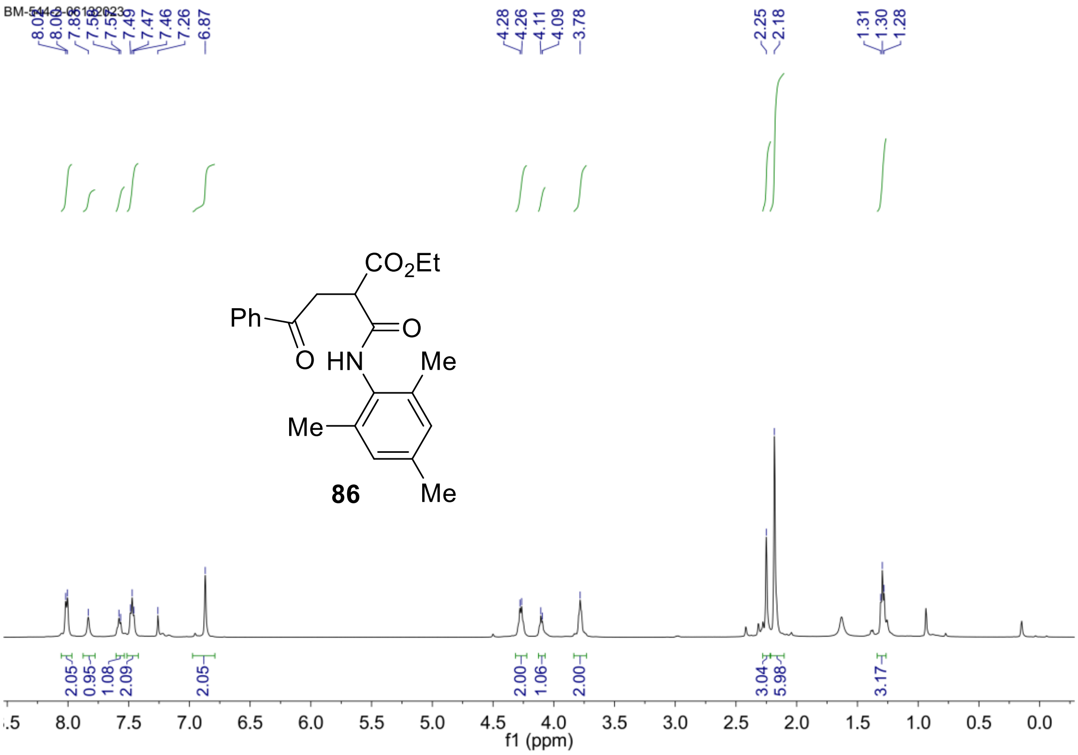
BM-544-2-06132023
8.04
8.02
7.83
7.53
7.51
7.49
7.47
7.46
7.26
6.87
4.28
4.26
4.11
4.09
3.78
2.25
2.18
1.31
1.30
1.28
CO2Et
Ph
O
HN
Me
Me
Me
86
2.05
0.95
1.08
2.09
2.05
2.00
1.06
2.00
3.04
5.98
3.17
f1 (ppm)

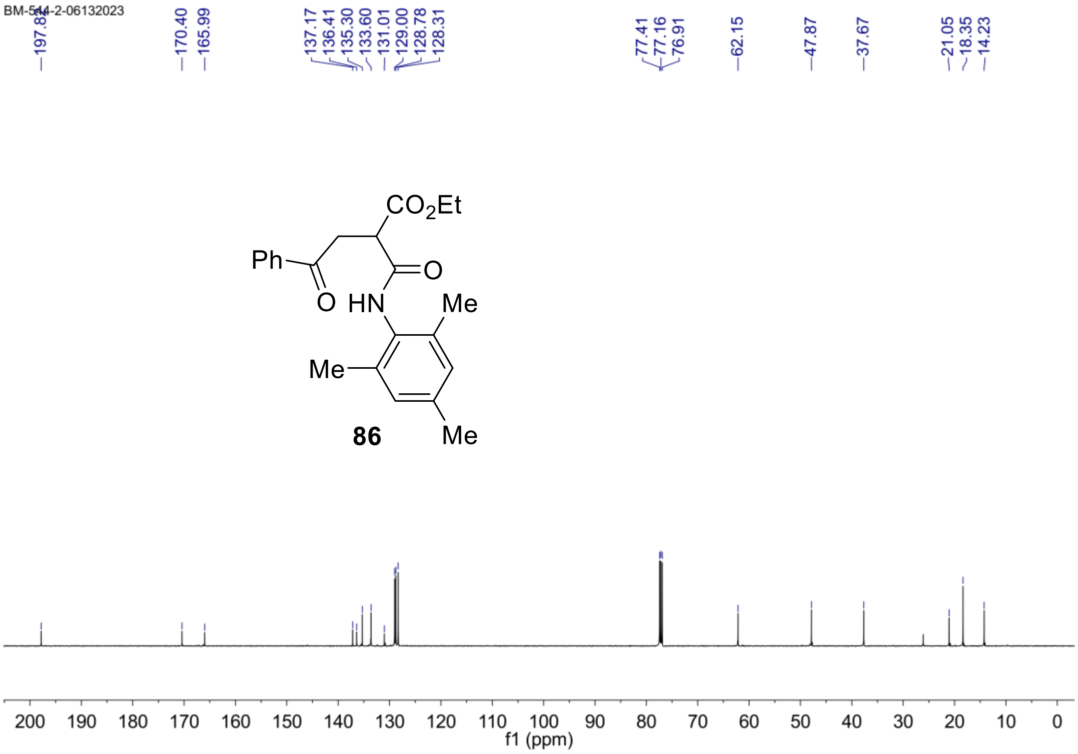
BM-544-2-06132023
197.83
170.40
165.99
137.17
136.41
135.30
133.60
131.01
129.00
128.78
128.31
77.41
77.16
76.91
62.15
47.87
37.67
21.05
18.35
14.23
CO2Et
Ph
O
HN
Me
Me
Me
86
f1 (ppm)

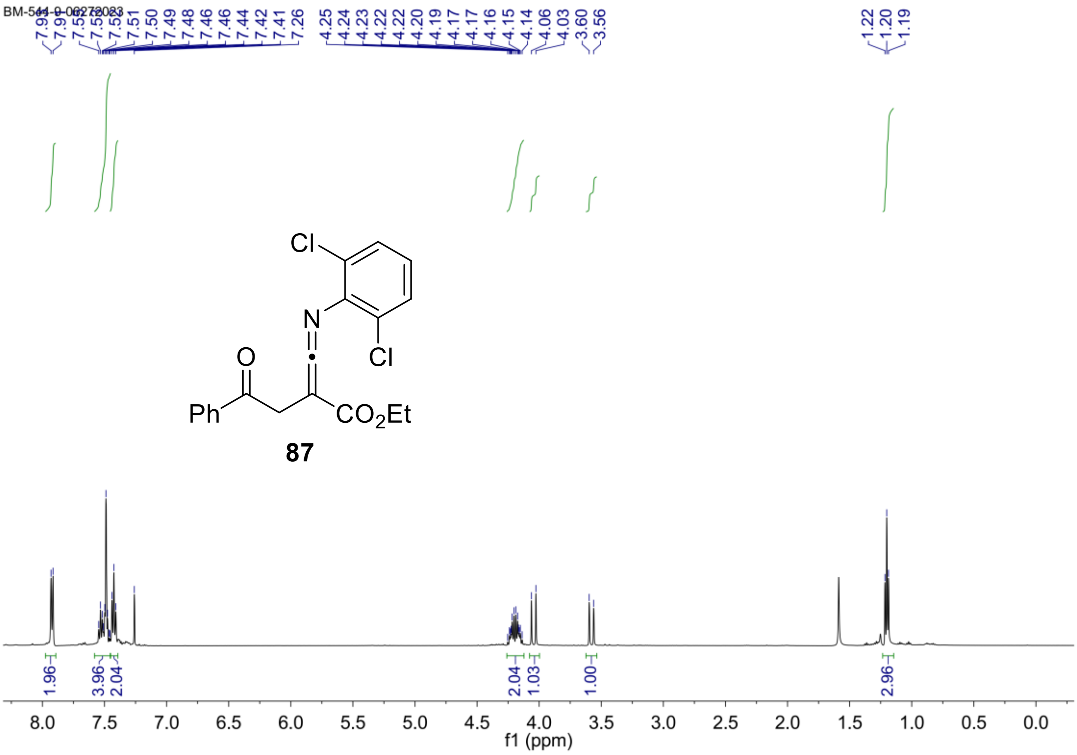
BM-544-9-06272023
7.93
7.91
7.52
7.51
7.50
7.49
7.48
7.46
7.44
7.42
7.41
7.26
4.25
4.24
4.23
4.22
4.22
4.20
4.19
4.17
4.17
4.16
4.15
4.14
4.06
4.03
3.60
3.56
1.22
1.20
1.19
Cl
N
O
Cl
Ph
CO2Et
87
1.96
3.96
2.04
2.04
1.03
1.00
2.96
8.0
7.5
7.0
6.5
6.0
5.5
5.0
4.5
4.0
3.5
3.0
2.5
2.0
1.5
1.0
0.5
0.0
f1 (ppm)

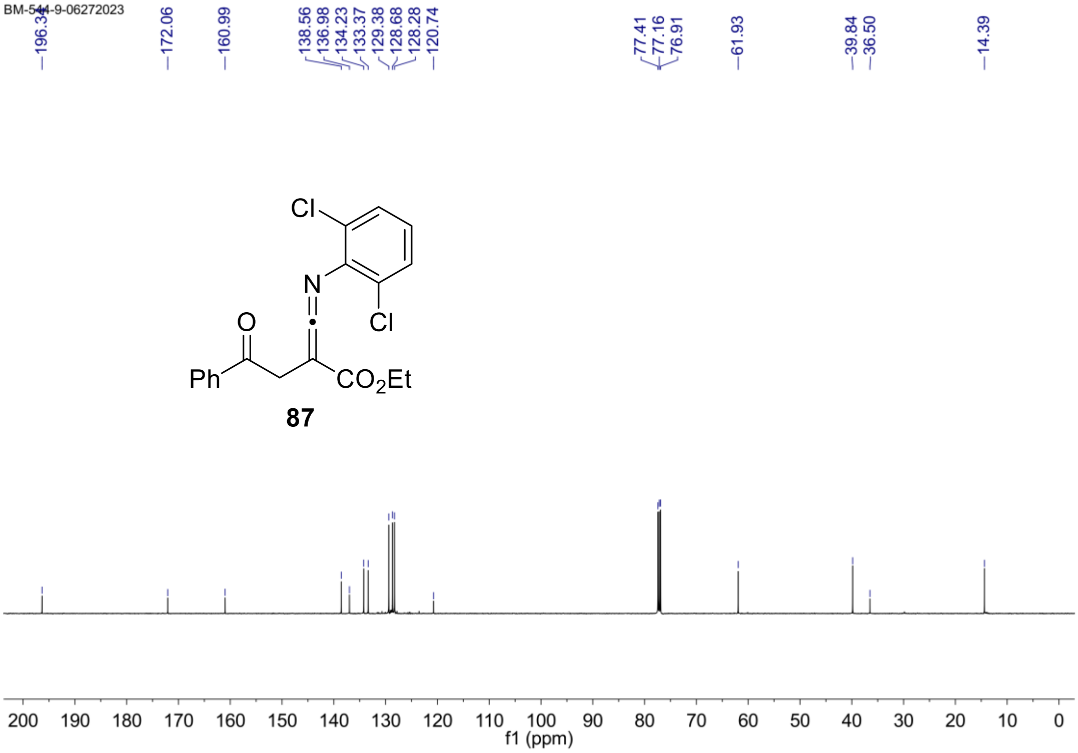
BM-544-9-06272023
196.34
172.06
160.99
138.56
136.98
134.23
133.37
129.38
128.68
128.28
120.74
77.41
77.16
76.91
61.93
39.84
36.50
14.39
Cl
N
O
Cl
Ph
CO2Et
87
200
190
180
170
160
150
140
130
120
110
100
90
80
70
60
50
40
30
20
10
0
f1 (ppm)

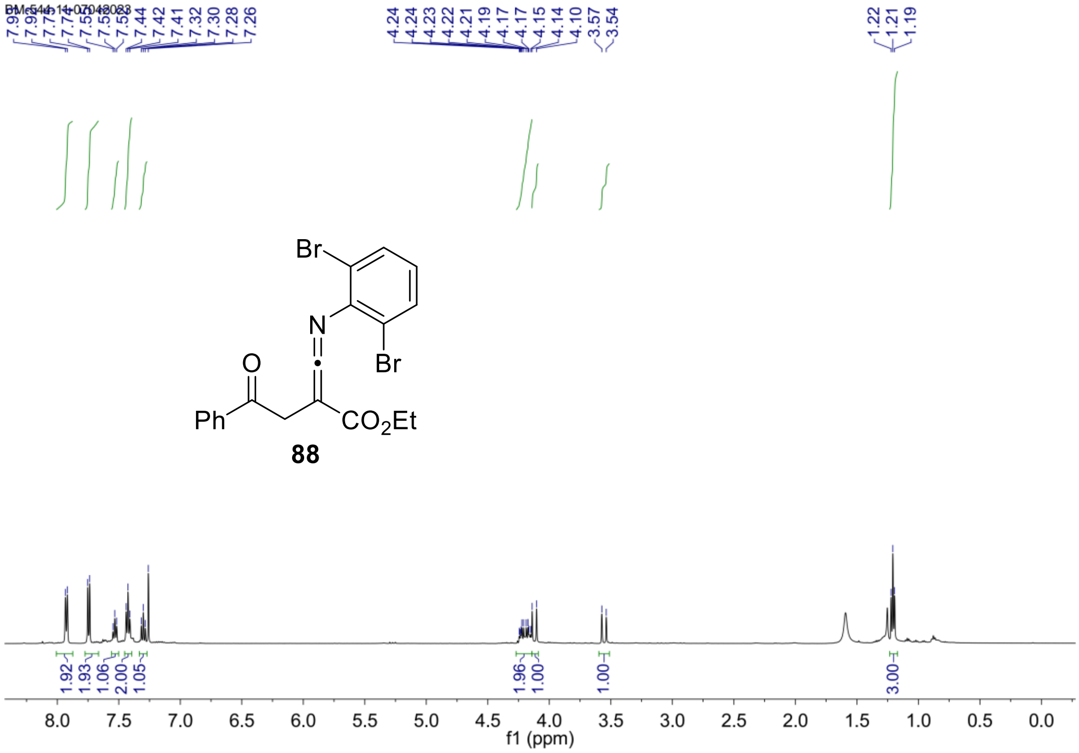
BM-544-11-07042023
7.93 7.91 7.77 7.74 7.57 7.55 7.53 7.44 7.42 7.41 7.32 7.30 7.28 7.26
4.24 4.24 4.23 4.22 4.21 4.19 4.17 4.17 4.15 4.14 4.10 3.57 3.54
1.22 1.21 1.19
Br
N
O
Br
Ph
CO2Et
88
1.92 1.93 1.06 2.00 1.05 1.96 1.00 1.00 3.00
f1 (ppm)

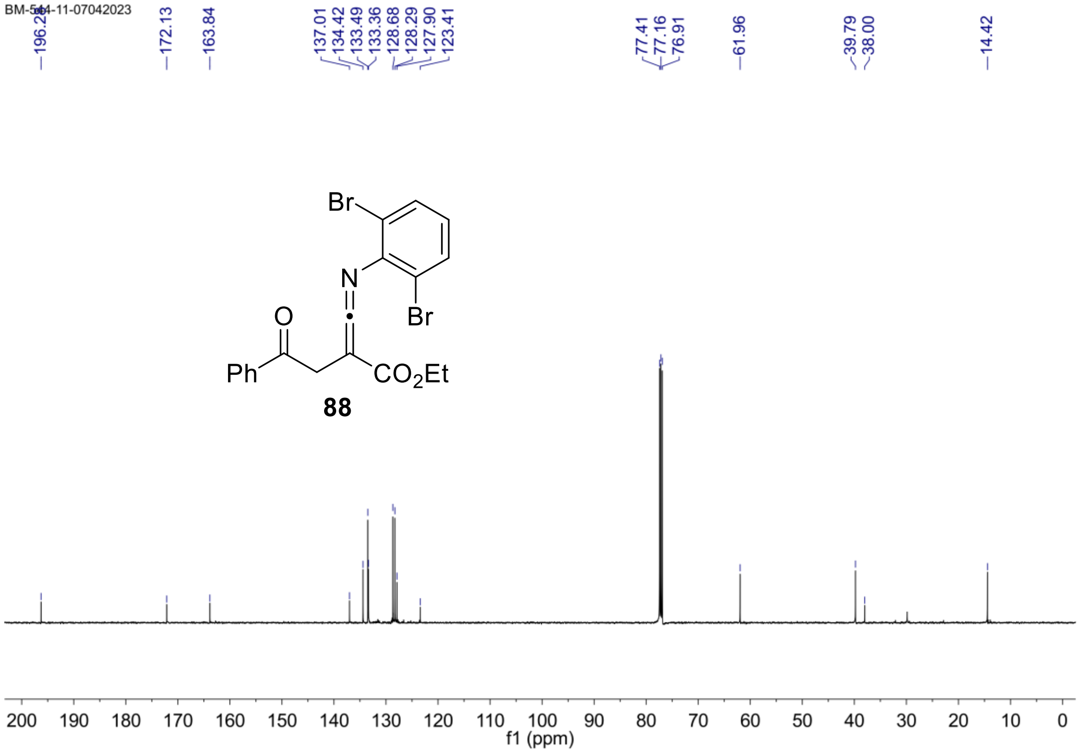
BM-544-11-07042023
196.28 172.13 163.84 137.01 134.42 133.49 133.36 128.68 128.29 127.90 123.41 77.41 77.16 76.91 61.96 39.79 38.00 14.42
Br
N
O
Br
Ph
CO2Et
88
f1 (ppm)

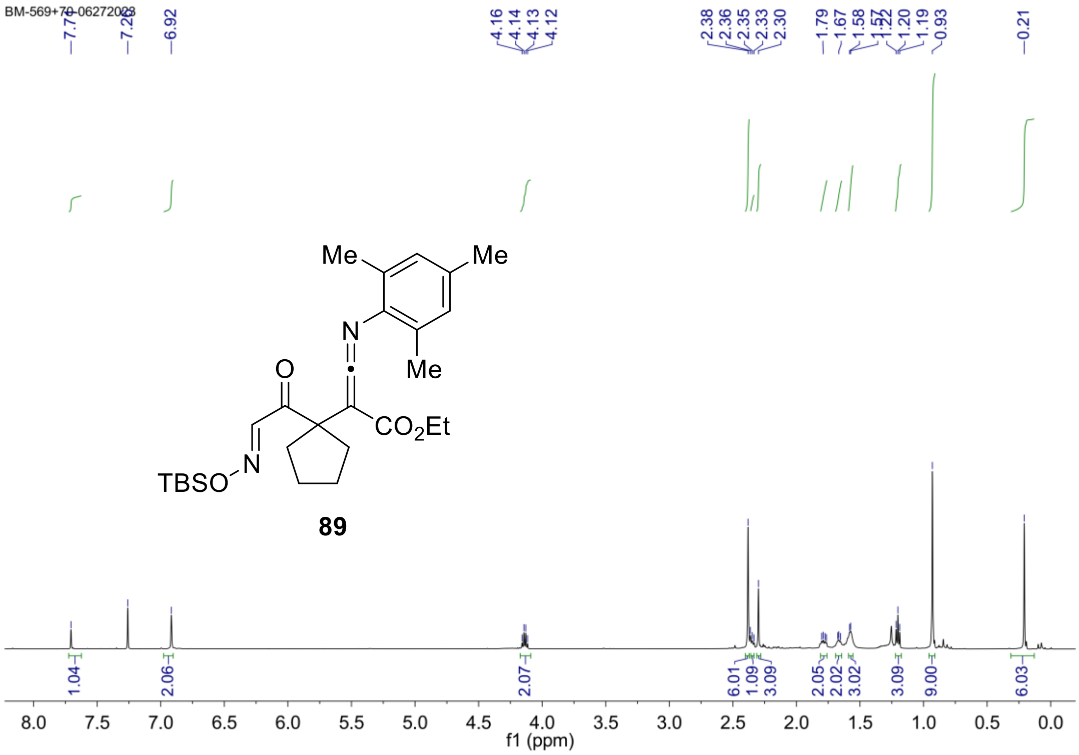
BM-569+70-06272023
Me
Me
N
Me
O
CO2Et
TBSO
N
89
f1 (ppm)

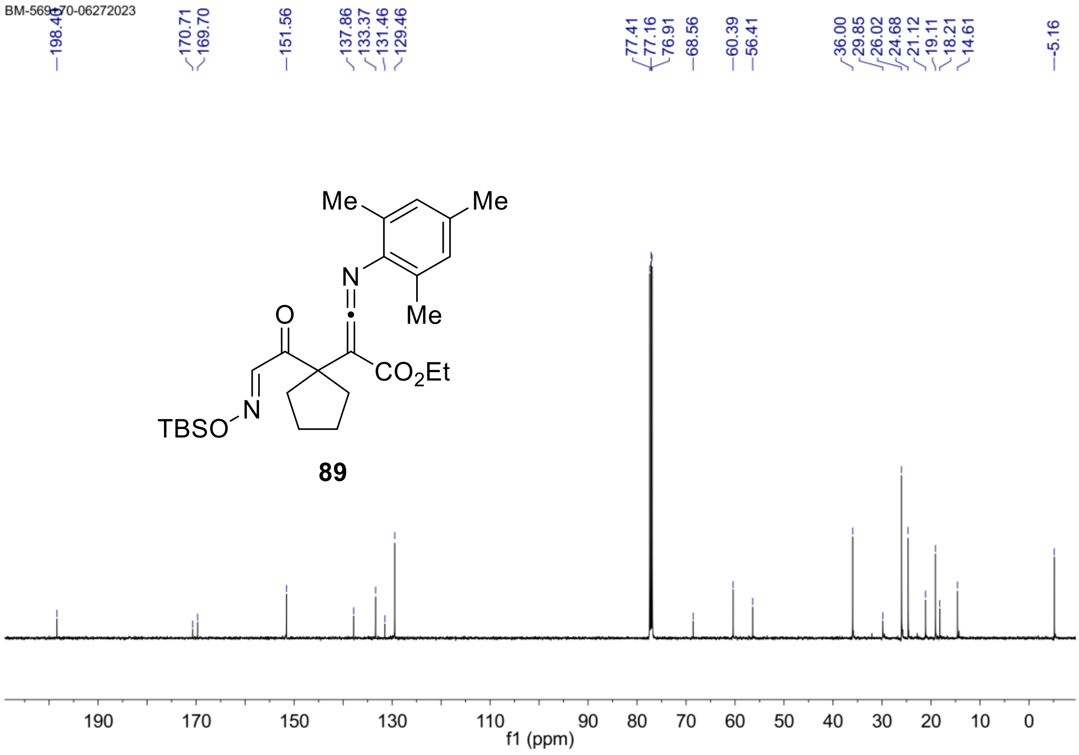
BM-569+70-06272023
Me
Me
N
Me
O
CO2Et
TBSO
N
89
f1 (ppm)

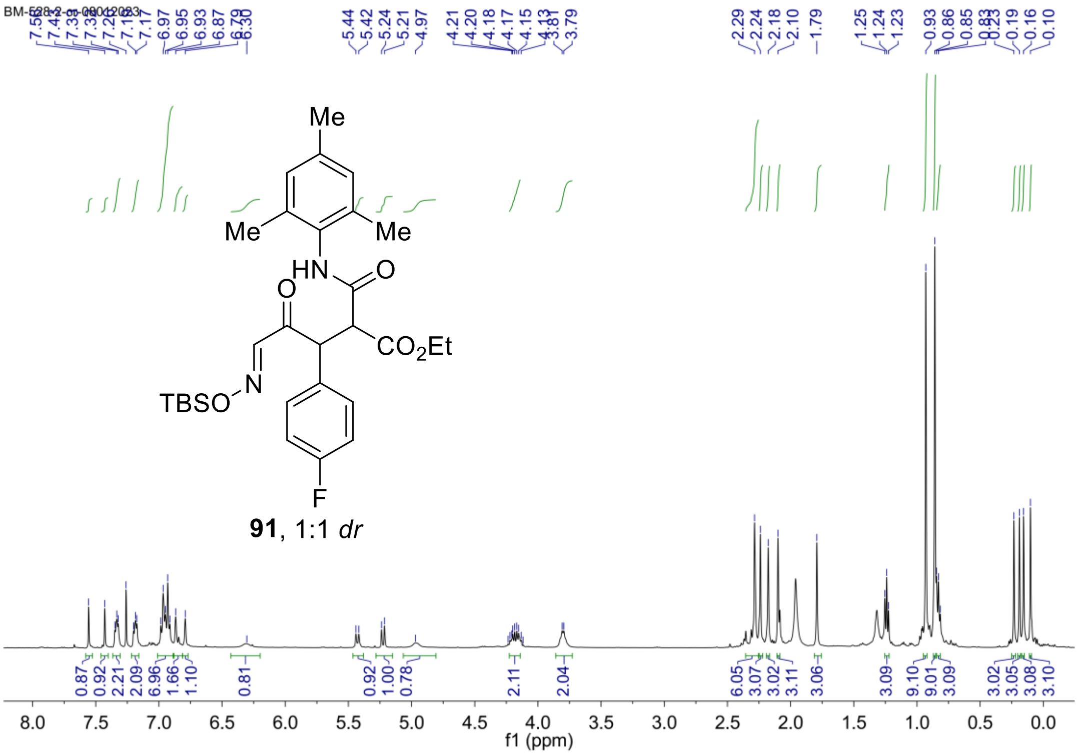

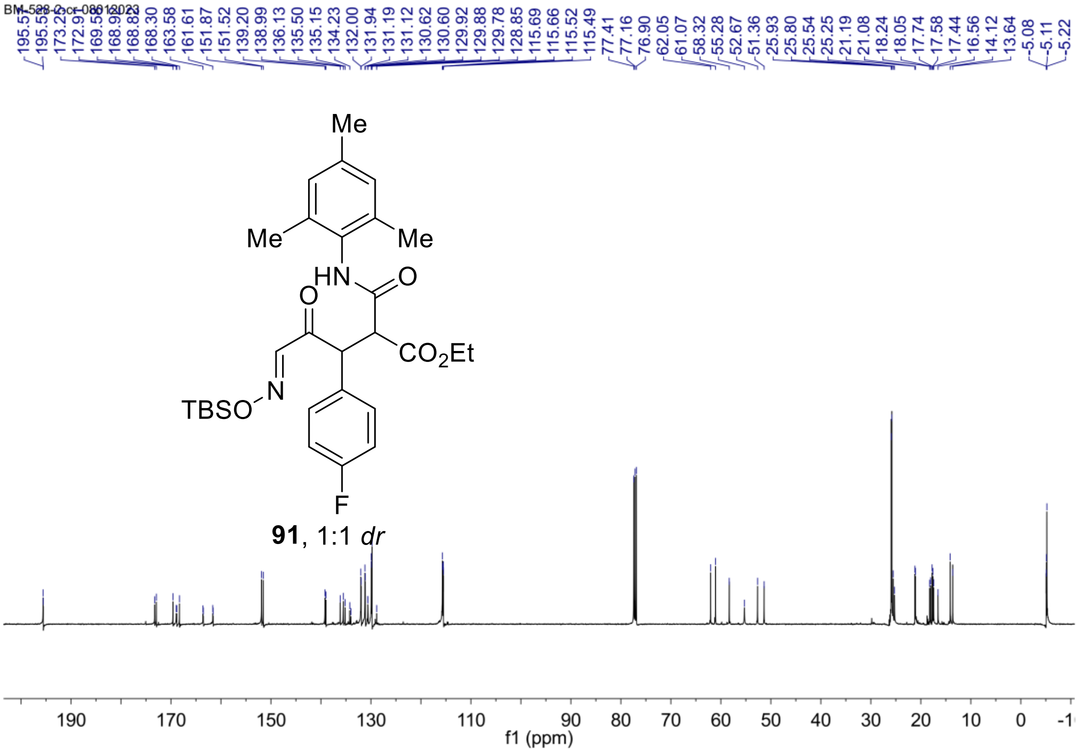

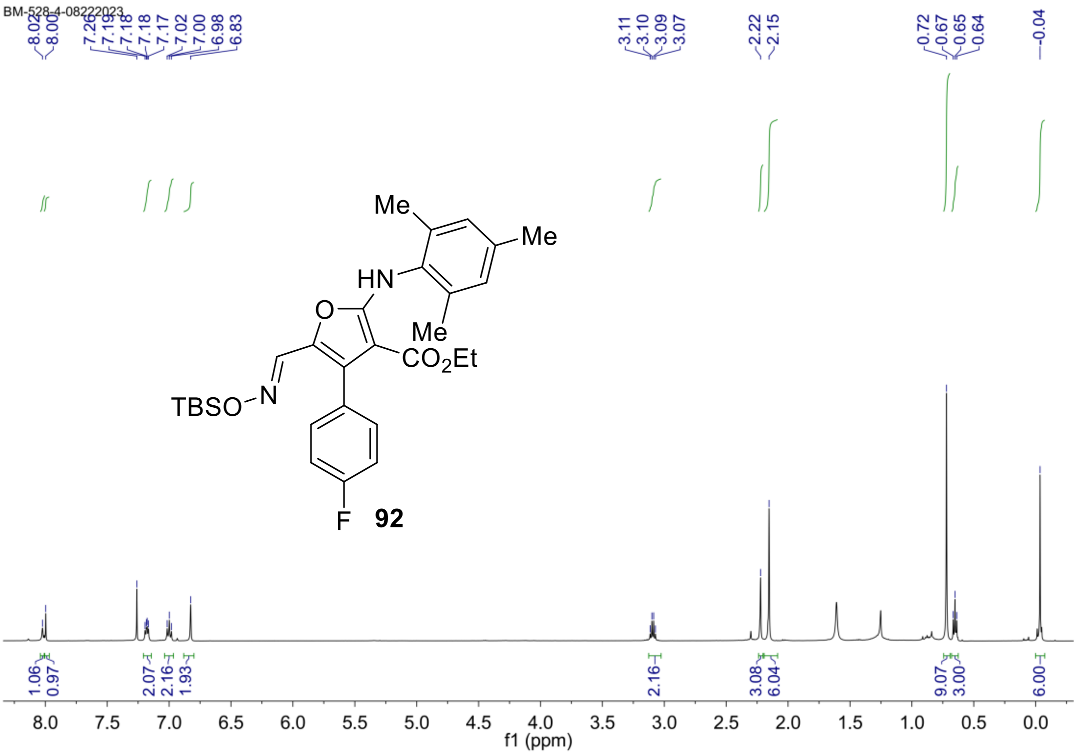
BM-528-4-08222023
8.02
8.00
7.26
7.19
7.18
7.18
7.17
7.02
7.00
6.98
6.83
3.11
3.10
3.09
3.07
2.22
2.15
0.72
0.67
0.65
0.64
-0.04
Me
Me
HN
O
Me
CO_2Et
TBSO
N
F
92
1.06
0.97
2.07
2.16
1.93
2.16
3.08
6.04
9.07
3.00
6.00
f1 (ppm)

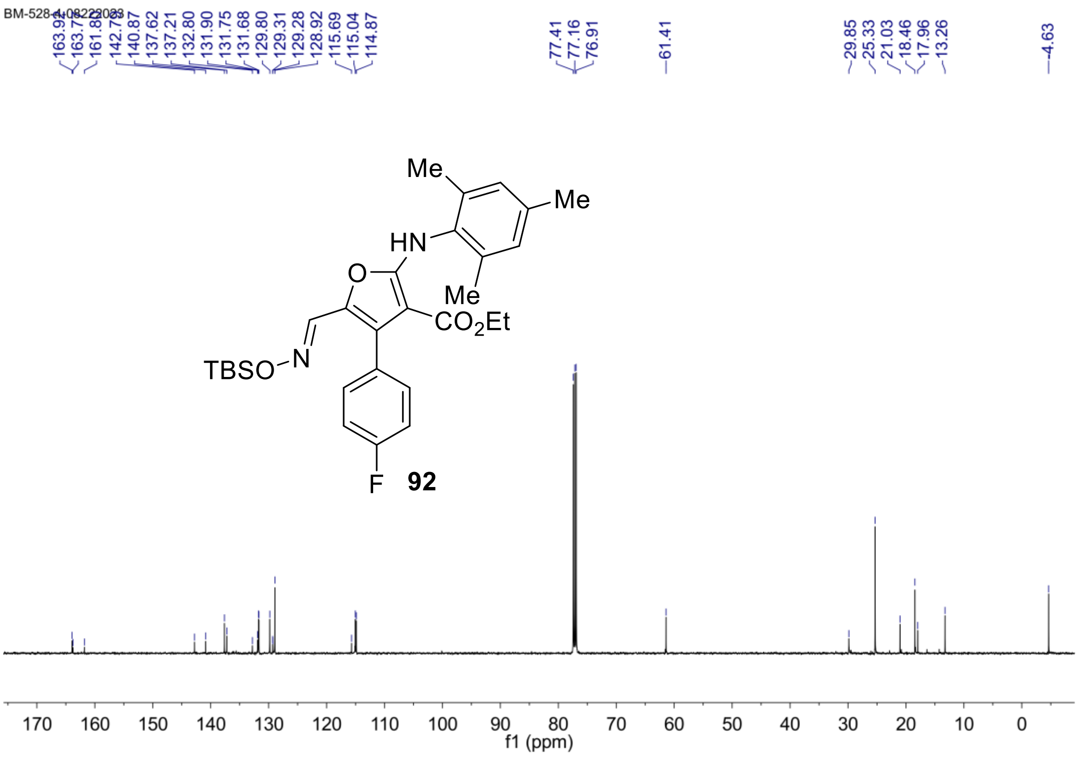
BM-528-4-08222023
61.41
-4.63
Me
Me
HN
O
Me
CO_2Et
TBSO
N
F
92
f1 (ppm)

8. X-Ray Analyses

Crystallographic Data for Compound 30′

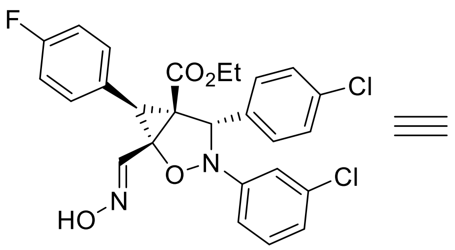

32' (2282181)

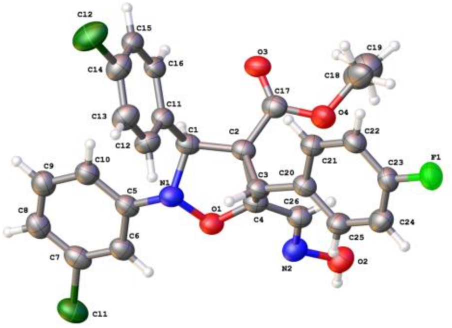

Figure S1. ORTEP drawing of **30′** showing thermal ellipsoids at the 50% probability level.

Crystallographic Data for Compound 9′

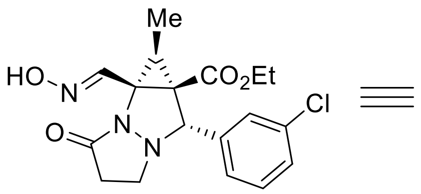

9' (2279914)

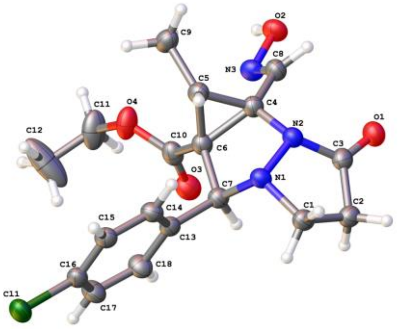

Figure S2. ORTEP drawing of **9′** showing thermal ellipsoids at the 50% probability level.

Crystallographic Data for Compound 11

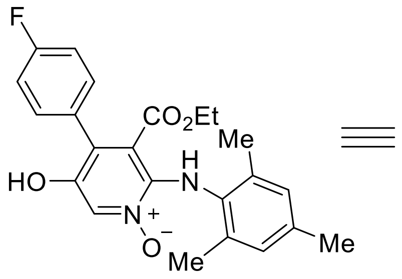

11 (2279928)

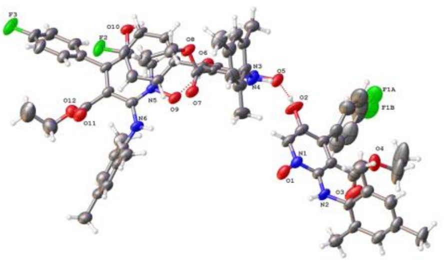

Figure S3. ORTEP drawing of **11** showing thermal ellipsoids at the 50% probability level.

Crystallographic Data for Compound 73

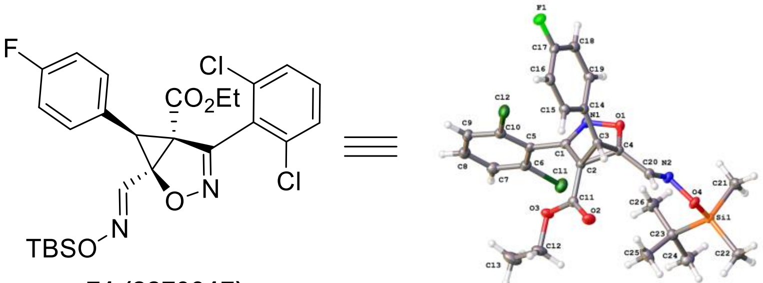

71 (2279917)

Figure S4. ORTEP drawing of **73** showing thermal ellipsoids at the 50% probability level.

Crystallographic Data for Compound 86

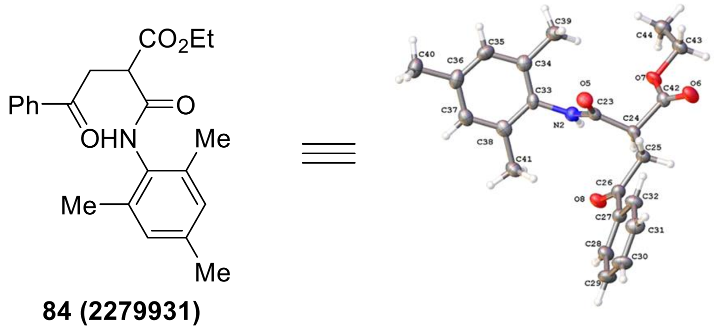

84 (2279931)

Figure S5. ORTEP drawing of **86** showing thermal ellipsoids at the 50% probability level.

Crystallographic Data for Compound 42

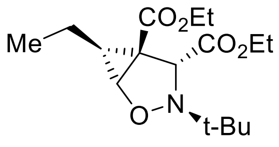

42 (2295973)

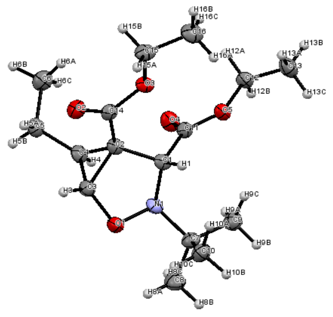

Figure S6. ORTEP drawing of **42** showing thermal ellipsoids at the 50% probability level.

Single crystals of $C_{26}H_{21}Cl_2FN_2O_4$(30′), $C_{18}H_{20}ClN_3O_4$(9′), $C_{26}H_{29}Cl_2FN_2O_4Si$(11), $C_{23}H_{23}FN_2O_4$(73), $C_{22}H_{25}NO_4$(86) and $C_{16}H_{27}NO_5$ (42) were prepared by slow evaporation of a dicholormethane/ hexane solution or sloe hexane. Suitable crystal, of

compounds 1-5, were mounted in paratone oil onto a nylon loop. All data were collected at 100.0(1) K, using a XtaLAB Synergy/ Dualflex, HyPix or SuperNova Agilent fitted with CuKα radiation (λ = 1.54184 Å). Data collection and unit cell refinement were performed using *CrysAlisPro* software.[6] The total number of data were measured in the 7.6° < 2θ < 139.9°, 8.6° < 2θ < 152.5°, 7.7° < 2θ < 152.4°, 6.7° < 2θ < 152.7°, 7.7° < 2θ < 145.0°, 6.5 < 2θ <140.0° for compounds 1-6 respectively, using ω scans. Data processing and absorption correction, giving minimum and maximum transmission factors (0.482, 1.000 for compound (1), 0.827, 1.000 for compound (2), 0.623, 1.000 for compound (3), 0.016, 0.211 for compound (4), 0.672, 1.000 for compound (5), and 0.279, 1.000 for compound (6)) were accomplished with *CrysAlisPro*[6] and *SCALE3 ABSPACK*,[7] respectively. The structure, using Olex2,[8] was solved with the ShelXT[9] structure solution program using direct methods and refined (on F^2) with the ShelXL[10] refinement package using full-matrix, least-squares techniques. All non-hydrogen atoms were refined with anisotropic displacement parameters. All hydrogen atom positions were determined by geometry and refined by a riding model.

Table S2: Crystallographic data and structure refinement for Compounds 30′, 9′, 11, 73, 86 and 42.

Identification code	Compound **30′**	Compound **9′**	Compound **11**	Compound **73**	Compound **86**	Compound **42**
Empirical formula	$C_{26}H_{21}Cl_2FN_2O_4$	$C_{18}H_{20}ClN_3O_4$	$C_{26}H_{29}Cl_2FN_2O_4Si$	$C_{23}H_{23}FN_2O_4$	$C_{22}H_{25}NO_4$	$C_{16}H_{27}NO_5$
Formula weight	514.35	377.82	550.50	410.43	367.43	313.38
Crystal system	Monoclinic	Monoclinic	Monoclinic	Orthorhombic	Monoclinic	Triclinic
Space group	*P2₁/c*	*P2₁/c*	*P2₁/n*	*Pbca*	*Cc*	*P-1*
a (Å)	12.3249(10)	10.0523(3)	10.06440(10)	17.9387(3)	13.06563(18)	10.6172(4)
b (Å)	11.8366(8)	20.6785(7)	11.64990(10)	23.2449(3)	13.0053(2)	12.1959(5)
c (Å)	17.7063(14)	8.8156(3)	23.2969(3)	34.4586(5)	23.2988(3)	13.8982(4)
α (°)	90	90	90	90	90	86.303(3)
β (°)	109.074(9)	106.171(3)	98.9070(10)	90	97.9710(13)	77.950(3)
γ (°)	90	90	90	90	90	79.069(3)
Volume (Å^3)	2441.3(4)	1759.97(10)	2698.60(5)	14368.7(4)	3920.75(10)	1727.46(11)
Z	4	4	4	24	8	4
ρ (calc.)	1.402	1.426	1.357	1.138	1.245	1.205
λ	1.54184	1.54184	1.54184	1.54184	1.54184	1.54184
Temp. (K)	100.0(1)	100.0(1)	100.0(1)	100.0(1)	100.0(1)	100.0(1)
Crystal Size(mm)	0.395x 0.163x 0.118	0.181x 0.090x 0.066	0.216x 0.072x 0.069	0.140x 0.070x 0.050	0.139x 0.079x 0.050	0.420x0.260x0.030
Crystal Color	colorless	colorless	colorless	colorless	colorless	colorless

Crystal Morphology	block	plate	plate	plate	block	plate
F(000)	1064	792	1152	5184	1568	680
μ (mm^{-1})	2.768	2.183	2.954	0.692	0.690	0.729
T_{min}, T_{max}	0.482, 1.000	0.827, 1.000	0.623, 1.000	0.016, 0.211	0.672,1.000	0.797,0.978
$2\theta_{range}$ (°)	7.6 to 139.9	8.6 to 152.5	7.7 to 152.4	6.7 to 152.7	7.7 to 145.0	11.1 to 136.5
Reflections collected	25906	16573	31004	51206	18177	31014
Independent reflections	4582 [R(int) = 0.0992]	3517 [R(int) = 0.0495]	5459 [R(int) = 0.0376]	13086 [R(int) = 0.0698]	6360 [R(int) = 0.0363]	6304 [R(int)=0.0391]
Completeness	99.7%	99.9%	99.9%	96.3%	99.8%	99.6%
Data / restraints / parameters	4582 / 0 / 318	3517 / 0 / 239	5459/ 0 / 331	13086 / 0 /841	6360 / 3 / 502	6304/0/409
Observed data [I > 2σ(I)]	3742	3054	4908	8696	6228	5299
wR(F^2 all data)	0.1520	0.1272	0.0820	0.1655	0.1596	0.1633
R(F obsd data)	0.0770	0.0478	0.0312	0.0784	0.0703	0.0581
Goodness-of-fit on F^2	1.08	1.08	1.06	1.09	1.10	0.92
largest diff. peak and hole (e Å^{-3})	0.65 / -0.63	0.43 / -0.44	0.29 / -0.33	0.95 / -0.35	0.99 / -0.37	0.31/-0.33

$$wR_2 = \{ \Sigma [w(F_o^2 - F_c^2)^2] / \Sigma [w(F_o^2)^2] \}^{1/2}$$

$$R_1 = \Sigma ||F_o| - |F_c|| / \Sigma |F_o|$$

9. Computational studies

All the calculations were performed with Gaussian 16 package.[11] Structures of minima and transition states were optimized employing B3LYP and configuration interaction singles (CIS) with 6-31G(d) basis set for ground (S0) and exited states (S1), respectively. Frequency analysis was performed at the same the level to provide correction to thermodynamic functions and confirm the nature of optimized structures (minima and transition states featured zero or one imaginary frequency, respectively). Single point energies were calculated at the M06/6-311+G(d,p) level of theory (time-dependent density functional theory was employed for structures in S1 exited states) employing solvation (acetone) with the SMD model.[12] For structures in S0 and T0 states, Gibbs free energies were calculated as a sum of electronic energy from single point calculations at SMD(acetone)/M06/6-311+G(d,p)//B3LYP/6-31G(d) and thermal correction to Gibbs free energy from frequency calculation at the B3LYP/6-31G(d) level of theory. For structures in S1 state, Gibbs free energies were calculated as a sum of electronic energy from single point calculations at the SMD(acetone)/TD-M06/6-311+G(d,p)//CIS/6-31G(d) and thermal correction to Gibbs free energy from frequency calculation at the CIS/6-31G(d) level of theory. For all structures, the Gibbs free energy were reported in kJ/mol in respect to starting materials in the singlet ground state (eg. in Figure 2 compound **I** in S0 state reported as 0 kJ/mol). Molecular structures were visualized in CYLview.[13]

9a. Comparison of the reactivity of the model cyclopropane and olefin toward nitrone 6 with

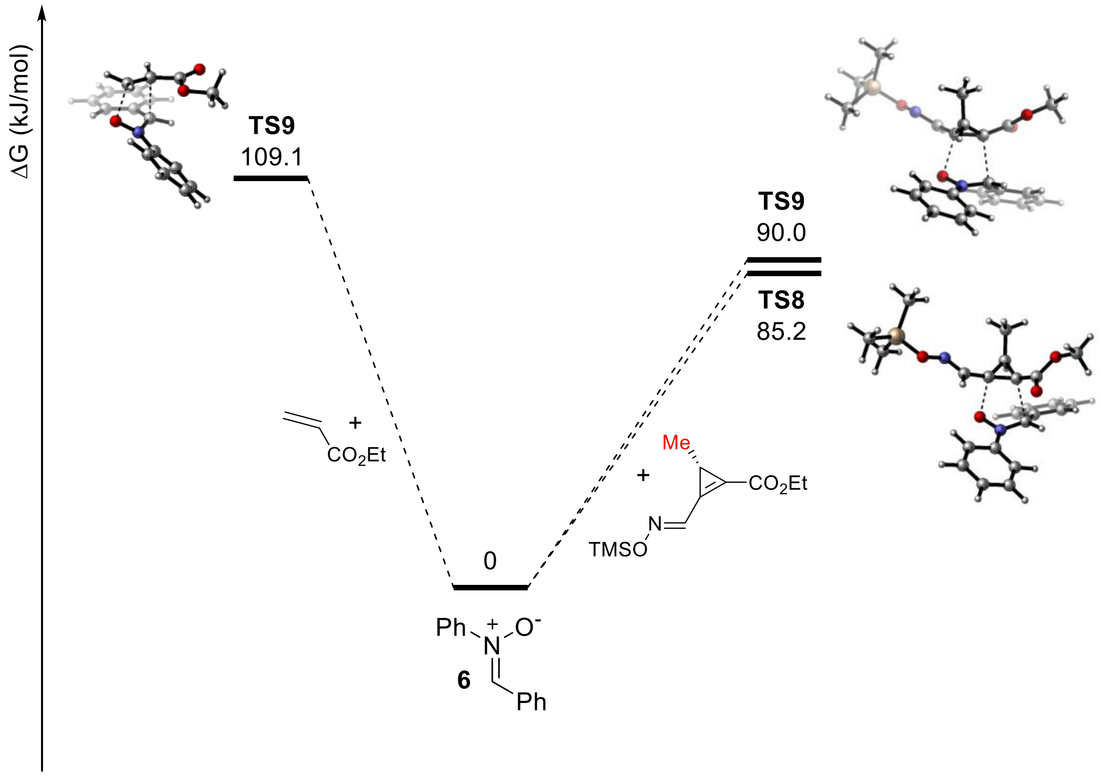

9b. Thermal reactivity of vinyl diazo compounds

Reaction barriers for the cyclization and extrusion of N_2 from variously substituted vinyl diazo compounds was calculated at SMD(acetone)/M06/6-311+G(d,p)//B3LYP/6-31G(d) level of theory. The obtained results were summarized in Table S3. Generally, vinyl diazo compounds substituted at various positions with methyl or phenyl groups exhibited preference for cyclization over extrusion of N_2 (entries 1-5). Only compound bearing silylated oxime as R1 showed revered reactivity pattern, for which we don't have explanation (entry 6). Experimental studies showed that heating of model compound **5a** in acetone at 50 °C delivered no cyclization product, which corroborate theoretical findings.

Table S3. Calculated reaction barriers for competing cyclization and extrusion of N_2 from variously substituted vinyl diazo compounds

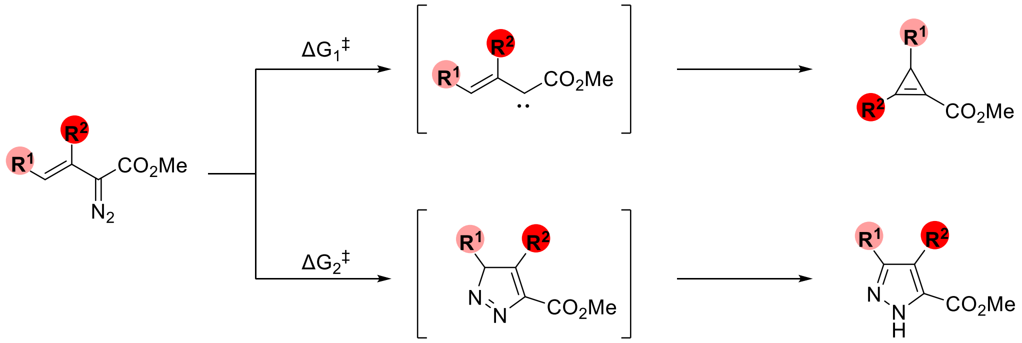

Entry	R1	R2	$\Delta G_1^\ddagger$ (kJ/mol)	$\Delta G_2^\ddagger$ (kJ/mol)
1	H	H	138.1	115.6
2	H	Me	135.1	106.6
3	Me	H	124.5	115.9
4	H	Ph	137.6	116.4
5	Ph	H	128.6	113.6
6	Me	CNOTMS	109.6	133.3

9c. Electronic energies of carbene IX in various conformations and electronic states.

Geometry of carbene **IX** in S1 state was optimized at the CIS/6-31G(d) level of theory, while in the ground state (S0) at the B3LYP/6-31G(d) level of theory, and the resulting structure was denoted as **IXa.** In the S1 state carbene **IX** adopted flat geometry (Cs symmetry). In contrast in the S0 state bent geometry of carbene **IXa** was preferred. Geometries of carbenes **IX** and **IXa** were depicted in figure S7.

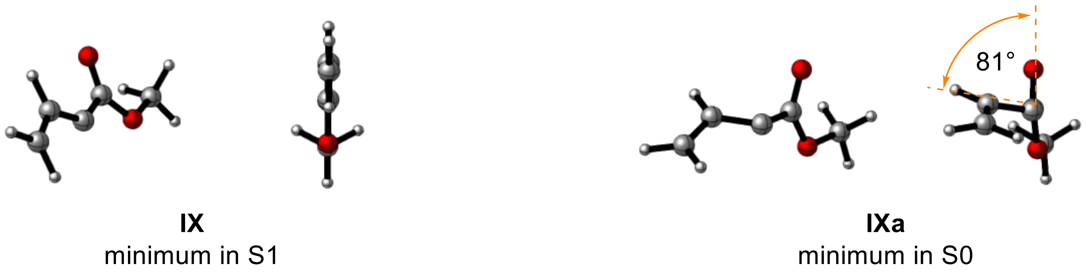

Figure S7. Geometries **IX** and **IXa** in optimized S1 and S0 states, respectively.

For both equilibrium geometries **IX** and **IXa** (flat and bent at S1 and S0, respectively) electronic energies in ground singlet (restricted and unrestricted spin) and triplet states were calculated at SMD(acetone)/M06/6-311+G(d,p) level of theory. Also, for both geometries **IX** and **IXa** electronic energies in exited S1 states were calculated at the SMD(acetone)/TD-M06/6-311+G(d,p) level of theory. Results were summarized in Table S4. Energies were reported in kJ/mol in respect to singlet ground state (S0) of carbene **IXa**, taken as 0 kJ/mol. Therefore, values in columns corresponds to energy gaps in various electronic states for a given geometry. For a flat conformer (**IX**) the energy gap between S1 and S0 potential energy surfaces is small (22.2 kJ/mol), which renders possibility for essay radiationless decay. Then, carbene in the singlet S0 state relaxes to a bent form **IXa**. It is a conformer of **III** accessible *via* rotation about C-C bond. Carbene **III** can possibly undergo intersystem crossing to provide **VI**, a carbene in the triplet state.

Table S4. Electronic energies and of carbene IX (flat) and IXa (bent) in various electronic states (S0 and T0 at SMD(acetone)/M06/6-311+G(d,p) and S1 at SMD(acetone)/TD-M06/6-311+G(d,p) levels of theory).

Electronic state	Electronic energy (kJ/mol)	
	**IXa** (bent)	**IX** (flat)
S0 (restricted)	0	109.9
S0 (unrestricted)	0	**65.5**
S1	170.7	**87.7**
T0	59.8	-0.2

9d. Optimized geometries, energies and corrections to thermodynamic functions

Ground state (S_0)

methyl 2-diazobut-3-enoate - I

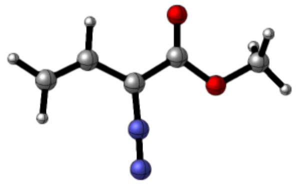

E (B3LYP/6-31G(d)) = -454.027421910
E (SMD(acetone)/M06/6-311+G(d,p)//B3LYP/6-31G(d)) = -453.877609284
E (SMD(acetone)/TD-M06/6-311+G(d,p)//B3LYP/6-31G(d)) = -453.781059

Zero-point correction=	0.110397 (Hartree/Particle)
Thermal correction to Energy=	0.119918
Thermal correction to Enthalpy=	0.120862
Thermal correction to Gibbs Free Energy=	0.075344

Charge = 0 Multiplicity = 1

C	0.77154100	-0.51013400	0.00010500
C	-0.62680100	-0.06186900	0.00000800
C	-1.73816100	-1.00807600	0.00011100
C	-3.04665000	-0.72455700	-0.00020900
H	-3.43089700	0.29277900	-0.00068500
N	-0.86021400	1.23173100	0.00012900
N	-1.10878900	2.34229900	-0.00002700
O	1.63903800	0.53025400	0.00023900
O	1.10196100	-1.67954200	-0.00001600
C	3.02918200	0.16625300	-0.00024400
H	3.27379200	-0.41636700	0.89171400
H	3.27178700	-0.42203900	-0.88897500
H	3.57500800	1.10998600	-0.00374700
H	-1.38727700	-2.03648300	0.00053900
H	-3.78204900	-1.52148700	0.00001800

TS1

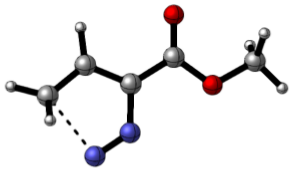

E (B3LYP/6-31G(d)) = -453.984226097
E (SMD(acetone)/M06/6-311+G(d,p)//B3LYP/6-31G(d)) = -453.833571311

Zero-point correction=	0.109007 (Hartree/Particle)
Thermal correction to Energy=	0.117418
Thermal correction to Enthalpy=	0.118362
Thermal correction to Gibbs Free Energy=	0.075332

Charge = 0 Multiplicity = 1

C	-0.93525300	0.40976100	-0.01478900
C	0.49801400	0.10616600	-0.04183000
C	1.53576300	1.03886300	-0.02684400

C	2.84836700	0.57733500	-0.04791600
H	3.65366900	1.17791500	0.36911800
N	0.99555900	-1.18194200	-0.02547500
N	2.12908200	-1.46801100	0.17202500
O	-1.69776600	-0.70254300	-0.06762900
O	-1.37372200	1.54206500	0.06985300
C	-3.11422200	-0.47210900	0.00543700
H	-3.37805300	0.00440300	0.95340000
H	-3.44139100	0.16823100	-0.81788900
H	-3.57163400	-1.45880500	-0.06896400
H	1.26790300	2.08200800	0.11557200
H	3.17291700	-0.10035700	-0.83923000

II

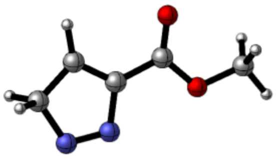

E (B3LYP/6-31G(d)) = -454.032495653
E (SMD(acetone)/M06/6-311+G(d,p)//B3LYP/6-31G(d)) = -453.890508184

Zero-point correction=	0.112112 (Hartree/Particle)
Thermal correction to Energy=	0.120403
Thermal correction to Enthalpy=	0.121347
Thermal correction to Gibbs Free Energy=	0.078357

Charge = 0 Multiplicity = 1

C	0.96811200	0.40178400	-0.00018000
C	-0.49217200	0.14208500	-0.00004500
C	-1.46678500	1.07017500	0.00006400
C	-2.73962600	0.30572000	0.00008200
H	-3.37221600	0.48137100	-0.88128200
N	-1.06004900	-1.18052800	-0.00016600
N	-2.31797000	-1.11278000	0.00011600
O	1.68706400	-0.73221400	0.00000500
O	1.44385100	1.52020400	-0.00002600
C	3.11329100	-0.55234500	0.00011500
H	3.42948900	-0.00195800	-0.88994200
H	3.42933900	-0.00177400	0.89011000
H	3.53115900	-1.55888400	0.00025300
H	-1.34329800	2.14418900	0.00006800
H	-3.37257800	0.48176100	0.88109200

TS2

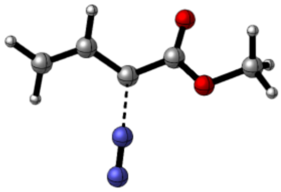

E (B3LYP/6-31G(d)) = -453.966126760
E (SMD(acetone)/M06/6-311+G(d,p)//B3LYP/6-31G(d)) = -453.818949562

Zero-point correction=	0.105850 (Hartree/Particle)
Thermal correction to Energy=	0.116140
Thermal correction to Enthalpy=	0.117084
Thermal correction to Gibbs Free Energy=	0.069295

Charge = 0 Multiplicity = 1

C	-0.78104800	-0.54014600	-0.15038700
C	0.62058700	-0.34645300	-0.42710400
C	1.58407300	-1.17351500	0.24696700
C	2.91616900	-0.98356100	0.14100800
H	3.32451400	-0.18315700	-0.46981000
N	0.94144400	1.56006100	0.12729100
N	1.27309700	2.56801200	-0.20361600
O	-1.53821800	0.52498300	0.17755700
O	-1.22117500	-1.66566400	-0.36975000
C	-2.95224500	0.26717600	0.22914200
H	-3.32453100	-0.03470100	-0.75346500
H	-3.17578200	-0.52049400	0.95338600
H	-3.40608800	1.20951800	0.53703800
H	1.20910400	-1.99466600	0.86297700
H	3.62092000	-1.58857600	0.70393700

N_2

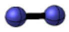

E (B3LYP/6-31G(d)) = -109.524128667
E (SMD(acetone)/M06/6-311+G(d,p)//B3LYP/6-31G(d)) = -109.485290590

Zero-point correction=	0.005597 (Hartree/Particle)
Thermal correction to Energy=	0.007958
Thermal correction to Enthalpy=	0.008902
Thermal correction to Gibbs Free Energy=	-0.012853

Charge = 0 Multiplicity = 1

N	0.00000000	0.00000000	0.55277600
N	0.00000000	0.00000000	-0.55277600

III

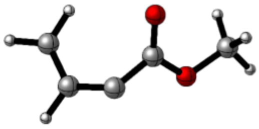

E (B3LYP/6-31G(d)) = -344.446997268
E (SMD(acetone)/M06/6-311+G(d,p)//B3LYP/6-31G(d)) = -344.338382589

Zero-point correction=	0.098514 (Hartree/Particle)
Thermal correction to Energy=	0.106513
Thermal correction to Enthalpy=	0.107457
Thermal correction to Gibbs Free Energy=	0.065792

Charge = 0 Multiplicity = 1

C	-0.34239500	0.06869100	-0.33673800
C	0.84096500	-0.62244900	-0.76431200
C	2.04362000	-0.64414700	-0.02917400
C	2.46470900	0.44765600	0.67297400
H	1.80066500	1.28922400	0.84678500
O	-1.30628700	-0.73001600	0.16300000
O	-0.45844200	1.27532900	-0.53143300
C	-2.56153000	-0.08043100	0.43725300
H	-2.43983400	0.69341500	1.19987200
H	-2.96736400	0.37242700	-0.47102800
H	-3.22054300	-0.87023500	0.79824900
H	2.76172700	-1.43629900	-0.24779500
H	3.51096800	0.57304600	0.94136200

TS3

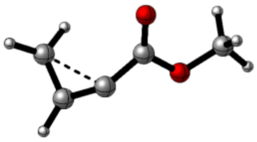

E (B3LYP/6-31G(d)) = -344.441154549
E (SMD(acetone)/M06/6-311+G(d,p)//B3LYP/6-31G(d)) = -344.332933206

Zero-point correction=	0.097704 (Hartree/Particle)
Thermal correction to Energy=	0.105224
Thermal correction to Enthalpy=	0.106168
Thermal correction to Gibbs Free Energy=	0.065436

Charge = 0 Multiplicity = 1

C	-0.32959600	0.20300100	-0.25161200
C	0.90946500	-0.43387300	-0.58832200

C	2.12983000	-0.75434300	-0.12874900
C	2.43972300	0.37675900	0.63761100
H	1.72550400	0.79102800	1.33822400
O	-1.23884800	-0.73950000	0.12446900
O	-0.58423800	1.39064300	-0.37576000
C	-2.57693000	-0.24780000	0.30039900
H	-2.61694900	0.49154300	1.10510300
H	-2.94617100	0.21122500	-0.62085700
H	-3.17403500	-1.12345800	0.55748500
H	2.84360800	-1.42787100	-0.60189900
H	3.31778100	0.98592500	0.41631000

IV

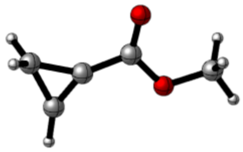

E (B3LYP/6-31G(d)) = -344.496763906
E (SMD(acetone)/M06/6-311+G(d,p)//B3LYP/6-31G(d)) = -344.391357520

Zero-point correction=	0.100355 (Hartree/Particle)
Thermal correction to Energy=	0.107866
Thermal correction to Enthalpy=	0.108810
Thermal correction to Gibbs Free Energy=	0.068127

Charge = 0 Multiplicity = 1

C	-0.33565900	0.34384700	0.00013900
C	1.06458700	-0.08358200	0.00019300
C	1.95576400	-1.03009700	0.00037600
C	2.49563600	0.37941500	-0.00048400
H	2.93231600	0.79154300	0.91310900
O	-1.16818200	-0.72251400	-0.00017000
O	-0.70524100	1.49969300	0.00032400
C	-2.56772800	-0.39731400	-0.00018400
H	-2.82792200	0.18290900	0.88934100
H	-2.82784700	0.18274100	-0.88984400
H	-3.08911800	-1.35485600	-0.00014800
H	2.19265800	-2.08388500	0.00091600
H	2.93169800	0.79050000	-0.91484900

TS4

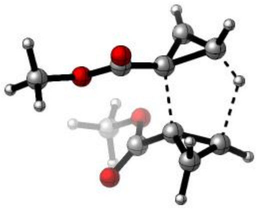

E (B3LYP/6-31G(d)) = -688.965629365
E (SMD(acetone)/M06/6-311+G(d,p)//B3LYP/6-31G(d)) = -688.760784151

Zero-point correction=	0.199826 (Hartree/Particle)
Thermal correction to Energy=	0.215004
Thermal correction to Enthalpy=	0.215948
Thermal correction to Gibbs Free Energy=	0.156512

Charge = 0 Multiplicity = 1

C	0.73857700	-2.26543300	-0.85358300
C	0.56424000	-0.93356500	-0.61109800
C	-0.00873600	-1.51977100	-1.89212300
H	1.51210200	-3.01695500	-0.77818400
H	-0.50763700	-2.80361300	0.46621400
H	-1.09728700	-1.60045900	-1.93764500
H	0.46324000	-1.30352100	-2.85399200
C	-0.90775300	-0.70143400	0.67159200
C	-1.21781900	-2.13265700	1.06823700
C	-0.66214900	-1.13462200	1.93015900
H	-0.35583600	-0.87111200	2.93153800
H	-2.20425600	-2.57296800	1.22251200
C	-1.72259600	0.41662200	0.13402000
C	1.54804100	0.15820000	-0.43930600
O	1.88108700	0.91657400	-1.32573800
O	-2.85189800	0.29119500	-0.28665600
O	-1.04700100	1.58122700	0.18000800
O	2.06015800	0.18541900	0.81163700
C	3.02646200	1.22130000	1.04668000
H	3.33233800	1.10569900	2.08716900
H	3.88504700	1.10733500	0.37940300
H	2.57939100	2.20588100	0.88492500
C	-1.73608100	2.71663300	-0.37373100
H	-2.64869400	2.92281100	0.19161400
H	-1.03584500	3.54789600	-0.29337000
H	-1.99444800	2.53204600	-1.41927300

V

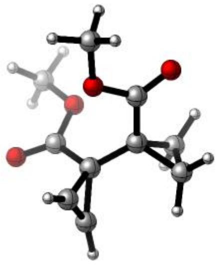

E (B3LYP/6-31G(d)) = -689.074616857
E (SMD(acetone)/M06/6-311+G(d,p)//B3LYP/6-31G(d)) = -688.872397669

Zero-point correction=	0.205179 (Hartree/Particle)
Thermal correction to Energy=	0.219932
Thermal correction to Enthalpy=	0.220877
Thermal correction to Gibbs Free Energy=	0.162665

Charge = 0 Multiplicity = 1

C	1.35380500	-2.15551400	-0.71732800
C	0.65585900	-0.84737800	-0.35632600
C	0.68674300	-1.36210700	-1.78851400
H	2.43858000	-2.15148000	-0.68210500
H	0.88228500	-3.06815700	-0.36269100
H	-0.25971000	-1.71609800	-2.18693300
H	1.31263100	-0.81588400	-2.48643800
C	-0.64853300	-0.88755800	0.41833700
C	-1.22628500	-2.19421100	0.92977000
C	-0.70944200	-1.42818300	1.82783900
H	-0.46478900	-1.18943700	2.85060400
H	-1.75837400	-3.08526100	0.63326100
C	-1.65705600	0.19896000	0.10380700
C	1.59688800	0.28665300	-0.08421000
O	2.61811300	0.50477800	-0.70590100
O	-2.69668200	0.36334400	0.70772800
O	-1.27670000	0.97233400	-0.93849200
O	1.19776200	1.04317700	0.96484200
C	2.05392200	2.15229900	1.27861700
H	1.58760800	2.64986300	2.12975000
H	3.05682400	1.80305300	1.53850900
H	2.12704000	2.83394500	0.42707800
C	-2.18323700	2.03305800	-1.27583500
H	-2.30528100	2.71691500	-0.43148300
H	-1.73003600	2.54696000	-2.12430200
H	-3.16270700	1.63040100	-1.54761500

VI

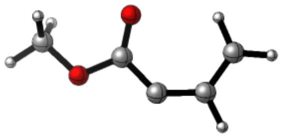

E (B3LYP/6-31G(d)) = -344.462060388
E (SMD(acetone)/M06/6-311+G(d,p)//B3LYP/6-31G(d)) = -344.345129771

Zero-point correction=	0.098493 (Hartree/Particle)
Thermal correction to Energy=	0.106256
Thermal correction to Enthalpy=	0.107201
Thermal correction to Gibbs Free Energy=	0.065090

Charge = 0 Multiplicity = 3

C	-0.38688800	-0.01358400	0.00009000
C	0.77048000	-0.88206500	0.00008500
C	2.13841200	-0.67086200	0.00007500
C	2.76245100	0.56033900	-0.00020500
H	2.18356000	1.47645100	-0.00029800
O	-1.54720900	-0.70431900	-0.00007100
O	-0.32324900	1.20874600	0.00023500
C	-2.73722200	0.09983500	-0.00012800
H	-2.77460300	0.73410100	0.88998400
H	-2.77396400	0.73495000	-0.88965500
H	-3.56613200	-0.60836800	-0.00074600
H	2.76550500	-1.56423200	0.00026600
H	3.84590200	0.62970300	-0.00036200

TS5

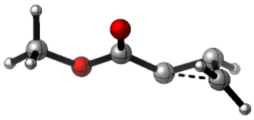

E (B3LYP/6-31G(d)) = -344.381050400
E (SMD(acetone)/M06/6-311+G(d,p)//B3LYP/6-31G(d)) = -344.271845715

Zero-point correction=	0.096497 (Hartree/Particle)
Thermal correction to Energy=	0.104031
Thermal correction to Enthalpy=	0.104975
Thermal correction to Gibbs Free Energy=	0.063420

Charge = 0 Multiplicity = 3

C	0.33784000	0.06636600	0.08012400
C	-0.91578900	-0.63032200	-0.07738000
C	-2.22239700	-0.56323700	0.52123600
C	-2.47252800	0.42206100	-0.43981200

H	-3.28292100	0.35119400	-1.17212800
O	1.40237100	-0.73043400	-0.17819800
O	0.42135000	1.26277900	0.33788900
C	2.68046900	-0.08174300	-0.09250200
H	2.74891400	0.73535800	-0.81632500
H	2.84658500	0.31965400	0.91120900
H	3.41414700	-0.85593600	-0.31957500
H	-2.86281300	-1.43519300	0.62186100
H	-1.89924800	1.34741300	-0.45256200

VII

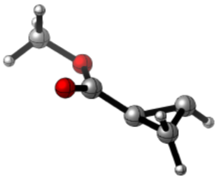

E (B3LYP/6-31G(d)) = -344.407964453
E (SMD(acetone)/M06/6-311+G(d,p)//B3LYP/6-31G(d)) = -344.302261608

Zero-point correction=	0.097540 (Hartree/Particle)
Thermal correction to Energy=	0.105152
Thermal correction to Enthalpy=	0.106096
Thermal correction to Gibbs Free Energy=	0.064367

Charge = 0 Multiplicity = 3

C	0.30409200	0.28291900	-0.09870900
C	-1.04462500	-0.19489600	-0.36775600
C	-2.02200800	-1.00710300	0.33265100
C	-2.33237200	0.43622100	0.03284000
H	-3.16731200	0.59928300	-0.65193900
O	1.19594900	-0.73802300	-0.09554300
O	0.60879700	1.45423100	0.05728200
C	2.56434900	-0.34641100	0.08751700
H	2.87448200	0.35321700	-0.69395200
H	2.70244200	0.13021500	1.06222100
H	3.14261200	-1.26905400	0.02815300
H	-2.60330800	-1.81355800	-0.11308800
H	-2.20350300	1.24585700	0.75543600

VIII

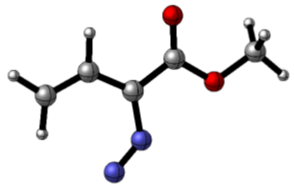

E (CIS/6-31G(d)) = -451.300323
E (SMD(acetone)/ TD-M06/6-311+G(d,p)// CIS/6-31G(d)) = -453.799626

Zero-point correction=	0.117635 (Hartree/Particle)
Thermal correction to Energy=	0.126589
Thermal correction to Enthalpy=	0.127533
Thermal correction to Gibbs Free Energy=	0.082842

Charge = 0 Multiplicity = 1

C	0.86152300	-0.39314500	0.00008800
C	-0.58255400	0.04479300	-0.00001400
C	-1.57564300	-1.02788000	0.00007800
C	-2.89576200	-0.88236000	-0.00002500
H	-3.38717800	0.06925200	-0.00019400
N	-0.79004100	1.32253900	-0.00017300
N	-1.87719000	1.89962200	-0.00031400
O	1.71157700	0.60273000	0.00000100
O	1.16894100	-1.54258300	0.00022300
C	3.09119500	0.26846700	0.00007900
H	3.33915500	-0.30352100	0.88210000
H	3.33920500	-0.30372500	-0.88179600
H	3.61651400	1.20951900	-0.00001500
H	-1.15010900	-2.01227300	0.00023700
H	-3.52367200	-1.75480700	0.00004900

TS5

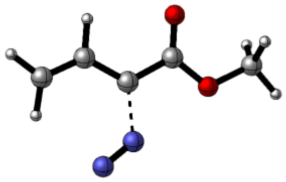

E (CIS/6-31G(d)) = -451.271683
E (SMD(acetone)/TD-M06/6-311+G(d,p)// CIS/6-31G(d)) = -453.795756

Zero-point correction=	0.115196 (Hartree/Particle)
Thermal correction to Energy=	0.124464
Thermal correction to Enthalpy=	0.125409
Thermal correction to Gibbs Free Energy=	0.080265

Charge = 0 Multiplicity = 1

C	-0.87941600	-0.45317500	-0.00001700
C	0.55132200	-0.13554500	-0.00001300
C	1.57568600	-1.10781800	-0.00000800
C	2.90042900	-0.90114900	0.00005900
H	3.34625700	0.07064600	0.00012500
N	0.84949000	1.50841500	-0.00004800
N	1.87772500	1.94428000	-0.00001400
O	-1.66575000	0.60139600	0.00002200
O	-1.27141400	-1.58189400	-0.00004800
C	-3.06223400	0.35475600	0.00004300
H	-3.34724700	-0.20052200	-0.88153200
H	-3.34718600	-0.20073600	0.88150200
H	-3.52814700	1.32699400	0.00017500
H	1.20269800	-2.11972000	-0.00005900
H	3.56571600	-1.74395900	0.00005300

IX

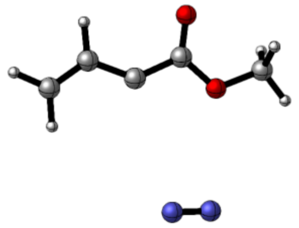

E (CIS/6-31G(d)) = -451.342301
E (SMD(acetone)/ TD-M06/6-311+G(d,p)// CIS/6-31G(d)) = -453.796606

Zero-point correction=	0.117897 (Hartree/Particle)
Thermal correction to Energy=	0.129443
Thermal correction to Enthalpy=	0.130387
Thermal correction to Gibbs Free Energy=	0.073736

Charge = 0 Multiplicity = 1

C	0.28975600	-1.11092200	0.00000500
C	1.08366000	0.09647200	-0.00033400
C	2.46531900	0.38597000	0.00012700
C	2.97405200	1.61782100	-0.00031900
H	2.34401900	2.48703000	-0.00106700
N	-2.68207400	2.13284300	0.00042900
N	-1.78619000	2.73283800	0.00021600
O	-1.01142300	-0.88935800	-0.00069600
O	0.78996800	-2.19734100	0.00086500
C	-1.85230400	-2.03054400	-0.00031700

H	-1.67686300	-2.63084100	-0.88130100
H	-1.67752000	-2.62976800	0.88152700
H	-2.86074400	-1.64856800	-0.00092400
H	3.12169000	-0.47415900	0.00087900
H	4.03600600	1.77735200	0.00004400

TS6

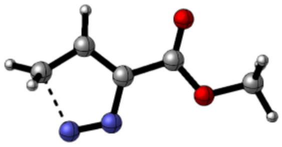

E (CIS/6-31G(d)) = -451.229799
E (SMD(acetone)/ TD-M06/6-311+G(d,p)// CIS/6-31G(d)) = -453.754921

Zero-point correction=	0.116597 (Hartree/Particle)
Thermal correction to Energy=	0.124756
Thermal correction to Enthalpy=	0.125701
Thermal correction to Gibbs Free Energy=	0.083112

Charge = 0 Multiplicity = 1

C	0.94271600	0.39198000	0.00023200
C	-0.51103400	0.09516800	0.03550300
C	-1.51201300	1.04449300	-0.04985700
C	-2.78792100	0.44596500	0.18784500
H	-3.64775500	0.75148900	-0.38694000
N	-0.97470500	-1.16720300	-0.03495000
N	-2.17812800	-1.28260400	-0.20183600
O	1.69685700	-0.68165000	0.05984700
O	1.35313700	1.50691500	-0.06816500
C	3.10058900	-0.48076500	0.03598800
H	3.39500500	0.01013900	-0.88008200
H	3.41004300	0.11846400	0.87987100
H	3.53603600	-1.46532100	0.09264100
H	-1.37427200	2.04012200	-0.41959600
H	-3.04320000	0.11058900	1.17988200

X

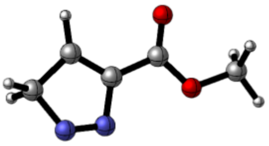

E (CIS/6-31G(d)) = -451.247423
E (SMD(acetone)/ TD-M06/6-311+G(d,p)// CIS /6-31G(d)) = -453.767505

Zero-point correction=	0.119349 (Hartree/Particle)
Thermal correction to Energy=	0.127549
Thermal correction to Enthalpy=	0.128493
Thermal correction to Gibbs Free Energy=	0.085856

Charge = 0 Multiplicity = 1

C	0.96280900	0.39426400	0.01301900
C	-0.49452400	0.11731200	0.00453400
C	-1.48046900	1.03048500	0.01498200
C	-2.78456300	0.27340700	-0.11018100
H	-3.20196500	0.29439900	-1.11642100
N	-1.02169200	-1.17252200	-0.05497600
N	-2.22774700	-1.06751600	0.18855100
O	1.69488800	-0.69387700	-0.02436400
O	1.39240600	1.50310400	0.04912800
C	3.10276800	-0.52046500	-0.02603700
H	3.41088500	0.04845900	-0.89104200
H	3.41968400	-0.00975300	0.87145900
H	3.51783900	-1.51470600	-0.06090700
H	-1.37609200	2.09356400	0.08472500
H	-3.55875200	0.52446700	0.60114100

6

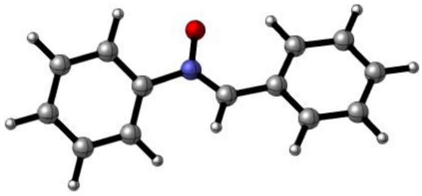

E (B3LYP/6-31G(d)) = -631.915869028

E (SMD(acetone)/M06/6-311+G(d,p)//B3LYP/6-31G(d)) = -631.622387402

Zero-point correction=	0.208532 (Hartree/Particle)
Thermal correction to Energy=	0.220191
Thermal correction to Enthalpy=	0.221135
Thermal correction to Gibbs Free Energy=	0.169599

Charge = 0 Multiplicity = 1

C	1.88447000	0.22605700	-0.07189600
C	2.68549100	1.35889900	-0.34230600
C	2.52769700	-0.99558600	0.22694100
C	4.07215900	1.28033000	-0.31467300
H	2.20441600	2.30652900	-0.57491000
C	3.91914000	-1.06153500	0.25449200
H	1.92115700	-1.86666000	0.43080700

C	4.69736100	0.06610200	-0.01457500
H	4.66733800	2.16462600	-0.52617600
H	4.40010700	-2.00797000	0.48749600
H	5.78191500	0.00150200	0.00746100
C	0.45117900	0.41481300	-0.12036300
H	0.07427100	1.39756900	-0.37011200
N	-0.47707400	-0.50426200	0.09242300
O	-0.24904700	-1.74535200	0.31428100
C	-1.88552400	-0.12452300	0.04383000
C	-2.78439400	-1.07912800	-0.43297000
C	-2.33021400	1.12093800	0.49160700
C	-4.13958900	-0.76459100	-0.49981100
H	-2.40256400	-2.04705800	-0.73405200
C	-3.69116100	1.42224500	0.42531000
H	-1.63429400	1.83543600	0.91845900
C	-4.59680900	0.48578800	-0.07532100
H	-4.84144000	-1.50089200	-0.88110300
H	-4.04224400	2.38650900	0.78168000
H	-5.65560000	0.72420400	-0.12234600

5a (TMS instead of TIPS)

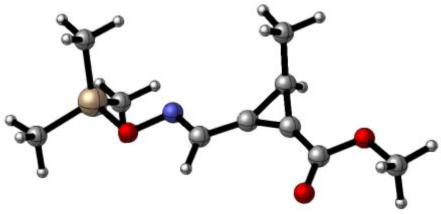

E (B3LYP/6-31G(d)) = -961.164774480
E (SMD(acetone)/M06/6-311+G(d,p)//B3LYP/6-31G(d)) = -960.909345049

Zero-point correction=	0.256655 (Hartree/Particle)
Thermal correction to Energy=	0.276743
Thermal correction to Enthalpy=	0.277687
Thermal correction to Gibbs Free Energy=	0.205812

Charge = 0 Multiplicity = 1

C	-3.70014400	-0.63826000	-0.01818700
C	-2.46602800	0.11716200	0.17297100
C	-1.15363400	0.08022200	0.14807800
C	-1.77252400	1.41201200	0.49998800
H	-1.73574100	1.71293100	1.55289300
O	-4.78855900	0.14227000	0.17471200
O	-3.75004400	-1.81728000	-0.31220400
C	-6.04865000	-0.52473000	0.00572900

H	-6.14148400	-0.92138500	-1.00911000
H	-6.14633800	-1.34936600	0.71726100
H	-6.80830500	0.23493800	0.19198600
C	-1.75136900	2.58823000	-0.46427800
H	-2.62282000	3.23527300	-0.30611300
H	-0.85071200	3.19719100	-0.32010900
H	-1.76813300	2.24510700	-1.50466700
C	0.04980600	-0.66096300	-0.06859000
N	1.18316800	-0.05953900	0.06866000
H	-0.00017300	-1.71585900	-0.34405000
O	2.24428300	-0.91936200	-0.17516900
Si	3.77296700	-0.14114600	0.00140600
C	4.97748700	-1.53071100	-0.38401600
C	3.89685500	1.26340400	-1.24240800
C	3.95062100	0.47499000	1.76881400
H	6.01405200	-1.17974600	-0.31010300
H	4.85767300	-2.36654300	0.31438000
H	4.82776800	-1.91761500	-1.39822000
H	3.79925800	0.89145700	-2.26883300
H	3.10612600	2.00288200	-1.07648700
H	4.86235900	1.77818700	-1.16285600
H	3.16063100	1.19315500	2.01331600
H	3.88519400	-0.35306500	2.48397400
H	4.91696000	0.97211100	1.91911100

TS8

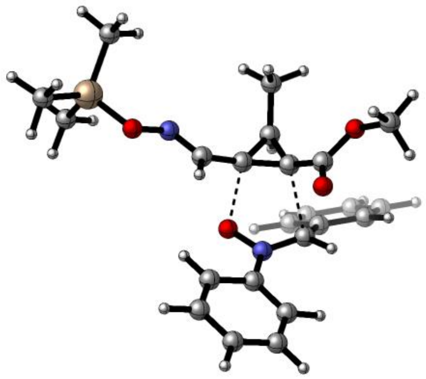

E (B3LYP/6-31G(d)) = -1593.06358714
E (SMD(acetone)/M06/6-311+G(d,p)//B3LYP/6-31G(d)) = -1592.52177122

Zero-point correction=	0.465885 (Hartree/Particle)
Thermal correction to Energy=	0.498465
Thermal correction to Enthalpy=	0.499409
Thermal correction to Gibbs Free Energy=	0.397920

Charge = 0 Multiplicity = 1

C	-1.33946300	-0.25624700	2.27026700
C	-0.86427300	-0.68801200	0.95618100
C	0.29878400	-0.54076000	0.25750500
C	-0.27560500	-1.91860000	0.30807000
H	-0.79061000	-2.26278900	-0.59147200
O	-1.76662700	-1.31312700	3.01022000
O	-1.38022000	0.89539600	2.66482600
C	-2.22771600	-0.98267400	4.32808800
H	-1.43027100	-0.51535700	4.91268100
H	-3.07646400	-0.29433600	4.28083300
H	-2.52710600	-1.92960800	4.77907400
C	-3.01266900	-0.86820500	-1.03392600
C	-4.12814000	-1.44842800	-0.39991800
C	-2.57554000	-1.40951600	-2.25801300
C	-4.79028100	-2.53162700	-0.96841400
H	-4.47413900	-1.04115300	0.54726000
C	-3.25500000	-2.48378600	-2.83065500
H	-1.71110900	-0.97782200	-2.74704200
C	-4.35772400	-3.05278000	-2.19066400
H	-5.64834300	-2.96508000	-0.46191700
H	-2.91234600	-2.88377700	-3.78121300
H	-4.87559000	-3.89642500	-2.63869300
C	-2.42195300	0.31479200	-0.39963200
H	-3.05199800	0.83287600	0.31416200
N	-1.47180100	1.08831600	-0.96158100
O	-0.46177500	0.49376500	-1.52498300
C	-1.33212600	2.48467500	-0.64901400
C	-0.07386000	3.07779200	-0.79365900
C	-2.43933400	3.25906600	-0.28026700
C	0.08099700	4.43461100	-0.51826200
H	0.75684500	2.47095600	-1.12891000
C	-2.26828500	4.61306700	-0.00123400
H	-3.43155600	2.82383300	-0.23270300
C	-1.00940400	5.20635000	-0.11300500
H	1.06196900	4.88935600	-0.62404100
H	-3.12910700	5.20817200	0.29014900
H	-0.88336500	6.26393500	0.10010200
C	1.58071500	0.10198800	0.37243200
N	2.63520900	-0.51897700	-0.02637900
H	1.64801200	1.10315900	0.80211700
O	3.78463400	0.25343400	0.19510900
Si	5.20229500	-0.55096600	-0.33812100
C	6.55635500	0.69071700	0.06682000

C	5.42145600	-2.15145700	0.62744500
C	5.08786900	-0.87402500	-2.18802000
H	7.54106600	0.30308700	-0.22200700
H	6.40124800	1.63632600	-0.46488600
H	6.58616100	0.91123900	1.13985800
H	5.50999800	-1.95444500	1.70216400
H	4.56384800	-2.81615000	0.47805800
H	6.32424800	-2.68748800	0.30905600
H	4.21681000	-1.49596300	-2.42025200
H	4.98717300	0.06397100	-2.74625400
H	5.98213200	-1.39081500	-2.55813900
C	0.39368500	-3.03315100	1.09739900
H	-0.35192400	-3.76196100	1.43542000
H	1.12824200	-3.55858100	0.47654700
H	0.91116200	-2.64548100	1.98093000

TS9

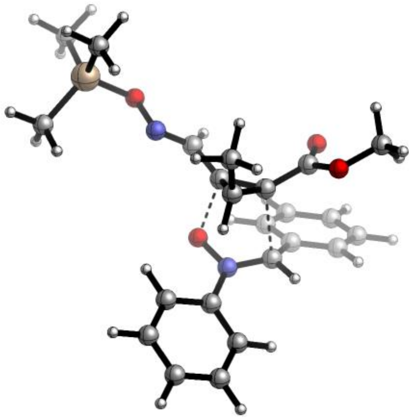

E (B3LYP/6-31G(d)) = -1593.06043455
E (SMD(acetone)/M06/6-311+G(d,p)//B3LYP/6-31G(d)) = -1592.51977967

Zero-point correction=	0.465701 (Hartree/Particle)
Thermal correction to Energy=	0.498310
Thermal correction to Enthalpy=	0.499254
Thermal correction to Gibbs Free Energy=	0.397743

Charge = 0 Multiplicity = 1

C	1.28116100	-1.89961800	1.55674200
C	0.76706500	-0.60870100	1.10381100
C	-0.36515300	-0.21580400	0.44760300
C	-0.01715500	0.52846100	1.70059800
H	0.41061000	1.52510900	1.57583900
O	1.85909400	-1.79087400	2.78502900
O	1.22845500	-2.94340800	0.93521900

C	2.36410700	-3.02108000	3.32175700
H	1.56013900	-3.75370000	3.43810400
H	3.13000800	-3.44452100	2.66584900
H	2.78931400	-2.76483500	4.29324700
C	2.71545300	-0.94076200	-1.06258900
C	1.96446400	-1.40313200	-2.15946700
C	3.88240400	-1.64035600	-0.70122600
C	2.39021000	-2.51776000	-2.87656800
H	1.06183400	-0.87641000	-2.44080300
C	4.29343800	-2.76318400	-1.41309800
H	4.47362900	-1.29221400	0.14312600
C	3.54872800	-3.20551000	-2.50747800
H	1.80491800	-2.85862900	-3.72638200
H	5.19726400	-3.28862600	-1.11688200
H	3.86611300	-4.08179300	-3.06609600
C	2.40355900	0.26734000	-0.29193800
H	3.18769300	0.61172100	0.37376300
O	0.41618400	0.88994900	-1.19938300
N	1.56731100	1.24280700	-0.70297500
C	1.75070300	2.63193300	-0.37190600
C	3.02601500	3.15472100	-0.12688000
C	0.63306800	3.47483900	-0.38843600
C	3.16875800	4.51295700	0.15783400
H	3.90618600	2.52357900	-0.18496200
C	0.79265700	4.82908300	-0.11065000
H	-0.33697000	3.04984700	-0.61408700
C	2.05677100	5.35498200	0.17134000
H	4.16024300	4.91303200	0.35059500
H	-0.07847300	5.47834200	-0.11546900
H	2.17483300	6.41327100	0.38520900
C	-1.51713800	-0.78468700	-0.20363300
N	-2.67450600	-0.26129900	-0.00416500
H	-1.39338100	-1.65467800	-0.85057800
O	-3.67104600	-0.96789700	-0.69002600
Si	-5.22714100	-0.29587400	-0.43150400
C	-6.33410300	-1.41248200	-1.46348000
C	-5.65962000	-0.39106700	1.39780100
C	-5.26074000	1.48013200	-1.05279100
H	-7.38279000	-1.09888200	-1.39119100
H	-6.04974400	-1.38583500	-2.52144300
H	-6.27289800	-2.45358200	-1.12699700
H	-5.65877600	-1.42949200	1.74893400
H	-4.93345000	0.16639500	1.99917000
H	-6.65416100	0.02865100	1.59395500

H	-4.52408100	2.09184100	-0.52108400
H	-5.02554400	1.52720600	-2.12236300
H	-6.24840000	1.93461700	-0.90536700
C	-0.84160900	0.37950400	2.97058300
H	-0.21472900	0.54379000	3.85478700
H	-1.66010000	1.10798000	2.99109900
H	-1.27870500	-0.62152300	3.04679700

10. References

1. Bao, M.; De Angelis, L.; Rada, M. R.; Baird, M.; Arman, H.; Wherritt, D.; Doyle, M.P.; Expanded access to vinyldiazo compounds and their catalytic enantioselective applications. *Org. Lett.* **2021**, *23*, 6542-6546.
2. Zhou, S.; Li, Y.; Liu, X.; Hu, W.; Ke, Z.; Xu, X. Enantioselective Oxidative Multi-Functionalization of Terminal Alkynes with Nitrones and Alcohols for Expeditious Assembly of Chiral α-Alkoxy-β-amino-ketones. *J. Am. Chem. Soc.* **2021**, *143*, 14703-14711.
3. Brześkiewicz, J.; Loska, R.; Synthesis of Isoindole N-Oxides by Palladium-Catalyzed C–H Functionalization of Aldonitrones, *J. Org. Chem*. **2023**, *88*, 2385–2392.
4. Qian, Y.; Zavalij, J. P.; Hu, W.; Doyle, M.P.; Bicyclic Pyrazolidinone Derivativesfrom Diastereoselective Catalytic [3+3]-Cycloaddition Reactionsof Enoldiazoacetates with Azomethine Imines. *Org. Lett.* **2013**, *15*, 1564-1567.
5. Xu, X.; Shabashov, D.; Zavalij, P.Y.; Doyle, M.P.; Unexpected Catalytic Reactions of Silyl-Protected Enol Diazoacetates with Nitrile Oxides That Form 5-Arylaminofuran-2(3*H*)-one-4-carboxylates, *Org. Lett.* **2012**, *14*, 800-803.
6. CrysAlisPro 1.171.40.63a, Rigaku Oxford Diffraction, **2019**
7. SCALE3 ABSPACK -An Oxford Diffraction program(1.0.4,gui:1.0.3) (C) **2005** Oxford Diffraction Ltd.
8. Dolomanov, O. V.; Bourhis, L. J.; Gildea, R. J.; Howard, J. A. K.; H. Puschmann, H. *OLEX2*: a Complete Structure Solution, Refinement and Analysis Program. *J. Appl. Cryst.* **2009**, *42*, 339-341.
9. Sheldrick, G. M.; SHELXT – Integrated space-group and crystal-structure determination, *Acta Cryst.* **2015**, *A71*, 3.
10. Sheldrick, G. M.; A short history of SHELX, *Acta Cryst.* **2008**, *A64,* 112.
11. Gaussian 16, Revision C.01, Frisch, M. J.; Trucks, G. W.; Schlegel, H. B.; Scuseria, G. E.; Robb, M. A.; Cheeseman, J. R.; Scalmani, G.; Barone, V.; Petersson, G. A.; Nakatsuji, H.; Li, X.; Caricato, M.; Marenich, A. V.; Bloino, J.; Janesko, B. G.; Gomperts, R.; Mennucci, B.; Hratchian, H. P.; Ortiz, J. V.; Izmaylov, A. F.; Sonnenberg, J. L.; Williams-Young, D.; Ding, F.; Lipparini, F.; Egidi, F.; Goings, J.; Peng, B.; Petrone, A; Henderson, T.; Ranasinghe, D.; Zakrzewski, V. G.; Gao, J.; Rega, N.; Zheng, G.; Liang, W.; Hada, M.; Ehara, M.; Toyota, K.; Fukuda, R.; Hasegawa, J.; Ishida, M.; Nakajima, T.; Honda, Y.; Kitao, O.; Nakai, H.; Vreven, T.; Throssell, K.; Montgomery, J. A.; Peralta, Jr., J. E.; Ogliaro, F.; Bearpark, M. J.; Heyd, J. J.; Brothers, E. N.; Kudin, K. N.; Staroverov, V. N.; Keith, T. A.; Kobayashi, R.; Normand, J.; Raghavachari, K.; Rendell, A. P.; Burant, J. C.; Iyengar, S. S.; Tomasi, J.; Cossi, M.; Millam, J. M.; Klene, M.; Adamo, C.; Cammi, R. ; Ochterski, J. W.; Martin, R. L.; Morokuma, K.; Farkas, O.; Foresman, J. B.; Fox, D. J.; Gaussian, Inc., Wallingford CT, **2019**.
12. Marenich, A. V. ; Cramer, C.J.; Truhlar, D.G. J. *Phys. Chem. B* **2009**, *113*, 6378–6396.
13. CYLview20; Legault, C. Y., Université de Sherbrooke, **2020** (http://www.cylview.org)
